# Supplementary material for: Doubly linked chiral phenanthrene oligomers for homogeneously π-extended helicenes with large effective conjugation length
Source: Nat Commun. 2022 Apr 4;13:1475. doi: 10.1038/s41467-022-29108-8 (PMC8980098; doi:10.1038/s41467-022-29108-8)
Supplement: Supplementary file 1 — Supplementary Information [file 41467_2022_29108_MOESM1_ESM.pdf]

Supplementary Information

Doubly linked chiral phenanthrene oligomers for homogeneously π-extended helicenes with large effective conjugation length

Yusuke Nakakuki,[1] Takashi Hirose,[2,3,*] Hikaru Sotome,[4] Min Gao,[5] Daiki Shimizu,[1] Ruiji Li,[1,6] Jun-ya Hasegawa,[5] Hiroshi Miyasaka,[4] and Kenji Matsuda[1,*]

[1]Department of Synthetic Chemistry and Biological Chemistry, Graduate School of Engineering, Kyoto University, Katsura, Nishikyo-ku, Kyoto 615-8510, Japan

[2]Institute for Chemical Research, Kyoto University, Uji, Kyoto 611-0011, Japan

[3]PRESTO, Japan Science and Technology Agency (JST), 4-1-8 Honcho, Kawaguchi, Saitama 332-0012, Japan

[4]Division of Frontier Materials Science and Center for Promotion of Advanced Interdisciplinary Research, Graduate School of Engineering Science, Osaka University, Toyonaka, Osaka 560-8531, Japan

[5]Institute for Catalysis, Hokkaido University, Sapporo, Hokkaido 001-0021, Japan

[6]School of Pharmacy, Jining Medical University, 669 Xueyuan Road, Rizhao, Shandong, 276800, China

E-mail: hirose@scl.kyoto-u.ac.jp (T.H.), kmatsuda@sbchem.kyoto-u.ac.jp (K.M.)

Table of Contents

1. Experimental Details

A. Synthesis of Materials p.2
B. X-ray Crystallography p.15
C. UV-vis., CD, and Fluorescence Spectroscopy p.15
D. Transient Absorption Spectroscopy p.15
E. Theoretical Calculations p.16
F. Cyclic Voltammetry p.17

2. Supplementary Figures and Tables p.18

3. Charts of ^{1}H and ^{13}C NMR Spectra p.84

4. Supplementary References p.107

1. Experimental Details

A. Synthesis of Materials

Unless specifically mentioned, all commercially available reagents and solvents were of standard reagent quality and used without further purification. Spectral grade solvents were used for spectroscopic measurements. All reactions were monitored by thin-layer chromatography carried out on 0.2 mm Merck silica gel plates (60F-254). Column chromatography was performed on silica gel (Nacalai Tesque, 70–230 mesh) or on a Biotage Isolera One instrument with a Rening flash silica gel cartridge. Compound **1** was purified by a preparative gel permeation chromatography (GPC) (Japan Analytical Industry Co., Ltd., JAIGEL-1H and 2H, eluent: chloroform) and by a HPLC (Kanto chemical, Mightysil Si 60 250-4.6, 5μm). ^{1}H and ^{13}C NMR spectra were recorded on a JEOL JNM-ECZ 500R, a JNM-ECS400, or a JNM-ECA600P instrument. Proton chemical shifts recorded in $CDCl_3$ are reported in ppm downfield from tetramethylsilane (TMS, 0 ppm) and carbon chemical shifts listed relative to the residual $^{13}CDCl_3$ (77.00 ppm) as an internal reference. Proton and carbon chemical shifts recorded in $CDCl_2CDCl_2$ listed relative to residual $CDCl_2CHCl_2$ (6.00 ppm) and $^{13}CDCl_2CDCl_2$ (73.79 ppm) as internal reference. Mass spectra were obtained by a Thermo Scientific LTQ orbitrapXL mass spectrometer. Compound **21** was purchased from Tokyo chemical industry (TCI). Characterization of compound **13** has been reported in literature.[1]

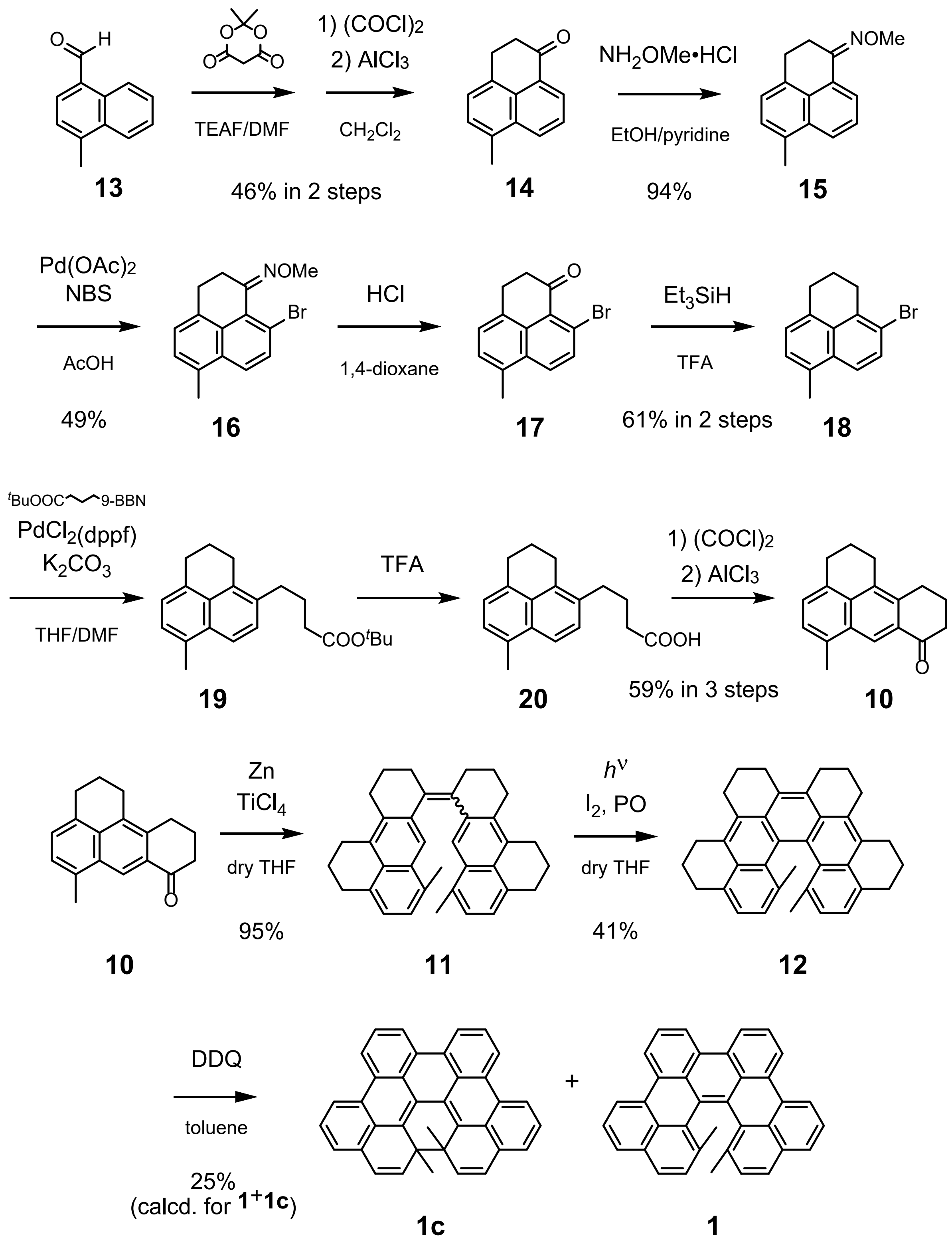

Supplementary Figure 1: Synthesis of π-extended [5]helicene 1.

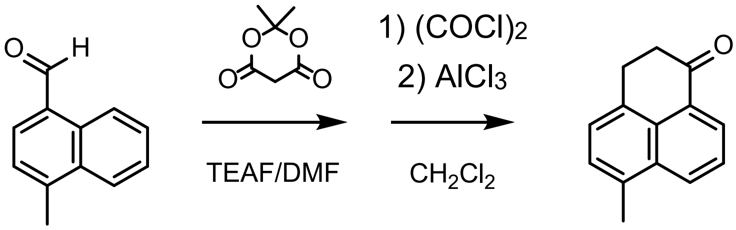

13 46% in 2 steps **14**

To a solution of **13** (3.9 g, 23 mmol) in triethylammonium formate (10 mL) and DMF (12 mL) was added Meldrum's acid (3.2 g, 22 mmol). The mixture was stirred for 9.5 hours at 100 °C. The resulting solution was then cooled to room temperature and poured into aq. HCl (1 M, 25 mL). The solution was cooled under ice bath. The precipitate was filtered with suction, then was dried under reduced pressure to give crude product as a brownish solid. The product was used for the next reaction without further purification.

A solution of crude product and oxalyl chloride (4 mL, ca. 50 mmol) in CH_2Cl_2 (30 mL) was stirred at 30 °C for 2 hours. Excess oxalyl chloride and CH_2Cl_2 were removed under reduced pressure. The resulting oil was dissolved in dry CH_2Cl_2 (100 mL), then was purged with nitrogen and cooled to −78 °C. After the addition of grounded $AlCl_3$ (4.8 g, 36 mmol) to the solution at −78 °C under nitrogen atmosphere, the resulting solution was stirred at the temperature for 3 h, then was gradually warmed to room temperature overnight. Aq. HCl (1 M, 50 mL) was added to the solution. The reaction mixture was extracted with CH_2Cl_2 and washed with water. The organic layer was dried over $MgSO_4$ and concentrated in vacuo. The crude product was purified by silica gel column chromatography (hexane/CH_2Cl_2 = 90/10 to 40/60) to give **14** (2.0 g, 10 mmol, 46% in 2 steps) as a yellow solid.

^{1}H NMR (500 MHz, $CDCl_3$, δ): 2.69 (s, 3H), 2.94 (t, *J* = 7.3 Hz, 2H), 3.37 (t, *J* = 7.0 Hz, 2H), 7.29–7.33 (m, 2H), 7.61 (t, *J* = 7.8 Hz, 1H), 8.19–8.23 (m, 2H); ^{13}C NMR (126 MHz, $CDCl_3$, δ): 19.3, 28.3, 38.5, 124.7, 125.2, 125.3, 126.8, 130.1, 130.4, 131.1, 131.7, 132.6, 132.7, 198.9; HRMS–DI–EI (*m*/*z*): $[M]^+$ calcd for $C_{14}H_{12}O^+$, 196.0883; found, 196.0884.

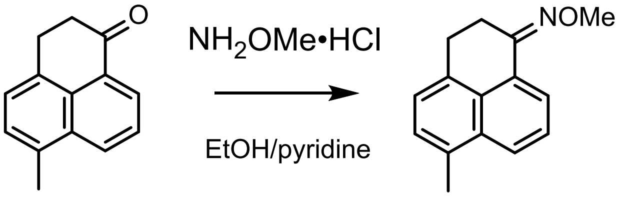

14 94% **15**

A mixture of **14** (1.3 g, 6.6 mmol), $NH_2OMe\cdot HCl$ (1.0 g, 12 mmol), and pyridine (5 mL) in EtOH (50 mL) was stirred at 90 °C for 90 minutes. After cooling the solution to room temperature, aq. HCl (1 M, 20 mL) was added. The reaction mixture was extracted with CH_2Cl_2 (ca. 200 mL). The organic layer was dried over $MgSO_4$, filtered, and the solvent was evaporated to dryness. The residue was purified by silica gel column chromatography (hexane/CH_2Cl_2 = 70/30) to give **15** (1.4 g, 6.2 mmol, 94%) as a yellow solid.

^{1}H NMR (400 MHz, $CDCl_3$, δ): 2.65 (s, 3H), 3.03 (t, *J* = 6.8 Hz, 2H), 3.15 (t, *J* = 7.0 Hz, 2H), 4.05

(s, 3H), 7.17–7.24 (m, 2H), 7.51 (t, *J* = 7.8 Hz, 1H), 8.00 (d, *J* = 8.4 Hz, 1H), 8.10 (d, *J* = 7.2 Hz, 1H); ^{13}C NMR (101 MHz, $CDCl_3$, δ): 19.4, 22.5, 28.3, 62.0, 121.1, 124.1, 125.5, 125.8, 126.2, 129.1, 129.5, 131.8, 132.3, 132.6, 155.0; HRMS–DI–EI (*m*/*z*): $[M]^+$ calcd for $C_{15}H_{15}ON^+$, 225.1148; found, 225.1150.

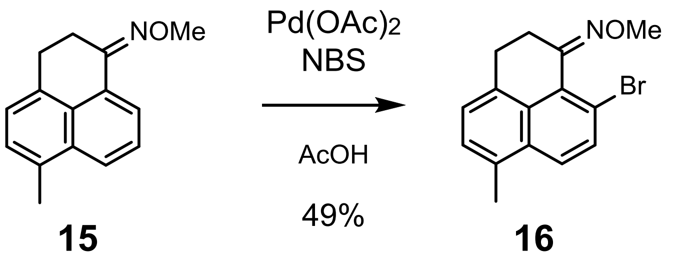

15 **16**

A mixture of **15** (1.3 g, 5.8 mmol), $Pd(OAc)_2$ (0.11 g, 0.48mmol), and NBS (1.1 g, 6.2 mmol) in AcOH (40 mL) was stirred at 90 °C for 1 hour. The solution was cooled to room temperature, and poured into aq. NaOH (3 M, 100 mL). The mixture was extracted with CH_2Cl_2 (ca. 300 mL). The organic layer was washed with brine, dried over $MgSO_4$, and concentrated in vacuo. The residue was purified by silica gel column chromatography (hexane/CH_2Cl_2 = 70/30) to give **16** (0.87 g, 2.8 mmol, 49%) as a yellow solid.

^{1}H NMR (400 MHz, $CDCl_3$, δ) 2.58 (s, 3H), 3.00 (t, *J* = 7.0 Hz, 2H), 3.13 (t, *J* = 6.6 Hz, 2H), 4.08 (s, 3H), 7.14–7.21 (m, 2H), 7.66–7.73 (m, 2H); ^{13}C NMR (101 MHz, $CDCl_3$, δ): 19.3, 23.3, 30.2, 62.1, 117.2, 125.4, 125.9, 126.7, 128.9, 131.5, 131.65, 131.75, 131.9, 132.5, 154.7; HRMS–DI–EI (*m*/*z*): $[M]^+$ calcd for $C_{15}H_{14}ONBr^+$, 303.0253; found, 303.0262.

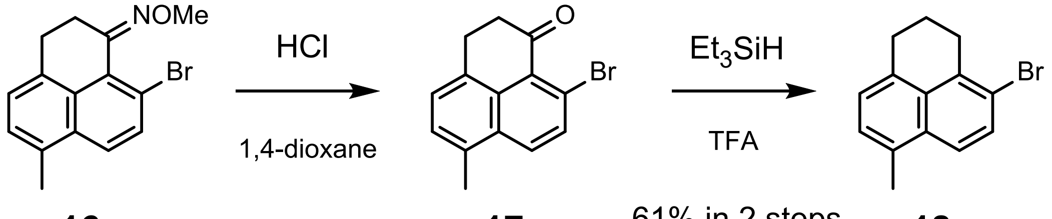

16 **17** 61% in 2 steps **18**

A solution of **16** (7.1 g, 23 mmol) in 1,4-dioxane (120 mL) and aq. HCl (6 M, 180 mL) was stirred at 110 °C for 90 minutes. After cooling the resulting solution to room temperature, the mixture was extracted with CH_2Cl_2 and washed with water. The organic layer was dried over $MgSO_4$, filtered, and concentrated in vacuo. The reside was passed through a plug of silica gel (CH_2Cl_2 as an eluent) to give crude **17**, which was used for the next reaction without further purification.

A solution of crude **17** in TFA (40 mL) and Et_3SiH (10 mL, 63 mmol) was stirred at room temperature for 11 hours. The reaction mixture was extracted with hexane and washed with water. The organic layer was dried over $MgSO_4$, filtered, and concentrated in vacuo. The residue was passed through a plug of silica gel (hexane as eluent), and then further purified by silica gel column chromatography (hexane) to give **18** (3.7 g, 14 mmol, 61%) as a white solid.

^{1}H NMR (500 MHz, $CDCl_3$, δ): 2.05 (quint., *J* = 6.3 Hz, 2H), 2.62 (s, 3H), 3.02 (t, *J* = 6.3 Hz, 2H), 3.13 (t, *J* = 6.3 Hz, 2H), 7.15 (d, *J* = 7.0 Hz, 1H), 7.21 (d, *J* = 7.0 Hz, 1H), 7.60 (d, *J* = 9.0 Hz, 1H), 7.67 (d, *J* = 9.0 Hz, 1H); ^{13}C NMR (126 MHz, $CDCl_3$, δ): 19.5, 22.9, 31.1, 31.8, 120.7, 123.6, 124.5, 126.5, 129.4, 131.5,

131.7, 132.2, 134.1, 135.4; HRMS–DI–EI (*m/z*): [M]$^+$ calcd for $C_{14}H_{13}Br^+$, 260.0195; found, 260.0199.

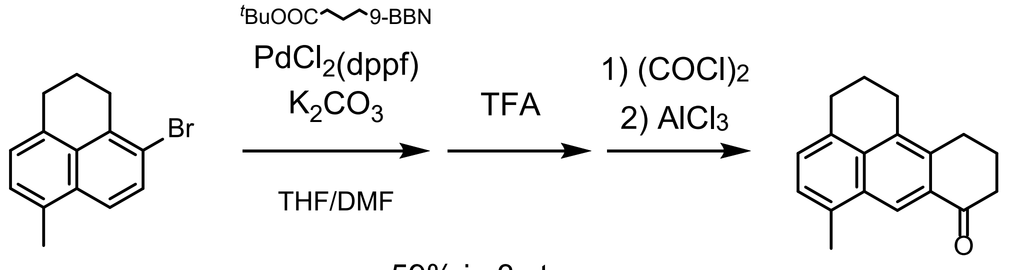

18 **10**

To a solution of 9-borabicyclo[3.3.1]nonane (9-BBN, 0.5 M solution in THF, 54 mL, 27 mmol) was added *tert*-butyl 3-butenoate (3.8 g, 27 mmol) at room temperature. The mixture was stirred for 3 hours. After addition of DMF (80 mL), $PdCl_2$(dppf) (0.50 g, 0.68 mmol), **18** (5.0 g, 19 mmol), and K_2CO_3 (5.5 g, 40 mmol), the reaction mixture was further stirred for 12 hours at 55 °C. The reaction product was extracted with hexane, washed with water two times. The organic layer was dried over $MgSO_4$, and passed through a plug of silica gel (CH_2Cl_2 as eluent). The solution was concentrated in vacuo and the crude mixture was purified by silica gel column chromatography (hexane/CH_2Cl_2 = 90/10 to 80/40) to give crude product (*tert*-butyl ester, **19**) as a pale yellow wax.

The crude **19** was dissolved in trifluoroacetic acid (TFA, 40 mL) and stirred for 90 minutes at room temperature. The solution was evaporated to give the deprotected derivative (carboxylic acid, **20**), which was used for the next reaction without further purification.

A solution of crude **20** in oxalyl chloride (9 mL, ca. 100 mmol) and CH_2Cl_2 (40 mL) was stirred overnight at 30 °C. Excess oxalyl chloride was removed under reduced pressure. The resultant oil was diluted with CH_2Cl_2 (350 mL), then $AlCl_3$ (5.0 g, 38 mmol) was added to the solution at −78 °C. The solution was stirred at the temperature for 2 hours and further stirred at room temperature for 2 hours. After adding aq. HCl (1 M, 100 mL) slowly, the reaction mixture was extracted with CH_2Cl_2 (ca. 500 mL) and washed with water. The organic layer was dried over $MgSO_4$ and passed through a plug of silica gel (CH_2Cl_2 as eluent), and the solution was concentrated in vacuo. The crude mixture was purified by silica gel column chromatography (hexane/CH_2Cl_2 = 60/40 to 50/50) to give **10** (2.8 g, 11 mmol, 59% in 3 steps) as a yellow solid.

^{1}H NMR (500 MHz, $CDCl_3$, δ): 2.06 (quint., *J* = 6.3 Hz, 2H), 2.18 (quint., *J* = 6.3 Hz, 2H), 2.69 (s, 3H), 2.71 (t, *J* = 6.8 Hz, 2H), 2.97–3.02 (m, 6H), 7.16 (s, 2H), 8.69 (s, 1H); ^{13}C NMR (126 MHz, $CDCl_3$, δ): 19.5, 22.6, 22.9, 26.4, 27.8, 30.8, 39.1, 122.9, 125.9, 126.1, 129.7, 130.7, 132.7, 133.7, 133.9, 134.5, 134.6, 199.4; HRMS–DI–EI (*m/z*): [M]$^+$ calcd for $C_{18}H_{18}O^+$, 250.1352; found, 250.1357.

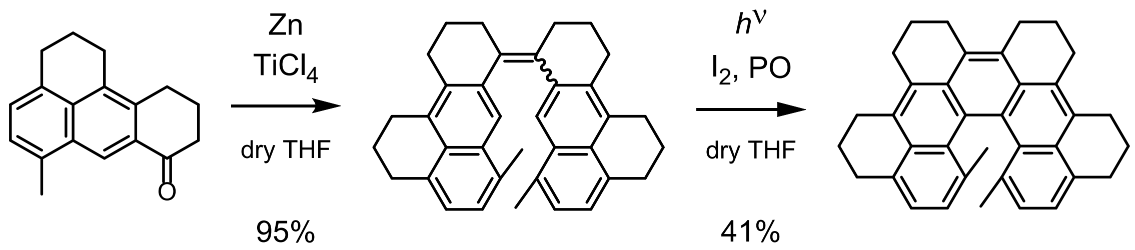

10 **11** **12**

To a suspension of zinc powder (1.0 g, 15 mmol) in dry THF (50 mL) was added $TiCl_4$ (1.3 g, 6.9 mmol). A solution of **10** (1.0 g, 4.0 mmol) in dry THF (30 mL) was then added to the reaction mixture. The solution was refluxed for 2 hours, cooled to room temperature, and then aq. HCl (1 N, ca. 50 mL) was added. The reaction product was extracted with CH_2Cl_2 (ca. 200 mL) and washed with water. The organic layer was dried over $MgSO_4$, filtered, and concentrated in vacuo. The crude product was passed through a plug of silica (CH_2Cl_2) to give **11** (0.89 g, 1.9 mmol, 95%) as a yellow solid, which was used for the next reaction without further purification.

A round-bottom quartz flask was charged with **11** (100 mg, 0.21 mmol), iodine (330 mg, 1.3 mmol), and dry THF (130 mL). The solution was purged with N_2 bubbling for 5 minutes. After the addition of propylene oxide (15 mL, ca. 220 mmol), the solution was further purged with N_2 bubbling for 2 minutes. The solution was stirred and irradiated with a super-high-pressure mercury lamp (500 W) through a sharp-cut filter (UV-29) for 75 minutes. The resulting solution was stirred with $Na_2S_2O_3$ powder, filtered, and then was concentrated in vacuo. The crude product was purified by silica gel column chromatography (hexane/dichloromethane = 90/10), which was further purified by gel permeation chromatography (GPC) to give **12** (41 mg, 0.088 mmol, 41%) as a yellow solid.

^{1}H NMR (600 MHz, $CDCl_3$, δ): 0.76 (s, 6H), 2.02–2.13 (m, 4H), 2.20–2.31 (m, 4H), 3.07–3.31 (m, 16H), 6.82 (d, J = 6.0 Hz, 2H), 7.18 (d, J = 6.0 Hz, 2H); ^{13}C NMR (151 MHz, $CDCl_3$, δ): 22.1, 23.1, 23.4, 27.2, 27.7, 28.6, 31.3, 124.1, 124.4, 126.7, 127.0, 127.8, 128.8, 129.1, 129.2, 131.3, 132.0, 132.5; HRMS–APCI–Orbitrap (*m*/*z*): [M + H]$^+$ calcd for $C_{36}H_{35}^+$, 467.2733; found, 467.2727.

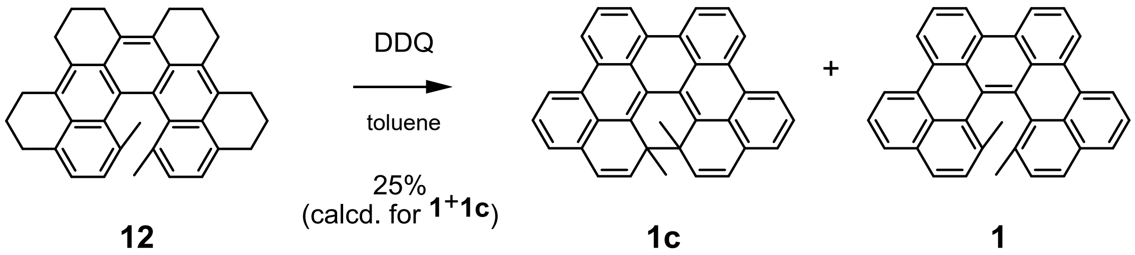

A solution of compound **12** (41 mg, 0.088 mmol) in toluene (60 mL) was degassed by N_2 bubbling at 80 °C for 5 minutes. After the addition of 2,3-dichloro-5,6-dicyano-1,4-benzoquinone (DDQ, 150 mg, 0.66 mmol), the resulting solution was stirred at 80 °C for 2 minutes under nitrogen atmosphere. Then the solution was cooled and immediately quenched by the addition of aq. $Na_2S_2O_3$ (conc. ca. 10 mL). The reaction product was extracted with toluene (ca. 100 mL) and the combined organic layer was concentrated in vacuo. The crude product was purified by silica gel column chromatography (hexane/CH_2Cl_2 = 90/10 to 85/15) and GPC to give a mixture of **1** and **1c** (10 mg, 0.022 mmol, 25%) as a deep red solid. A pure form of **1** was obtained by HPLC (HPLC column, Mightysil Si 60 250 mm-4.6 mmϕ, 5 μm; eluent, hexane/CH_2Cl_2 = 95/5; flow rate, 1.0 mL/min; detection wavelength, 500 nm; R_f = 10.0 and 11.6 min for **1c** and **1**, respectively).

For compound **1**

^{1}H NMR (500 MHz, $CDCl_3$, δ): 1.59 (s, 6H), 6.99 (d, J = 8.5 Hz, 2H), 7.54–7.58 (m, 4H), 7.70 (t, J = 7.7 Hz, 2H), 7.74 (d, J = 8.0 Hz, 2H), 8.28 (d, J = 7.5 Hz, 2H), 8.32 (d, J = 7.5 Hz, 2H), 8.53 (d, J = 7.5 Hz, 2H); ^{13}C NMR (151 MHz, $CDCl_3$, δ): 22.0, 120.8, 121.5, 122.3, 125.7, 126.3, 127.0, 127.2, 129.4, 129.96, 130.04, 130.1, 130.3, 130.5, 130.7, 131.6, 132.9, 133.5. HRMS–APCI–Orbitrap (*m*/*z*): $[M + H]^+$ calcd for $C_{36}H_{23}^+$, 455.1794 found, 455.1783 (measured for a sample in PSS under irradiation with 337 nm light).

For compound **1c**

^{1}H NMR (500 MHz, $CDCl_3$, δ): 1.75 (s, 6H), 6.43 (d, J = 9.5 Hz, 2H), 6.68 (d, J = 10.0 Hz, 2H), 7.17 (d, J = 6.0 Hz, 2H), 7.45 (dd, J = 8.5, 7.0 Hz, 2H), 7.66 (t, J = 8.0 Hz, 2H), 8.38 (d, J = 8.0 Hz, 2H), 8.39 (d, J = 7.0 Hz, 2H), 8.51 (d, J = 8.0 Hz, 2H); ^{13}C NMR (126 MHz, $CDCl_3$, δ): 26.3, 44.2, 121.4, 122.5, 122.6, 124.7, 125.0, 126.4, 126.49, 126.55, 127.2, 127.3, 129.6, 130.1, 130.9, 131.3, 132.1, 133.4. HRMS–APCI–Orbitrap (*m*/*z*): $[M + H]^+$ calcd for $C_{36}H_{23}^+$, 455.1794 found, 455.1783.

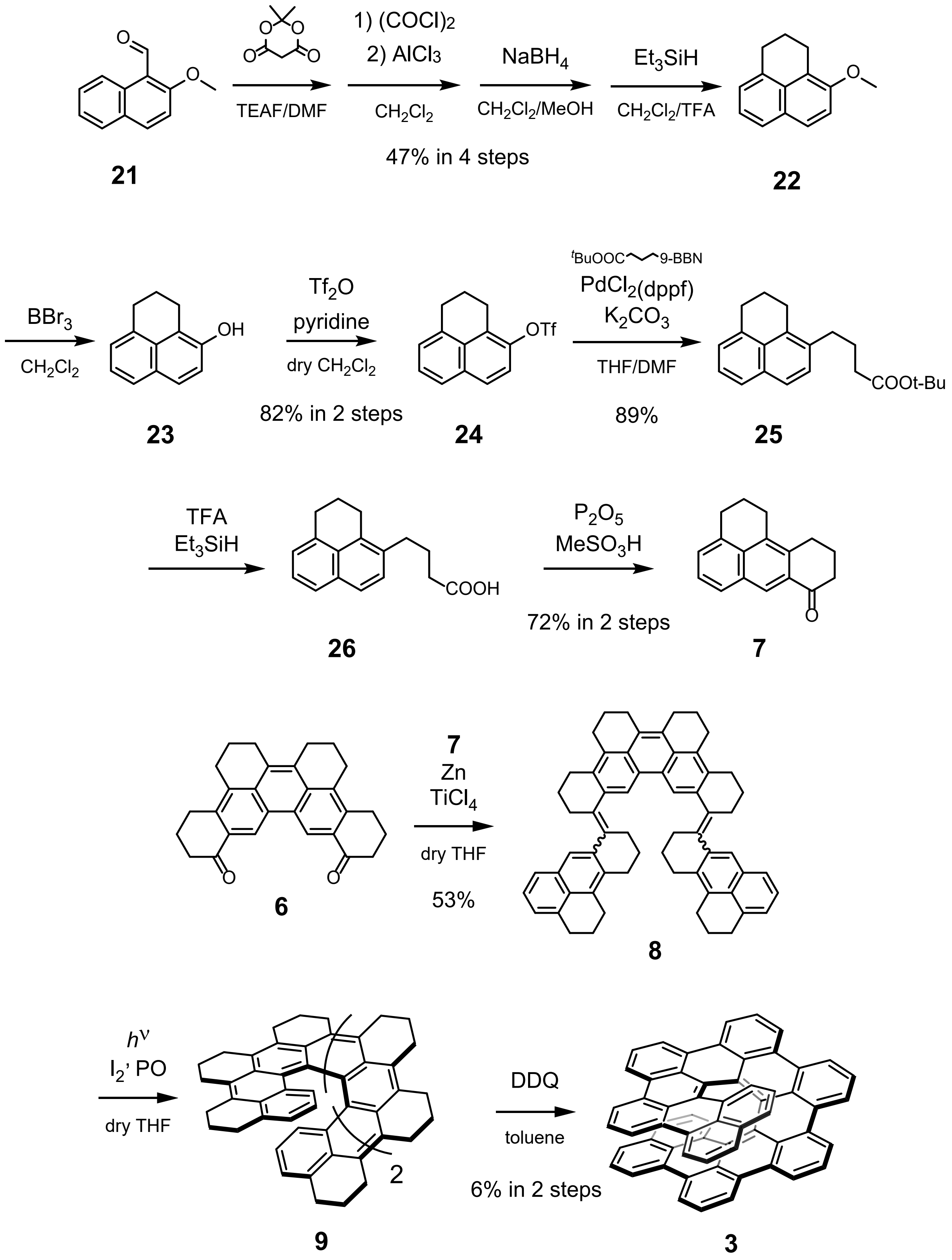

Supplementary Figure 2: Synthesis of π-extended [9]helicene 3

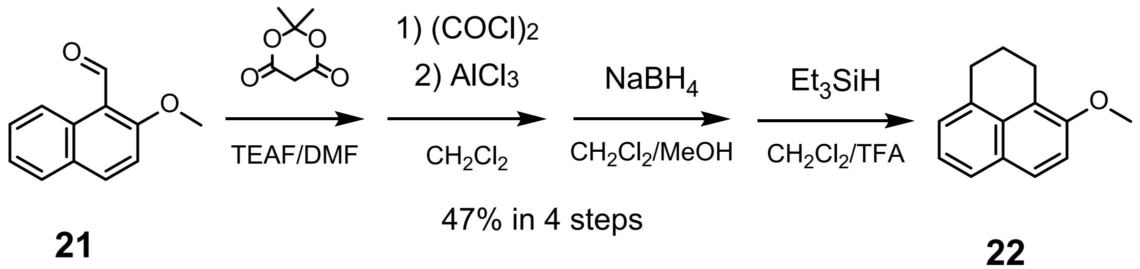

21 **22**

To a solution of **21** (18.0 g, 97 mmol) in triethylammonium formate (40 mL) and DMF (50 mL) was added Meldrum's acid (14.6 g, 101 mmol). The mixture was stirred for 19 hours at 100 °C. The resulting solution was then cooled to room temperature and poured into aq. HCl (4 M, 200 mL). The solution was cooled under ice bath to generate precipitates, which was filtered with suction and was dried under reduced pressure to give crude product (21.2 g) as a brownish solid. The product was used for the next reaction without further purification.

A solution of crude product (21.2 g) in oxalyl chloride (18 mL, ca. 210 mmol) and dry CH_2Cl_2 (50 mL) was stirred at 30 °C for 13 hours. Excess oxalyl chloride and CH_2Cl_2 were removed under reduced pressure. The resultant oil was dissolved in dry CH_2Cl_2 (730 mL), which was purged with nitrogen and cooled to −78 °C. After addition of grounded $AlCl_3$ (16.4 g, 123 mmol) at −78 °C under nitrogen, the resulting solution was stirred at the temperature for 4 hours, then was gradually warmed up to 0 °C over 3 hours. After addition of aq. HCl (1 M, 200 mL), reaction mixture was extracted with CH_2Cl_2 (ca. 500 mL) and washed with brine. The organic layer was dried over $MgSO_4$ and concentrated in vacuo. The crude solid was used for the next reaction without further purification.

To a solution of crude product in MeOH (350 mL) and CH_2Cl_2 (400 mL) was slowly added $NaBH_4$ (8.0 g, 211 mmol) at 0 °C. The solution was stirred at 0 °C for 14 hours. After slowly adding aq. HCl (1 M, 200 mL) to the solution at 0 °C, the reaction mixture was extracted with CH_2Cl_2 and washed with water. The organic layer was dried over $MgSO_4$ and concentrated in vacuo. The resulting oil was used for the next reaction without further purification.

To a solution of crude product in CH_2Cl_2 (500 mL) and TFA (50 mL) was added Et_3SiH (18 mL, 113 mmol) at room temperature. The reaction mixture was stirred at room temperature for 5.5 hours. The solvents were evaporated. The crude product was purified by silica gel column chromatography (hexane/CH_2Cl_2 = 90/10) to give **22** (9.1 g, 46 mmol, 47% in 4 steps) as a colorless oil.

^{1}H NMR (500 MHz, $CDCl_3$, δ): 2.03 (quint., J = 6.1 Hz, 2H), 3.02–3.06 (m, 4H), 3.93 (s, 3H), 7.17 (d, J = 7.0 Hz, 1H), 7.20–7.24 (m, 2H), 7.60 (d, J = 8.0 Hz, 1H), 7.68 (d, J = 9.0 Hz, 1H); ^{13}C NMR (126 MHz, $CDCl_3$, δ): 22.3, 23.8, 30.9, 56.3, 112.9, 121.3, 123.2, 124.0, 125.7, 126.6, 128.9, 130.9, 135.4, 152.6; HRMS–DI–EI (m/z): $[M]^+$ calcd for $C_{14}H_{14}O^+$, 198.1039; found, 198.1041.

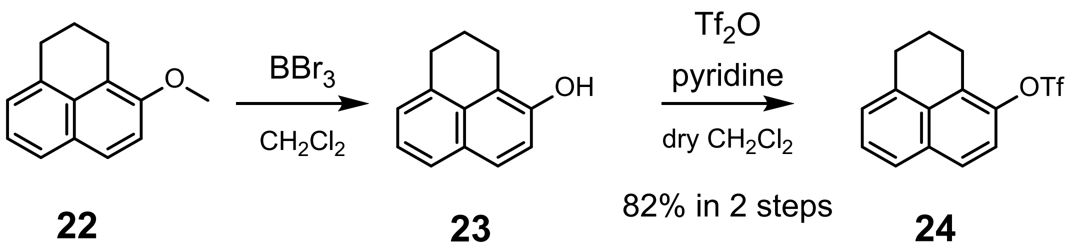

To a solution of **22** (4.0 g, 20 mmol) in CH_2Cl_2 (150 mL) was added BBr_3 (28 mL, 28 mmol, 1.0 M in CH_2Cl_2) at 0 ºC. The mixture was stirred for 5 hours at room temperature, and then saturated aq. $NaHCO_3$ (50 mL) was added to the solution. The reaction mixture was extracted with CH_2Cl_2 (ca. 500 mL), and the organic layer was washed with water and concentrated in vacuo. The resultant solid was purified with silica gel column chromatography (hexane/CH_2Cl_2 = 50/50 to 0/100) to give crude **23** (3.2 g) as a white solid. The solid was used for the next reaction without further purification.

To a solution of crude solid **23** (2.8 g) and dry pyridine (4.5 mL, 56 mmol) in dry CH_2Cl_2 (70 mL) was slowly added triflic anhydride (3.8 mL, 23 mmol) at 0 ºC. The mixture was stirred at room temperature for 13 hours. After the addition of aq. HCl (1 M, 100 mL), the mixture was extracted with CH_2Cl_2 (ca. 300 mL). The organic layer was washed with water, and then was concentrated in vacuo. The crude product was purified by silica gel column chromatography (hexane/CH_2Cl_2 = 50/50) to give **24** (4.6 g, 15 mmol, 82% in 2 steps) as a colorless oil. The reaction yield was calculated by considering the amount of the crude solid of **23** actually used in the second step (2.8 g out of 3.2 g).

^{1}H NMR (400 MHz, $CDCl_3$, δ): 2.06 (quint., *J* = 6.2 Hz, 2H), 3.08 (t, *J* = 6.2 Hz, 2H), 3.15 (t, *J* = 6.2 Hz, 2H), 7.28–7.32 (m, 2H), 7.42 (t, *J* = 7.6 Hz, 1H), 7.67 (d, *J* = 8.0 Hz, 1H), 7.70 (d, *J* = 9.2 Hz, 1H); ^{13}C NMR (101 MHz, $CDCl_3$, δ): 21.9, 24.9, 30.5, 118.7 (quart., *J* = 321.7 Hz, $-CF_3$), 119.0, 125.4, 126.0, 126.6, 127.8, 128.4, 130.5, 132.6, 136.7, 143.5; HRMS–DI–EI (*m*/*z*): $[M]^+$ calcd for $C_{14}H_{11}O_3F_3S^+$, 316.0376; found, 316.0373.

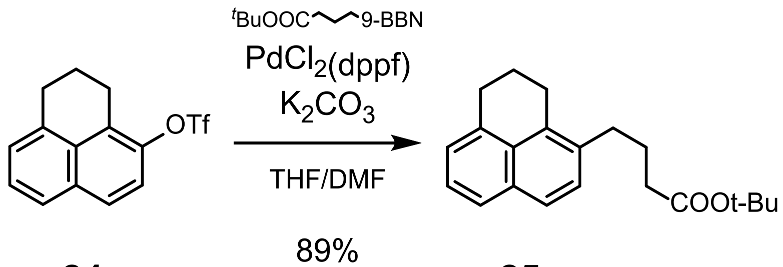

24 **25**

To a dry 100 mL round bottom flask, nitrogen gas was flushed and *tert*-butyl 3-butenoate (3.1 mL, 2.7 g, 19 mmol) and a solution of 9-borabicyclo[3.3.1]nonane (9-BBN, 0.5 M solution in THF, 40 mL, 20 mmol) were added in this order at room temperature. The mixture was stirred for 5.5 hours. After the addition of dry DMF (40 mL), $PdCl_2$(dppf) (0.15 g, 0.21 mmol), **24** (4.6 g, 15 mmol), and K_2CO_3 (5.4 g, 39 mmol), the reaction mixture was stirred for 19 hours at 55°C, and then poured into water. The reaction mixture was extracted with hexane (ca. 200 mL) and washed with water. The organic layer was dried over $MgSO_4$, filtered, and concentrated in vacuo. The residue was purified by silica gel column chromatography (hexane/CH_2Cl_2 = 70/30) to give **25** (4.0 g, 13 mmol, 89%) as a colorless oil.

^{1}H NMR (500 MHz, $CDCl_3$, δ): 1.45 (s, 9H), 1.90 (quint., *J* = 7.5 Hz, 2H), 2.06 (quint., *J* = 6.1 Hz, 2H), 2.28 (t, *J* = 7.5 Hz, 2H), 2.78 (t, *J* = 7.8 Hz, 2H), 3.02–3.08 (m, 4H), 7.18 (d, *J* = 6.5 Hz, 1H), 7.25 (d, *J* = 8.5 Hz, 1H), 7.29 (d, *J* = 7.5 Hz, 1H), 7.59–7.62 (m, 2H); ^{13}C NMR (126 MHz, $CDCl_3$, δ): 22.9, 25.8, 27.3, 28.1, 31.2, 32.5, 35.2, 80.1, 123.9, 124.59, 125.65, 125.8, 127.9, 130.3, 132.4, 132.8, 134.8, 136.1, 172.8; HRMS–DI–EI (*m*/*z*): $[M]^+$ calcd for $C_{21}H_{26}O_2^+$, 310.1927; found, 310.1928.

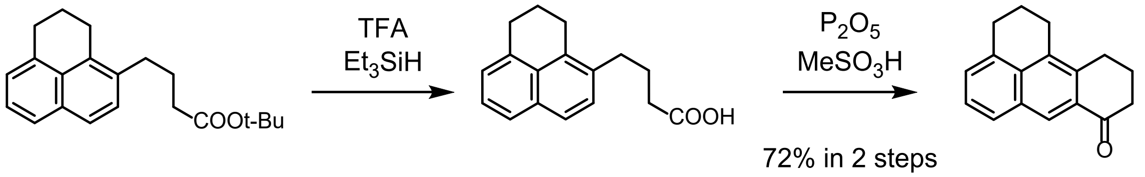

25 **26** **7**

To a solution of trifluoroacetic acid (TFA, 30 mL) and triethylsilane (6.0 mL, 38 mmol) was added **25** (2.0 g, 6.4 mmol) at room temperature. The solution was stirred for 90 minutes at room temperature and then was poured into water (60 mL). The resulting precipitate was filtered with suction. The obtained solid was dried under reduced pressure to give crude **26** as a pale yellow solid.

To the stirred solution of P_2O_5 (1.9 g, 13 mmol) in methansulfonic acid ($MeSO_3H$, 20 mL) was added the crude **26** at 60 °C. The reaction mixture was stirred at the temperature for 90 minutes and then was poured into ice water (ca. 150 mL). The resulting precipitate was dissolved in CH_2Cl_2 and washed with water. The organic layer was dried over $MgSO_4$, filtered through a plug of silica gel (CH_2Cl_2/ethyl acetate =80/20), and concentrated in vacuo. The residue was purified by silica gel column chromatography (hexane/CH_2Cl_2 = 50/50 to 0/100) to give **7** (1.1 g, 4.7 mmol, 72%) as a pale yellow solid.

^{1}H NMR (600 MHz, $CDCl_3$, δ): 2.10 (quint., *J* = 6.2 Hz, 2H), 2.19 (quint., *J* = 6.5 Hz, 2H), 2.72 (t, *J* = 6.6 Hz, 2H), 3.00–3.03 (m, 4H), 3.06 (t, *J* = 6.0 Hz, 2H), 7.29 (d, *J* = 7.2 Hz, 1H), 7.35 (t, *J* = 7.5 Hz, 1H),

7.77 (d, J = 8.4 Hz, 1H), 8.51 (s, 1H); ^{13}C NMR (151 MHz, $CDCl_3$, δ): 22.6, 22.8, 26.5, 27.6, 30.7, 39.2, 125.3, 126.4, 126.6, 128.1, 130.2, 131.4, 132.5, 133.3, 134.7, 136.0, 199.3; HRMS–DI–EI (*m*/*z*): $[M]^+$ calcd for $C_{17}H_{16}O^+$, 236.1196; found, 236.1200.

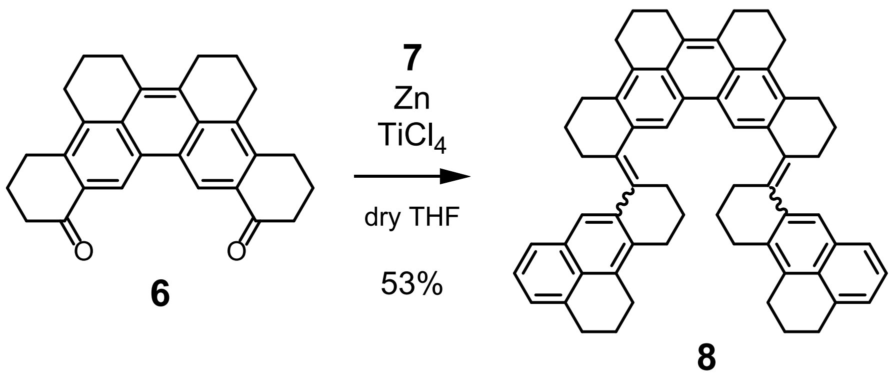

To a suspension of zinc powder (4.5×10^2 mg, 6.9 mmol) in dry THF (8.5 mL) was added $TiCl_4$ (7.9×10^2 mg, 4.2 mmol). A solution of **6** (78 mg, 0.20 mmol) and **7** (4.5×10^2 mg, 1.9 mmol, 9.6 eq.) in dry THF (25 mL) were then added to the reaction mixture. The resulting solution was refluxed for 2 hours and then was cooled to room temperature. The reaction was quenched by adding aq. HCl (1 M, ca. 15 mL), and the resulting solution was extracted with CH_2Cl_2 (ca. 150 mL) and washed with water. The organic layer was dried over $MgSO_4$, filtrated, and concentrated in vacuo. The residue was purified by silica gel column chromatography (hexane/CH_2Cl_2 = 85/15 to 70/30) to remove the dimer of **7**, affording crude **8** (84 mg, 0.10 mmol, 53%) as a yellow solid. ^{1}H NMR spectrum of the product was very complicated probably due to the mixture of *trans*/*cis* isomers, which hampered further assignment (Supplementary Figure 53). Note that the production of **8** was confirmed by LR-MALDI-TOF mass spectrometry. LRMS–MALDI–TOF (*m*/*z*): $[M]^+$ calcd for $C_{62}H_{58}^+$, 802; found, 802.

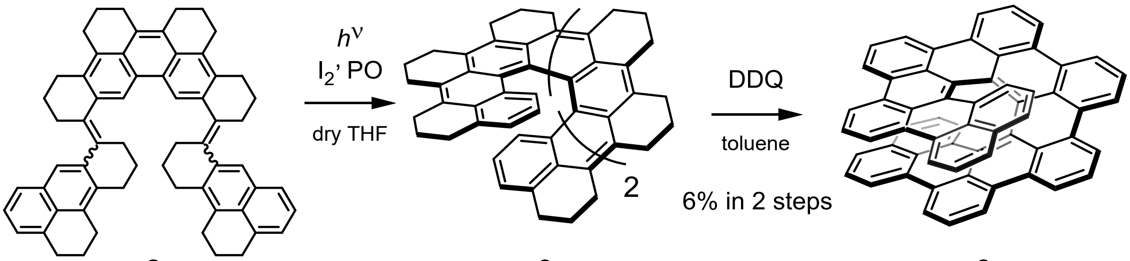

8 **9** **3**

A round-bottom quartz flask was charged with **8** (68 mg, 0.085 mmol), iodine (107 mg, 0.42 mmol), and dry THF (90 mL). The solution was purged with N_2 bubbling for 5 minutes. After the addition of propylene oxide (10 mL, ca. 150 mmol), the solution was further purged with N_2 bubbling for 2 minutes. The solution was stirred and irradiated with a super-high-pressure mercury lamp (500 W) through a sharp-cut filter UV-29 for 75 minutes. The mixture was stirred with $Na_2S_2O_3$ powder, filtered, and then was concentrated in vacuo. The crude product was passed through a plug of silica gel (hexane/dichloromethane = 80/20) to give crude **9** (13 mg) as a yellow solid.

A solution of crude **9** (13 mg) in toluene (13 mL) was degassed by N_2 bubbling at 90 °C for 5 m. After the addition of 2,3-dichloro-5,6-dicyano-1,4-benzoquinone (DDQ, 44 mg, 0.19 mmol), the resulting solution was stirred at 90 °C for 5 minutes. Then the solution was cooled and immediately quenched by the addition of aq. $Na_2S_2O_3$ (conc., ca. 10 mL). The reaction product was extracted with CH_2Cl_2 (ca. 100 mL) and washed with water. The combined organic layer was dried over $MgSO_4$ and was passed through a plug of silica gel (CH_2Cl_2 as an eluent). The solution was concentrated in vacuo. The crude product was purified by silica gel column chromatography (hexane/CH_2Cl_2 = 60/40 to 0/100) and recrystallization (CH_2Cl_2/MeOH) to give **3** (3.7 mg, 0.0048 mmol, 6%) as a deep red solid.

^{1}H NMR (600 MHz, $C_2D_2Cl_4$ with a small excess amount of $N_2H_4{\cdot}H_2O$, δ) 6.36 (t, *J* = 7.5 Hz, 2H), 6.83 (d, *J* = 7.8 Hz, 2H), 7.18 (t, *J* = 7.8 Hz, 2H), 7.23–7.30 (m, 6H), 7.40 (t, *J* = 7.8 Hz, 2H), 7.60–7.65 (m, 4H), 7.75 (d, *J* = 7.8 Hz, 2H), 7.82 (d, *J* = 7.8 Hz, 2H), 8.01 (d, *J* = 7.2 Hz, 2H), 8.13 (d, *J* = 7.8 Hz, 2H), 8.42 (d, *J* = 7.8 Hz, 2H), 8.52 (d, *J* = 7.2 Hz, 2H). ^{13}C NMR (151 MHz, $C_2D_2Cl_4$ with a small excess amount of $N_2H_4{\cdot}H_2O$, δ): 120.9, 121.1, 121.2, 121.6 (2 peaks overlapped), 121.9, 122.2, 123.4, 125.2, 125.5, 125.6, 126.1 (2 peaks overlapped), 126.4, 126.5, 126.9, 127.8, 128.0, 128.2, 128.7, 129.1, 129.5, 129.6, 129.67, 129.73, 130.1, 130.2, 130.6, 130.8, 131.3, 133.5; HRMS–APCI–Orbitrap (*m/z*): $[M + H]^+$ calcd for $C_{62}H_{31}^+$, 775.2420 found, 775.2425.

B. X-ray Crystallography

Single crystals of compound **1**, **1c**, and **3** were prepared by a slow vapor diffusion of methanol into a concentrated solution of **1** or **1c** in chloroform and a slow evaporation of toluene solution of **3** at room temperature. X-ray crystallographic analyses for **1c** and **3** was performed on a Rigaku Saturn724+ CCD diffractometer with a graphite-monochromated Mo Kα radiation (λ = 0.71075 Å). The data collection and cell refinement were performed using CrystalClear-SM Expert 2.1 b46 software (Rigaku, 2016). X-ray crystallographic analysis for **1** was performed on a Bruker Single Crystal CCD X-ray Diffractometer (SMART APEX II) with Mo Kα radiation (λ = 0.71073 Å). The data collection and cell refinement were performed using APEX3 software (v2016.9-0, Bruker AXS, 2016). The structure of **1** observed based on the diffraction data was refined as a two-component twin using Olex2 software.

The all structures were solved by direct methods (SHELXT) and refined by a full-matrix least-squares techniques against F^2 (SHELXL). The all non-hydrogen atoms were refined anisotropically. Hydrogen atoms were placed using AFIX instructions.

C. UV-vis., CD Spectroscopy

Absorption spectra were measured on a JASCO V-670 spectrophotometer (conditions: scan rate, 200 nm/min; band width, 1 nm) equipped with a ETCS-761 Peltier-type temperature controller. CD spectra were measured on a JASCO J-720WI (conditions: scan rate, 100 nm/min; response, 1 sec; slit width, 1 nm). For the spectroscopic measurements, a quartz cuvette with 10 mm optical path was used.

D. Transient Absorption Spectroscopy

Transient absorption (TA) measurements were conducted with a home-built setup based on a Ti:Sapphire regenerative amplifier (Spitfire, Spectra-Physics, 802 nm, 100 fs, 1 mJ, 1 kHz) seeded by a Ti:Sapphire oscillator (Tsunami, Spectra-Physics, 802 nm, 100 fs, 0.65 W, 80 MHz). The output was split into two portions, and introduced to two optical parametric amplifiers (OPAs, TOPAS-Prime, Light-Conversion) for generation of excitation and probe beams. The first OPA provides near-infrared pulses centered at 1180 nm, which are further converted into white light continuum probe pulses by focusing into a 2-mm CaF_2 plate. This probe pulse was divided into signal and reference pulses. The signal one was guided to the sample and the reference one was used for correction of the intensity fluctuation of the probe pulse. Both the pulses were detected with a pair of multichannel photodiode arrays (PMA-10, Hamamatsu) equipped with polychromators for calculation of transient absorbance. The wavelength of the other OPA was set to 530 or 680 nm, and the output was respectively used for photoexcitation of **1** and **2**. The remaining fundamental pulses at 802 nm after the wavelength conversion in the OPA were used for the excitation of **3**. The delay time between the excitation and probe pulses was controlled with an optical delay stage. The polarization of the excitation pulse was set to the magic angle with respect to that of probe pulse with a Berek variable waveplate. The group velocity dispersion of the white light continuum was corrected on the basis of an optical Kerr effect of carbon tetrachloride. The temporal resolution of the measurements was typically 150 fs, which slightly depends on the observation wavelength. The sample solutions were set in a rotation cell with an optical length of 2 mm. During the measurement, the sample cell was kept rotating to provide a fresh portion of the sample solution

for individual excitation and probe pulses. Steady-state absorption spectra were measured before and after the measurements, and no notable absorbance change was confirmed.

Special care was taken for the acquisition and analysis of TA data of **1** because it is intrinsically mixture of the open- and closed-ring isomers in the photostationary state and the photoirradiation at 530 nm pumps both the isomers into each excited state. In order to obtain TA spectra of the open-ring isomer, which is not contaminated by that of the closed-ring one, we measured and analyzed TA data in a following manner. First, the concentration of the open-ring isomer in solution was maximized by UV irradiation around 340 nm (Xe lamp output filtered by a HOYA U340 filter). The relative portion of the open-ring isomer is 86% and TA spectra were recorded under this condition (ΔA_{open}). Second, the concentration of the closed-ring isomer was maximized by visible irradiation at 400 nm (Xe lamp output filtered by a bandpass filter centered at 400 nm with 10-nm fwhm). The portion of the closed-ring isomer is 94% and TA spectra were measured (ΔA_{closed}) as well as the case of the open-ring isomer. Both the obtained spectra contain transient signals of the counterpart with small amplitude. Thus, to extract TA spectra of pure open-ring isomer, we subtracted scaled ΔA_{closed} from ΔA_{open} so that the spectral feature due to the closed-ring isomer completely vanished. In this subtraction, the optimized scaling factor was 0.12 and fixed at all the delay times. This scaling factor is in line with the relative portion of the remaining closed-ring isomer although it is also affected by the molar absorption coefficients and excitation powers.

E. Theoretical Calculations

The geometrical optimization was carried out at the B3LYP/6-311G(2d,p) level of theory on Gaussian 16 package[2] (for the results in Figures 3 and 4, as well as Table 1 in the main text). For the calculations using Gaussian 16 package, convergence at a local minimum structure was confirmed by no imaginary frequencies on frequency analysis. Successively the optimized local minimum structures were subjected to time-dependent density functional theory (TD-DFT) calculations to obtain excited states at B3LYP/6-311G(2d,p) level. NICS(0) and NICS(1) values[4,5] were calculated at the GIAO-B3LYP/6-311G(2d,p) level of theory. For NICS(1) calculations, the ghost atoms (Bq) were located at the distance of 1 Å from the least-squares plane defined by the six carbon atoms composing a hexagonal ring structure. The anisotropy of current-induced density (ACID) was calculated at the CSGT-B3LYP/6-311G(2d,p) level of theory using the Gaussian 09 package (specified by the keywords of "nmr=CSGT" and "IOp(10/93=1)") and the ACID 2.0.0 program provided by Prof. Rainer Herges (Institut für Organische Chemie, Universität Kiel).[6,7] The geometrical optimization and electronic energies to investigate the minimum energy conical intersection (MECI) structures were calculated at the SF-ωB97xd/6-31G(d) level of theory on Q-Chem 5.3 package[3] (for the results in Figure 5b and 5c in the main text, as well as Supplementary Figure 30–32 and Supplementary Table 7). For the carbohelicenes, i.e., [5], [6], and [7]helicene (Supplementary Table 7), the geometrical optimization in the ground (S_0) state were conducted at the RBHHLYP/6-31G(d) level of theory, and the resulting structures were used for calculating the excitation energy to the Franck-Condon (FC) state at the SF-ωB97xd/6-31G(d) level of theory.

F. Cyclic Voltammetry

Redox potentials were measured by the cyclic voltammetry method on an ALS612E electrochemical analyzer model. Dry THF was used for electrochemical analysis. Electrochemical potentials are determined by using ferrocene/ferrocenium ion couple as an external standard.

2. Supplementary Figures and Tables

2.1. Orbital Coefficients of Phenanthrene and Electronic Coupling Interactions between Repeating Units.

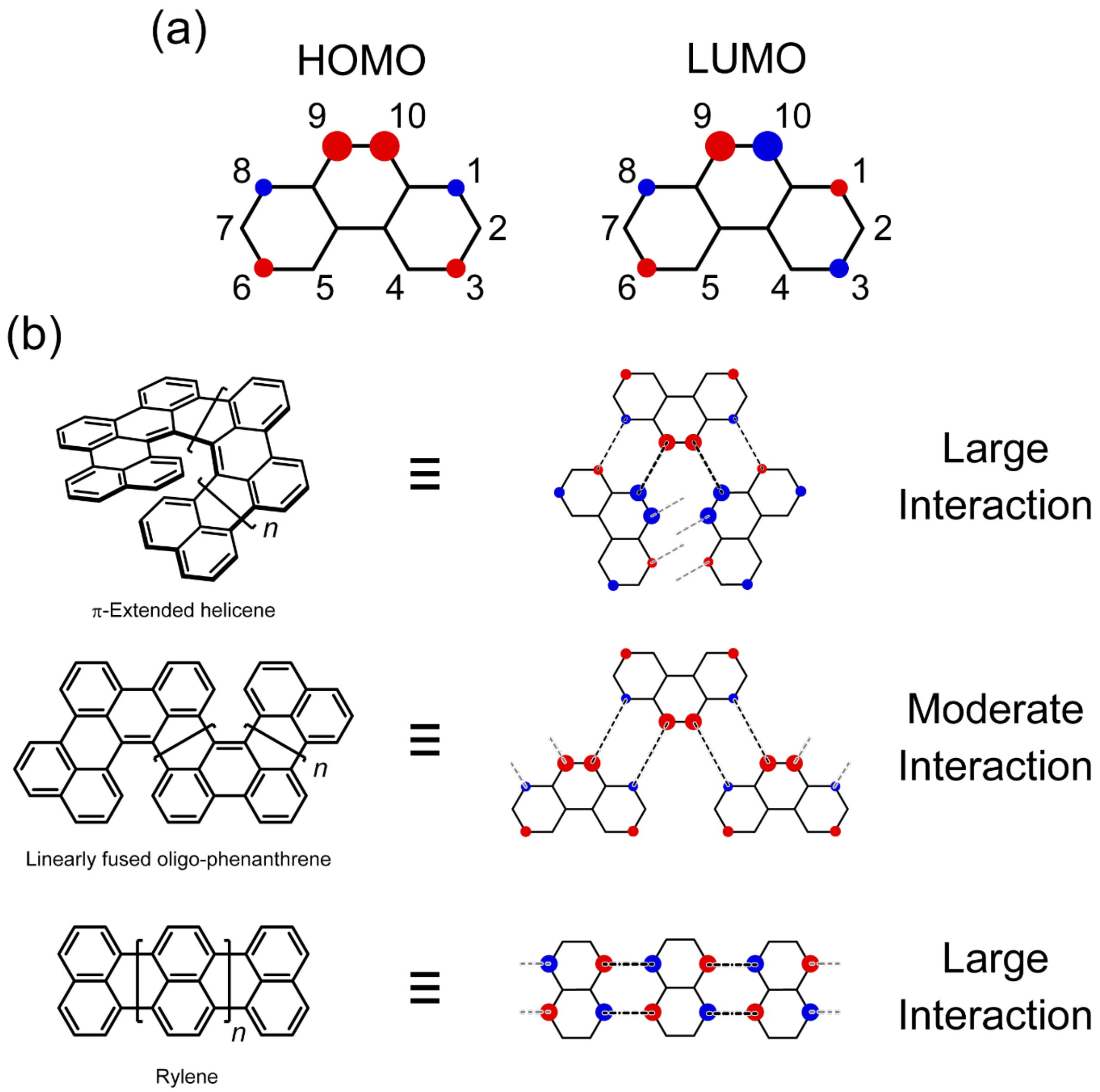

Supplementary Figure 3: Orbital interactions in the frontier orbitals. a Frontier orbitals of phenanthrene. **b** Molecular structures and the out-of-plane (anti-bonding) orbital interactions in the HOMOs for the helical phenanthrene-oligomers (top), the linearly fused oligo-phenanthrene (middle), and rylenes (bottom).

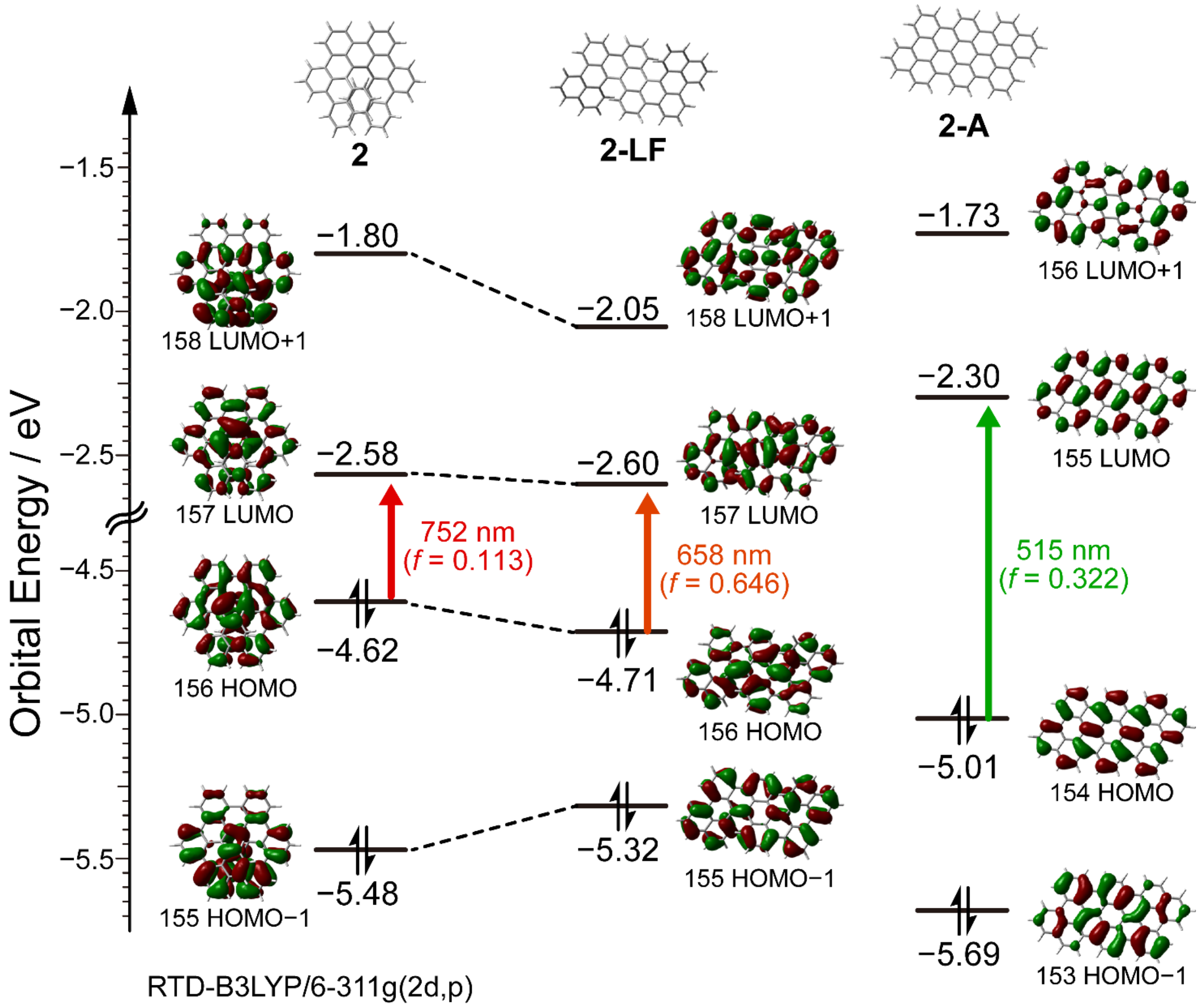

Supplementary Figure 4: Orbital correlation diagrams. π-extended [7]helicene (**2**, $C_{48}H_{24}$), linearly fused oligo-phenanthrene derivative (**2-LF**, $C_{48}H_{24}$), and the fully annulated derivative (**2-A**, $C_{48}H_{20}$) calculated at the RTD-B3LYP/6-311g(2d,p) level of theory. The HOMO–LUMO energy gap of the 1-to-1′ and 10-to-10′ doubly linked oligo-phenanthrene derivative **2** (ΔE_{DFT} = 2.04 eV) is smaller than that of the 1-to-10′ and 10-to-1′ doubly linked derivative **2-LF** (ΔE_{DFT} = 2.11 eV), which can be explained by the difference in electronic coupling between the phenanthrene subunits (see Supplementary Figure 3).

2.2. Photoisomerization Between 1 and 1c in Toluene

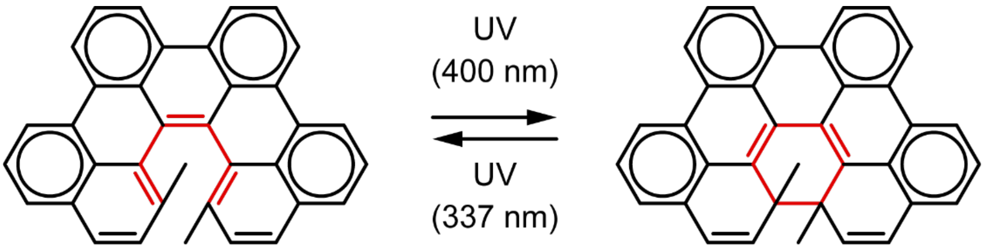

1 **1c**

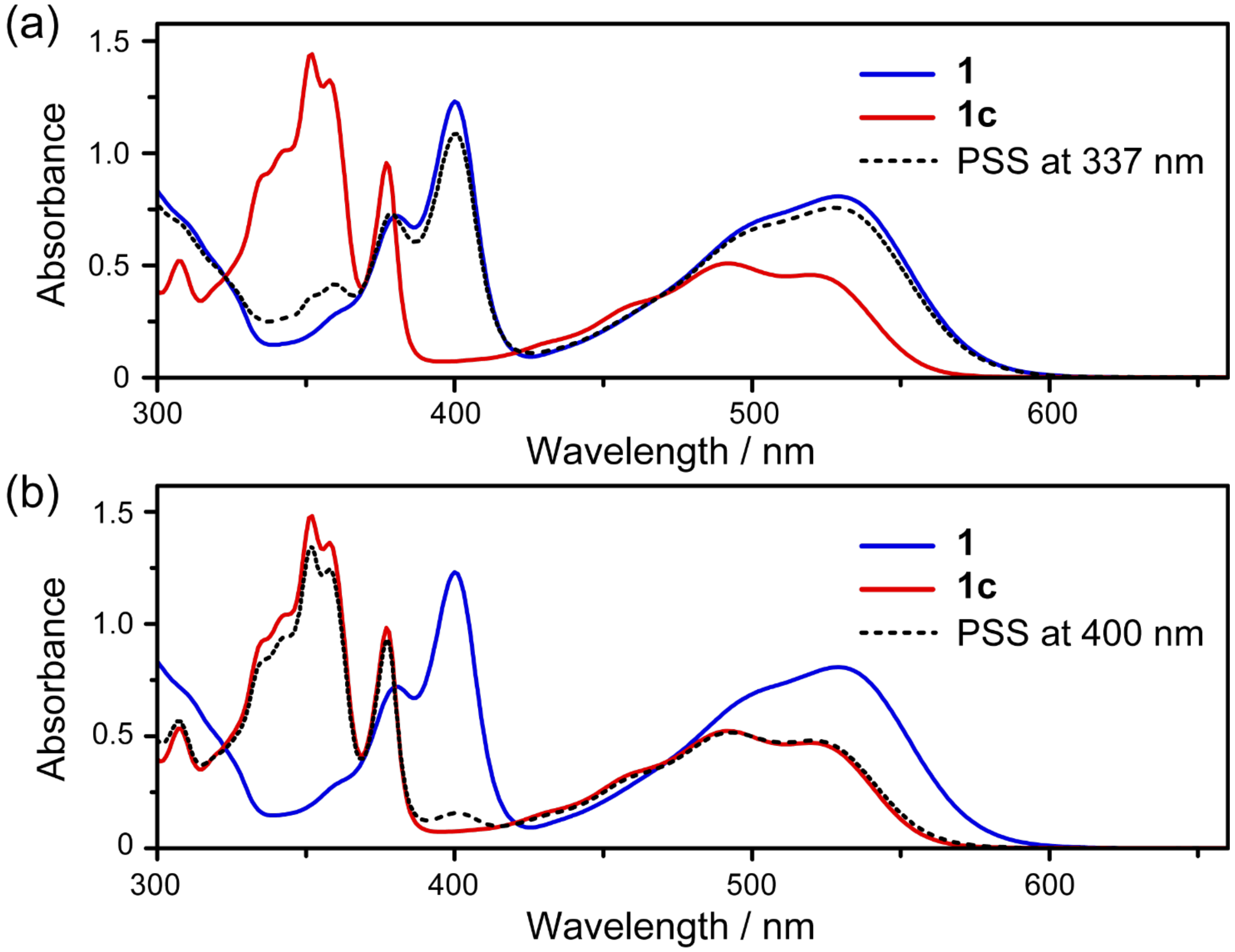

Supplementary Figure 5: Photoisomerization of π-extended [5]helicene 1. UV-vis absorption spectra of the open-ring isomer **1** (solid black line), its closed-ring isomer **1c** (solid red line). **a** The sample in photostationary state (PSS) under irradiation with 337 nm light (black dashed line). **b** The sample in PSS under irradiation with 400 nm light (black dashed line) recorded in toluene at 25 °C.

2.3. Single-Crystal X-ray Diffraction Analyses of 1 and 3

The crystal of **1** was racemate (space group *P*-1) and two crystallographically independent molecules were found in the asymmetric unit, one of which was severely disordered. The values of the non-disordered molecule are reported in discussion in the main text. The crystal of **3** was also racemate (space group *P*-1) consisting of two crystallographically independent molecules in the asymmetric unit. Since there was no distinguishable structural difference between the two structures of **3**, the mean values of the two independent molecules are reported in the discussion.

Supplementary Table 1: Crystallographic parameters for compound 1.

CCDC number	2118093
chemical formula	$2(C_{36}H_{22})$
formula weight	909.151
T / K	100(2)
wavelength / Å	0.71075 (Mo Kα)
color	Red
crystal size, mm	0.15 × 0.057 × 0.051
crystal system	Triclinic
space group	*P*-1 (#2)
a / Å	7.470(3)
b / Å	10.664(4)
c / Å	27.916(12)
α / °	88.744(4)
β / °	87.446(4)
γ / °	89.443(4)
V / Å^3	2220.8(16)
Z	2
Density (calculated)	1.360 g/cm^3
Data/restraints/parameters	7801/528/981
Goodness-of-fit on F^2	1.092
R_1 [$I > 2\sigma(I)$]	0.0659
wR_2 (for all data)	0.1926
Largest peak and hole / eÅ^{-3}	0.4071 and −0.4452

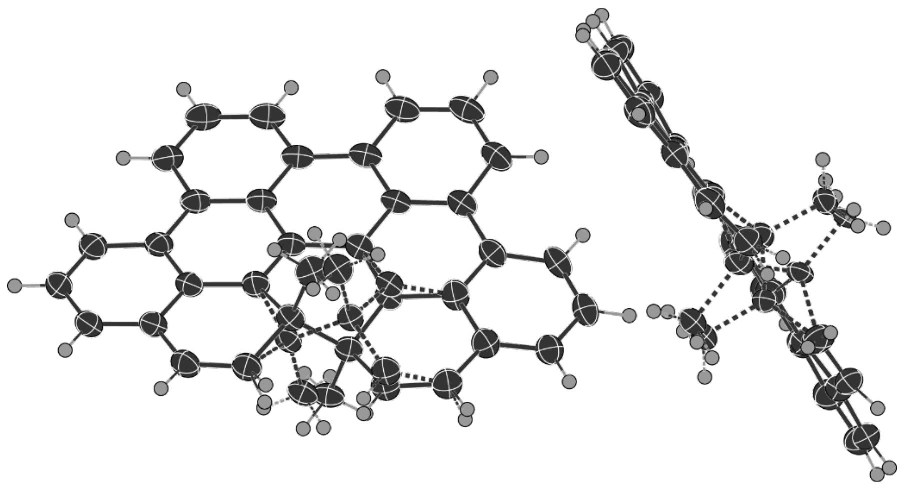

Supplementary Figure 6: X-ray crystal structure. ORTEP drawing of **1c** (closed-ring isomer of **1**) recorded at 143 K, showing 50% probability thermal ellipsoids.

Supplementary Table 2: Crystallographic parameters for compound 1c.

CCDC number	2118109
chemical formula	$2(C_{36}H_{22})$
formula weight	909.07
T / K	143(2)
wavelength / Å	0.71075 (Mo Kα)
color	Red
crystal size, mm	0.35 × 0.35 × 0.35
crystal system	Monoclinic
space group	$P2_1/n$ (#14)
a / Å	9.238(2)
b / Å	26.480(6)
c / Å	18.049(4)
α / °	90
β / °	94.606(4)
γ / °	90
V / Å^3	4400.9(17)
Z	4
Density (calculated)	1.372 g/cm^3
Data/restraints/parameters	9962/0/723
Goodness-of-fit on F^2	1.096
R_1 [$I > 2\sigma(I)$]	0.0583
wR_2 (for all data)	0.1525
Largest peak and hole / eÅ^{-3}	0.234 and −0.211

Supplementary Table 3: Crystallographic parameters for compound 3.

CCDC number	2117834
chemical formula	$2(C_{62}H_{30})$
formula weight	1549.72
T / K	143(2)
wavelength / Å	0.71075 (Mo Kα)
color	Red
crystal size, mm	0.60 × 0.09 × 0.07
crystal system	triclinic
space group	*P*-1 (#2)
a / Å	12.530(3)
b / Å	14.553(3)
c / Å	21.141(5)
α / °	79.609(9)
β / °	86.774(10)
γ / °	70.198(6)
V / $Å^3$	3567.5(13)
Z	2
Density (calculated)	1.443 g/cm^3
Data/restraints/parameters	15538/0/1117
Goodness-of-fit on F^2	0.933
R_1 [$I > 2\sigma(I)$]	0.0536
wR_2 (for all data)	0.1253
Largest peak and hole / e$Å^{-3}$	0.332 and −0.262

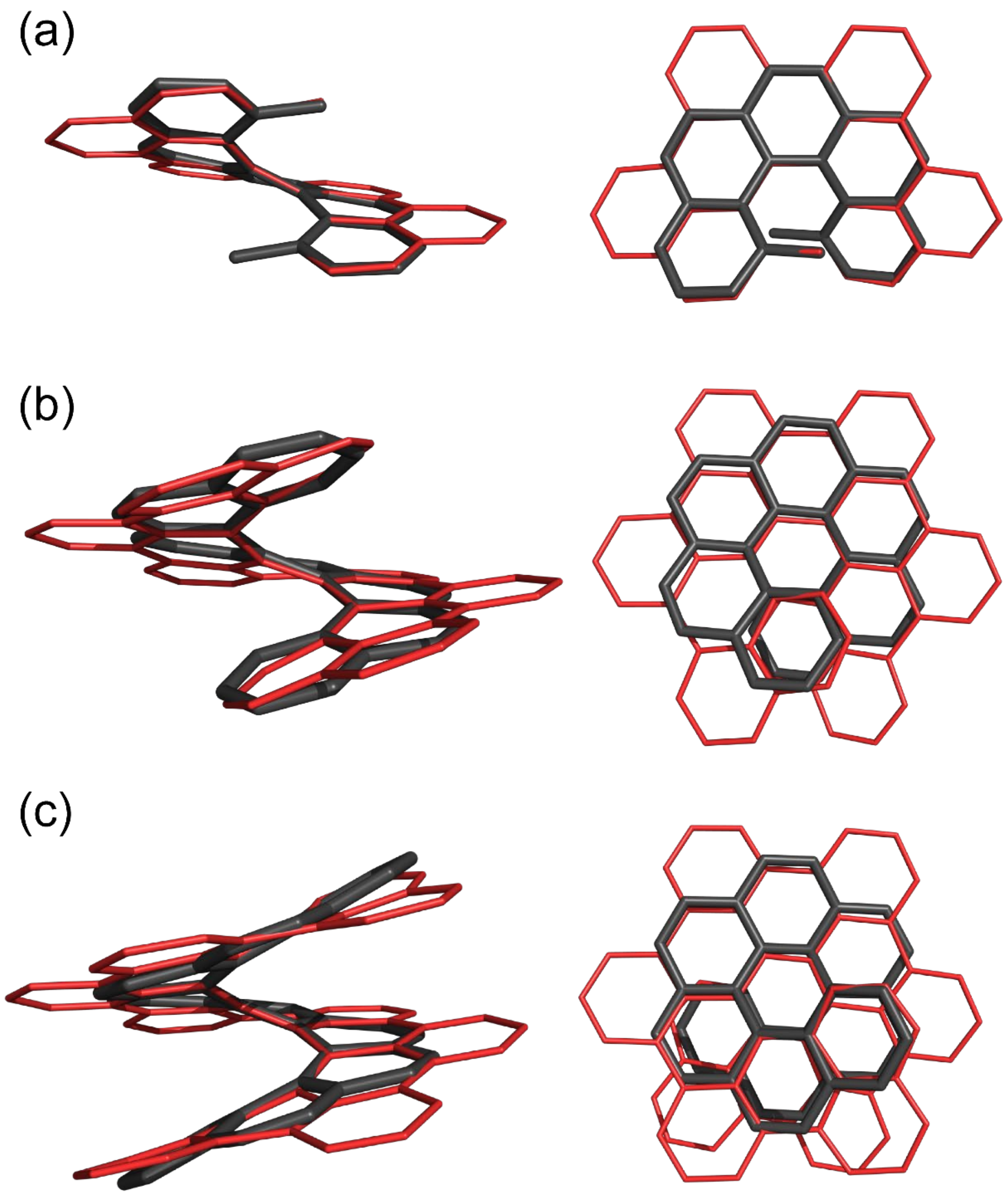

Supplementary Figure 7: Comparison of X-ray crystal structures before and after π-extension. Superimposed X-ray crystal structure of (a) **1** (red thin bond, CCDC Number: 2118093) and 4,11-dibromo-1,14-dimethyl[5]helicene (gray thick bond, CCDC Number: 1528976), (b) **2** (red thin bond, CCDC Number: 1585563) and [7]helicene (gray thick bond, CCDC Number: 1178373), (a) **3** (red thin bond, CCDC Number: 2117834) and [9]helicene (gray thick bond, CCDC Number: 1051158).

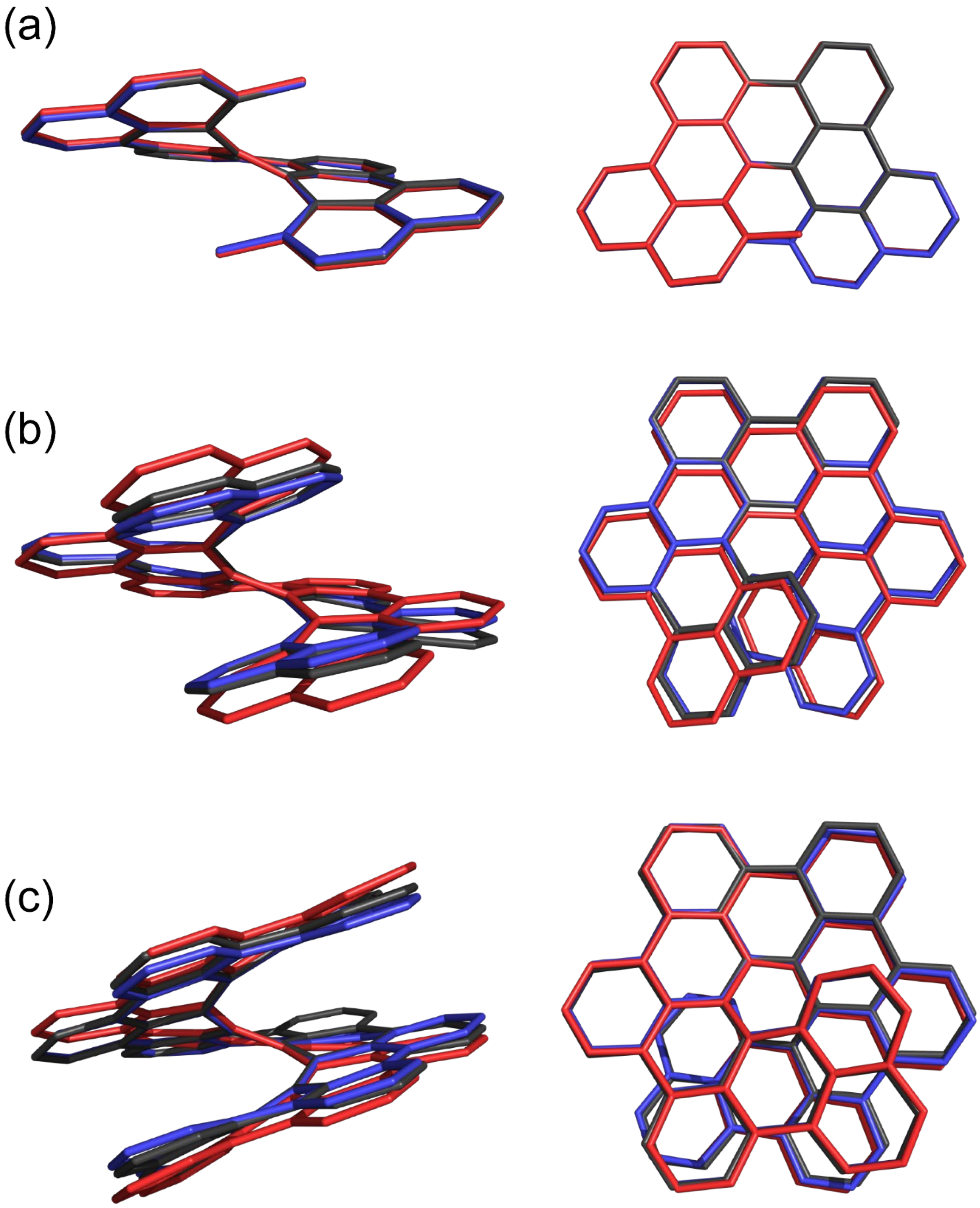

Supplementary Figure 8: Comparison between the X-ray crystal structure and the structure optimized by DFT calculations. Molecular structure of **a 1** (top), **b 2** (middle), and **c 3** (bottom) observed by the X-ray single crystal analysis (black), and the optimized geometry in the S_0 state calculated at the B3LYP/6-311G(2d,p) level of theory (red) and that calculated at the B3LYP-GD3BJ/6-311G(2d,p) level of theory (blue).

2.4. NICS and ACID Calculations of 1 and 3

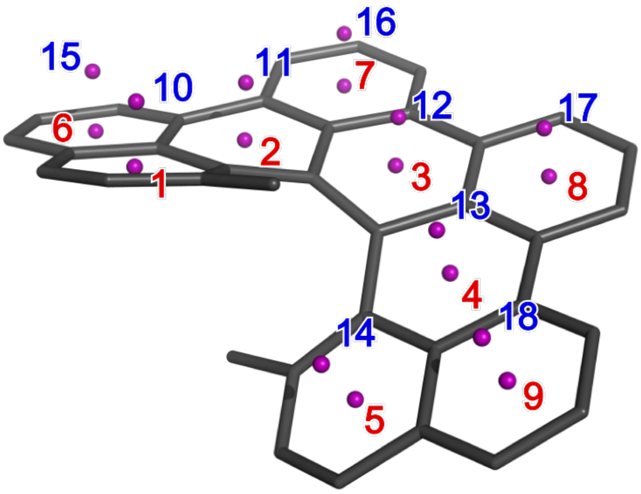

Ring	Atom No.	NICS(0)
A, E	1, 5	−5.68
B, D	2, 4	6.57
C	3	−1.08
K, N	6, 9	−5.97
L, M	7, 8	−6.24

Ring	Atom No.	NICS(1)
A	10	−8.98
B	11	0.61
C	12	−5.21
D	13	1.93
E	14	−7.84
K	15	−9.44
L	16	−8.81
M	17	−9.35
N	18	−8.35

Supplementary Figure 9: NICS calculation of 1. Spatial arrangement of the points where NICS(0) and NICS(1) values were calculated. NICS values calculated at the GIAO-B3LYP/6-311G(2d,p) level of theories are summarized in tables.

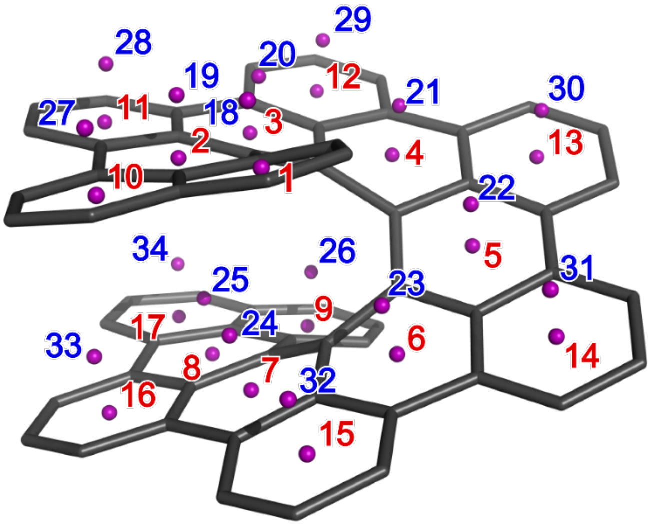

Ring	Atom No.	NICS(0)
A	1	−6.80
B	2	6.23
C	3	−1.97
D	4	5.72
E	5	−1.36
F	6	5.73
G	7	−1.96
H	8	6.24
I	9	−6.97
K	10	−6.06
L	11	−6.01
M	12	−6.13
N	13	−5.38
O	14	−5.39
P	15	−6.12
Q	16	−6.01
R	17	−6.06

Ring	Atom No.	NICS(1)
A	18	−9.83
B	19	0.13
C	20	−6.24
D	21	0.83
E	22	−4.97
F	23	−0.31
G	24	−5.65
H	25	1.00
I	26	−9.76
K	27	−9.44
L	28	−8.92
M	29	−9.36
N	30	−7.91
O	31	−8.83
P	32	−9.11
Q	33	−9.15
R	34	−8.91

Supplementary Figure 10: NICS calculation of 3. Spatial arrangement of the points where NICS(0) and NICS(1) values were calculated. NICS values calculated at the GIAO-B3LYP/6-311G(2d,p) level of theories are summarized in tables.

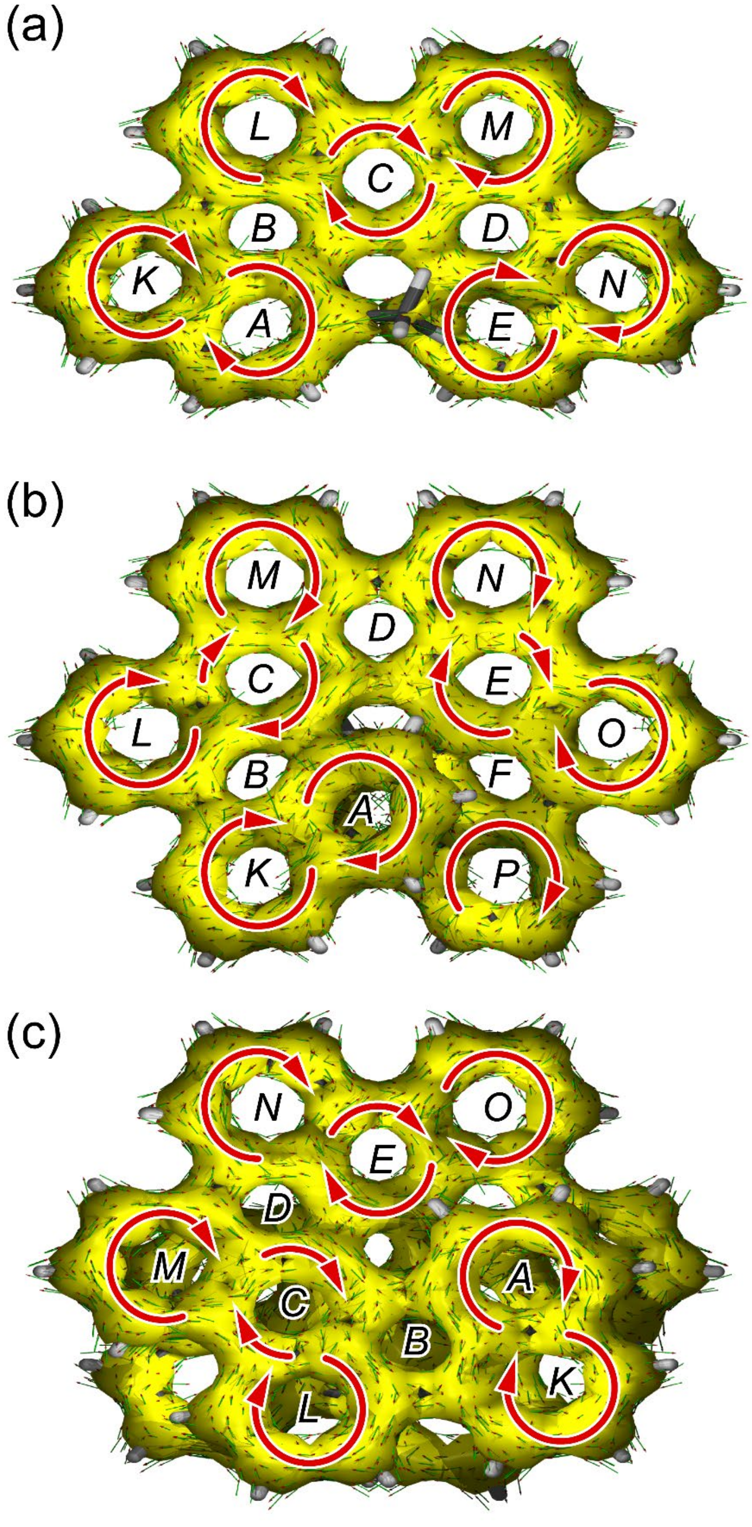

Supplementary Figure 11: ACID calculations. ACID plot for **a 1** (top), **b 2** (middle), and **c 3** (bottom) calculated at the CSGT-B3LYP/6-311G(2d,p) level of theory. The diamagnetic (clockwise) ring currents under the magnetic field parallel to the *z*-axis are highlighted by red arrows.

2.5. UV-vis-NIR Absorption Spectra 1–3 in Toluene

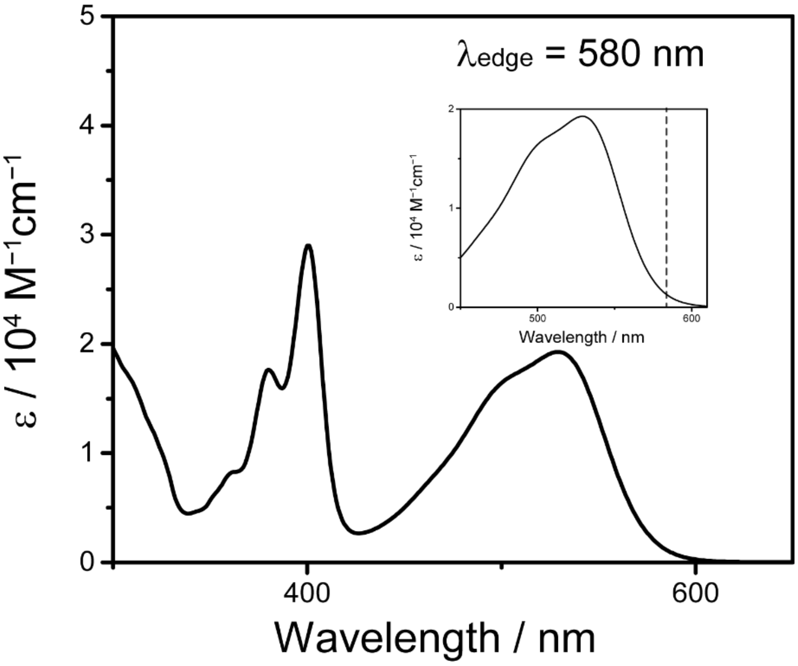

Supplementary Figure 12: UV-vis absorption spectrum of 1 in toluene at 25 °C. The absorption edge of **1** was determined to be 580 nm based on the energy of the longest wavelength optical transition, determined at 10% of the maximum absorption.

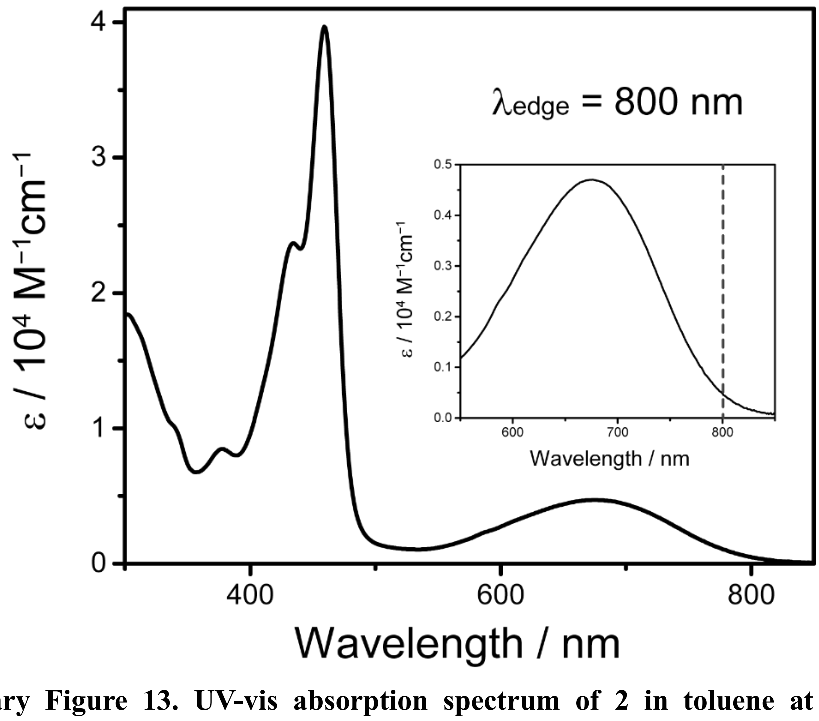

Supplementary Figure 13. UV-vis absorption spectrum of 2 in toluene at 25 °C. The absorption edge of **2** was determined to be 800 nm based on the energy of the longest wavelength optical transition, determined at 10% of the maximum absorption.

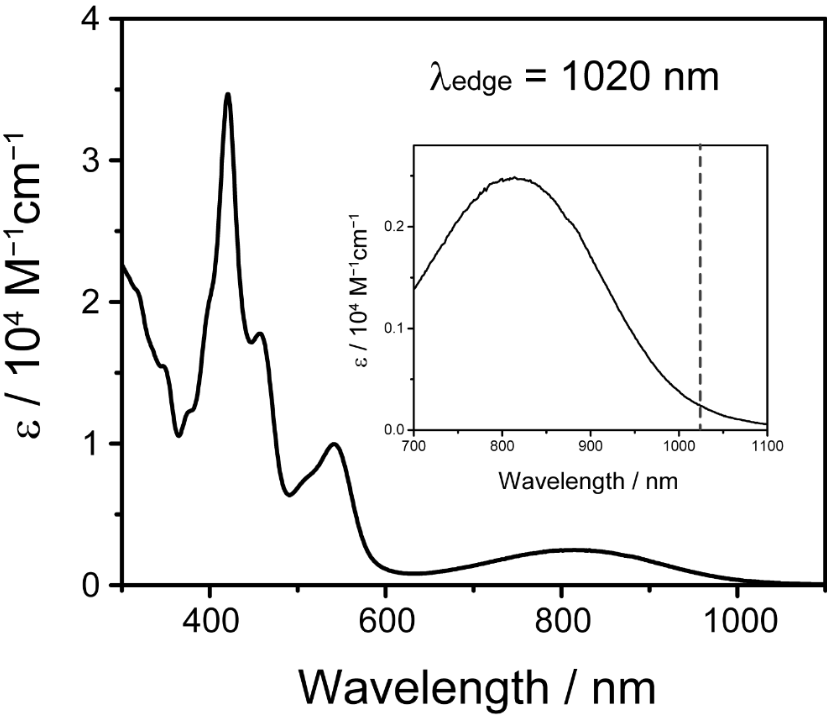

Supplementary Figure 14: UV-vis-NIR absorption spectrum of 3 in toluene at 25 °C. The absorption edge of **3** was determined to be 1020 nm based on the energy of the longest wavelength optical transition, determined at 10% of the maximum absorption.

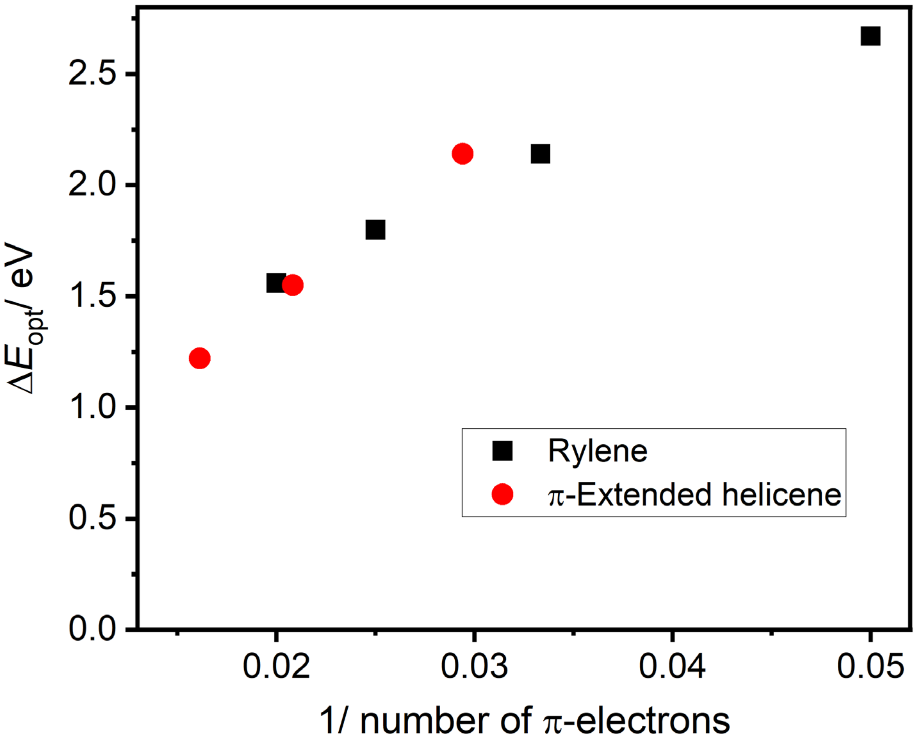

Supplementary Figure 15: Comparison of the optical energy gaps. π-Extended helicenes **1**–**3** (red circle) and rylenes[8] (black square).

Supplementary Table 4: Excitation parameters for the $S_0 \rightarrow S_1$ transitions. π-Extended [*n*]helicenes, [*n*]rylenes, and carbo[*n*]helicenes calculated at the RTD-B3LYP/6-311G(2d,p) level of theory.

Compd.	Excitation energy / eV	Major contribution
π-extended [3]helicene[a]	2.8526	HOMO→LUMO (99%)
π-extended [5]helicene	2.2451	HOMO→LUMO (100%)
π-extended [7]helicene (**2**)	1.6488	HOMO→LUMO (100%)
π-extended [9]helicene (**3**)	1.379	HOMO→LUMO (100%)
π-extended [11]helicene	1.2545	HOMO→LUMO (100%)
π-extended [13]helicene	1.1706	HOMO→LUMO (100%)
π-extended [15]helicene	1.1192	HOMO→LUMO (100%)
π-extended [17]helicene	1.0853	HOMO→LUMO (100%)
[3]rylene[b]	2.1967	HOMO→LUMO (100%)
[4]rylene[c]	1.8256	HOMO→LUMO (100%)
[5]rylene	1.5782	HOMO→LUMO (100%)
[6]rylene	1.397	HOMO→LUMO (100%)
[7]rylene	1.2565	HOMO→LUMO (100%)
[8]rylene	1.1428	HOMO→LUMO (100%)
[9]rylene	1.0472	HOMO→LUMO (100%)
[3]helicene[d]	3.9979	H-1→L (38%), H→L+1 (61%)
[5]helicene	3.2761	H-1→L (38%), H→L+1 (61%)
[7]helicene	3.08	H-1→L (38%), H→L+1 (61%)
[9]helicene	2.9111	H-1→L (41%), H→L+1 (55%)
[11]helicene	2.8053	H-1→L (51%), H→L+1 (38%)
[13]helicene	2.7348	H-1→L (44%), H→L+1 (41%)
[15]helicene	2.6772	H-1→L (41%), H→L+1 (41%)
[17]helicene	2.628	H-1→L (39%), H→L+1 (44%)

[a]The same chemical structure with [2]rylene, or perylene. [b]Terrylene. [c]Quarterrylene. [d]Phenanthrene.

2.6. Electrochemical Measurements of 1–3

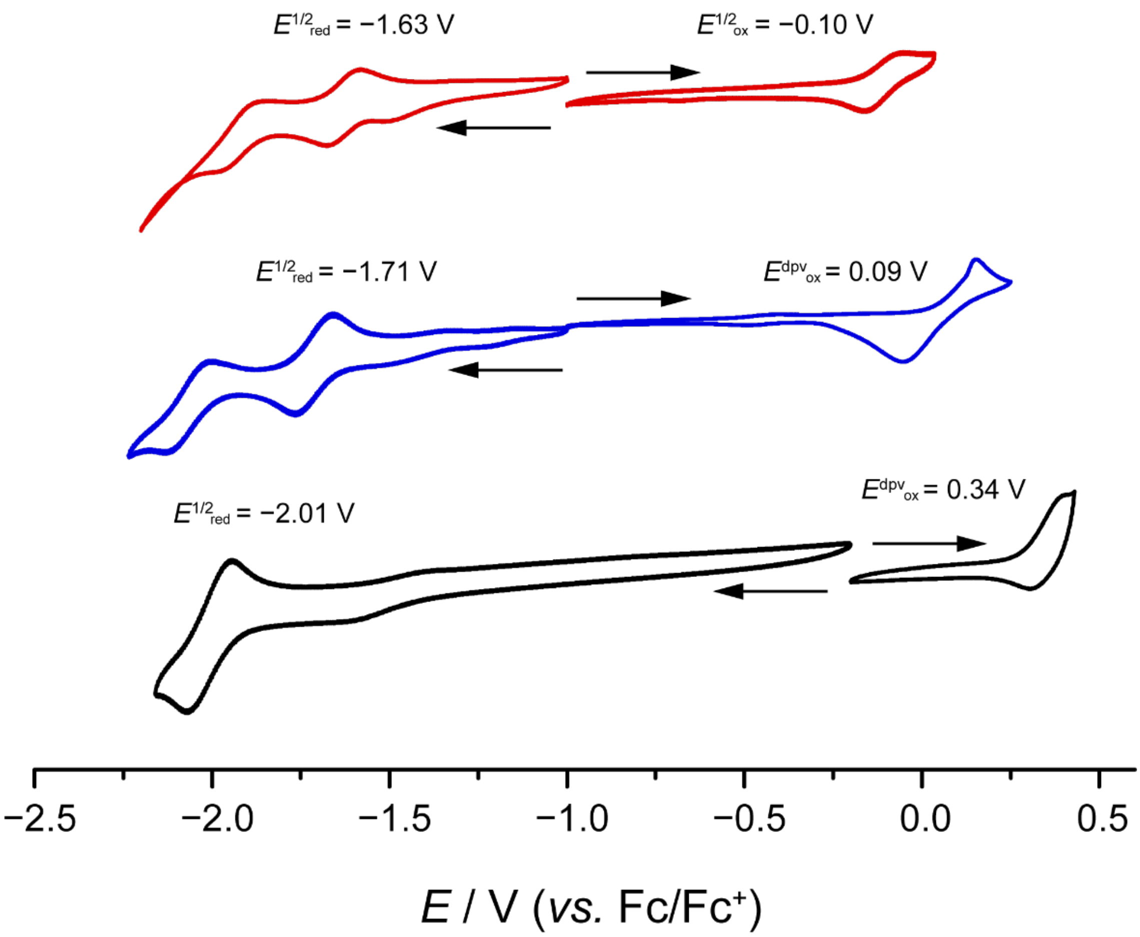

Supplementary Figure 16: Cyclic voltammograms. π-Extended [5]helicene (**1**, bottom), [7]helicene (**2**, middle), [9]helicene (**3**, top) in THF. supporting electrolyte: 0.1 M *n*-Bu_4NPF_6. reference electrode: 0.1 M $AgNO_3$ in MeCN working/counter electrodes: Pt/Pt wire, scan speed: 0.05 V/s, external standard: Fc/Fc^+. E_{ox} of **1** and **2** was determined by DPV measurement.

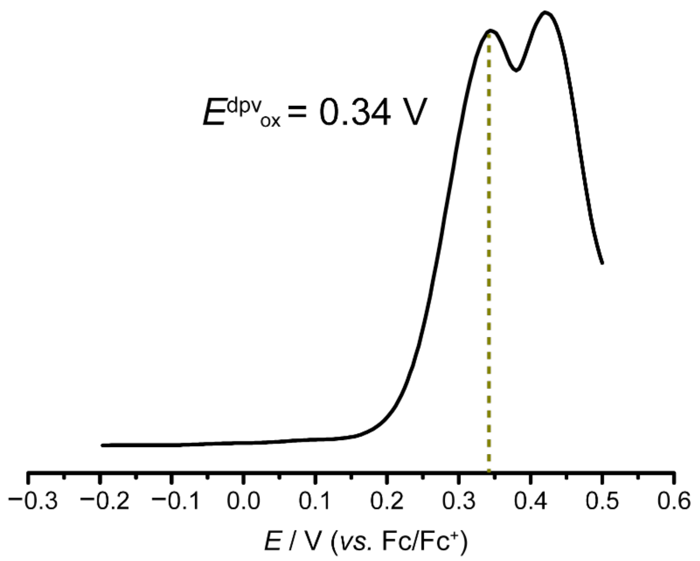

Supplementary Figure 17: Differential pulse voltammograms of compound 1 in THF. Supporting electrolyte: 0.1 M *n*-Bu_4NPF_6. reference electrode: 0.1 M $AgNO_3$ in MeCN working/counter electrodes: Pt/Pt wire, external standard: Fc/Fc^+.

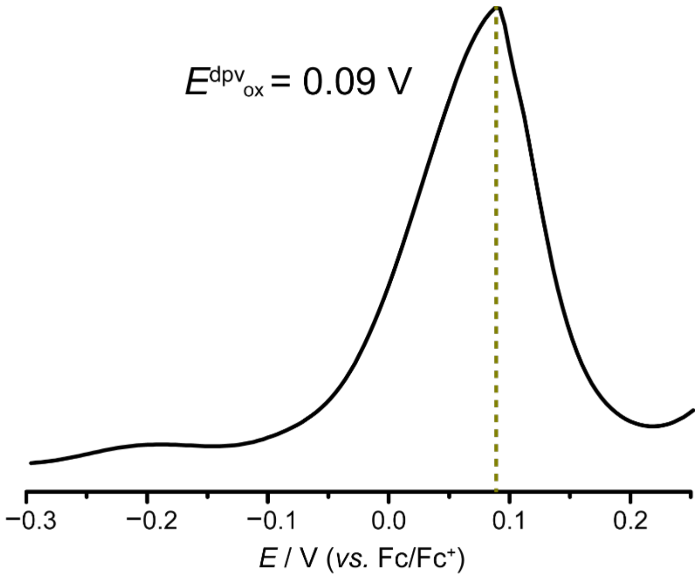

Supplementary Figure 18: Differential pulse voltammograms of compound 2 in THF. Supporting electrolyte: 0.1 M *n*-Bu_4NPF_6. reference electrode: 0.1 M $AgNO_3$ in MeCN working/counter electrodes: Pt/Pt wire, external standard: Fc/Fc^+.

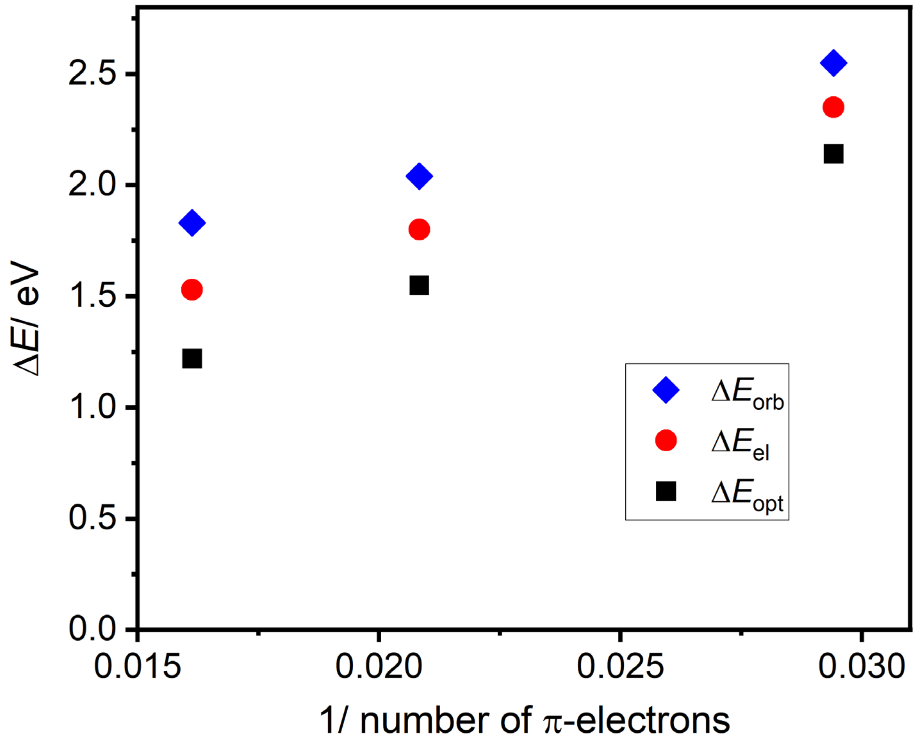

Supplementary Figure 19: Energy gap plot of π-extended helicenes 1–3. Calculated HOMO–LUMO gaps (blue rhombus), electrochemical energy gaps (red circle), and optical energy gaps (black square) are shown.

2.7. Isolation of Enantiomers of 1 and 3 and their CD spectra in Toluene

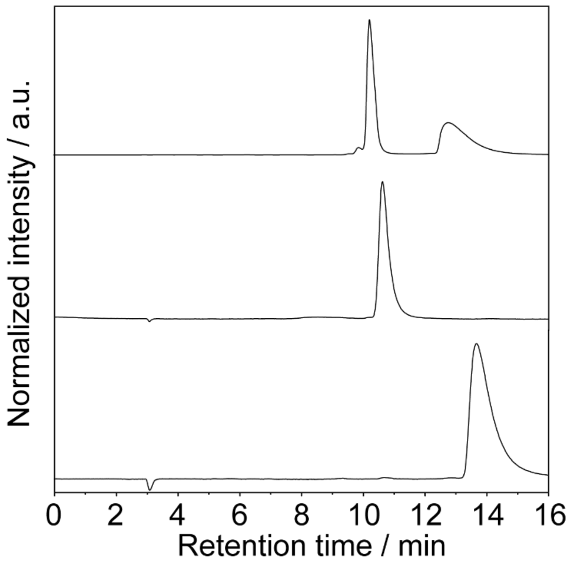

Supplementary Figure 20: HPLC chart for the separation of enantiomers of 1. A racemic solution (top) and those after separation (middle, the first fraction showing the first positive Cotton effect, (*P*)-**1**; bottom, the second fraction showing the first negative Cotton effect, (*M*)-**1**). Conditions: HPLC column, CHIRALPAK IB 250 mm-10 mmϕ (5 µm); eluent, hexane/2-propanol = 98/2; flow rate, 5.0 mL/min; detection wavelength, 254 nm; R_f = 10.2 and 12.8 min for (*P*)-**1** and (*M*)-**1**, respectively. The unit of the *y*-axis is arbitrary (a.u.).

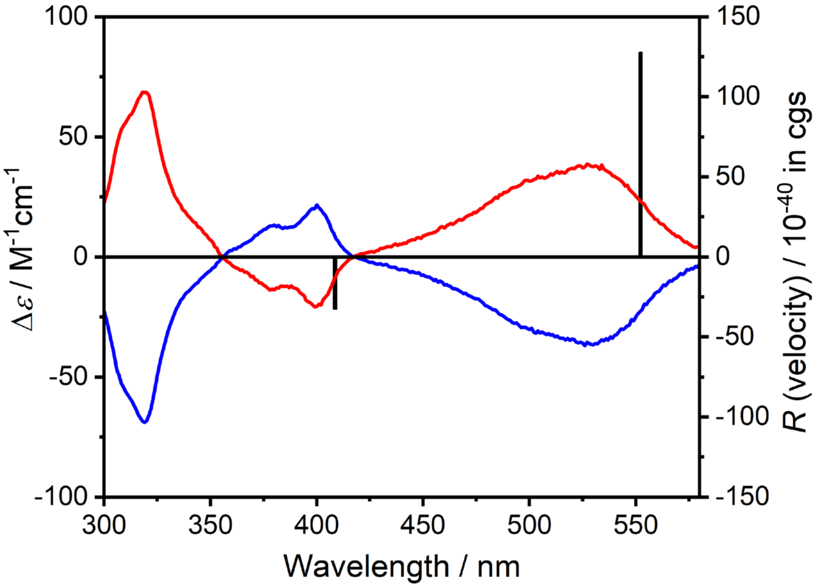

Supplementary Figure 21: Comparison between experimental and theoretical CD signals of 1. Experimental CD spectra of the first fraction (red, (*P*)-**1**) and the second fraction (blue, (*M*)-**1**) in toluene and calculated rotatory strength for the (*P*)-isomer of **1** at the TD-B3LYP/6-311G(2d,p) level of theory (black bar). From the comparison of experimental CD spectra with the result of TD-DFT calculations, the chirality of **1** was determined as follows; (*P*)-**1** and (*M*)-**1** show the first positive and negative Cotton effects, respectively.

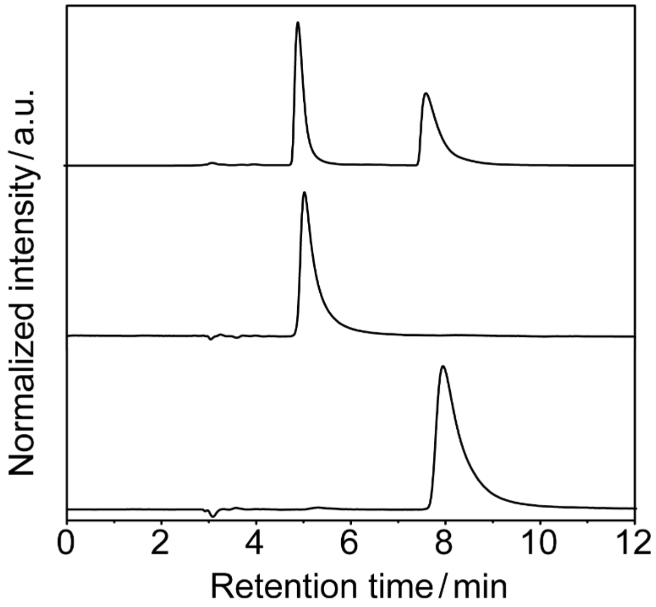

Supplementary Figure 22: HPLC chart for the separation of enantiomers of 3. A racemic solution (top) and those after separation (middle, the first fraction showing the first positive Cotton effect, (*P*)-**3**; bottom, the second fraction showing the first negative Cotton effect, (*M*)-**3**). Conditions: HPLC column, CHIRALPAK IB 250 mm-10 mmϕ (5 μm); eluent, hexane/CH_2Cl_2 60/40; flow rate, 5.0 mL/min; detection wavelength, 254 nm; R_f = 4.9 and 7.6 min for (*P*)-**3** and (*M*)-**3**, respectively. The unit of the *y*-axis is arbitrary (a.u.).

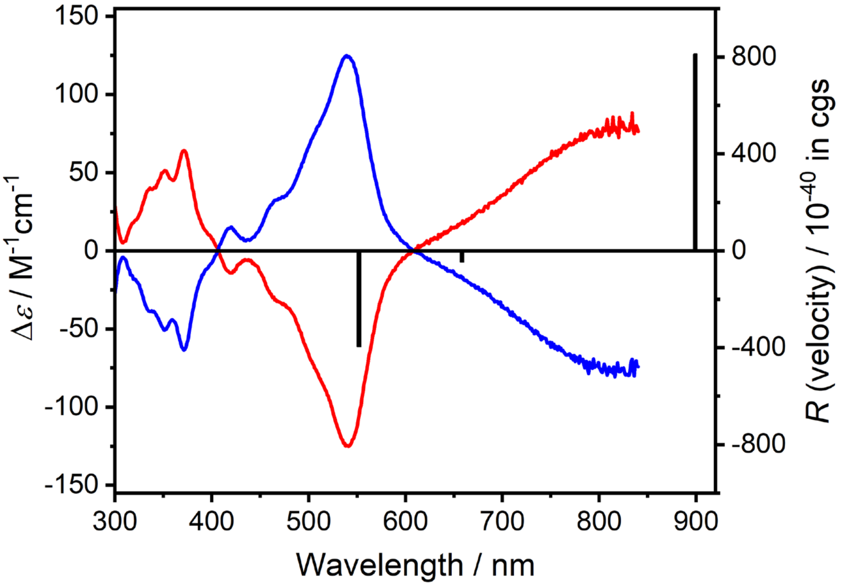

Supplementary Figure 23: Comparison between experimental and theoretical CD signals of 3. Experimental CD spectra of the first fraction (red, (*P*)-**3**) and the second fraction (blue, (*M*)-**3**) in toluene and calculated rotatory strength for the (*P*)-isomer of **3** at the TD-B3LYP/6-311G(2d,p) level of theory (black bar). From the comparison of experimental CD spectra with the result of TD-DFT calculations, the chirality of **1** was determined as follows; (*P*)-**3** and (*M*)-**3** show the first positive and negative Cotton effects, respectively.

The relationship between the helical chirality and the sign of the CD signal is consistent with the carbo[*n*]helicenes: the (*P*)-isomer shows the first positive Cotton effect that appeared at the longest wavelength.[9]

An impressive feature of the CD spectra of **1–3** is that the magnitude of the lowest-energy CD signal increased with increasing helical length (i.e., $\Delta\varepsilon$ = 39, 74, and 74 M^{-1} cm^{-1} for **1**, **2**, and **3**, respectively), despite the decrease in the molar absorption intensity (ε = 19000, 4700, and 2500 M^{-1} cm^{-1} for **1**, **2**, and **3**, respectively), suggesting that the dissymmetry factor of absorption (the g_{CD} value defined as follows: g_{CD} = Δε/ε) is enhanced with increasing helical length: $|g_{CD}|$ = 0.0020 at 529 nm, 0.016 at 680 nm, 0.030 at 809 nm for **1**, **2**, and **3**, respectively. A careful investigations using TD-DFT calculations suggested that the magnitude of the transition magnetic dipole moment is almost independent of the helical length of **1**–**3**, and the significant increase in the g_{CD} value was likely attributed to the difference in the angle of transition electric and magnetic dipole moments (i.e., $\theta_{\mu,m}$ values).

According to the theory, the dissymmetry factor for CD (g_{CD}) is described as follows:

$$g_{CD} = \frac{\varepsilon_{-} - \varepsilon_{+}}{\frac{1}{2}(\varepsilon_{-}+\varepsilon_{+})} = \frac{4\,|\boldsymbol{\mu}|\,|\mathbf{m}|\cos\theta_{\mu,m}}{|\boldsymbol{\mu}|^2 + |\mathbf{m}|^2}$$

where ε is the molar absorption coefficients, subscripts + and − represent the properties corresponding to the right- and left-handed circularly polarized light, **μ** and **m** are the transition electric dipole moment (TEDM) and transition magnetic dipole moment (TMDM) vectors, respectively, for excitation transitions, and $\theta_{\mu,m}$ is the angle between the **μ** and **m**.[10] The magnitudes of TEDMs and TMDMs and their angle $\theta_{\mu,m}$ for the $S_0 \rightarrow S_1$ transition are summarized in Supplementary Table5. It is noted that the angles $\theta_{\mu,m}$ were largely different for **1−3** ($\theta_{\mu,m}$ = 80.0, 47.1, 5.9° for **1**, **2**, and **3**, respectively), whereas the comparable magnitudes of **μ** and **m**. Thus, the difference in g_{CD} of π-extended helicenes is likely attributed to the difference in $\theta_{\mu,m}$ values.

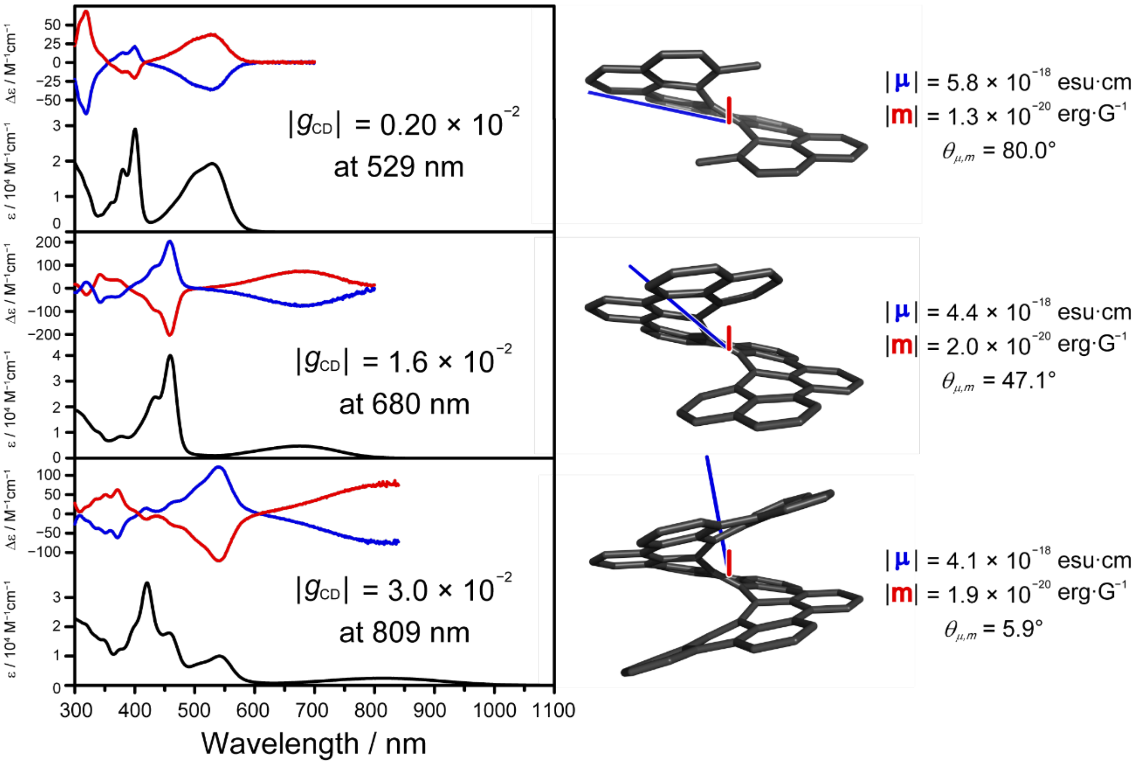

Supplementary Figure 24: The dissymmetry factors of absorption (g_{CD}) for 1–3. UV-vis-NIR absorption and circular dichroism spectra of **1** (top), **2** (middle), and **3** (bottom) in toluene at 25 °C for their (*P*)-isomer (red) and (*M*)-isomer (blue). (b) Transition dipole moments of **1** (top), **2** (middle), and **3** (bottom) for the $S_0 \rightarrow S_1$ transition. The directions of TEDM and TMDM were shown in blue and red lines, respectively.

Supplementary Table 5: Summary of the Chiroptical Properties of 1, 2 and 3

	experimental			calcd. for the $S_1 \rightarrow S_0$ transitions			
Compd.	λ / nm	$\Delta\varepsilon$ / M^{-1} cm^{-1}	$\lvert g_{CD} \rvert$	$\lvert \mu \rvert$ / 10^{-20} esu cm	$\lvert \mathbf{m} \rvert$ / 10^{-20} erg $Gauss^{-1}$	$\cos\theta_{\mu,m}$	$\lvert g_{CD,\ calcd} \rvert$
**1**	529	39	0.0020	580	1.3	0.17	0.0015
**2**	680	74	0.016	440	2.0	0.68	0.012
**3**	809	74	0.030	410	1.9	0.99	0.018

2.8. Thermal Stability of the Isolated Enantiomers of 1 and 3

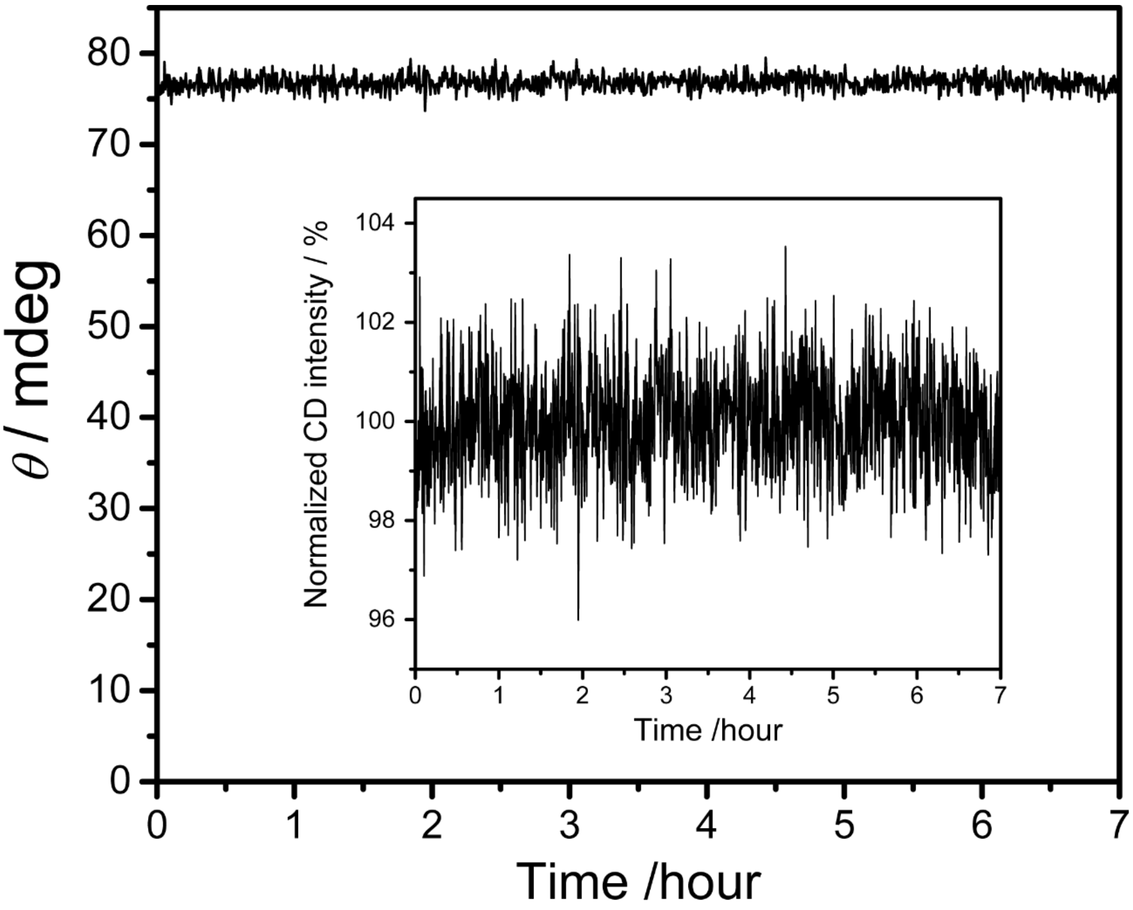

Supplementary Figure 25: Time-dependent change of CD intensity of (*M*)-3 in toluene. Temperature was kept at 90 °C and CD intensity was monitored at 539 nm. No racemization was detected in toluene at 90 °C at least for 7 hours. UV–vis and CD spectra were recorded before and after this measurement, and the spectra were almost completely overlapped, suggesting that the degradation of **3** under this condition is negligible.

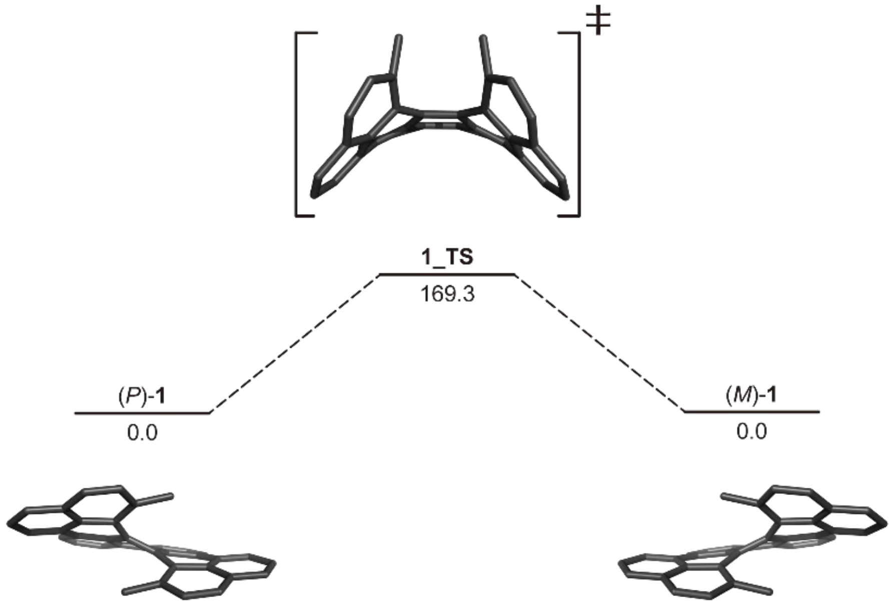

Supplementary Figure 26: Racemization process between (*P*)-1 and (*M*)-1. The relative Gibbs free energy (kJ·mol^{-1}) was calculated at the B3LYP/6-311G(2d,p) level of theory.

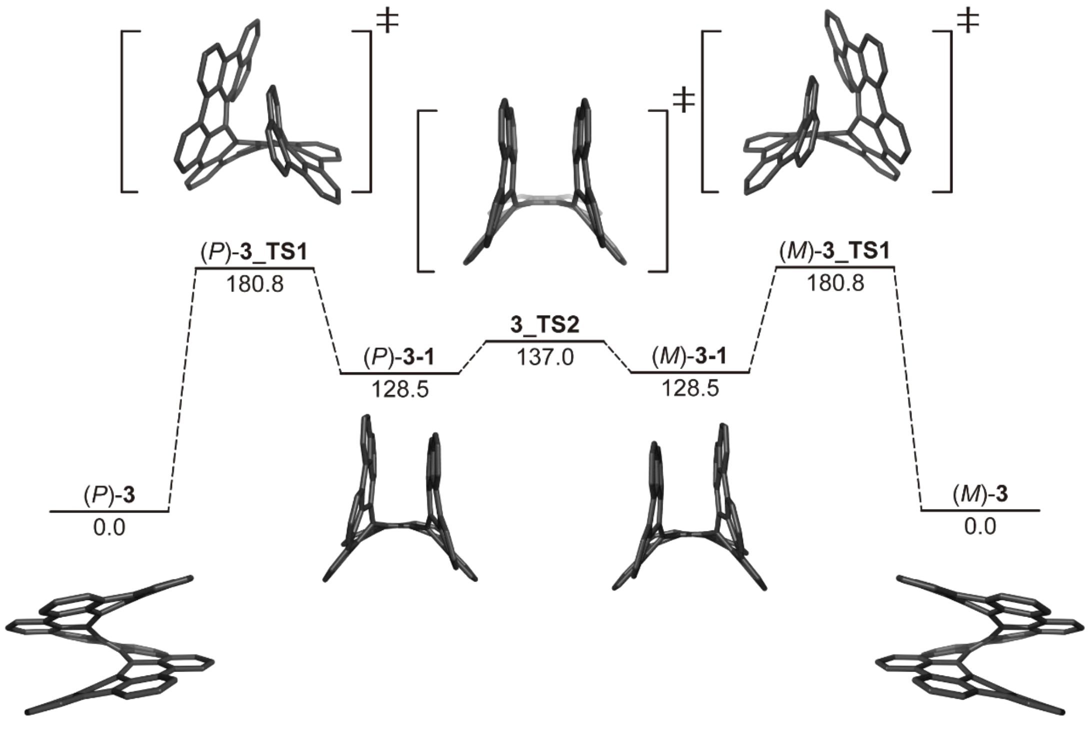

Supplementary Figure 27: Racemization process between (*P*)-3 and (*M*)-3. The relative Gibbs free energy (kJ·mol^{-1}) was calculated at the B3LYP/6-311G(2d,p) level of theory.

2.9. Transient Absorption Spectroscopy of 1 and 3 in Toluene

In the transient absorption (TA) spectroscopy measurements, compounds **1** and **3** in toluene were excited by femtosecond laser pulses at 530 and 800 nm, respectively, to selectively generate their S_1 states. Immediately after excitation, positive TA bands appeared in the wide wavelength regions of 400–1000 nm, together with several negative bands or significant decreases in the magnitude of the positive bands (Figure 5a in the main text). The positive bands are ascribable to the the excited state absorption (ESA) of the $S_1 \rightarrow S_n$ transitions, while the negative ones are safely attributed to the ground state bleaching (GSB) considering the wavelength regions of their steady-state absorption bands, i.e., ca. 400 and 530 nm for **1**, 450 and 680 nm for **2**,[11] and 420, 540, and 850 nm for **3** (Figure 4a in the main text). Most of the positive TA bands almost completely disappeared within a few picoseconds after the excitation, followed by the gradual decreases of relatively long-lasting positive TA bands detected at around 420 and 580 nm for **1**, 480 nm for **2**,[11] and 430, 480, and 570 nm for **3** over several picoseconds. These positive TA bands with different time constants can be attributed to (i) the ultrafast nonradiative decay from the excited state ($S_1 \rightarrow S_0$) and (ii) the subsequent vibrational cooling in the ground state. It is noted that the negative bands of **1** observed at 400 and 530 nm clearly remained even after 100 ps, which are likely due to the photoisomerization reaction from **1** to the closed-ring isomer **1c**, causing a steady decrease in the concentration of **1** upon photoexcitation.

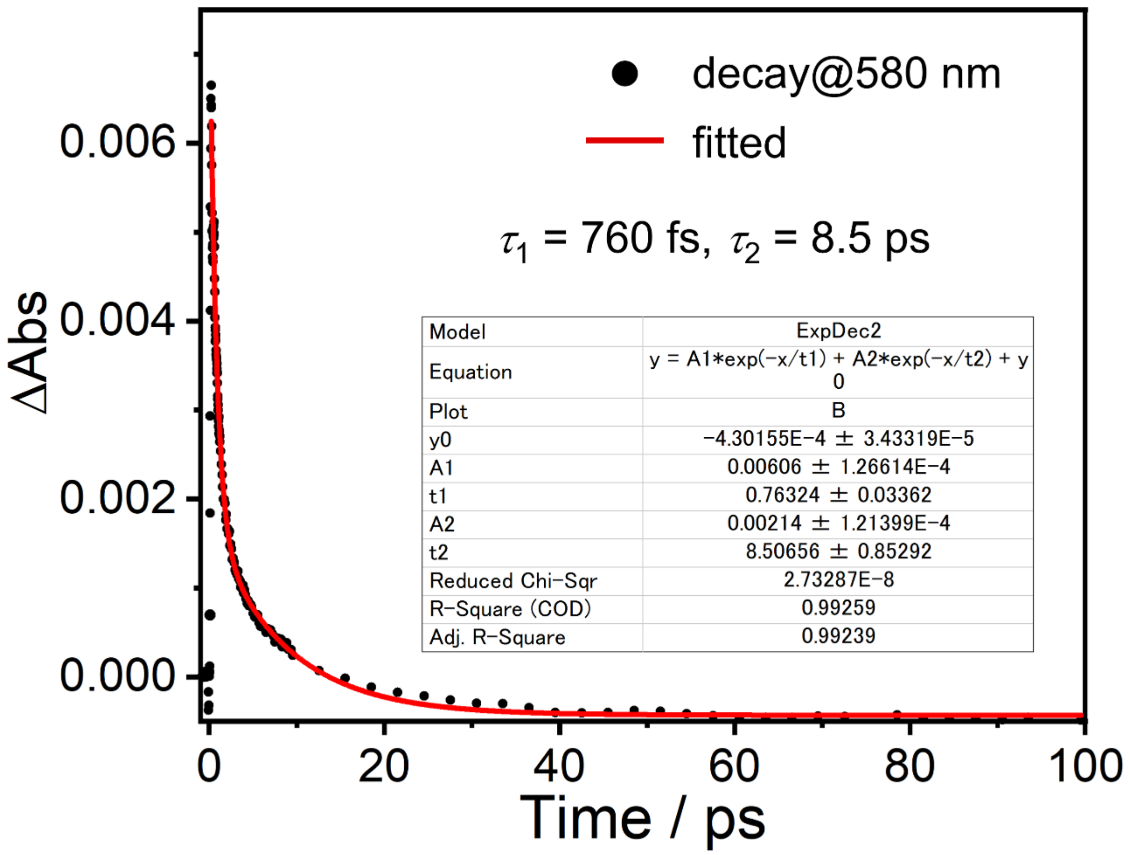

Supplementary Figure 28: Fitting analysis of transient absorption (TA) decay curve of 1. Global fit of the decay of TA spectra of **1** in toluene at room temperature (excited with a femtosecond 530 nm laser pulse) monitored at 580 nm (black circle). Lifetimes of the first and second exponential terms, t_1 and t_2, were optimized as global parameters by a non-linear regression analysis.

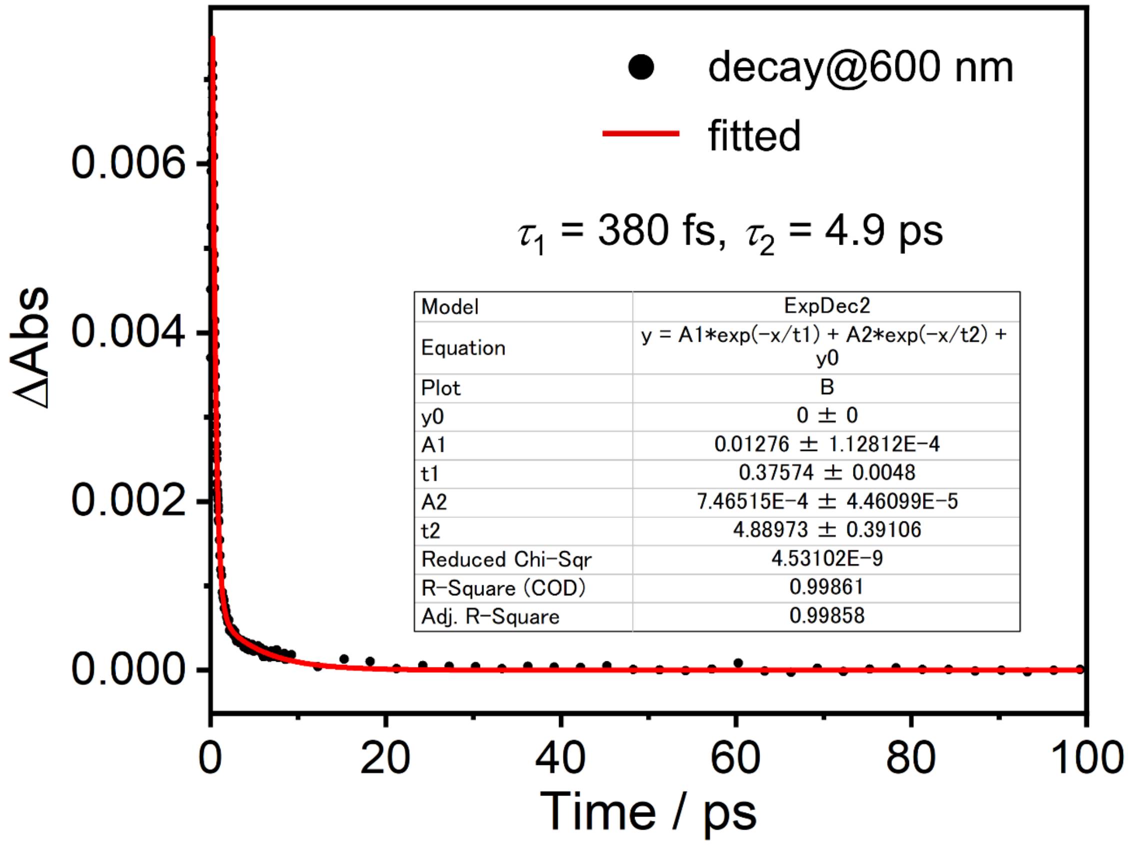

Supplementary Figure 29: Fitting analysis of transient absorption (TA) decay curve of 3. Global fit of the decay of transient absorption spectra of **3** in toluene at room temperature (excited with a femtosecond 800 nm laser pulse) monitored at 600 nm (black circle). Lifetimes of the first and second exponential terms, t_1 and t_2, were optimized as global parameters by a non-linear regression analysis.

Supplementary Table 6: Experimental summary of the excited-state dynamics of π-extended helicenes, helicenes and rylenes

Compd.	Φ_f	τ_{S1} / ps	k_f / ns^{-1}	k_{nr} / ns^{-1}
**1**	n.d.	0.76	n.d.	1300
**2**	n.d.	1.2	n.d.	830
**3**	n.d.	0.38	n.d.	2600
[5]helicene[12]	0.04	26000	0.0016	0.037
[7]helicene[12]	0.021	14000	0.0015	0.070
[9]helicene[12]	0.014	9600	0.0015	0.10
[2]rylene[13]	0.98	3950	0.25	0.0051
[3]rylene[14]	0.70	3800	0.18	0.079
[4]rylene[14]	0.05	n.d.	n.d.	n.d.

2.10. Structures of the Franck–Condon (FC) and minimum energy conical intersection (MECI) geometries of 1 and 3

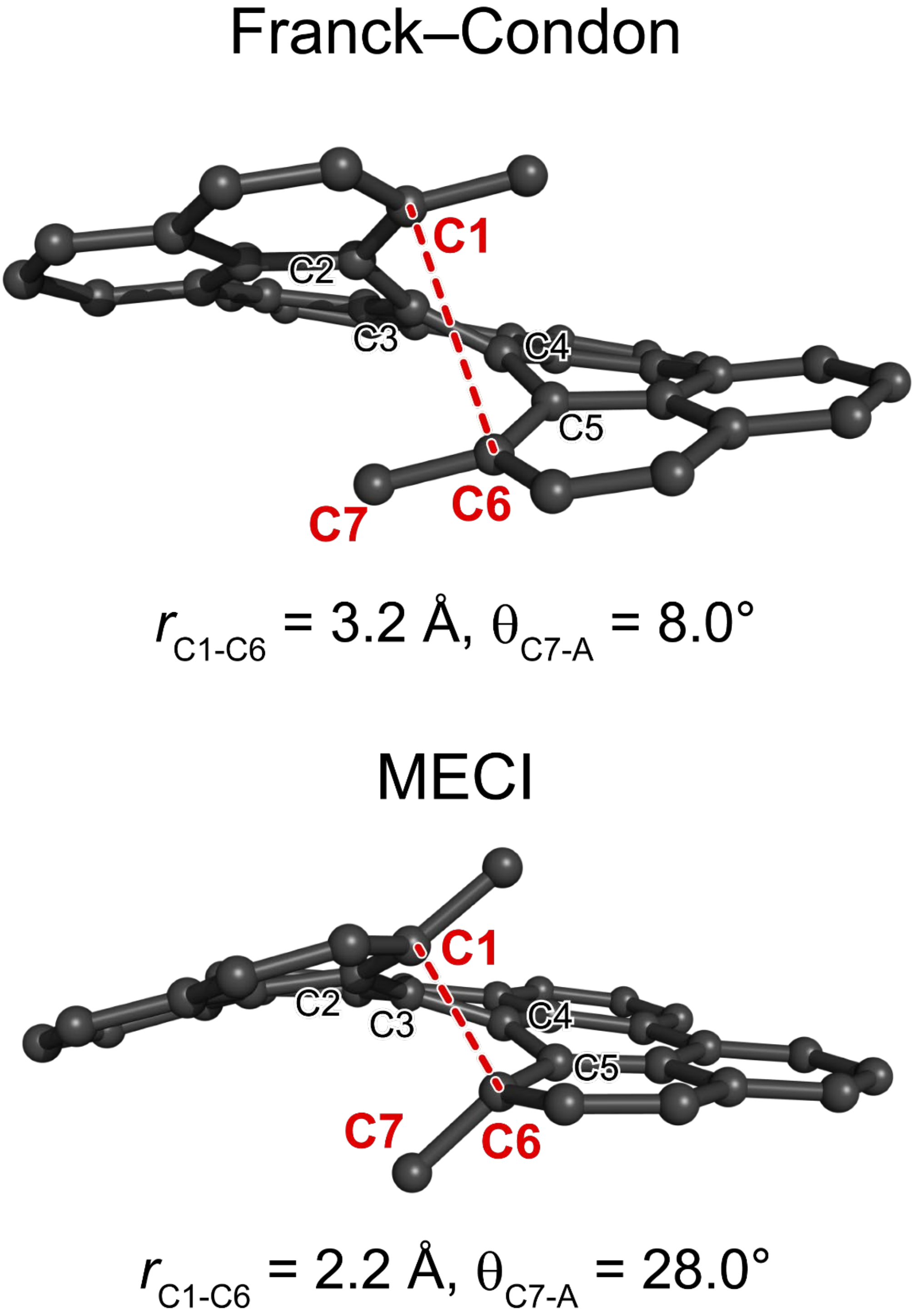

Supplementary Figure 30: FC and MECI geometries of π-extended [5]helicene 1. The geometries were optimized using SF-ωb97xd/6-31G(d) level of theory.

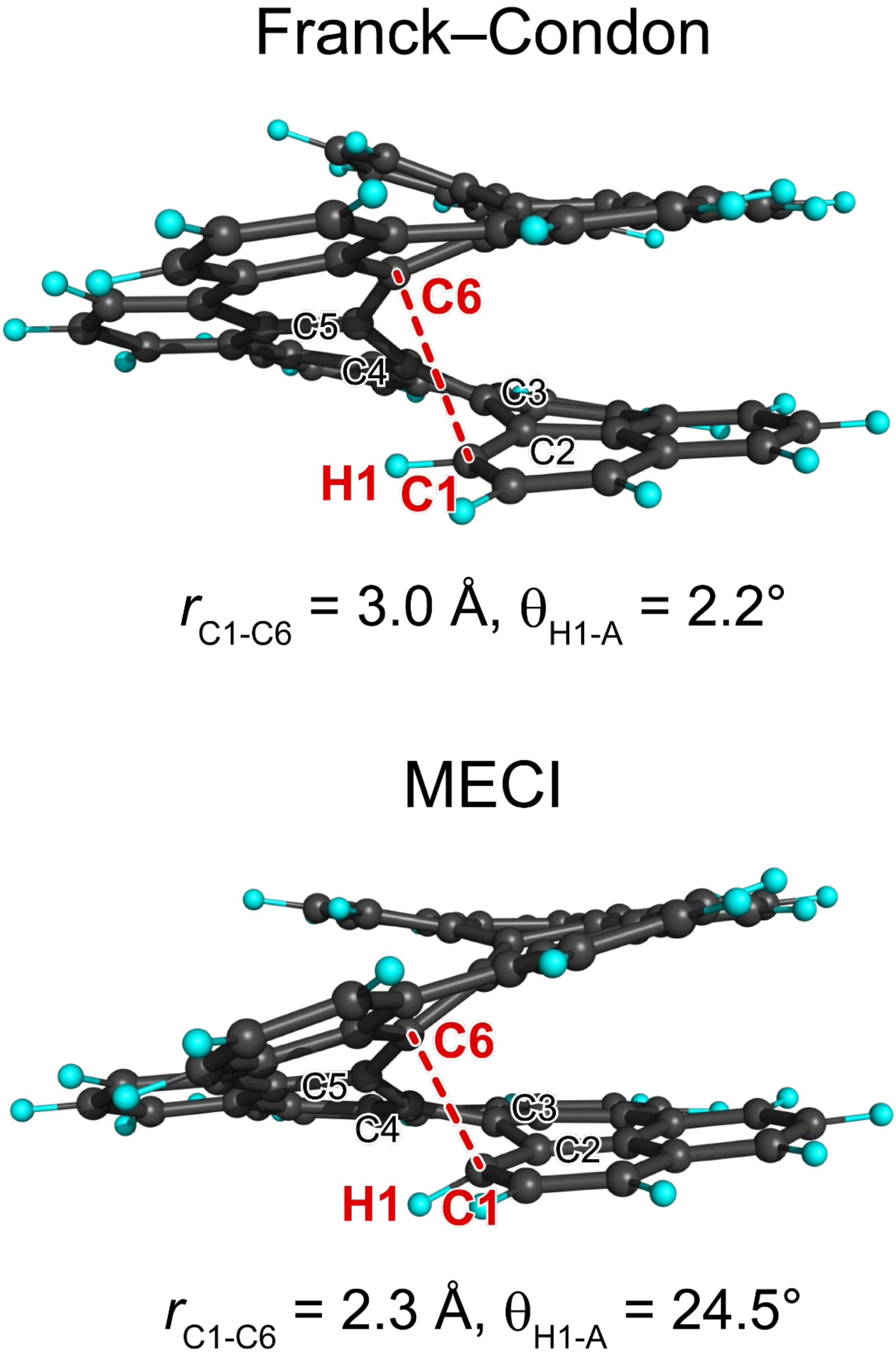

Supplementary Figure 31: FC and MECI geometries of π-extended [9]helicene 3. The geometries were optimized using SF-ωb97xd/6-31G(d) level of theory.

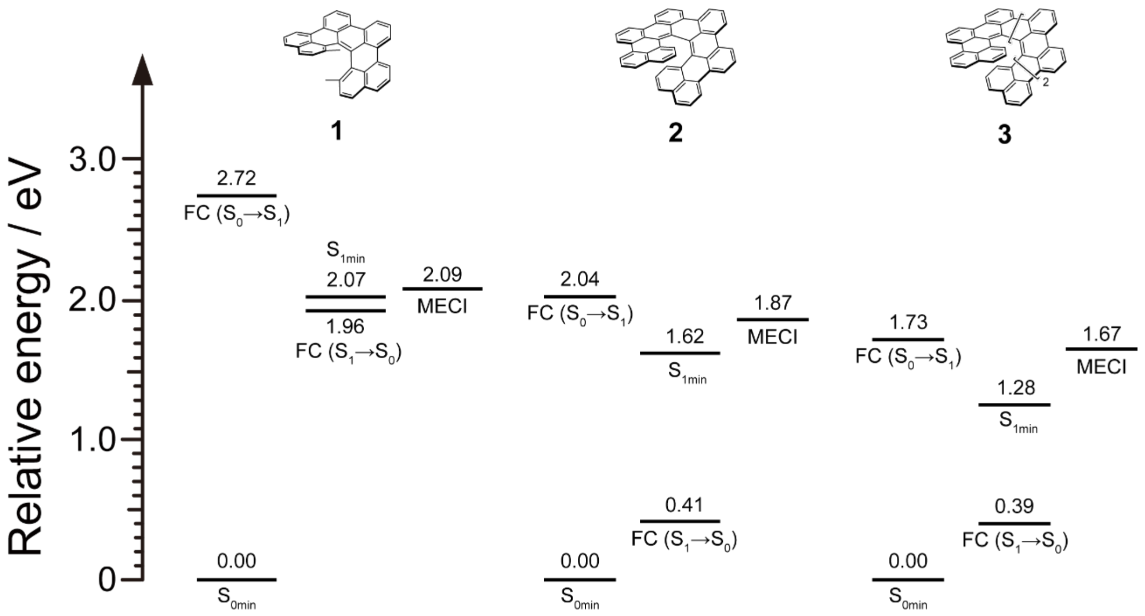

Supplementary Figure 32: Potential energies of π-extended [5]-, [7]-, and [9]helicene 1–3. The energies are given relative to the S_0 energy in the ground state equilibrium structure (See Supplementary Table 7 for detailed values).

Supplementary Table 7: Theoretical summary of the excited-state dynamics of π-extended helicenes and helicenes[a]

Compd.	$E_{FC(S0\rightarrow S1)}$ / eV	E_{S1min} / eV	$E_{FC(S1\rightarrow S0)}$ / eV	E_{MECI} / eV	$\Delta E_{MECI\text{-}FC(S0\rightarrow S1)}$[b] / eV	$\Delta E_{MECI\text{-}S1min}$[c] / eV
**1**	2.72	2.07	1.96	2.09	−0.63	0.02
**2**	2.04	1.62	0.41	1.87	−0.17	0.25
**3**	1.73	1.28	0.39	1.67	−0.06	0.39
[5]helicene	3.38[d]	n.d.	n.d.	4.01	0.63	n.d.
[7]helicene	3.60[d]	n.d.	n.d.	4.10	0.50	n.d.
[9]helicene	2.68[d]	n.d.	n.d.	3.53	0.85	n.d.

[a]The optimized structures and electronic energies with respect to the ground state (S_0) structure were calculated at the SF-ωB97xd/6-31G(d) level of theory for π-extended helicenes and for the MECI structures of carbo[*n*]helicenes (*n* = 5, 7, and 9). The energy diagram of compounds **1**–**3** is shown in Supplementary Figure 32. [b]$\Delta E_{MECI\text{-}FC(S0\rightarrow S1)}$, the relative energy of minimum energy conical intersection (MECI) with respective to the Franck-Condon (FC) state. [c]$\Delta E_{MECI\text{-}S1min}$, the relative energy required to access the MECI from the energy minimum in the S_1 state. [d]The RBHHLYP/6-31G(d) level of theory was used for geometrical optimization of carbo[*n*]helicenes (*n* = 5, 7, and 9) in the S_0 state, and resulting optimized structure was used for the calculation of $E_{FC(S0\rightarrow S1)}$ values at the SF-ωB97xd/6-31G(d) level of theory.

Supplementary Table 8: Emission properties of recently reported π-extended helicenes

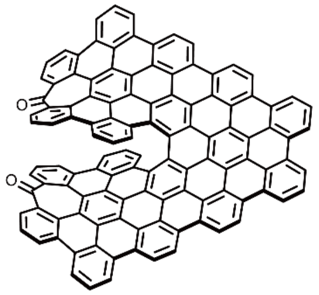

A ($C_{114}H_{30}$)
Campaña, 2018

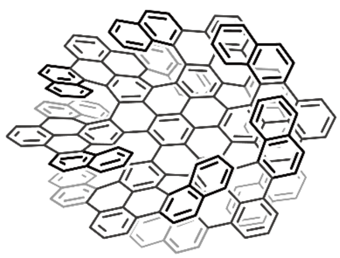

B ($C_{198}H_{72}$)
Wang, 2019

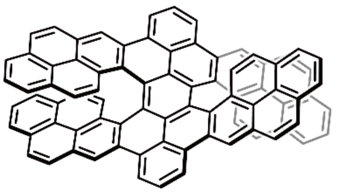

C ($C_{82}H_{36}$)
Müllen and Narita, 2019

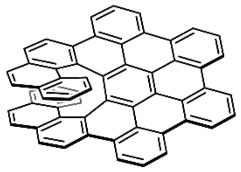

D ($C_{58}H_{26}$)
Müllen and Narita, 2021

Compd.	Solvent	$\lambda_{em,max}$ / nm	Φ_f	$\langle\tau_{S1}\rangle$ / ns	k_f / ns^{-1}	k_{nr} / ns^{-1}
**A**[15]	CH_2Cl_2	610	0.098	18	0.005	0.050
**B**[16]	CH_2Cl_2	870	0.046	14	0.003	0.068
**C**[17]	CH_2Cl_2	697	0.035	1	0.04	0.9
**D**[18]	THF	528	0.41	7.1	0.058	0.083

Supplementary Table 9: Cartesian coordinates (Å) of the optimized structure of compound **1** in the ground state (S_0) calculated at the B3LYP/6-311G(2d,p) level of theory.

```
  1  C-2.821628    1.552658   -0.184178
  2  C-3.502996    2.751361   -0.372987
  3  C-2.824924    3.964682   -0.426518
  4  C-1.452207    3.999008   -0.295296
  5  C-0.721347    2.821263   -0.079000
  6  C-1.411455    1.585007   -0.001563
  7  C 0.721333    2.821265    0.078993
  8  C 1.411446    1.585011    0.001557
  9  C 0.680779    0.346443   -0.156040
 10  C-0.680784    0.346441    0.156030
 11  C 1.452189    3.999013    0.295291
 12  C 2.824905    3.964692    0.426519
 13  C 3.502982    2.751372    0.372991
 14  C 2.821619    1.552667    0.184179
 15  C-1.436537   -0.823137    0.633054
 16  C-3.517874    0.259060   -0.188126
 17  C 3.517869    0.259071    0.188132
 18  C 1.436539   -0.823135   -0.633059
 19  C-0.889544   -1.832081    1.435457
 20  C-1.665404   -2.982623    1.711201
 21  C-2.938918   -3.139598    1.241039
 22  C-3.573779   -2.079295    0.555448
 23  C-2.835208   -0.882940    0.321122
 24  C-4.919914   -2.164553    0.135084
 25  C-5.528942   -1.087645   -0.457567
 26  C-4.837160    0.123789   -0.595195
 27  C 2.835209   -0.882932   -0.321118
 28  C 3.573786   -2.079285   -0.555437
 29  C 2.938935   -3.139591   -1.241032
 30  C 1.665425   -2.982621   -1.711205
 31  C 0.889557   -1.832082   -1.435468
 32  C 4.837153    0.123805    0.595210
 33  C 5.528940   -1.087628    0.457589
 34  C 4.919919   -2.164538   -0.135064
 35  C 0.465405   -1.773891    2.092920
 36  C-0.465380   -1.773897   -2.092953
 37  H-4.579187    2.750703   -0.478573
 38  H-3.377507    4.883294   -0.583763
 39  H-0.937814    4.946245   -0.380626
 40  H 0.937793    4.946247    0.380621
 41  H 3.377485    4.883306    0.583767
 42  H 4.579172    2.750718    0.478582
 43  H-1.216346   -3.766883    2.310950
 44  H-3.490918   -4.053186    1.431293
 45  H-5.462044   -3.089340    0.297255
 46  H-6.556696   -1.155980   -0.793772
 47  H-5.359279    0.968495   -1.023907
 48  H 3.490940   -4.053177   -1.431282
 49  H 1.216375   -3.766881   -2.310960
 50  H 5.359267    0.968514    1.023924
 51  H 6.556692   -1.155958    0.793800
 52  H 5.462053   -3.089323   -0.297232
 53  H 0.835406   -0.754825    2.189500
 54  H 0.396529   -2.210082    3.092311
 55  H 1.217416   -2.343882    1.542696
 56  H-1.217399   -2.343889   -1.542740
 57  H-0.835383   -0.754832   -2.189543
 58  H-0.396487   -2.210091   -3.092341
```

```
----------------------------------------------------------------------
B3LYP/6-311g(2d,p)
E(RB3LYP) = -1385.50626416 hartree
# of imaginary frequencies = 0

<Thermal energies>
----------------------------------------------------------------------
Zero-point correction=          0.460399 (Hartree/Particle)
 Thermal correction to Energy=        0.485081
 Thermal correction to Enthalpy=        0.486025
 Thermal correction to Gibbs Free Energy=      0.408949
 Sum of electronic and zero-point Energies=    -1385.045865
 Sum of electronic and thermal Energies=       -1385.021184
 Sum of electronic and thermal Enthalpies=     -1385.020239
 Sum of electronic and thermal Free Energies=     -1385.097315
----------------------------------------------------------------------

<Transition moments>
----------------------------------------------------------------------
 Ground to excited state transition electric dipole moments (Au):
       state          X           Y           Z        Dip. S.      Osc.
----------------------------------------------------------------------
         1        -2.2018      0.0000      0.5507      5.1512      0.2833
         2         0.0000     -0.1941      0.0000      0.0377      0.0025
         3         0.0000      1.7726      0.0000      3.1422      0.2336
         4         0.2504      0.0000      0.0552      0.0657      0.0057
         5         0.0000     -0.1674      0.0000      0.0280      0.0025
         6        -0.3452      0.0000     -0.1471      0.1408      0.0128
         7         0.3266      0.0000     -0.0154      0.1069      0.0097
         8         0.0000      0.0696      0.0000      0.0048      0.0004
         9         0.0000     -0.0831      0.0000      0.0069      0.0006
        10         0.8740     -0.0003      0.2269      0.8154      0.0758
----------------------------------------------------------------------
```

Ground to excited state transition magnetic dipole moments (Au):

state	X	Y	Z
1	-0.0965	0.0000	-1.3492
2	0.0000	-0.0033	0.0000
3	0.0000	0.0769	0.0000
4	-0.0554	0.0000	0.1983
5	0.0000	0.0025	0.0000
6	-0.0620	0.0000	0.3594
7	0.3838	0.0000	-1.8752
8	0.0000	0.0823	0.0001
9	0.0000	0.1355	0.0000
10	0.5410	0.0006	-3.0169

Rotatory Strengths (R) in cgs (10**-40 erg-esu-cm/Gauss)

state	XX	YY	ZZ	R(velocity)	E-M Angle
1	363.6044	336.1722	-316.8171	127.6532	79.97
2	0.6802	0.0000	-1.1665	-0.1621	90.00
3	-74.5462	0.0000	-23.9196	-32.8219	90.00
4	-2.1736	-1.4868	5.0227	0.4541	87.79
5	-0.9826	0.0000	1.2529	0.0901	90.00
6	50.1915	-14.9301	-12.0199	7.7472	76.41
7	-14.8421	-39.9838	-57.3253	-37.3837	105.13
8	-3.3856	0.0000	-0.9537	-1.4464	90.00
9	3.5488	0.0000	4.0158	2.5215	90.00
10	225.4364	85.5128	-136.0799	58.2898	84.89

Rotatory Strengths (R) in cgs (10**-40 erg-esu-cm/Gauss)

state	XX	YY	ZZ	R(length)
1	-150.2159	0.0000	525.3985	125.0609
2	0.0000	-0.4554	0.0000	-0.1518
3	0.0000	-96.4501	0.0000	-32.1500
4	9.8047	0.0000	-7.7449	0.6866
5	0.0000	0.2908	0.0000	0.0969
6	-15.1328	0.0000	37.3834	7.4169
7	-88.6296	0.0000	-20.4220	-36.3505
8	0.0000	-4.0539	0.0000	-1.3513
9	0.0000	7.9613	0.0000	2.6538
10	-334.3603	0.0001	484.1543	49.9314

<Summary of chiroptical properties>

state	|TEDM| (10-20 esu-cm)	|TMDM| (10-20 erg/Gauss)	cos(theta) (E-M Angle)	g (4R/D)	Eex (nm)
1	576.88	1.254	0.173	0.0015	552.24
2	49.34	0.003	-0.999	-0.0002	455.71
3	450.55	0.071	-1.000	-0.0006	408.60
4	65.17	0.191	0.055	0.0007	351.86
5	42.55	0.002	0.980	0.0002	346.10
6	95.38	0.338	0.230	0.0033	335.34
7	83.11	1.775	-0.246	-0.0210	334.37
8	17.69	0.076	-0.999	-0.0172	332.11
9	21.12	0.126	1.000	0.0238	329.41
10	229.51	2.843	0.076	0.0038	326.89

Supplementary Table 10: Cartesian coordinates (Å) of the optimized structure of compound **1** in the transition state calculated at the B3LYP/6-311G(2d,p) level of theory.

1	C	2.772573	-0.241586	3.347537
2	C	1.582935	-0.231571	2.626603
3	C	1.547030	0.410553	1.364066
4	C	2.769125	0.731024	0.726635
5	C	3.943362	0.752263	1.496384
6	C	3.931149	0.314200	2.807030
7	C	0.277669	0.656508	0.699202
8	C	0.277669	0.656508	-0.699202
9	C	1.547030	0.410553	-1.364066
10	C	2.769125	0.731024	-0.726635
11	C	1.582935	-0.231571	-2.626603
12	C	2.772573	-0.241586	-3.347537
13	C	3.931149	0.314200	-2.807030
14	C	3.943362	0.752263	-1.496384
15	C	-0.900864	0.599187	-1.602665
16	C	-0.900864	0.599187	1.602665
17	C	0.371730	-0.929950	-3.068013
18	C	0.371730	-0.929950	3.068013
19	C	-0.874947	-0.463702	2.569109
20	C	-2.077878	-0.927540	3.169661
21	C	-3.270557	-0.203855	2.915829
22	C	-3.190849	1.001398	2.280516
23	C	-1.987504	1.467169	1.682386
24	C	0.385576	-1.964421	3.989941
25	C	-0.808308	-2.535358	4.454500
26	C	-2.020712	-2.012430	4.071038
27	C	-1.987504	1.467169	-1.682386
28	C	-3.190849	1.001398	-2.280516
29	C	-3.270557	-0.203855	-2.915829
30	C	-2.077878	-0.927540	-3.169661
31	C	-0.874947	-0.463702	-2.569109
32	C	-2.020712	-2.012430	-4.071038
33	C	-0.808308	-2.535358	-4.454500
34	C	0.385576	-1.964421	-3.989941
35	C	-1.852989	2.955068	-1.499148
36	C	-1.852989	2.955068	1.499148
37	H	2.801114	-0.683542	4.335300
38	H	4.880179	1.055013	1.047824
39	H	4.842689	0.335400	3.392321
40	H	2.801114	-0.683542	-4.335300
41	H	4.842689	0.335400	-3.392321
42	H	4.880179	1.055013	-1.047824
43	H	-4.209343	-0.557883	3.326341
44	H	-4.060933	1.648316	2.238487
45	H	1.330574	-2.341194	4.360592
46	H	-0.768230	-3.362967	5.152598
47	H	-2.944694	-2.398874	4.486468
48	H	-4.060933	1.648316	-2.238487
49	H	-4.209343	-0.557883	-3.326341
50	H	-2.944694	-2.398874	-4.486468
51	H	-0.768230	-3.362967	-5.152598
52	H	1.330574	-2.341194	-4.360592
53	H	-1.802332	3.390213	-2.503742
54	H	-0.939291	3.240115	-0.988110
55	H	-2.708964	3.413666	-1.006215
56	H	-2.708964	3.413666	1.006215
57	H	-0.939291	3.240115	0.988110
58	H	-1.802332	3.390213	2.503742

```
 ---------------------------------------------------------------------
B3LYP/6-311g(2d,p)
E(RB3LYP) = -1385.44224098 hartree
# of imaginary frequencies = 1

<Thermal energies>
---------------------------------------------------------------------
 Zero-point correction=                           0.459236 (Hartree/Particle)
 Thermal correction to Energy=                        0.483033
 Thermal correction to Enthalpy=                      0.483977
 Thermal correction to Gibbs Free Energy=             0.409398
 Sum of electronic and zero-point Energies=              -1384.983005
 Sum of electronic and thermal Energies=                 -1384.959208
 Sum of electronic and thermal Enthalpies=               -1384.958264
 Sum of electronic and thermal Free Energies=            -1385.032843
---------------------------------------------------------------------
```

Supplementary Table 11: Cartesian coordinates (Å) of the optimized structure of compound **2** in the ground state (S_0) calculated at the B3LYP/6-311G(2d,p) level of theory.

1	C	-4.534884	-2.747500	-1.467259
2	C	-4.355250	-1.563365	-2.133351
3	C	-3.082825	-0.945371	-2.160834
4	C	-1.997141	-1.554533	-1.459329
5	C	-2.178432	-2.832879	-0.858709
6	C	-3.448796	-3.387306	-0.849819
7	C	-2.855094	0.245792	-2.884564
8	C	-1.597929	0.796063	-2.930510
9	C	-0.549495	0.233505	-2.192351
10	C	-0.737382	-0.889733	-1.397464
11	C	-0.996565	-3.539996	-0.341957
12	C	0.340753	-1.459283	-0.578784
13	C	0.252253	-2.857281	-0.238767
14	C	1.381601	-3.537116	0.296178
15	C	1.246316	-4.871643	0.706623
16	C	0.041094	-5.529374	0.583027
17	C	-1.066741	-4.869913	0.060857
18	C	1.431101	-0.728998	-0.102351
19	C	2.657021	-1.430447	0.178271
20	C	2.648550	-2.833662	0.375299
21	C	3.887352	-0.716695	0.179075
22	C	5.059504	-1.407692	0.466005
23	C	5.045771	-2.780313	0.701392
24	C	3.863292	-3.487324	0.637740
25	C	1.432561	0.726233	0.102163
26	C	3.888781	0.709020	-0.179235
27	C	0.343697	1.458652	0.578690
28	C	0.258019	2.856881	0.238914
29	C	1.388720	3.534505	-0.295981
30	C	2.654240	2.828495	-0.375263
31	C	2.659887	1.425242	-0.178391
32	C	3.870295	3.479735	-0.637646
33	C	5.051347	2.770348	-0.701379
34	C	5.062321	1.397683	-0.466104
35	C	-0.989406	3.542111	0.342284
36	C	-1.056897	4.872237	-0.060296
37	C	0.052247	5.529532	-0.582419
38	C	1.256123	4.869372	-0.706197
39	C	-0.735596	0.891092	1.397201
40	C	-2.172690	2.837303	0.858952
41	C	-1.993994	1.558441	1.459255
42	C	-3.080910	0.951299	2.160601
43	C	-4.352068	1.571906	2.133317
44	C	-4.529295	2.756583	1.467542
45	C	-3.441920	3.394322	0.850230
46	C	-0.550014	-0.232821	2.191672
47	C	-1.599592	-0.793455	2.929672
48	C	-2.855613	-0.240557	2.883956
49	H	-5.512814	-3.213213	-1.435211
50	H	-5.180604	-1.086841	-2.649972
51	H	-3.624486	-4.339714	-0.368889
52	H	-3.679490	0.699944	-3.422317
53	H	-1.410042	1.680831	-3.526793
54	H	0.425520	0.697293	-2.237783
55	H	2.089040	-5.392452	1.139494
56	H	-0.047524	-6.562377	0.897791
57	H	-1.996146	-5.414284	-0.030485
58	H	6.003759	-0.882027	0.505268
59	H	5.973715	-3.297130	0.915150
60	H	3.884251	-4.559866	0.772429
61	H	3.893417	4.552249	-0.772211
62	H	5.980330	3.285319	-0.915079
63	H	6.005521	0.870129	-0.505379
64	H	-1.985190	5.418480	0.031186
65	H	-0.034288	6.562767	-0.897002
66	H	2.099882	5.388542	-1.139023
67	H	-5.178386	1.096928	2.649820
68	H	-5.506274	3.224297	1.435631
69	H	-3.615679	4.347200	0.369528
70	H	0.424039	-0.698654	2.236886
71	H	-1.413524	-1.678817	3.525643
72	H	-3.680932	-0.693184	3.421578

```
----------------------------------------------------------------
B3LYP/6-311g(2d,p)
E(RB3LYP) = -1844.13211909 hartree
# of imaginary frequencies = 0

<Thermal energies>
-----------------------------------------------------------------
 Zero-point correction=                      0.557188 (Hartree/Particle)
 Thermal correction to Energy=                    0.587914
 Thermal correction to Enthalpy=                  0.588858
 Thermal correction to Gibbs Free Energy=         0.497866
 Sum of electronic and zero-point Energies=          -1843.574931
 Sum of electronic and thermal Energies=             -1843.544205
 Sum of electronic and thermal Enthalpies=           -1843.543261
 Sum of electronic and thermal Free Energies=        -1843.634253
-----------------------------------------------------------------

<Transition moments>
-----------------------------------------------------------------
 Ground to excited state transition electric dipole moments (Au):
```

state	X	Y	Z	Dip. S.	Osc.
1	-0.0013	-1.2720	-1.1434	2.9253	0.1182
2	0.2140	-0.0003	0.0000	0.0458	0.0025
3	-0.0001	-0.4503	0.1894	0.2387	0.0145
4	-2.4640	0.0022	-0.0002	6.0711	0.3946
5	-0.0013	-1.0044	0.2838	1.0893	0.0745
6	0.1316	0.0000	-0.0001	0.0173	0.0014
7	-0.0007	-0.8179	0.6134	1.0451	0.0835
8	-0.0684	0.0002	-0.0001	0.0047	0.0004
9	0.2442	0.0003	-0.0008	0.0596	0.0050
10	0.0013	-0.0376	0.1341	0.0194	0.0016

Ground to excited state transition magnetic dipole moments (Au):

state	X	Y	Z
1	0.0003	0.0046	2.1085
2	0.1659	-0.0002	0.0001
3	0.0001	-0.0123	0.1067
4	-0.8671	0.0008	-0.0001
5	0.0000	0.0919	0.2870
6	0.2903	-0.0003	0.0002
7	0.0002	0.0986	-0.4907
8	0.3182	-0.0004	0.0002
9	-0.0283	-0.0010	0.0010
10	0.0002	0.2022	-0.1219

Rotatory Strengths (R) in cgs (10**-40 erg-esu-cm/Gauss)

state	XX	YY	ZZ	R(velocity)	E-M Angle
1	954.1594	974.2737	-166.6635	587.2565	47.06
2	0.0000	-15.4485	-11.2972	-8.9152	90.00
3	-39.3509	18.9233	1.4563	-6.3238	119.03
4	-0.0008	-941.8646	-593.4382	-511.7679	90.00
5	-59.9426	33.1722	35.6645	2.9647	87.75
6	0.0000	-8.2016	-16.5547	-8.2521	90.00
7	-123.6030	266.0041	134.9316	92.4442	41.47
8	0.0000	8.7226	5.6893	4.8040	90.00
9	0.0002	7.5361	-2.5942	1.6474	90.00
10	3.7658	9.3226	6.8220	6.6368	35.65

Rotatory Strengths (R) in cgs (10**-40 erg-esu-cm/Gauss)

state	XX	YY	ZZ	R(length)
1	0.0002	4.1516	1704.8209	569.6576
2	-25.1086	0.0000	0.0000	-8.3696
3	0.0000	-3.9291	-14.2940	-6.0744
4	-1510.9129	-0.0013	0.0000	-503.6381
5	0.0000	65.3055	-57.5833	2.5741
6	-27.0098	0.0000	0.0000	-9.0033
7	0.0001	57.0192	212.8483	89.9559
8	15.3836	0.0001	0.0000	5.1279
9	4.8894	0.0002	0.0006	1.6300
10	-0.0002	5.3808	11.5611	5.6473

<Summary of chiroptical properties>

state	|TEDM| (10-20 esu-cm)	|TMDM| (10-20 erg/Gauss)	cos(theta) (E-M Angle)	g (4R/D)	Eex (nm)
1	434.73	1.955	0.670	0.0120	751.96
2	54.39	0.154	-1.000	-0.0113	551.04
3	124.17	0.100	-0.490	-0.0016	499.98
4	626.29	0.804	-1.000	-0.0051	467.37
5	265.29	0.279	0.034	0.0001	443.97
6	33.45	0.269	-1.000	-0.0322	387.22
7	259.86	0.464	0.746	0.0053	380.15
8	17.39	0.295	1.000	0.0678	365.08
9	62.07	0.026	0.999	0.0017	363.99
10	35.40	0.219	0.728	0.0180	363.77

Supplementary Table 12: Cartesian coordinates (Å) of the optimized structure of compound **3** in the ground state (S_0) calculated at the B3LYP/6-311G(2d,p) level of theory.

1	C	-3.853885	-3.456848	-2.277363	47	C	-0.744606	-1.683766	1.914169
2	C	-4.346769	-2.190521	-2.458109	48	C	-2.152437	-1.879831	1.885852
3	C	-3.476066	-1.075776	-2.435507	49	C	0.119705	-2.804661	2.093355
4	C	-2.084524	-1.283866	-2.186143	50	C	-0.444568	-4.065365	2.254569
5	C	-1.576948	-2.611653	-2.105498	51	C	-1.822756	-4.252518	2.218263
6	C	-2.475058	-3.667245	-2.122063	52	C	-2.664821	-3.178027	2.029721
7	C	-3.944668	0.233670	-2.681675	53	C	1.577442	-2.611355	2.105484
8	C	-3.064953	1.287333	-2.706130	54	C	1.215559	-0.160053	2.058243
9	C	-1.713642	1.091662	-2.395243	55	C	1.713449	1.091987	2.395213
10	C	-1.215523	-0.160284	-2.058260	56	C	3.064728	1.287911	2.706080
11	C	-0.119175	-2.804686	-2.093350	57	C	3.944639	0.234412	2.681617
12	C	0.190145	-0.373243	-1.682620	58	C	3.476278	-1.075121	2.435461
13	C	0.744924	-1.683630	-1.914156	59	C	2.084771	-1.283473	2.186117
14	C	2.152791	-1.879430	-1.885822	60	C	4.347189	-2.189704	2.458055
15	C	2.665420	-3.177531	-2.029680	61	C	3.854540	-3.456123	2.277321
16	C	1.823559	-4.252180	-2.218229	62	C	2.475750	-3.666779	2.122041
17	C	0.445337	-4.065286	-2.254553	63	H	-4.523217	-4.309149	-2.282221
18	C	1.020306	0.620575	-1.156885	64	H	-5.404934	-2.025648	-2.626395
19	C	2.438715	0.518957	-1.383385	65	H	-2.121434	-4.684115	-2.022305
20	C	3.016969	-0.722608	-1.747584	66	H	-4.999469	0.386332	-2.879383
21	C	3.244945	1.689219	-1.321278	67	H	-3.412657	2.284244	-2.949008
22	C	4.614747	1.568444	-1.529391	68	H	-1.049921	1.944078	-2.407711
23	C	5.189395	0.339409	-1.842092	69	H	3.731734	-3.346446	-1.978241
24	C	4.401981	-0.785250	-1.969318	70	H	2.234031	-5.248114	-2.335660
25	C	0.536855	1.808375	-0.446425	71	H	-0.189399	-4.925274	-2.416088
26	C	2.604932	2.993100	-1.096170	72	H	5.254337	2.437093	-1.454938
27	C	-0.537205	1.808275	0.446429	73	H	6.258241	0.273019	-2.006875
28	C	-1.260786	3.037480	0.631693	74	H	4.862112	-1.717958	-2.263953
29	C	-0.677378	4.278600	0.261487	75	H	1.002672	6.409134	-0.141462
30	C	0.676560	4.278727	-0.261494	76	H	3.260624	6.329515	-1.081881
31	C	1.260203	3.037716	-0.631691	77	H	4.300993	4.188743	-1.651578
32	C	1.422270	5.455252	-0.430032	78	H	-4.301789	4.187937	1.651586
33	C	2.701536	5.411553	-0.944836	79	H	-3.261829	6.328903	1.081869
34	C	3.290376	4.191798	-1.267233	80	H	-1.003895	6.408944	0.141444
35	C	-2.605504	2.992612	1.096179	81	H	-4.861783	-1.718866	2.264012
36	C	-3.291174	4.191182	1.267236	82	H	-6.258290	0.271847	2.006939
37	C	-2.702567	5.411046	0.944829	83	H	-5.254801	2.436106	1.454979
38	C	-1.423311	5.454985	0.430020	84	H	0.190330	-4.925234	2.416098
39	C	-1.020428	0.620385	1.156895	85	H	-2.233040	-5.248528	2.335702
40	C	-3.245268	1.688612	1.321302	86	H	-3.731103	-3.347142	1.978295
41	C	-2.438817	0.518501	1.383409	87	H	1.049569	1.944279	2.407687
42	C	-3.016834	-0.723171	1.747619	88	H	3.412249	2.284888	2.948949
43	C	-4.401831	-0.786073	1.969368	89	H	4.999413	0.387272	2.879310
44	C	-5.189459	0.338437	1.842144	90	H	5.405325	-2.024633	2.626326
45	C	-4.615046	1.567579	1.529431	91	H	4.524030	-4.308299	2.282173
46	C	-0.190075	-0.373276	1.682622	92	H	2.122314	-4.683716	2.022291

```
-----------------------------------------------------------------
B3LYP/6-311g(2d,p)
E(RB3LYP) = -2381.39819381 hartree
# of imaginary frequencies = 0

<Thermal energies>
------------------------------------------------------------------
 Zero-point correction=                      0.708944 (Hartree/Particle)
 Thermal correction to Energy=                   0.748702
 Thermal correction to Enthalpy=                 0.749646
 Thermal correction to Gibbs Free Energy=        0.640123
 Sum of electronic and zero-point Energies=         -2380.689250
 Sum of electronic and thermal Energies=            -2380.649492
 Sum of electronic and thermal Enthalpies=          -2380.648548
 Sum of electronic and thermal Free Energies=       -2380.758071
------------------------------------------------------------------
```

<Transition moments>

Ground to excited state transition electric dipole moments (Au):

state	X	Y	Z	Dip. S.	Osc.
1	0.4710	0.0000	1.5573	2.6469	0.0894
2	0.0000	-0.4469	0.0000	0.1997	0.0092
3	0.0001	-1.3936	0.0000	1.9422	0.1069
4	0.0000	-0.4712	0.0000	0.2220	0.0127
5	0.3025	0.0000	-0.0253	0.0921	0.0053
6	-0.8424	-0.0001	0.5274	0.9878	0.0617
7	-0.0001	0.8138	0.0000	0.6623	0.0440
8	2.1673	0.0002	0.1580	4.7220	0.3318
9	0.0000	0.2474	0.0000	0.0612	0.0045
10	0.0000	-0.3048	0.0000	0.0929	0.0070

Ground to excited state transition magnetic dipole moments (Au):

state	X	Y	Z
1	-0.7973	-0.0001	-1.9044
2	0.0000	-0.4455	0.0000
3	0.0001	-1.2009	0.0000
4	0.0000	0.0057	0.0000
5	0.1971	0.0000	0.0008
6	-0.4207	0.0000	-0.5884
7	0.0000	0.0051	0.0000
8	0.3281	0.0000	-0.4770
9	0.0000	-0.0965	0.0000
10	0.0000	0.2417	0.0000

Rotatory Strengths (R) in cgs (10**-40 erg-esu-cm/Gauss)

state	XX	YY	ZZ	R(velocity)	E-M Angle
1	1001.6220	1266.2951	166.8150	811.5774	5.87
2	-24.8086	0.0000	-116.2722	-47.0269	90.00
3	-105.8109	0.0000	-1082.2348	-396.0153	90.00
4	-33.6476	0.0000	35.6562	0.6695	90.00
5	1.4492	-17.7371	-28.8143	-15.0341	175.23
6	133.3113	-6.4251	-160.3532	-11.1557	93.69
7	-63.8668	0.0000	60.8498	-1.0057	90.00
8	10.6850	-216.9874	-251.3848	-152.5624	120.34
9	-1.4149	0.0000	19.0796	5.8882	90.00
10	2.1614	0.0000	49.7875	17.3163	90.00

Rotatory Strengths (R) in cgs (10**-40 erg-esu-cm/Gauss)

state	XX	YY	ZZ	R(length)
1	265.5876	0.0000	2097.1928	787.5935
2	0.0000	-140.8160	0.0000	-46.9387
3	0.0000	-1183.4793	0.0000	-394.4931
4	0.0000	1.8999	0.0000	0.6333
5	-42.1690	0.0000	0.0138	-14.0517
6	-250.6247	0.0000	219.4406	-10.3947
7	0.0000	-2.9571	0.0000	-0.9857
8	-502.8350	0.0000	53.2997	-149.8451
9	0.0000	16.8797	0.0000	5.6266
10	0.0000	52.0914	0.0000	17.3638

<Summary of chiroptical properties>

state	|TEDM| (10-20 esu-cm)	|TMDM| (10-20 erg/Gauss)	cos(theta) (E-M Angle)	g (4R/D)	Eex (nm)
1	413.53	1.915	0.995	0.0184	899.10
2	113.59	0.413	-1.000	-0.0145	658.33
3	354.22	1.114	-1.000	-0.0126	551.91
4	119.77	0.005	0.998	0.0002	531.32
5	77.16	0.183	-0.996	-0.0094	525.50
6	252.62	0.671	-0.061	-0.0007	486.00
7	206.85	0.005	-1.000	-0.0001	456.69
8	552.33	0.537	-0.505	-0.0020	432.25
9	62.88	0.089	1.000	0.0057	410.39
10	77.47	0.224	1.000	0.0116	402.82

Supplementary Table 13: Cartesian coordinates (Å) of the optimized structure of compound **3** in the local minimum state ((*P*)-**3-1**) calculated at the B3LYP/6-311G(2d,p) level of theory.

1	C	5.345540	-2.208802	-1.606062
2	C	4.454276	-2.553407	-2.587858
3	C	3.063113	-2.470109	-2.350483
4	C	2.590702	-2.003244	-1.084193
5	C	3.533826	-1.694683	-0.060470
6	C	4.885939	-1.797190	-0.347473
7	C	2.134804	-2.865844	-3.339456
8	C	0.789698	-2.811039	-3.084159
9	C	0.321299	-2.336197	-1.853238
10	C	1.183245	-1.899666	-0.853583
11	C	3.043413	-1.398991	1.293975
12	C	0.693262	-1.535688	0.481941
13	C	1.653254	-1.493998	1.568936
14	C	1.209347	-1.468909	2.919666
15	C	2.117173	-1.159383	3.938187
16	C	3.447146	-0.932468	3.642800
17	C	3.908595	-1.081790	2.340108
18	C	-0.657048	-1.413724	0.804912
19	C	-1.049085	-1.933277	2.093841
20	C	-0.153380	-1.892597	3.182861
21	C	-2.281789	-2.623426	2.208310
22	C	-2.664297	-3.112241	3.451935
23	C	-1.835983	-2.952642	4.562276
24	C	-0.587422	-2.374429	4.427181
25	C	-1.784623	-0.970051	-0.034298
26	C	-3.060269	-2.838685	0.984303
27	C	-2.002907	0.300636	-0.581979
28	C	-3.250741	0.504257	-1.300946
29	C	-3.979166	-0.594644	-1.821170
30	C	-3.837731	-1.858681	-1.118254
31	C	-2.888965	-1.909051	-0.071536
32	C	-4.746078	-2.921158	-1.250799
33	C	-4.797746	-3.923646	-0.300142
34	C	-4.002037	-3.851933	0.842833
35	C	-3.788833	1.804804	-1.458851
36	C	-4.793196	2.015570	-2.398265
37	C	-5.326732	0.944058	-3.112714
38	C	-4.966249	-0.350895	-2.788794
39	C	-1.249660	1.528350	-0.257782
40	C	-3.344778	2.819618	-0.502806
41	C	-2.086469	2.646640	0.125609
42	C	-1.607597	3.628422	1.022082
43	C	-2.435647	4.710971	1.351314
44	C	-3.680632	4.851361	0.767956
45	C	-4.124561	3.922212	-0.169643
46	C	0.125301	1.787864	-0.267585
47	C	0.644752	2.725238	0.707446
48	C	-0.239847	3.513295	1.488582
49	C	2.044854	2.817851	0.936657
50	C	2.488368	3.520400	2.056177
51	C	1.600654	4.186262	2.893746
52	C	0.252598	4.217115	2.592806
53	C	2.974257	2.251774	-0.051237
54	C	1.035914	1.356005	-1.328288
55	C	0.553349	0.873255	-2.538121
56	C	1.403766	0.608453	-3.618616
57	C	2.754532	0.809514	-3.505640
58	C	3.303191	1.315322	-2.304926
59	C	2.435396	1.628155	-1.213936
60	C	4.692655	1.537512	-2.173239
61	C	5.203301	2.062405	-1.014358
62	C	4.347179	2.431436	0.032385
63	H	6.411974	-2.276551	-1.786107
64	H	4.800145	-2.905750	-3.553080
65	H	5.620506	-1.587802	0.417071
66	H	2.506159	-3.230449	-4.290601
67	H	0.075902	-3.144466	-3.828191
68	H	-0.742701	-2.345871	-1.674705
69	H	1.773995	-1.086713	4.961833
70	H	4.141050	-0.667004	4.431477
71	H	4.962935	-0.943036	2.147231
72	H	-3.613075	-3.621927	3.562986
73	H	-2.155451	-3.325384	5.528059
74	H	0.078277	-2.344093	5.279790
75	H	-5.458407	-2.926882	-2.065128
76	H	-5.506570	-4.735436	-0.411416
77	H	-4.120385	-4.592332	1.623735
78	H	-5.171328	3.015279	-2.569735
79	H	-6.081870	1.122298	-3.868921
80	H	-5.484113	-1.179544	-3.253688
81	H	-2.078257	5.471341	2.033385
82	H	-4.305741	5.698310	1.024122
83	H	-5.096626	4.051000	-0.628184
84	H	3.543999	3.564603	2.284540
85	H	1.974787	4.709691	3.765569
86	H	-0.426979	4.777100	3.221947
87	H	-0.510672	0.731372	-2.665912
88	H	0.981979	0.240459	-4.545744
89	H	3.418947	0.593290	-4.334159
90	H	5.343264	1.288420	-3.003422
91	H	6.269584	2.226964	-0.911956
92	H	4.781286	2.905490	0.901659

```
--------------------------------------------------------------------
B3LYP/6-311g(2d,p)
E(RB3LYP) = -2381.34906281 hartree
# of imaginary frequencies = 0


<Thermal energies>
---------------------------------------------------------------------
 Zero-point correction=                      0.708061 (Hartree/Particle)
 Thermal correction to Energy=                   0.747672
 Thermal correction to Enthalpy=                 0.748616
 Thermal correction to Gibbs Free Energy=        0.639930
 Sum of electronic and zero-point Energies=          -2380.641002
 Sum of electronic and thermal Energies=             -2380.601391
 Sum of electronic and thermal Enthalpies=           -2380.600447
 Sum of electronic and thermal Free Energies=        -2380.709132
```

Supplementary Table 14: Cartesian coordinates (Å) of the optimized structure of compound **3** in the transition state ((*P*)-**3_TS1** and **3_TS2**) calculated at the B3LYP/6-311G(2d,p) level of theory.

(*P*)-**3_TS1**

1	C	5.690433	-0.480983	-0.656554
2	C	5.066085	0.141542	0.403531
3	C	4.999510	-1.412588	-1.421156
4	C	3.788950	1.217226	2.839288
5	C	3.744714	-0.172479	0.748349
6	C	3.671763	-1.730107	-1.149788
7	C	3.103674	4.323990	-0.780571
8	C	3.172822	1.651171	3.994641
9	C	3.045284	2.121558	-1.919554
10	C	3.420437	-3.096129	-3.239429
11	C	3.105225	0.409409	1.913286
12	C	3.029931	-1.128533	-0.026542
13	C	2.419824	5.357488	-0.189326
14	C	2.922274	-2.639843	-2.027328
15	C	2.420014	3.150372	-1.178864
16	C	2.737704	-4.058617	-3.998004
17	C	2.331450	1.012285	-2.300980
18	C	1.888120	1.213119	4.309030
19	C	1.747606	0.104953	2.157694
20	C	1.641803	-1.418903	0.259705
21	C	1.021179	5.308395	-0.074059
22	C	1.657938	-3.117774	-1.584719
23	C	1.029950	3.034876	-0.892145
24	C	1.181755	0.406980	3.426153
25	C	1.571730	-4.608111	-3.531161
26	C	1.012167	0.824672	-1.863145
27	C	0.962384	-0.614814	1.180528
28	C	1.053264	-2.596184	-0.400578
29	C	0.311038	4.186014	-0.470396
30	C	1.014863	-4.170712	-2.306833
31	C	0.355291	1.797508	-1.124939
32	C	-0.043931	-0.301522	3.801201
33	C	-0.017596	-3.300303	0.134730
34	C	-0.424246	-0.488970	5.126517
35	C	-0.494088	-0.632534	1.391926
36	C	-0.141293	-4.774297	-1.764356
37	C	-1.137518	4.170203	-0.698539
38	C	-0.850284	-0.859717	2.775788
39	C	-1.073386	1.687773	-0.840600
40	C	-0.611439	-4.375701	-0.538777
41	C	-1.885906	5.342981	-0.749486
42	C	-1.784686	2.935556	-0.994626
43	C	-1.562832	-1.222930	5.453028
44	C	-1.573816	-0.508751	0.479471
45	C	-1.830825	0.526231	-0.548915
46	C	-2.004430	-1.595031	3.110897
47	C	-2.343537	-1.778354	4.457358
48	C	-3.225589	5.332140	-1.127496
49	C	-2.690455	-1.410613	0.772234
50	C	-3.128768	2.940784	-1.423749
51	C	-2.788417	-2.102079	2.008724
52	C	-3.153514	0.507062	-1.172768
53	C	-3.837188	4.146463	-1.486273
54	C	-3.708400	1.664904	-1.773467
55	C	-3.660441	-1.701932	-0.222052
56	C	-3.657069	-3.191915	2.144054
57	C	-3.901752	-0.694844	-1.249372
58	C	-4.809434	1.553930	-2.631696
59	C	-4.453470	-3.585501	1.086793
60	C	-4.477427	-2.821822	-0.074070
61	C	-4.965102	-0.783707	-2.145971
62	C	-5.392809	0.324793	-2.866796
63	H	3.156458	-4.385605	-4.942362
64	H	1.065874	-5.389511	-4.086814
65	H	4.360103	-2.719462	-3.619380
66	H	-0.623112	-5.576296	-2.311891
67	H	-1.459647	-4.874319	-0.085977
68	H	-0.412378	-3.022270	1.098532
69	H	5.614892	0.875596	0.975914
70	H	6.721219	-0.245935	-0.894276
71	H	5.512992	-1.893080	-2.242048
72	H	1.451163	1.487120	5.260506
73	H	3.708984	2.290458	4.685705
74	H	4.819020	1.493271	2.664243
75	H	-3.240997	-2.325098	4.716941
76	H	-1.836122	-1.354839	6.493026
77	H	0.183610	-0.079982	5.922800
78	H	-5.180829	-3.082042	-0.852542
79	H	-5.095924	-4.453118	1.177926
80	H	-3.683552	-3.738975	3.077587
81	H	-5.185044	2.436928	-3.132182
82	H	-6.214532	0.232768	-3.566656
83	H	-5.503449	-1.714801	-2.255849
84	H	-1.413591	6.290787	-0.528720
85	H	-3.783057	6.260733	-1.155754
86	H	-4.877797	4.144016	-1.783754
87	H	0.482970	-0.079292	-2.135283
88	H	2.793027	0.256340	-2.921068
89	H	4.085294	2.231817	-2.202868
90	H	4.169992	4.397897	-0.961624
91	H	2.948835	6.245975	0.134623
92	H	0.498389	6.182446	0.292179

```
---------------------------------------------------------------------
B3LYP/6-311g(2d,p)
E(RB3LYP) = -2381.33147842 hartree
# of imaginary frequencies = 1

<Thermal energies>
---------------------------------------------------------------------
 Zero-point correction=                       0.708042 (Hartree/Particle)
 Thermal correction to Energy=                     0.746689
 Thermal correction to Enthalpy=                   0.747633
 Thermal correction to Gibbs Free Energy=          0.642283
 Sum of electronic and zero-point Energies=            -2380.623436
 Sum of electronic and thermal Energies=               -2380.584790
 Sum of electronic and thermal Enthalpies=             -2380.583845
 Sum of electronic and thermal Free Energies=          -2380.689195
```

3_TS2

1	C	-1.294598	-5.346029	2.003856
2	C	-2.460059	-4.628140	1.939790
3	C	-2.426430	-3.218733	1.839246
4	C	-1.165962	-2.547046	1.786468
5	C	0.033338	-3.305706	1.919558
6	C	-0.058175	-4.686861	2.007906
7	C	-3.620024	-2.462544	1.813690
8	C	-3.570038	-1.095286	1.736429
9	C	-2.339904	-0.434082	1.648593
10	C	-1.133070	-1.123995	1.646411
11	C	1.305624	-2.591999	2.097714
12	C	0.149483	-0.420416	1.716361
13	C	1.302611	-1.172929	2.169215
14	C	2.465907	-0.498780	2.627870
15	C	3.653327	-1.215679	2.807180
16	C	3.683045	-2.580596	2.593884
17	C	2.514950	-3.263258	2.276086
18	C	0.284010	0.964767	1.587871
19	C	1.188956	1.591960	2.524737
20	C	2.318573	0.894234	3.005625
21	C	0.855389	2.859747	3.062313
22	C	1.719770	3.455542	3.974331
23	C	2.890298	2.811017	4.368653
24	C	3.178716	1.540772	3.904735
25	C	-0.413086	1.912999	0.701355
26	C	-0.403006	3.476348	2.638229
27	C	-0.413086	1.912999	-0.701355
28	C	-0.903134	3.109044	-1.364780
29	C	-1.841776	3.955263	-0.726945
30	C	-1.841776	3.955263	0.726945
31	C	-0.903134	3.109044	1.364780
32	C	-2.512027	4.916212	1.499933
33	C	-2.153662	5.132633	2.817915
34	C	-1.058506	4.465648	3.364138
35	C	-0.403006	3.476348	-2.638229
36	C	-1.058506	4.465648	-3.364138
37	C	-2.153662	5.132633	-2.817915
38	C	-2.512027	4.916212	-1.499933
39	C	0.284010	0.964767	-1.587871
40	C	0.855389	2.859747	-3.062313
41	C	1.188956	1.591960	-2.524737
42	C	2.318573	0.894234	-3.005625
43	C	3.178716	1.540772	-3.904735
44	C	2.890298	2.811017	-4.368653
45	C	1.719770	3.455542	-3.974331
46	C	0.149483	-0.420416	-1.716361
47	C	1.302611	-1.172929	-2.169215
48	C	2.465907	-0.498780	-2.627870
49	C	1.305624	-2.591999	-2.097714
50	C	2.514950	-3.263258	-2.276086
51	C	3.683045	-2.580596	-2.593884
52	C	3.653327	-1.215679	-2.807180
53	C	0.033338	-3.305706	-1.919558
54	C	-1.133070	-1.123995	-1.646411
55	C	-2.339904	-0.434082	-1.648593
56	C	-3.570038	-1.095286	-1.736429
57	C	-3.620024	-2.462544	-1.813690
58	C	-2.426430	-3.218733	-1.839246
59	C	-1.165962	-2.547046	-1.786468
60	C	-2.460059	-4.628140	-1.939790
61	C	-1.294598	-5.346029	-2.003856
62	C	-0.058175	-4.686861	-2.007906
63	H	-1.321075	-6.426729	2.078402
64	H	-3.421632	-5.127501	1.973514
65	H	0.835609	-5.284244	2.121297
66	H	-4.570064	-2.981270	1.873314
67	H	-4.483842	-0.513084	1.744047
68	H	-2.335686	0.645598	1.619443
69	H	4.555799	-0.697974	3.104923
70	H	4.611150	-3.127962	2.707554
71	H	2.558001	-4.338400	2.172935
72	H	1.488232	4.434158	4.375296
73	H	3.560429	3.294733	5.069099
74	H	4.051185	1.022991	4.281268
75	H	-3.275999	5.536490	1.050305
76	H	-2.679666	5.875864	3.405045
77	H	-0.715673	4.722693	4.358263
78	H	-0.715673	4.722693	-4.358263
79	H	-2.679666	5.875864	-3.405045
80	H	-3.275999	5.536490	-1.050305
81	H	4.051185	1.022991	-4.281268
82	H	3.560429	3.294733	-5.069099
83	H	1.488232	4.434158	-4.375296
84	H	2.558001	-4.338400	-2.172935
85	H	4.611150	-3.127962	-2.707554
86	H	4.555799	-0.697974	-3.104923
87	H	-2.335686	0.645598	-1.619443
88	H	-4.483842	-0.513084	-1.744047
89	H	-4.570064	-2.981270	-1.873314
90	H	-3.421632	-5.127501	-1.973514
91	H	-1.321075	-6.426729	-2.078402
92	H	0.835609	-5.284244	-2.121297

B3LYP/6-311g(2d,p)
E(RB3LYP) = -2381.34783321 hartree
# of imaginary frequencies = 1

<Thermal energies>

Zero-point correction= 0.707821 (Hartree/Particle)
Thermal correction to Energy= 0.746592
Thermal correction to Enthalpy= 0.747536
Thermal correction to Gibbs Free Energy= 0.641955
Sum of electronic and zero-point Energies= -2380.640012
Sum of electronic and thermal Energies= -2380.601242
Sum of electronic and thermal Enthalpies= -2380.600297
Sum of electronic and thermal Free Energies= -2380.705878

Supplementary Table 15: Cartesian coordinates (Å) of the optimized structures of **1**, **2**, and **3** in the ground state (S_0) calculated at the B3LYP-GD3BJ/6-311G(2d,p) level of theory.

Compound **1**

1	C	2.820701	1.562079	0.143155
2	C	3.508593	2.758428	0.313988
3	C	2.833779	3.973623	0.369567
4	C	1.459118	4.011994	0.261955
5	C	0.722317	2.835758	0.064607
6	C	1.410102	1.599838	-0.020801
7	C	-0.722307	2.835760	-0.064601
8	C	-1.410095	1.599842	0.020807
9	C	-0.676500	0.365593	0.163958
10	C	0.676503	0.365591	-0.163948
11	C	-1.459104	4.011998	-0.261952
12	C	-2.833764	3.973630	-0.369569
13	C	-3.508582	2.758436	-0.313993
14	C	-2.820694	1.562087	-0.143156
15	C	1.414725	-0.812382	-0.630115
16	C	3.506600	0.264461	0.156898
17	C	-3.506595	0.264470	-0.156906
18	C	-1.414728	-0.812378	0.630121
19	C	0.843173	-1.834476	-1.392903
20	C	1.595867	-3.005730	-1.634521
21	C	2.871521	-3.165336	-1.170352
22	C	3.529299	-2.092757	-0.527849
23	C	2.810907	-0.880514	-0.324597
24	C	4.876667	-2.182829	-0.115312
25	C	5.502813	-1.097238	0.441992
26	C	4.826438	0.125113	0.556174
27	C	-2.810908	-0.880507	0.324592
28	C	-3.529304	-2.092748	0.527839
29	C	-2.871534	-3.165329	1.170346
30	C	-1.595884	-3.005726	1.634528
31	C	-0.843186	-1.834473	1.392917
32	C	-4.826430	0.125126	-0.556190
33	C	-5.502809	-1.097224	-0.442014
34	C	-4.876670	-2.182817	0.115293
35	C	-0.519340	-1.773677	-2.028530
36	C	0.519318	-1.773675	2.028564
37	H	4.585684	2.753899	0.405327
38	H	3.391091	4.891299	0.511974
39	H	0.948211	4.960296	0.351013
40	H	-0.948194	4.960298	-0.351010
41	H	-3.391073	4.891308	-0.511980
42	H	-4.585672	2.753911	-0.405338
43	H	1.127323	-3.803537	-2.199671
44	H	3.407760	-4.093189	-1.331735
45	H	5.405465	-3.118501	-0.254418
46	H	6.532138	-1.168921	0.771272
47	H	5.359285	0.973817	0.962057
48	H	-3.407777	-4.093181	1.331726
49	H	-1.127348	-3.803532	2.199684
50	H	-5.359273	0.973832	-0.962074
51	H	-6.532133	-1.168903	-0.771300
52	H	-5.405471	-3.118488	0.254395
53	H	-0.887794	-0.754086	-2.118869
54	H	-0.466266	-2.212674	-3.027315
55	H	-1.261228	-2.340478	-1.462867
56	H	1.261214	-2.340478	1.462915
57	H	0.887772	-0.754083	2.118907
58	H	0.466227	-2.212669	3.027350

Compound **2**

1	C	-4.605575	2.293952	0.788170
2	C	-4.454547	1.075118	1.395849
3	C	-3.162055	0.551308	1.630999
4	C	-2.026370	1.295772	1.195322
5	C	-2.196334	2.600648	0.654260
6	C	-3.483084	3.061147	0.434736
7	C	-2.963697	-0.684454	2.281557
8	C	-1.690863	-1.145514	2.521009
9	C	-0.581446	-0.448225	2.033676
10	C	-0.731655	0.721362	1.303182
11	C	-1.000819	3.409667	0.385308
12	C	0.392035	1.384938	0.644777
13	C	0.282796	2.790160	0.382757
14	C	1.431184	3.533871	-0.002468
15	C	1.284842	4.893495	-0.307616
16	C	0.047098	5.501470	-0.247206
17	C	-1.085111	4.765291	0.087300
18	C	1.510973	0.711026	0.164509
19	C	2.733672	1.439372	-0.021206
20	C	2.713570	2.855785	-0.071767
21	C	3.964687	0.730938	-0.083533
22	C	5.137820	1.458138	-0.249909
23	C	5.118473	2.849111	-0.316363
24	C	3.928823	3.540697	-0.215027
25	C	1.510967	-0.711034	-0.164504
26	C	3.964683	-0.730965	0.083533
27	C	0.392022	-1.384938	-0.644772
28	C	0.282776	-2.790161	-0.382760
29	C	1.431160	-3.533879	0.002464
30	C	2.713550	-2.855802	0.071763
31	C	2.733662	-1.439389	0.021206
32	C	3.928798	-3.540724	0.215012
33	C	5.118454	-2.849146	0.316348
34	C	5.137811	-1.458175	0.249903
35	C	-1.000842	-3.409663	-0.385318
36	C	-1.085142	-4.765287	-0.087307
37	C	0.047063	-5.501471	0.247203
38	C	1.284810	-4.893502	0.307613
39	C	-0.731668	-0.721350	-1.303169
40	C	-2.196354	-2.600643	-0.654279
41	C	-2.026384	-1.295761	-1.195323
42	C	-3.162068	-0.551288	-1.630989
43	C	-4.454562	-1.075099	-1.395854
44	C	-4.605595	-2.293941	-0.788194
45	C	-3.483106	-3.061143	-0.434766
46	C	-0.581456	0.448247	-2.033645
47	C	-1.690872	1.145551	-2.520960
48	C	-2.963706	0.684488	-2.281520
49	H	-5.596487	2.686224	0.593776
50	H	-5.317354	0.495029	1.699791
51	H	-3.643208	4.030749	-0.016297
52	H	-3.827905	-1.252834	2.602369
53	H	-1.539237	-2.073679	3.057550
54	H	0.408436	-0.853690	2.188993
55	H	2.143236	5.473719	-0.615557
56	H	-0.048078	6.555256	-0.478709
57	H	-2.043631	5.264512	0.112972
58	H	6.087470	0.947424	-0.325284
59	H	6.048492	3.391226	-0.435470
60	H	3.943127	4.621429	-0.232205
61	H	3.943094	-4.621456	0.232179
62	H	6.048470	-3.391269	0.435445
63	H	6.087466	-0.947468	0.325274
64	H	-2.043663	-5.264504	-0.112981
65	H	-0.048120	-6.555257	0.478706
66	H	2.143201	-5.473731	0.615555
67	H	-5.317366	-0.495002	-1.699786
68	H	-5.596508	-2.686213	-0.593806
69	H	-3.643233	-4.030750	0.016256
70	H	0.408428	0.853711	-2.188955
71	H	-1.539243	2.073722	-3.057490
72	H	-3.827912	1.252873	-2.602328

Compound **3**

1	C	-3.619641	3.522087	1.635218
2	C	-4.148544	2.279920	1.868107
3	C	-3.298153	1.159356	2.013862
4	C	-1.891121	1.336744	1.863955
5	C	-1.353947	2.645676	1.710714
6	C	-2.229002	3.707527	1.578458
7	C	-3.800023	-0.123639	2.316780
8	C	-2.936894	-1.174119	2.514639
9	C	-1.563224	-1.010976	2.308352
10	C	-1.035962	0.202627	1.892278
11	C	0.104082	2.814412	1.752925
12	C	0.371419	0.364225	1.514364
13	C	0.949718	1.673326	1.638560
14	C	2.357195	1.843518	1.553487
15	C	2.891341	3.135230	1.671195
16	C	2.069685	4.226352	1.845533
17	C	0.687062	4.069052	1.869360
18	C	1.149309	-0.667823	1.000619
19	C	2.580059	-0.566395	1.072577
20	C	3.194948	0.678910	1.353908
21	C	3.374256	-1.734183	0.912314
22	C	4.756981	-1.608104	0.931862
23	C	5.363049	-0.374980	1.161686
24	C	4.596692	0.745991	1.391905
25	C	0.582372	-1.868184	0.381647
26	C	2.716652	-3.038953	0.764860
27	C	-0.582188	-1.868237	-0.381636
28	C	-1.323778	-3.090183	-0.478498
29	C	-0.703048	-4.330821	-0.174648
30	C	0.703457	-4.330757	0.174681
31	C	1.324075	-3.090061	0.478513
32	C	1.469342	-5.503362	0.234494

33	C	2.804540	-5.454567	0.578281
34	C	3.424398	-4.233727	0.832473
35	C	-2.716361	-3.039205	-0.764838
36	C	-3.424002	-4.234045	-0.832402
37	C	-2.804035	-5.454824	-0.578192
38	C	-1.468828	-5.503495	-0.234425
39	C	-1.149241	-0.667923	-1.000587
40	C	-3.374087	-1.734496	-0.912310
41	C	-2.580001	-0.566628	-1.072538
42	C	-3.195003	0.678627	-1.353838
43	C	-4.596751	0.745575	-1.391875
44	C	-5.363006	-0.375478	-1.161708
45	C	-4.756825	-1.608547	-0.931894
46	C	-0.371454	0.364199	-1.514343
47	C	-0.949868	1.673254	-1.638491
48	C	-2.357356	1.843322	-1.553367
49	C	-0.104331	2.814415	-1.752861
50	C	-0.687426	4.069007	-1.869227
51	C	-2.070061	4.226190	-1.845321
52	C	-2.891618	3.134993	-1.670990
53	C	1.353715	2.645797	-1.710744
54	C	1.035921	0.202723	-1.892327
55	C	1.563259	-1.010835	-2.308436
56	C	2.936931	-1.173863	-2.514800
57	C	3.799983	-0.123310	-2.316994
58	C	3.298025	1.159641	-2.014040
59	C	1.890988	1.336911	-1.864043
60	C	4.148334	2.280272	-1.868320
61	C	3.619344	3.522390	-1.635374
62	C	2.228693	3.707715	-1.578523
63	H	-4.272315	4.377603	1.510278
64	H	-5.219543	2.137549	1.948770
65	H	-1.849417	4.705150	1.411248
66	H	-4.870015	-0.259122	2.415204
67	H	-3.317360	-2.147324	2.798289
68	H	-0.908487	-1.861214	2.431551
69	H	3.957555	3.287638	1.598755
70	H	2.498902	5.216433	1.939563
71	H	0.063360	4.943921	1.986889
72	H	5.383186	-2.473838	0.769015
73	H	6.443733	-0.303233	1.175085
74	H	5.088386	1.683705	1.604605
75	H	1.019622	-6.455852	-0.008638
76	H	3.382206	-6.369205	0.631270
77	H	4.476503	-4.226432	1.079827
78	H	-4.476112	-4.226854	-1.079734
79	H	-3.381623	-6.369514	-0.631148
80	H	-1.019019	-6.455938	0.008729
81	H	-5.088527	1.683244	-1.604585
82	H	-6.443697	-0.303841	-1.175151
83	H	-5.382959	-2.474338	-0.769088
84	H	-0.063810	4.943938	-1.986750
85	H	-2.499363	5.216241	-1.939285
86	H	-3.957840	3.287306	-1.598481
87	H	0.908581	-1.861125	-2.431595
88	H	3.317461	-2.147034	-2.798480
89	H	4.869980	-0.258703	-2.415481
90	H	5.219340	2.137989	-1.949041
91	H	4.271955	4.377958	-1.510454
92	H	1.849042	4.705306	-1.411274

Supplementary Table 16: Cartesian coordinates (Å) of the ground state equilibrium structures and the MECI structures of π-extended helicenes **1−3** calculated at the SF-ωB97xd/6-31G(d) level of theory. Relative energies in eV and the <S^2> values for each state are also shown.

π-extended [5]helicene **1**
S_0 minimum 0.00 [0.17] 2.08 [2.19] 2.72 [0.21]

C	2.81922	1.57258	0.07700
C	3.52124	2.76611	0.18322
C	2.85388	3.98997	0.21728
C	1.47653	4.03183	0.16573
C	0.72673	2.85125	0.02875
C	1.40640	1.61611	-0.06396
C	-0.72678	2.85123	-0.02850
C	-1.40641	1.61607	0.06420
C	-0.66665	0.37570	0.17436
C	0.66666	0.37571	-0.17400
C	-1.47665	4.03176	-0.16562
C	-2.85399	3.98983	-0.21720
C	-3.52133	2.76595	-0.18310
C	-2.81923	1.57247	-0.07686
C	1.40509	-0.81815	-0.62418
C	3.49928	0.26531	0.14265
C	-3.49925	0.26518	-0.14248
C	-1.40506	-0.81823	0.62431
C	0.82999	-1.84902	-1.36547
C	1.56685	-3.04658	-1.54814
C	2.83120	-3.20853	-1.05693
C	3.50167	-2.11516	-0.45392
C	2.79798	-0.89229	-0.30221
C	4.84721	-2.20411	-0.02833
C	5.47812	-1.10532	0.49779
C	4.81006	0.12973	0.56373
C	-2.79793	-0.89242	0.30229
C	-3.50158	-2.11533	0.45391
C	-2.83109	-3.20867	1.05695
C	-1.56673	-3.04672	1.54814
C	-0.82992	-1.84913	1.36553
C	-4.81001	0.12959	-0.56360
C	-5.47803	-1.10549	-0.49784
C	-4.84711	-2.20429	0.02827
C	-0.51989	-1.79121	-2.03170
C	0.51994	-1.79123	2.03182
H	4.60427	2.75945	0.24140
H	3.42160	4.91125	0.30263
H	0.97605	4.99037	0.24327
H	-0.97628	4.99036	-0.24337
H	-3.42174	4.91110	-0.30244
H	-4.60438	2.75925	-0.24102
H	1.08676	-3.86392	-2.08042
H	3.35320	-4.15530	-1.16481
H	5.37041	-3.15135	-0.12814
H	6.50564	-1.17523	0.84129
H	5.34860	0.98693	0.95260
H	-3.35304	-4.15547	1.16480
H	-1.08669	-3.86400	2.08054
H	-5.34844	0.98678	-0.95261
H	-6.50549	-1.17542	-0.84153
H	-5.37026	-3.15156	0.12805
H	-0.89707	-0.77132	-2.12488
H	-0.43984	-2.22112	-3.03603
H	-1.26945	-2.37500	-1.48682
H	1.26967	-2.37457	1.48670
H	0.89684	-0.77126	2.12539
H	0.44009	-2.22157	3.03598

S_1 minimum 1.96 [0.08] 2.04 [1.76] 2.07 [0.31]

C	2.87916	1.59382	-0.04906	C	-1.43816	-3.18823	0.94059
C	3.58206	2.80094	0.00717	C	-0.70392	-1.95956	0.94036
C	2.90764	4.00809	0.10132	C	-4.86523	0.26370	-0.41228
C	1.52128	4.05273	0.12982	C	-5.50257	-0.97539	-0.44460
C	0.76574	2.88138	0.03992	C	-4.81965	-2.13837	-0.13770
C	1.45620	1.64042	-0.05177	C	-0.41949	-1.91329	-1.91920
C	-0.70056	2.89565	0.00748	C	0.34543	-1.85277	2.03431
C	-1.40358	1.66449	0.06257	H	4.66539	2.80957	-0.02242
C	-0.68531	0.43435	0.16570	H	3.47231	4.93417	0.15608
C	0.70641	0.41850	-0.12515	H	1.03507	5.01629	0.22319
C	-1.43159	4.08401	-0.05869	H	-0.91928	5.03788	-0.10811
C	-2.81902	4.07821	-0.05768	H	-3.36532	5.01529	-0.08342
C	-3.50513	2.87223	-0.03367	H	-4.58970	2.88687	-0.03685
C	-2.82096	1.65610	-0.00073	H	0.70857	-4.14486	-0.89763
C	1.37028	-0.79178	-0.45335	H	3.10099	-4.28708	-0.43703
C	3.55135	0.29454	-0.05888	H	5.27255	-3.21038	-0.05758
C	-3.51646	0.36880	-0.07251	H	6.62488	-1.15422	0.29581
C	-1.39051	-0.77394	0.47140	H	5.54002	1.03580	0.31663
C	0.58739	-1.97482	-0.78413	H	-3.24475	-4.23401	0.49803
C	1.28124	-3.24386	-0.69628	H	-0.91966	-4.07676	1.29653
C	2.60835	-3.31735	-0.45098	H	-5.43212	1.14625	-0.68509
C	3.42210	-2.14371	-0.27030	H	-6.55274	-1.02419	-0.71821
C	2.77624	-0.87603	-0.26973	H	-5.32875	-3.09752	-0.15641
C	4.79787	-2.23212	-0.06943	H	-0.86963	-0.92706	-2.02947
C	5.55445	-1.08443	0.13277	H	0.11747	-2.14500	-2.84614
C	4.92963	0.15724	0.13811	H	-1.21039	-2.65878	-1.78886
C	-2.79262	-0.82800	0.21257	H	1.09914	-2.64173	1.92368
C	-3.46051	-2.09137	0.19897	H	0.85825	-0.89144	2.03616
C	-2.72695	-3.27983	0.52572	H	-0.13181	-1.97490	3.01303

MECI 2.09 [0.12] 2.10 [0.08] 2.18 [1.95]

C	2.89376	1.56789	-0.03751	C	-1.51911	-3.16139	1.09650
C	3.60567	2.77154	0.01850	C	-0.76489	-1.94691	1.08114
C	2.94366	3.98517	0.10734	C	-4.77459	0.29220	-0.63012
C	1.55715	4.04148	0.13897	C	-5.40580	-0.94699	-0.71581
C	0.79038	2.87803	0.08781	C	-4.78706	-2.10999	-0.29585
C	1.47125	1.62692	0.00079	C	-0.46878	-1.93264	-1.79873
C	-0.67621	2.90401	0.07496	C	0.29327	-1.82074	2.15900
C	-1.38082	1.67521	0.11378	H	4.68867	2.77460	-0.00325
C	-0.67954	0.43962	0.24724	H	3.51565	4.90801	0.14842
C	0.71268	0.40901	-0.03715	H	1.08286	5.01428	0.20623
C	-1.40390	4.09606	0.03001	H	-0.89357	5.05353	0.01345
C	-2.79311	4.09567	0.01839	H	-3.33491	5.03676	0.01586
C	-3.47778	2.88953	-0.00581	H	-4.56276	2.89557	-0.02461
C	-2.79217	1.67436	0.01023	H	0.61176	-4.16820	-0.66870
C	1.34326	-0.81006	-0.36557	H	3.02320	-4.31470	-0.40842
C	3.55274	0.26010	-0.10540	H	5.24272	-3.25219	-0.24958
C	-3.47384	0.38947	-0.13554	H	6.64310	-1.19270	-0.08528
C	-1.39935	-0.76112	0.54784	H	5.57716	0.99556	0.02532
C	0.52361	-1.98420	-0.65411	H	-3.30779	-4.20780	0.57596
C	1.20212	-3.26608	-0.53683	H	-1.03502	-4.04098	1.51881
C	2.54463	-3.33621	-0.39917	H	-5.28743	1.17513	-0.99533
C	3.39004	-2.17511	-0.29886	H	-6.41414	-0.99456	-1.12083
C	2.75491	-0.90328	-0.25680	H	-5.30360	-3.06480	-0.34504
C	4.77684	-2.26850	-0.22814	H	-0.90681	-0.94370	-1.93572
C	5.56150	-1.12616	-0.12045	H	0.09121	-2.18269	-2.70884
C	4.94517	0.11836	-0.05747	H	-1.26373	-2.67517	-1.68291
C	-2.79181	-0.81073	0.22254	H	1.07164	-2.58483	2.04357
C	-3.46568	-2.06997	0.18045	H	0.77441	-0.84219	2.15489
C	-2.78300	-3.25706	0.60705	H	-0.16378	-1.95383	3.14661

π-extended [7]helicene **2**

S_0 minimum 0.00 [0.17] 1.58[2.17] 2.04[0.17]

C	4.60973	-2.37143	0.70989	C	4.47411	-1.16290	1.34081

C	3.18531	-0.63034	1.59310
C	2.04345	-1.35391	1.15646
C	2.19630	-2.65623	0.59960
C	3.47219	-3.12668	0.35902
C	3.00516	0.60305	2.26239
C	1.74141	1.07562	2.51558
C	0.61724	0.39026	2.02668
C	0.75358	-0.76736	1.28163
C	0.98131	-3.45277	0.33849
C	-0.38958	-1.40757	0.61770
C	-0.29541	-2.82229	0.35437
C	-1.44843	-3.55318	-0.01623
C	-1.32247	-4.91562	-0.33096
C	-0.09160	-5.53658	-0.28467
C	1.05174	-4.80807	0.04243
C	-1.49698	-0.72260	0.15056
C	-2.73204	-1.44699	-0.03705
C	-2.72612	-2.86062	-0.06848
C	-3.96206	-0.73609	-0.07484
C	-5.14216	-1.45915	-0.19725
C	-5.13698	-2.85388	-0.23383
C	-3.94802	-3.54717	-0.15618
C	-1.49689	0.72283	-0.15107
C	-3.96182	0.73630	0.07484
C	-0.38937	1.40778	-0.61804
C	-0.29507	2.82262	-0.35456
C	-1.44802	3.55353	0.01628
C	-2.72565	2.86084	0.06867
C	-2.73177	1.44720	0.03701
C	-3.94748	3.54740	0.15636
C	-5.13645	2.85416	0.23406
C	-5.14174	1.45945	0.19735
C	0.98166	3.45312	-0.33867
C	1.05218	4.80846	-0.04224
C	-0.09118	5.53693	0.28496
C	-1.32207	4.91603	0.33106
C	0.75361	0.76716	-1.28194
C	2.19636	2.65600	-0.59968
C	2.04352	1.35351	-1.15642
C	3.18538	0.62977	-1.59293
C	4.47418	1.16238	-1.34051
C	4.60975	2.37091	-0.70955
C	3.47224	3.12634	-0.35900
C	0.61727	-0.39082	-2.02652
C	1.74137	-1.07606	-2.51567
C	3.00512	-0.60340	-2.26269
H	5.59741	-2.77008	0.49674
H	5.34674	-0.59093	1.64361
H	3.62227	-4.09429	-0.10861
H	3.88097	1.15830	2.58523
H	1.60262	2.00506	3.05871
H	-0.37202	0.80469	2.19056
H	-2.19108	-5.48688	-0.63918
H	-0.00642	-6.59249	-0.52360
H	2.00722	-5.32081	0.05820
H	-6.09747	-0.94987	-0.24648
H	-6.07744	-3.39262	-0.30098
H	-3.96873	-4.63111	-0.13768
H	-3.96797	4.63131	0.13718
H	-6.07690	3.39287	0.30102
H	-6.09690	0.94991	0.24602
H	2.00745	5.32167	-0.05792
H	-0.00609	6.59283	0.52409
H	-2.19063	5.48764	0.63880
H	5.34699	0.59088	-1.64370
H	5.59740	2.76980	-0.49666
H	3.62217	4.09434	0.10791
H	-0.37180	-0.80580	-2.18992
H	1.60257	-2.00548	-3.05885
H	3.88074	-1.15841	-2.58643

S_1 minimum 0.41 [0.24] 1.14 [2.21] 1.62 [0.21]

C	4.60442	-2.48032	0.91542
C	4.45799	-1.21887	1.44858
C	3.17571	-0.65648	1.60724
C	2.03775	-1.39506	1.18014
C	2.19701	-2.71321	0.67142
C	3.48126	-3.22747	0.54313
C	2.99474	0.63715	2.16826
C	1.73586	1.14880	2.34430
C	0.61123	0.44957	1.87708
C	0.74722	-0.78525	1.23286
C	0.99542	-3.47913	0.32004
C	-0.34566	-1.41307	0.54049
C	-0.26277	-2.81825	0.26884
C	-1.43145	-3.53806	-0.09220
C	-1.31470	-4.88919	-0.42741
C	-0.08739	-5.53151	-0.37454
C	1.05139	-4.83700	0.00912
C	-1.52013	-0.70205	0.15409
C	-2.74783	-1.42855	0.00567
C	-2.72282	-2.84676	-0.08090
C	-3.98391	-0.72936	-0.05461
C	-5.16391	-1.46623	-0.18304
C	-5.13991	-2.85199	-0.23295
C	-3.93318	-3.53531	-0.18103
C	-1.51973	0.70260	-0.15380
C	-3.98355	0.73107	0.05432
C	-0.34487	1.41306	-0.54007
C	-0.26139	2.81820	-0.26849
C	-1.42975	3.53854	0.09253
C	-2.72143	2.84784	0.08122
C	-2.74711	1.42966	-0.00559
C	-3.93144	3.53699	0.18128
C	-5.13852	2.85428	0.23268
C	-5.16322	1.46853	0.18248
C	0.99702	3.47863	-0.32002
C	1.05352	4.83658	-0.00954
C	-0.08494	5.53161	0.37425
C	-1.31246	4.88970	0.42754
C	0.74767	0.78487	-1.23267
C	2.19828	2.71222	-0.67134
C	2.03845	1.39417	-1.18009
C	3.17606	0.65521	-1.60738
C	4.45858	1.21708	-1.44878
C	4.60557	2.47836	-0.91538
C	3.48273	3.22594	-0.54295
C	0.61111	-0.44983	-1.87694
C	1.73541	-1.14945	-2.34439
C	2.99453	-0.63832	-2.16847
H	5.59474	-2.90957	0.79391
H	5.32733	-0.64023	1.74883
H	3.63626	-4.21745	0.12860
H	3.87043	1.20289	2.47331
H	1.59849	2.11711	2.81534
H	-0.37643	0.85422	2.05811
H	-2.18540	-5.45236	-0.74377
H	-0.01525	-6.58315	-0.63314

H	1.99360	-5.37018	0.06319
H	-6.12115	-0.96174	-0.24297
H	-6.07118	-3.40480	-0.31163
H	-3.94552	-4.61902	-0.21168
H	-3.94321	4.62071	0.21218
H	-6.06953	3.40755	0.31125
H	-6.12075	0.96450	0.24192
H	1.99587	5.36950	-0.06417
H	-0.01243	6.58335	0.63248
H	-2.18293	5.45324	0.74396
H	5.32768	0.63810	-1.74909
H	5.59607	2.90718	-0.79386
H	3.63813	4.21574	-0.12816
H	-0.37672	-0.85420	-2.05769
H	1.59761	-2.11770	-2.81546
H	3.86996	-1.20443	-2.47360

MECI 1.87 [0.36] 1.87 [0.21] 1.99 [1.72]

C	5.37828	0.44934	1.48193
C	4.53089	1.33087	2.09923
C	3.12846	1.13942	2.04032
C	2.60167	0.01759	1.33369
C	3.50813	-0.92388	0.75395
C	4.86712	-0.67722	0.81638
C	2.24538	2.04862	2.67607
C	0.89746	1.83341	2.62282
C	0.36839	0.75383	1.88995
C	1.17984	-0.14386	1.21717
C	2.96137	-2.15315	0.14787
C	0.66793	-1.20095	0.32369
C	1.55614	-2.30674	0.02570
C	1.04833	-3.52908	-0.47033
C	1.92605	-4.50765	-0.95903
C	3.29246	-4.33288	-0.88147
C	3.79567	-3.17705	-0.29185
C	-0.73919	-1.37756	0.05041
C	-1.27192	-2.70289	-0.10423
C	-0.38698	-3.77828	-0.38003
C	-2.67783	-2.92082	0.00435
C	-3.16060	-4.21681	-0.17185
C	-2.29052	-5.26471	-0.44062
C	-0.92621	-5.05817	-0.54312
C	-1.67496	-0.29930	-0.12156
C	-3.57035	-1.79963	0.30390
C	-1.23916	0.98576	-0.53926
C	-2.04000	2.14846	-0.29836
C	-3.37392	1.98292	0.16576
C	-3.91640	0.62774	0.33792
C	-3.05749	-0.48947	0.17152
C	-5.25191	0.40005	0.67506
C	-5.74460	-0.88627	0.85300
C	-4.90771	-1.97663	0.66058
C	-1.47868	3.43951	-0.51966
C	-2.29368	4.55463	-0.29315
C	-3.58802	4.39344	0.17302
C	-4.12530	3.13332	0.40761
C	-0.01078	1.11361	-1.19626
C	-0.08585	3.55621	-0.95237
C	0.61652	2.37815	-1.29581
C	1.95911	2.45114	-1.74317
C	2.58949	3.68857	-1.83270
C	1.90432	4.85260	-1.49691
C	0.58602	4.77958	-1.06524
C	0.65983	-0.09298	-1.59626
C	2.02398	0.00802	-2.02997
C	2.62675	1.22105	-2.09041
H	6.45367	0.60042	1.52229
H	4.92202	2.18624	2.64481
H	5.57338	-1.35615	0.35170
H	2.65966	2.90054	3.20884
H	0.21341	2.50800	3.12921
H	-0.70594	0.62885	1.88096
H	1.53291	-5.41445	-1.40455
H	3.96887	-5.09870	-1.24806
H	4.86953	-3.08382	-0.18216
H	-4.22390	-4.41760	-0.12101
H	-2.68640	-6.26808	-0.56309
H	-0.27639	-5.90593	-0.71997
H	-5.93260	1.23288	0.81257
H	-6.78208	-1.03886	1.13231
H	-5.31195	-2.97419	0.79818
H	-1.92797	5.55756	-0.48345
H	-4.19998	5.27191	0.35883
H	-5.14099	3.06706	0.77820
H	3.62459	3.73666	-2.15984
H	2.39891	5.81443	-1.56696
H	0.07685	5.70366	-0.81327
H	0.05982	-0.87898	-2.05127
H	2.54501	-0.88869	-2.34527
H	3.64437	1.28597	-2.46859

π-extended [9]helicene **3**

S_0 minimum 0.00 [0.15] 1.38 [2.14] 1.73 [0.16]

C	3.64381	-3.53003	1.61683
C	4.17393	-2.29091	1.86114
C	3.32205	-1.16823	2.01103
C	1.91901	-1.34130	1.86131
C	1.37811	-2.64979	1.69790
C	2.24817	-3.71101	1.55700
C	3.83113	0.11628	2.31541
C	2.97341	1.16974	2.51119
C	1.59343	1.00767	2.30893
C	1.06440	-0.20410	1.89828
C	-0.08702	-2.81798	1.74463
C	-0.35161	-0.36185	1.52665
C	-0.93348	-1.67689	1.65598
C	-2.33729	-1.84598	1.58347
C	-2.87377	-3.13929	1.69064
C	-2.05054	-4.23407	1.83770
C	-0.66571	-4.07479	1.84993
C	-1.13104	0.66452	1.02193
C	-2.56592	0.56474	1.11245
C	-3.17877	-0.67512	1.40075
C	-3.35522	1.73817	0.98322
C	-4.73695	1.62110	1.02809
C	-5.34706	0.38776	1.26273
C	-4.58092	-0.73813	1.47221
C	-0.56766	1.87277	0.39224
C	-2.69183	3.04930	0.83514
C	0.56589	1.87326	-0.39145
C	1.30402	3.10427	-0.51876
C	0.69616	4.34057	-0.19852
C	-0.70032	4.33993	0.19919
C	-1.30700	3.10307	0.51949
C	-1.46615	5.51571	0.27665

C	-2.78767	5.46662	0.66565	H	4.29536	-4.38904	1.48717
C	-3.39737	4.24080	0.93720	H	5.24816	-2.15021	1.94809
C	2.68888	3.05184	-0.83451	H	1.86946	-4.71089	1.37898
C	3.39321	4.24404	-0.93688	H	4.90449	0.24659	2.42079
C	2.78228	5.46929	-0.66554	H	3.35592	2.14557	2.79356
C	1.46077	5.51713	-0.27633	H	0.93945	1.86228	2.43955
C	1.13041	0.66553	-1.02114	H	-3.94349	-3.29524	1.62241
C	3.35352	1.74136	-0.98267	H	-2.47996	-5.22797	1.92018
C	2.56538	0.56717	-1.11190	H	-0.04162	-4.95578	1.94736
C	3.17939	-0.67204	-1.40060	H	-5.36718	2.49111	0.88272
C	4.58157	-0.73362	-1.47257	H	-6.43036	0.32287	1.30312
C	5.34666	0.39295	-1.26284	H	-5.07730	-1.67531	1.69453
C	4.73537	1.62562	-1.02773	H	-1.02818	6.47128	0.01044
C	0.35194	-0.36155	-1.52589	H	-3.36729	6.38193	0.73452
C	0.93507	-1.67603	-1.65551	H	-4.44301	4.23556	1.22391
C	2.33906	-1.84373	-1.58330	H	4.43878	4.23976	-1.22387
C	0.08978	-2.81799	-1.74417	H	3.36092	6.38520	-0.73480
C	0.66977	-4.07422	-1.84930	H	1.02177	6.47233	-0.01044
C	2.05475	-4.23217	-1.83725	H	5.07882	-1.67016	-1.69563
C	2.87688	-3.13649	-1.69061	H	6.43001	0.32914	-1.30341
C	-1.37553	-2.65128	-1.69785	H	5.36475	2.49619	-0.88218
C	-1.06423	-0.20515	-1.89752	H	0.04652	-4.95584	-1.94650
C	-1.59446	1.00616	-2.30802	H	2.48507	-5.22571	-1.91950
C	-2.97458	1.16690	-2.51032	H	3.94679	-3.29128	-1.62252
C	-3.83125	0.11249	-2.31512	H	-0.94147	1.86154	-2.43849
C	-3.32083	-1.17160	-2.01127	H	-3.35794	2.14244	-2.79245
C	-1.91768	-1.34326	-1.86102	H	-4.90472	0.24165	-2.42074
C	-4.17159	-2.29527	-1.86277	H	-5.24588	-2.15558	-1.95054
C	-3.64037	-3.53396	-1.61866	H	-4.29108	-4.39376	-1.48999
C	-2.24459	-3.71346	-1.55773	H	-1.86510	-4.71305	-1.37977

S_1 minimum 0.39 [0.26] 0.91 [2.19] 1.28 [0.22]

C	3.54137	-3.64976	-1.80514	C	-4.21400	3.52616	-0.88402
C	2.28224	-4.15813	-1.99542	C	-3.16212	-2.64810	0.79643
C	1.16297	-3.29437	-2.04317	C	-4.38714	-3.30972	0.88954
C	1.35438	-1.89788	-1.84991	C	-5.57623	-2.65631	0.59718
C	2.67155	-1.37926	-1.69916	C	-5.56645	-1.32145	0.22517
C	3.73588	-2.26547	-1.66831	C	-0.74493	-1.14047	0.98953
C	-0.13878	-3.78998	-2.29366	C	-1.88704	-3.33630	0.99759
C	-1.19632	-2.92383	-2.41239	C	-0.69723	-2.57105	1.12189
C	-1.01775	-1.55095	-2.18790	C	0.54081	-3.22124	1.35445
C	0.22091	-1.03080	-1.82290	C	0.56330	-4.61567	1.44505
C	2.84827	0.07741	-1.63490	C	-0.59509	-5.36022	1.29597
C	0.39712	0.35308	-1.40588	C	-1.80865	-4.72644	1.07302
C	1.70765	0.92304	-1.51451	C	0.37692	-0.37395	1.40103
C	1.88046	2.33260	-1.44865	C	1.65802	-1.00663	1.51002
C	3.17123	2.86051	-1.49209	C	1.76074	-2.42300	1.44561
C	4.27536	2.03284	-1.60063	C	2.83877	-0.21753	1.63003
C	4.11268	0.65693	-1.68306	C	4.07303	-0.85903	1.67581
C	-0.68562	1.17390	-0.99396	C	4.16739	-2.24139	1.59419
C	-0.56692	2.60098	-1.12476	C	3.02383	-3.01396	1.48862
C	0.70152	3.19008	-1.35692	C	2.73323	1.24625	1.69699
C	-1.71700	3.42456	-0.99905	C	0.26842	1.01604	1.81961
C	-1.56982	4.80922	-1.07205	C	-0.94431	1.59408	2.18436
C	-0.32685	5.38275	-1.29559	C	-1.05725	2.97383	2.40951
C	0.79302	4.58190	-1.44622	C	0.04099	3.78812	2.29329
C	-1.87172	0.65908	-0.36263	C	1.31796	3.23096	2.04410
C	-3.02469	2.80164	-0.79647	C	1.44235	1.82747	1.84815
C	-1.90262	-0.56785	0.35608	C	2.47772	4.03981	2.00048
C	-3.15026	-1.27198	0.44806	C	3.71143	3.47178	1.81166
C	-4.36920	-0.60276	0.16267	C	3.83940	2.08007	1.67118
C	-4.33313	0.82095	-0.16179	H	4.40114	-4.31231	-1.76925
C	-3.08194	1.42640	-0.45037	H	2.12506	-5.22559	-2.12504
C	-5.49186	1.60075	-0.21838	H	4.74745	-1.90471	-1.52423
C	-5.43430	2.93501	-0.58820	H	-0.27835	-4.85939	-2.42475

H	-2.18653	-3.29438	-2.65865
H	-1.86468	-0.88751	-2.30825
H	3.33188	3.92767	-1.40162
H	5.27192	2.46285	-1.62780
H	4.99479	0.03629	-1.78791
H	-2.42683	5.45869	-0.93820
H	-0.23114	6.46284	-1.35481
H	1.74863	5.05689	-1.63397
H	-6.45379	1.16998	0.03512
H	-6.34629	3.52126	-0.64013
H	-4.19903	4.56668	-1.18747
H	-4.42544	-4.34940	1.19386
H	-6.51698	-3.19472	0.65461
H	-6.50582	-0.84136	-0.02433
H	1.49452	-5.13703	1.63223
H	-0.55258	-6.44367	1.35584
H	-2.69773	-5.33231	0.94378
H	4.98564	-0.28342	1.77592
H	5.14168	-2.71982	1.61932
H	3.13152	-4.08791	1.39959
H	-1.82242	0.97135	2.29954
H	-2.02870	3.39219	2.65403
H	-0.04793	4.86304	2.42386
H	2.37167	5.11332	2.13193
H	4.60252	4.09191	1.78198
H	4.83309	1.67122	1.53050

MECI-1 1.67 [0.32] 1.67 [0.32] 1.77 [1.87]

C	-1.23828	-4.76169	-1.93660
C	-2.31176	-3.89062	-2.07348
C	-2.10282	-2.51241	-2.07146
C	-0.79112	-2.00605	-1.92135
C	0.30791	-2.88518	-1.79359
C	0.05251	-4.25936	-1.80148
C	-3.18351	-1.57026	-2.18139
C	-3.00769	-0.22492	-2.12784
C	-1.68860	0.31015	-1.97054
C	-0.60040	-0.60580	-1.86467
C	1.64919	-2.31130	-1.69315
C	0.62769	-0.09053	-1.44336
C	1.79671	-0.90768	-1.51907
C	3.06838	-0.27828	-1.43771
C	4.19243	-1.09334	-1.53144
C	4.05455	-2.46951	-1.68112
C	2.81494	-3.07985	-1.76520
C	0.69524	1.26607	-1.02184
C	1.95712	1.92463	-1.05945
C	3.14526	1.18450	-1.28765
C	2.02448	3.31604	-0.83832
C	3.27088	3.94791	-0.84559
C	4.43276	3.23349	-1.08816
C	4.36128	1.86181	-1.30764
C	-0.44413	1.88627	-0.41466
C	0.77211	4.04126	-0.62143
C	-1.54958	1.17073	0.17645
C	-2.80332	1.87137	0.31894
C	-2.89757	3.25869	0.07395
C	-1.66180	4.01453	-0.12998
C	-0.44353	3.32749	-0.39354
C	-1.63361	5.41139	-0.07867
C	-0.45628	6.10114	-0.31961
C	0.72879	5.43769	-0.59805
C	-3.97526	1.15228	0.66129
C	-5.19169	1.82825	0.72177
C	-5.29359	3.18152	0.40761
C	-4.15034	3.88691	0.08247
C	-1.38867	-0.08221	0.91134
C	-3.86964	-0.29700	0.91237
C	-2.58142	-0.89185	1.04987
C	-2.46737	-2.27818	1.32195
C	-3.63746	-3.04510	1.44402
C	-4.87925	-2.46880	1.27434
C	-4.99568	-1.10513	1.01099
C	-0.18004	-0.59356	1.39045
C	-0.01777	-2.01903	1.50864
C	-1.14261	-2.87487	1.41743
C	1.28378	-2.58894	1.62257
C	1.41555	-3.97201	1.64399
C	0.30795	-4.81040	1.53662
C	-0.95438	-4.26438	1.42358
C	2.46704	-1.70787	1.69522
C	0.95038	0.25327	1.78943
C	0.78615	1.57642	2.16700
C	1.88544	2.40952	2.43322
C	3.16438	1.92728	2.32740
C	3.38098	0.55513	2.05581
C	2.26479	-0.30148	1.83734
C	4.68793	0.01069	2.00612
C	4.86944	-1.33230	1.80698
C	3.76229	-2.19072	1.67272
H	-1.40398	-5.83346	-1.91764
H	-3.32479	-4.27572	-2.15617
H	0.86835	-4.96165	-1.67073
H	-4.18552	-1.96583	-2.32844
H	-3.84741	0.45081	-2.23487
H	-1.48285	1.29600	-2.37976
H	5.18894	-0.67699	-1.45455
H	4.94958	-3.08254	-1.73996
H	2.76071	-4.15311	-1.90598
H	3.34730	5.01219	-0.65055
H	5.39272	3.73852	-1.09716
H	5.28366	1.31991	-1.48475
H	-2.52790	5.97021	0.16985
H	-0.46067	7.18737	-0.28536
H	1.61833	6.01883	-0.80760
H	-6.09217	1.29936	1.01706
H	-6.25954	3.67606	0.42610
H	-4.23675	4.93903	-0.16877
H	-3.57608	-4.09975	1.68785
H	-5.77470	-3.07822	1.36000
H	-5.98654	-0.68706	0.87197
H	2.39626	-4.42502	1.74015
H	0.43958	-5.88832	1.54272
H	-1.80278	-4.92937	1.30847
H	-0.21324	1.99020	2.24055
H	1.70913	3.45016	2.68921
H	4.02199	2.57750	2.47584
H	5.53734	0.67475	2.14384
H	5.87217	-1.75031	1.77036
H	3.95402	-3.24857	1.53537

MECI-2 1.68 [0.32] 1.69 [0.32] 1.76 [2.12]

C	4.01218	3.39273	1.76787
C	2.79573	4.00971	1.90410
C	1.60143	3.25142	1.94057
C	1.66874	1.83493	1.76973

C	2.94724	1.20613	1.65589
C	4.08583	1.99502	1.66616
C	0.34510	3.87396	2.15252
C	-0.77846	3.10106	2.24360
C	-0.72930	1.71781	2.00292
C	0.45594	1.06743	1.68924
C	2.99281	-0.25912	1.54850
C	0.52939	-0.31543	1.20354
C	1.78934	-0.99453	1.37700
C	1.87251	-2.40910	1.33068
C	3.11457	-3.03890	1.35816
C	4.29004	-2.31199	1.47198
C	4.21536	-0.93460	1.59023
C	-0.65313	-1.09991	0.91899
C	-0.61284	-2.53786	1.10069
C	0.64137	-3.19396	1.25418
C	-1.82187	-3.30211	1.06658
C	-1.74515	-4.69341	1.10800
C	-0.51409	-5.32094	1.22253
C	0.65775	-4.59273	1.30855
C	-1.89264	-0.57924	0.41616
C	-3.11867	-2.63123	0.99067
C	-1.94784	0.65370	-0.28241
C	-3.17073	1.41377	-0.33231
C	-4.38894	0.79136	0.05304
C	-4.37578	-0.62525	0.43886
C	-3.12754	-1.27227	0.61432
C	-5.55958	-1.34479	0.62213
C	-5.54736	-2.66889	1.03914
C	-4.32333	-3.29310	1.23071
C	-3.12902	2.77092	-0.77079
C	-4.34180	3.46097	-0.85840
C	-5.52512	2.86478	-0.45396
C	-5.55794	1.55572	0.01209
C	-0.82314	1.17573	-0.96041
C	-1.84098	3.40246	-1.04843
C	-0.69402	2.58619	-1.11887
C	0.57645	3.17012	-1.30909
C	0.66644	4.55219	-1.42777
C	-0.46336	5.36546	-1.38720
C	-1.70416	4.79088	-1.18018
C	0.30906	0.31304	-1.17111
C	1.62978	0.88100	-1.30599
C	1.76706	2.29825	-1.33105

C	2.73806	0.00745	-1.47112
C	4.01085	0.57549	-1.54594
C	4.14729	1.95153	-1.50382
C	3.05477	2.80849	-1.45050
C	2.51711	-1.43748	-1.62289
C	0.10991	-0.98332	-1.81099
C	-1.13641	-1.35388	-2.28382
C	-1.37820	-2.67526	-2.69709
C	-0.36733	-3.60198	-2.65299
C	0.94351	-3.22657	-2.26783
C	1.19519	-1.88502	-1.89039
C	2.01170	-4.15046	-2.25488
C	3.27429	-3.73153	-1.92019
C	3.53152	-2.37638	-1.63528
H	4.92759	3.97844	1.75291
H	2.73646	5.09168	1.99470
H	5.06958	1.54607	1.57754
H	0.30675	4.95501	2.25289
H	-1.74209	3.55535	2.46321
H	-1.65692	1.16467	2.04671
H	3.17715	-4.11740	1.28392
H	5.25336	-2.81415	1.49482
H	5.13701	-0.37711	1.71808
H	-2.64007	-5.29840	1.03252
H	-0.47578	-6.40686	1.25426
H	1.59186	-5.12745	1.42930
H	-6.51975	-0.86703	0.45188
H	-6.47715	-3.20389	1.20406
H	-4.31600	-4.32330	1.57497
H	-4.36787	4.47668	-1.23738
H	-6.45323	3.42939	-0.50323
H	-6.50288	1.14145	0.34376
H	1.63546	5.02763	-1.53530
H	-0.36845	6.44246	-1.46389
H	-2.57467	5.43536	-1.11326
H	4.89304	-0.04596	-1.63493
H	5.14571	2.38040	-1.53443
H	3.22427	3.87608	-1.45129
H	-1.91452	-0.60681	-2.35526
H	-2.36856	-2.95890	-3.03569
H	-0.55523	-4.63214	-2.94349
H	1.81680	-5.19096	-2.50066
H	4.09135	-4.44238	-1.89333
H	4.54950	-2.07847	-1.41491

Supplementary Table 17: Cartesian coordinates (Å) of the ground state equilibrium structures and the MECI structures of [5]-, [7]-, and [9]helicene calculated at the SF-ωB97xd/6-31G(d)//RBHHLYP/6-31G(d) level of theory. Relative energies in eV and the <S^2> values for each state are also shown.

1,14-dimethyl[5]helicene

S_0 minimum 0.00 [0.13] 3.36 [1.11] 3.38 [2.09]

C	3.67950	1.35356	-0.03405	C	-3.67950	1.35357	0.03403
C	3.19521	2.46893	0.60726	C	-0.11074	1.28249	2.01971
C	1.94464	2.40514	1.23558	C	0.11074	1.28249	-2.01970
C	1.12114	1.29464	1.14134	H	4.68764	1.33822	-0.43980
C	1.54047	0.19944	0.32580	H	3.79985	3.36678	0.69095
C	2.88444	0.19539	-0.14158	H	1.62045	3.23953	1.85321
C	0.71903	-0.96500	0.01644	H	3.23251	-3.12115	-0.87702
C	1.37769	-2.18648	-0.24622	H	4.49254	-0.99907	-0.98116
C	2.76595	-2.17975	-0.59949	H	-1.19283	-4.33978	0.34159
C	3.45927	-1.01818	-0.64556	H	1.19282	-4.33978	-0.34158
C	-0.71903	-0.96500	-0.01644	H	-4.49254	-0.99906	0.98117
C	-1.37769	-2.18649	0.24621	H	-3.23251	-3.12115	0.87702
C	-0.65949	-3.40782	0.17440	H	-1.62044	3.23953	-1.85321
C	0.65949	-3.40782	-0.17439	H	-3.79985	3.36678	-0.69096
C	-1.54047	0.19945	-0.32580	H	-4.68764	1.33822	0.43978
C	-2.88444	0.19539	0.14157	H	-0.44838	0.26802	2.24197
C	-3.45927	-1.01817	0.64556	H	0.12929	1.77127	2.96944
C	-2.76596	-2.17974	0.59949	H	-0.95133	1.82198	1.57334
C	-1.12114	1.29464	-1.14133	H	-0.12928	1.77126	-2.96942
C	-1.94464	2.40514	-1.23558	H	0.44839	0.26802	-2.24195
C	-3.19520	2.46893	-0.60727	H	0.95134	1.82198	-1.57332

MECI 4.01 [0.20] 4.01 [0.24] 4.13 [2.01]

C	3.64233	1.13085	-0.04250	C	-3.33262	1.52928	0.11507
C	2.95478	2.38786	0.06319	C	0.12415	1.47681	1.98080
C	1.62252	2.55747	0.25346	C	0.07068	1.29996	-1.99974
C	0.78044	1.39879	0.60791	H	4.70828	1.10209	-0.23350
C	1.43984	0.06550	0.30847	H	3.55211	3.29996	0.00440
C	2.90170	0.01356	0.05570	H	1.21865	3.55520	0.39970
C	0.68830	-1.04441	0.11904	H	3.19202	-3.41550	-0.32182
C	1.27563	-2.37678	-0.08144	H	4.55437	-1.39271	-0.19248
C	2.72590	-2.44080	-0.19392	H	-1.53332	-4.36589	0.15813
C	3.47059	-1.32966	-0.11703	H	0.95145	-4.43988	-0.10802
C	-0.78212	-1.00645	0.04681	H	-4.74527	-0.82898	0.05598
C	-1.58679	-2.23489	0.12381	H	-3.62585	-3.00367	0.32498
C	-0.97622	-3.43692	0.12013	H	-0.82717	3.50768	-0.85163
C	0.47591	-3.46133	-0.02950	H	-3.06133	3.69049	0.31987
C	-1.40723	0.16626	-0.25860	H	-4.39082	1.60255	0.37424
C	-2.86107	0.28681	-0.15862	H	-0.50931	0.62113	2.21629
C	-3.66232	-0.91630	0.01097	H	0.97488	1.45210	2.66992
C	-3.04986	-2.09679	0.16355	H	-0.42336	2.40724	2.15050
C	-0.71459	1.38746	-0.68729	H	-0.60402	1.30561	-2.85934
C	-1.35045	2.62407	-0.48835	H	0.67673	0.39392	-2.01598
C	-2.59240	2.74520	0.08301	H	0.74936	2.15542	-2.06074

[7]helicene

S_0 minimum 0.00 [0.03] 3.46 [1.04] 3.60 [1.03]

C	-0.75512	-3.61073	0.06123	C	1.64107	-0.72561	-0.05117
C	-1.90791	-3.05484	-0.38593	C	2.86222	-1.39787	0.17366
C	-1.89329	-1.77211	-1.02101	C	2.85743	-2.79836	0.43930
C	-0.68245	-1.03992	-1.08346	C	1.68526	-3.48703	0.47974
C	0.47566	-1.52694	-0.35459	C	1.64266	0.72285	0.05122
C	0.47054	-2.86978	0.06117	C	2.86510	1.39261	-0.17415

C	4.09009	0.66226	-0.13499	H	-0.74508	-4.62213	0.45884
C	4.08873	-0.67015	0.13366	H	-2.84506	-3.60316	-0.33986
C	0.47902	1.52648	0.35509	H	3.80052	-3.28993	0.66172
C	0.47636	2.86935	-0.06056	H	1.66596	-4.53401	0.76966
C	1.69216	3.48424	-0.47942	H	5.02373	1.20356	-0.26221
C	2.86297	2.79316	-0.43962	H	5.02125	-1.21350	0.26034
C	-0.67985	1.04142	1.08400	H	1.67485	4.53127	-0.76934
C	-1.88949	1.77562	1.02133	H	3.80694	3.28279	-0.66255
C	-1.90179	3.05852	0.38656	H	-2.83801	3.60839	0.34042
C	-0.74800	3.61249	-0.06046	H	-0.73628	4.62391	-0.45803
C	-3.05134	-1.26187	-1.64964	H	-3.98038	-1.81976	-1.56363
C	-3.00255	-0.10157	-2.38449	H	-3.89709	0.28046	-2.86708
C	-1.77434	0.56399	-2.54785	H	-1.71236	1.44469	-3.17958
C	-0.64293	0.10067	-1.91857	H	0.29796	0.61291	-2.07722
C	-0.64242	-0.09931	1.91899	H	0.29763	-0.61294	2.07812
C	-1.77483	-0.56112	2.54756	H	-1.71457	-1.44220	3.17893
C	-3.00197	0.10626	2.38363	H	-3.89731	-0.27462	2.86561
C	-3.04861	1.26696	1.64926	H	-3.97679	1.82624	1.56291

MECI 4.10 [0.22] 4.10 [0.27] 4.22 [2.04]

C	-2.55168	-2.49830	0.44207	C	-1.34737	1.26544	-2.71250
C	-3.24549	-1.58668	-0.26736	C	-0.58487	0.47642	-1.86330
C	-2.59833	-0.56576	-1.05118	C	-0.54464	-0.12235	1.50030
C	-1.19182	-0.45484	-1.01469	C	-1.87169	0.04079	2.10677
C	-0.46417	-1.21376	0.03264	C	-2.43384	1.26409	2.24907
C	-1.11807	-2.41411	0.52334	C	-1.85393	2.49498	1.77753
C	1.05180	-1.27334	0.00219	H	-3.04875	-3.32215	0.94381
C	1.64376	-2.59778	-0.09763	H	-4.33003	-1.65282	-0.31988
C	0.95495	-3.57945	0.53888	H	1.45075	-4.54190	0.67270
C	-0.37297	-3.45475	1.04324	H	-0.85695	-4.31102	1.49695
C	1.84929	-0.17685	0.10278	H	4.87599	-1.67722	-0.74552
C	3.25785	-0.26662	-0.31098	H	3.49485	-3.69691	-0.52026
C	3.81637	-1.60350	-0.51773	H	3.84854	3.05719	-0.67510
C	3.05876	-2.70637	-0.41678	H	4.96913	0.84624	-0.89231
C	1.27633	1.12287	0.44922	H	-0.42547	4.58738	0.83986
C	2.01380	2.31400	0.04050	H	1.80819	4.48977	-0.14272
C	3.28158	2.16843	-0.40249	H	-4.43799	0.14175	-1.90690
C	3.94180	0.87637	-0.54401	H	-3.32965	1.80294	-3.38500
C	0.08526	1.16999	1.08377	H	-0.84512	1.97006	-3.36845
C	-0.62043	2.45024	1.23422	H	0.49313	0.57201	-1.88473
C	0.08498	3.63257	0.73725	H	0.13723	-0.75309	2.10480
C	1.31298	3.58158	0.19314	H	-2.34323	-0.82364	2.55895
C	-3.35652	0.24991	-1.90276	H	-3.37171	1.32284	2.79969
C	-2.74041	1.17013	-2.72913	H	-2.39093	3.43057	1.88912

[9]helicene

S_0 minimum 0.00 [0.16] 2.13 [2.22] 2.68 [0.25]

C	2.76921	0.71730	2.18451	C	2.14219	-0.66639	-1.63727
C	2.03830	1.87332	2.07902	C	3.51584	-0.68091	-1.57500
C	0.65281	1.82806	1.76949	C	4.23388	0.51617	-1.40468
C	0.03046	0.59290	1.50872	C	3.55804	1.71283	-1.36940
C	0.87540	-0.57661	1.30764	C	-3.55803	1.71285	1.36940
C	2.21942	-0.52317	1.81289	C	-4.23387	0.51618	1.40468
C	0.53778	-1.68850	0.51593	C	-3.51584	-0.68090	1.57500
C	1.27607	-2.92098	0.66798	C	-2.14219	-0.66638	1.63726
C	2.56395	-2.87486	1.31184	H	3.80880	0.75270	2.49821
C	3.02955	-1.70534	1.81093	H	2.49546	2.83641	2.28735
C	-0.53778	-1.68850	-0.51592	H	3.15613	-3.78467	1.35760
C	-1.27608	-2.92098	-0.66797	H	4.02926	-1.64076	2.23239
C	-0.67772	-4.09347	-0.26824	H	-1.18941	-5.04112	-0.40842
C	0.67770	-4.09346	0.26826	H	1.18939	-5.04112	0.40845
C	-0.87540	-0.57661	-1.30765	H	-4.02927	-1.64076	-2.23238
C	-2.21942	-0.52317	-1.81289	H	-3.15613	-3.78467	-1.35758
C	-3.02955	-1.70534	-1.81093	H	-2.49545	2.83641	-2.28736
C	-2.56395	-2.87486	-1.31183	H	-3.80879	0.75270	-2.49822
C	-0.03047	0.59290	-1.50872	H	0.39938	3.97730	1.88868
C	-0.65280	1.82805	-1.76948	H	-2.03382	3.92323	1.49112
C	-2.03830	1.87331	-2.07902	H	2.03383	3.92321	-1.49112
C	-2.76921	0.71729	-2.18452	H	-0.39937	3.97731	-1.88868
C	-0.11980	3.03240	1.75314	H	1.60886	-1.59360	-1.80947
C	-1.45813	3.00259	1.54068	H	4.04565	-1.62418	-1.66190
C	-2.14989	1.75445	1.45614	H	5.31771	0.49701	-1.33927
C	-1.41307	0.54126	1.51593	H	4.10124	2.65134	-1.29210
C	1.41307	0.54125	-1.51594	H	-4.10124	2.65136	1.29210
C	2.14990	1.75443	-1.45614	H	-5.31770	0.49703	1.33927
C	1.45813	3.00258	-1.54068	H	-4.04565	-1.62417	1.66190
C	0.11981	3.03240	-1.75315	H	-1.60887	-1.59360	1.80946

MECI-1 3.53 [0.26] 3.53 [0.19] 3.65 [2.07]

C	3.03147	0.07027	2.23632	C	1.84028	-0.64444	-1.80869
C	2.36744	1.35196	2.42550	C	3.19785	-0.81776	-1.95450
C	1.06193	1.55733	2.13893	C	4.06723	0.28386	-1.86727
C	0.27760	0.42338	1.67295	C	3.54892	1.54505	-1.68296
C	0.97772	-0.78933	1.27366	C	-3.16034	1.68964	1.74713
C	2.37823	-0.97111	1.66943	C	-3.83691	0.45579	1.46368
C	0.38754	-1.67814	0.43304	C	-3.21746	-0.71243	1.16600
C	0.97696	-3.00460	0.25349	C	-1.75573	-0.83120	1.20630
C	2.34992	-3.21796	0.69367	H	4.07733	-0.02246	2.50935
C	3.00729	-2.25656	1.36050	H	2.95649	2.17347	2.82754
C	-0.92301	-1.39397	-0.30188	H	2.81015	-4.18754	0.51760
C	-1.71279	-2.58667	-0.63581	H	4.03006	-2.41467	1.69278
C	-1.30823	-3.82652	-0.19853	H	-1.93645	-4.69505	-0.35032
C	0.08982	-4.00745	0.06491	H	0.45448	-5.03217	0.12367
C	-1.08770	-0.23211	-1.21808	H	-4.26657	-0.99186	-2.21685
C	-2.40674	-0.02284	-1.66319	H	-3.57307	-3.24883	-1.55623
C	-3.29141	-1.15155	-1.76024	H	-2.38519	3.35319	-2.05825
C	-2.92632	-2.39548	-1.37827	H	-3.89672	1.40599	-2.30504
C	-0.14689	0.82780	-1.42562	H	0.88372	3.69102	2.61917
C	-0.64480	2.13525	-1.64157	H	-1.53573	3.78395	2.33007
C	-2.02382	2.34312	-1.89312	H	2.27281	3.91716	-1.50851
C	-2.85813	1.26930	-2.01637	H	-0.15449	4.24764	-1.71463
C	0.33007	2.79908	2.33555	H	1.17738	-1.49465	-1.93940
C	-1.00581	2.84601	2.18163	H	3.59221	-1.81020	-2.14914
C	-1.81083	1.67067	1.85009	H	5.13748	0.14269	-1.98529
C	-1.07260	0.43060	1.62137	H	4.20508	2.41234	-1.67141
C	1.28720	0.62642	-1.54663	H	-3.73230	2.59269	1.92761
C	2.15694	1.74359	-1.54836	H	-4.92428	0.44621	1.52148
C	1.59990	3.06394	-1.52759	H	-3.80745	-1.61136	1.03236
C	0.26211	3.24734	-1.63511	H	-1.42629	-1.69935	1.81129

MECI-2 3.91 [0.13] 3.91 [0.17] 4.04 [2.07]

C	2.28087	-0.94418	-2.53891
C	1.43513	-2.03972	-2.16395
C	0.31494	-1.96098	-1.39746
C	-0.16533	-0.64760	-0.87175
C	0.73042	0.50903	-1.29018
C	1.91759	0.28875	-2.15075
C	0.62014	1.68563	-0.65405
C	1.40788	2.87537	-1.00349
C	2.50187	2.67852	-1.94495
C	2.72623	1.47060	-2.47591
C	-0.28387	1.83344	0.50440
C	-0.70539	3.18189	0.94203
C	-0.11217	4.26056	0.40550
C	1.00054	4.06622	-0.54026
C	-0.61366	0.74797	1.23498
C	-1.66814	0.85156	2.24278
C	-2.17474	2.16527	2.61144
C	-1.72554	3.26328	1.99411
C	-0.00009	-0.61628	1.00424
C	-0.76359	-1.75718	1.53174
C	-2.02158	-1.58906	2.03962
C	-2.38279	-0.27210	2.45724
C	-0.57310	-3.09152	-1.31695
C	-1.89761	-2.93442	-1.12090
C	-2.47650	-1.61136	-1.18341
C	-1.64609	-0.46439	-1.18015
C	1.47860	-0.72077	1.26009
C	2.05583	-2.00616	1.34228
C	1.20073	-3.17092	1.44094
C	-0.13125	-3.06216	1.55797
C	2.34083	0.37817	1.32061
C	3.71604	0.21260	1.42372
C	4.28067	-1.05886	1.46308
C	3.44374	-2.16109	1.43300
C	-3.86197	-1.46157	-1.37754
C	-4.41453	-0.22480	-1.64358
C	-3.58331	0.89391	-1.74244
C	-2.22249	0.76800	-1.52592
H	3.16335	-1.11519	-3.14374
H	1.66436	-3.02002	-2.58191
H	3.12828	3.52979	-2.20054
H	3.54997	1.31560	-3.16650
H	-0.37173	5.26443	0.72095
H	1.53778	4.95594	-0.86649
H	-2.95501	2.22305	3.36746
H	-2.10528	4.24694	2.25327
H	-2.63108	-2.43542	2.33034
H	-3.30435	-0.17746	3.03448
H	-0.14930	-4.08635	-1.43899
H	-2.56167	-3.79095	-1.05326
H	1.67516	-4.14836	1.48561
H	-0.76113	-3.92725	1.73560
H	1.94848	1.38645	1.32597
H	4.34889	1.09286	1.47541
H	5.35619	-1.18631	1.53974
H	3.85800	-3.16491	1.49056
H	-4.48817	-2.35066	-1.36604
H	-5.48266	-0.12957	-1.81579
H	-3.99671	1.86379	-1.99879
H	-1.59313	1.63964	-1.65067

Supplementary Table 18: Cartesian coordinates (Å) of the optimized structures of helicenes in the ground state (S_0) calculated at the B3LYP/6-311G(2d,p) level of theory.

[3]helicene

1	C	3.549348	-0.295226	0.000000	13	C	-2.873160	-1.524057	0.000000
2	C	2.828699	0.875593	0.000000	14	C	-1.496397	-1.561824	0.000001
3	C	1.418595	0.863062	0.000000	15	H	4.632588	-0.271277	0.000000
4	C	0.727182	-0.379663	0.000000	16	H	3.339406	1.832415	0.000001
5	C	1.496397	-1.561824	-0.000001	17	H	1.004789	-2.525260	-0.000001
6	C	2.873160	-1.524057	-0.000001	18	H	3.436241	-2.449917	-0.000001
7	C	0.676666	2.087466	0.000000	19	H	1.226974	3.021937	0.000001
8	C	-0.676666	2.087466	0.000000	20	H	-1.226974	3.021937	0.000000
9	C	-1.418595	0.863062	0.000000	21	H	-3.339406	1.832415	-0.000001
10	C	-0.727182	-0.379663	0.000000	22	H	-4.632588	-0.271277	0.000000
11	C	-2.828699	0.875593	-0.000001	23	H	-3.436241	-2.449917	0.000001
12	C	-3.549348	-0.295226	0.000000	24	H	-1.004789	-2.525260	0.000001

[5]helicene

1	C	-3.739145	-1.320983	-0.051432	22	C	3.739150	-1.320951	0.051455
2	C	-3.301551	-2.424083	0.639207	23	C	0.027584	-1.304664	2.043971
3	C	-2.058505	-2.376995	1.277948	24	C	-0.027544	-1.304665	-2.044007
4	C	-1.194625	-1.299621	1.153432	25	H	-4.739774	-1.287404	-0.467496
5	C	-1.566994	-0.213068	0.303142	26	H	-3.936423	-3.295143	0.750076
6	C	-2.912139	-0.189535	-0.184458	27	H	-1.770707	-3.195355	1.928991
7	C	-0.720486	0.934719	-0.001502	28	H	-3.193001	3.114004	-0.964338
8	C	-1.370290	2.166148	-0.286218	29	H	-4.479338	1.011874	-1.073333
9	C	-2.743883	2.170840	-0.673952	30	H	1.180477	4.313667	0.375539
10	C	-3.455096	1.019410	-0.718142	31	H	-1.180523	4.313657	-0.375542
11	C	0.720472	0.934725	0.001494	32	H	4.479313	1.011911	1.073358
12	C	1.370263	2.166159	0.286214	33	H	3.192961	3.114032	0.964348
13	C	0.653727	3.384008	0.192375	34	H	1.770756	-3.195338	-1.928996
14	C	-0.653764	3.384003	-0.192381	35	H	3.936456	-3.295109	-0.750052
15	C	1.566992	-0.213056	-0.303144	36	H	4.739774	-1.287363	0.467530
16	C	2.912132	-0.189511	0.184470	37	H	0.351354	-0.296859	2.300002
17	C	3.455075	1.019439	0.718158	38	H	-0.212973	-1.826515	2.972263
18	C	2.743853	2.170863	0.673958	39	H	0.878503	-1.815946	1.591903
19	C	1.194645	-1.299612	-1.153441	40	H	0.213040	-1.826512	-2.972295
20	C	2.058537	-2.376977	-1.277947	41	H	-0.878468	-1.815958	-1.591960
21	C	3.301575	-2.424055	-0.639190	42	H	-0.351319	-0.296864	-2.300044

[9]helicene

1	C	-0.712605	-3.708539	0.325566	25	C	-1.773376	0.103081	-2.767797
2	C	-1.849779	-3.289446	-0.280268	26	C	-0.686351	-0.180282	-1.974143
3	C	-1.859148	-2.077258	-1.029926	27	C	-0.685487	0.183579	1.974213
4	C	-0.702559	-1.243408	-1.042361	28	C	-1.774030	-0.095098	2.767498
5	C	0.434330	-1.590126	-0.205239	29	C	-2.943183	0.676241	2.672572
6	C	0.461489	-2.896944	0.339554	30	C	-2.968540	1.755740	1.825333
7	C	1.575540	-0.730141	0.038167	31	H	-0.671478	-4.674420	0.816668
8	C	2.808505	-1.362632	0.357065	32	H	-2.744982	-3.900886	-0.265544
9	C	2.823003	-2.715691	0.792215	33	H	3.769558	-3.154300	1.086966
10	C	1.668625	-3.426850	0.871859	34	H	1.662760	-4.435288	1.269649
11	C	1.578933	0.722958	-0.038037	35	H	4.964978	1.147670	-0.432375
12	C	2.814899	1.349642	-0.357133	36	H	4.959560	-1.170985	0.431611
13	C	4.034754	0.631040	-0.225287	37	H	1.683509	4.427689	-1.269269
14	C	4.031755	-0.649886	0.224879	38	H	3.784259	3.136902	-1.086806
15	C	0.441817	1.588222	0.205448	39	H	-2.726664	3.913910	0.265901
16	C	0.475055	2.894972	-0.339251	40	H	-0.649429	4.678001	-0.815664
17	C	1.684628	3.419190	-0.871584	41	H	-3.853625	-2.378138	-1.777454
18	C	2.835699	2.702602	-0.792120	42	H	-3.805247	-0.423187	-3.288155
19	C	-0.696756	1.246709	1.042478	43	H	-1.717019	0.919180	-3.478284
20	C	-1.849616	2.085797	1.029811	44	H	0.209708	0.408401	-2.092109
21	C	-1.834374	3.298204	0.280639	45	H	0.208015	-0.408916	2.092505
22	C	-0.695171	3.712107	-0.325022	46	H	-1.721410	-0.911343	3.478098
23	C	-2.976379	-1.742335	-1.825840	47	H	-3.803796	0.440033	3.287052
24	C	-2.945977	-0.663080	-2.673233	48	H	-3.842914	2.395477	1.776880

[11]helicene

1	C	-1.621051	-2.520168	-2.315310	23	C	4.492789	1.079499	0.302289
2	C	-1.951618	-1.353572	-2.927803	24	C	3.788870	2.216415	0.086959
3	C	-1.299828	-0.139549	-2.569836	25	C	2.556383	-1.369710	-1.728877
4	C	-0.314665	-0.141424	-1.548735	26	C	3.417363	-2.442367	-1.729153
5	C	-0.272977	-1.291183	-0.669234	27	C	4.589519	-2.417194	-0.957234
6	C	-0.776089	-2.518170	-1.170142	28	C	4.905268	-1.281082	-0.254374
7	C	0.273071	-1.291214	0.669409	29	C	-4.905188	-1.281021	0.253786
8	C	0.776203	-2.518214	1.170266	30	C	-4.589659	-2.417133	0.956743
9	C	0.419437	-3.740112	0.534846	31	C	-3.417686	-2.442342	1.728936
10	C	-0.419256	-3.740095	-0.534811	32	C	-2.556647	-1.369731	1.728826
11	C	0.314650	-0.141512	1.548989	33	C	-2.813347	-0.207527	0.965154
12	C	1.299724	-0.139669	2.570176	34	C	-4.060040	-0.150025	0.275222
13	C	1.951542	-1.353689	2.928104	35	C	-1.950389	0.964640	0.979836
14	C	1.621088	-2.520254	2.315491	36	C	-2.538999	2.195981	0.603119
15	C	1.924328	3.424333	-0.967403	37	C	-3.788567	2.216417	-0.087331
16	C	0.812526	3.428583	-1.747299	38	C	-4.492480	1.079527	-0.302816
17	C	0.132267	2.216369	-2.041197	39	C	-0.593683	0.989704	1.491852
18	C	0.593652	0.989808	-1.491641	40	C	-0.132360	2.216248	2.041499
19	C	1.950431	0.964709	-0.979834	41	C	-0.812551	3.428479	1.747521
20	C	2.539156	2.196029	-0.603231	42	C	-1.924210	3.424265	0.967420
21	C	2.813331	-0.207497	-0.965305	43	C	-1.622700	1.071594	-3.241325
22	C	4.060174	-0.150043	-0.275641	44	C	-0.995836	2.229073	-2.907831

45	C	1.622485	1.071440	3.241777
46	C	0.995633	2.228924	2.908276
47	H	-2.035931	-3.463272	-2.652179
48	H	-2.672918	-1.334481	-3.736758
49	H	0.782182	-4.669888	0.958169
50	H	-0.781987	-4.669857	-0.958176
51	H	2.672776	-1.334624	3.737119
52	H	2.035980	-3.463364	2.652327
53	H	2.406749	4.351940	-0.680826
54	H	0.405002	4.356723	-2.131506
55	H	5.432183	1.100619	0.843366
56	H	4.164595	3.173038	0.432621
57	H	1.679886	-1.408156	-2.356207
58	H	3.189913	-3.308396	-2.339267
59	H	5.254742	-3.272545	-0.946599
60	H	5.834587	-1.219940	0.301549
61	H	-5.834381	-1.219841	-0.302343
62	H	-5.254927	-3.272448	0.945980
63	H	-3.190426	-3.308363	2.339131
64	H	-1.680312	-1.408206	2.356373
65	H	-4.164184	3.173054	-0.433070
66	H	-5.431758	1.100682	-0.844091
67	H	-0.405066	4.356605	2.131800
68	H	-2.406551	4.351888	0.680758
69	H	-2.412779	1.058646	-3.983318
70	H	-1.301739	3.173317	-3.343796
71	H	2.412496	1.058473	3.983843
72	H	1.301485	3.173154	3.344307

[13]helicene

1	C	0.115487	2.669050	3.088843
2	C	-0.382426	1.517445	3.609882
3	C	-0.338601	0.308312	2.860778
4	C	0.234352	0.303101	1.563572
5	C	1.090819	1.417778	1.209646
6	C	0.871761	2.647564	1.883366
7	C	2.189993	1.366805	0.261183
8	C	2.578295	2.592885	-0.343625
9	C	2.161949	3.829620	0.222925
10	C	1.423882	3.850264	1.362445
11	C	2.976560	0.192994	-0.066916
12	C	3.712503	0.205207	-1.276589
13	C	3.880094	1.416457	-2.000637
14	C	3.412466	2.586381	-1.493735
15	C	0.210754	-3.292758	-0.645558
16	C	-0.210619	-3.292714	0.645955
17	C	-0.329298	-2.070711	1.365934
18	C	-0.025974	-0.845093	0.722677
19	C	0.025989	-0.845152	-0.722481
20	C	0.329384	-2.070807	-1.365634
21	C	-0.234363	0.302965	-1.563472
22	C	0.338637	0.308103	-2.860659
23	C	0.838172	-0.902598	-3.417000
24	C	0.736715	-2.069859	-2.728558
25	C	-1.090890	1.417634	-1.209663
26	C	-0.871860	2.647378	-1.883470
27	C	-0.115529	2.668808	-3.088912
28	C	0.382450	1.517184	-3.609847
29	C	2.940941	-0.887853	2.215677
30	C	3.210645	-1.955005	3.040634
31	C	3.199775	-0.934812	0.826190
32	C	3.853535	-2.101610	0.330624
33	C	4.078065	-3.200618	1.188109
34	C	3.753119	-3.140407	2.520517
35	C	-4.077761	-3.200952	-1.188020
36	C	-3.752612	-3.140846	-2.520385
37	C	-3.210115	-1.955465	-3.040527
38	C	-2.940599	-0.888221	-2.215628
39	C	-3.199657	-0.935063	-0.826179
40	C	-3.853421	-2.101853	-0.330602
41	C	-2.976643	0.192840	0.066851
42	C	-3.712738	0.205114	1.276431
43	C	-4.352504	-0.982574	1.743656
44	C	-4.367756	-2.113771	0.999031
45	C	-2.190124	1.366675	-0.261270
46	C	-2.578563	2.592782	0.343394
47	C	-3.412858	2.586323	1.493415
48	C	-3.880483	1.416415	2.000357
49	C	-0.838073	-0.902366	3.417225
50	C	-0.736594	-2.069674	2.728868
51	C	4.352273	-0.982485	-1.743799
52	C	4.367684	-2.113617	-0.999080
53	C	-1.424080	3.850090	-1.362681
54	C	-2.162238	3.829495	-0.223218
55	H	-0.001552	3.612617	3.609720
56	H	-0.866681	1.508975	4.579736
57	H	2.505116	4.750039	-0.235720
58	H	1.199413	4.784095	1.865163
59	H	4.460762	1.398800	-2.915993
60	H	3.641799	3.532030	-1.971647
61	H	0.404901	-4.222619	-1.167510
62	H	-0.404714	-4.222543	1.167985
63	H	1.240866	-0.883173	-4.423416
64	H	1.013107	-3.012234	-3.187254
65	H	0.001492	3.612341	-3.609855
66	H	0.866746	1.508662	-4.579680
67	H	2.553935	0.019168	2.652155
68	H	3.009536	-1.872103	4.102073
69	H	4.551380	-4.086161	0.777926
70	H	3.946014	-3.984666	3.171914
71	H	-4.551087	-4.086484	-0.777828
72	H	-3.945361	-3.985176	-3.171733
73	H	-3.008840	-1.872654	-4.101942
74	H	-2.553562	0.018778	-2.652126
75	H	-4.834532	-0.953445	2.714553
76	H	-4.841307	-3.016709	1.368059
77	H	-3.642304	3.532000	1.971219
78	H	-4.461260	1.398802	2.915645
79	H	-1.240734	-0.882881	4.423654
80	H	-1.012932	-3.012026	3.187645
81	H	4.834173	-0.953411	-2.714760
82	H	4.841238	-3.016560	-1.368093
83	H	-1.199631	4.783892	-1.865460
84	H	-2.505503	4.749931	0.235319

[15]helicene

1	C	1.401253	-3.228009	-0.828282
2	C	1.090426	-2.608733	-1.996863
3	C	0.582864	-1.279175	-1.997558
4	C	0.422832	-0.587475	-0.770724
5	C	1.125391	-1.109808	0.381877
6	C	1.434491	-2.492972	0.389535
7	C	1.578088	-0.325126	1.510945
8	C	1.762900	-0.998819	2.745234
9	C	1.846007	-2.419231	2.768492
10	C	1.793366	-3.128664	1.610715
11	C	1.938492	1.078201	1.460462
12	C	1.899588	1.806300	2.678272
13	C	1.869491	1.109369	3.918827
14	C	1.905134	-0.248479	3.946008
15	C	-1.401215	3.228030	-0.828222
16	C	-1.090397	2.608772	-1.996815
17	C	-0.582849	1.279209	-1.997534
18	C	-0.422827	0.587483	-0.770714
19	C	-1.125379	1.109802	0.381898
20	C	-1.434462	2.492971	0.389581
21	C	-1.578073	0.325108	1.510959
22	C	-1.762873	0.998787	2.745258
23	C	-1.845962	2.419199	2.768538
24	C	-1.793321	3.128649	1.610772
25	C	-1.938489	-1.078216	1.460459
26	C	-1.899586	-1.806329	2.678260
27	C	-1.869477	-1.109414	3.918824
28	C	-1.905109	0.248433	3.946023
29	C	5.230017	-1.722115	-1.946938
30	C	5.623605	-2.262947	-0.748098
31	C	5.228537	-1.641308	0.446611
32	C	4.399359	-0.544017	0.423602
33	C	3.919628	0.003524	-0.788838
34	C	4.417824	-0.568331	-1.996834
35	C	3.089011	1.197542	-0.852636
36	C	3.098455	1.916864	-2.072187
37	C	3.599496	1.310306	-3.263511
38	C	4.179077	0.086465	-3.240568
39	C	2.397267	1.787028	0.278244
40	C	2.226371	3.197897	0.263312
41	C	2.352369	3.917227	-0.955433
42	C	2.678569	3.274016	-2.106126
43	C	-5.230064	1.722112	-1.946923
44	C	-5.623665	2.262927	-0.748080
45	C	-5.228594	1.641283	0.446624
46	C	-4.399399	0.544005	0.423609
47	C	-3.919651	-0.003518	-0.788833
48	C	-4.417851	0.568342	-1.996824
49	C	-3.089016	-1.197525	-0.852641
50	C	-3.098453	-1.916834	-2.072200
51	C	-3.599493	-1.310268	-3.263519
52	C	-4.179088	-0.086435	-3.240565
53	C	-2.397273	-1.787025	0.278235
54	C	-2.226382	-3.197895	0.263286
55	C	-2.352374	-3.917211	-0.955469
56	C	-2.678566	-3.273985	-2.106155
57	C	0.234963	-0.637382	-3.218783
58	C	-0.234949	0.637438	-3.218770
59	C	1.913818	3.228150	2.655941
60	C	1.965675	3.896090	1.474919
61	C	-1.913832	-3.228179	2.655914
62	C	-1.965694	-3.896105	1.474885
63	H	1.690025	-4.272678	-0.811236
64	H	1.168039	-3.133677	-2.942011
65	H	2.021838	-2.913195	3.717454
66	H	1.973282	-4.197617	1.604409

67	H	1.881809	1.685726	4.836988
68	H	1.994672	-0.784302	4.884189
69	H	-1.689976	4.272702	-0.811156
70	H	-1.168005	3.133735	-2.941953
71	H	-2.021782	2.913150	3.717509
72	H	-1.973223	4.197604	1.604482
73	H	-1.881792	-1.685784	4.836978
74	H	-1.994631	0.784246	4.884212
75	H	5.571454	-2.152876	-2.882015
76	H	6.261807	-3.138406	-0.723257
77	H	5.584512	-2.020256	1.397219
78	H	4.139999	-0.071489	1.357621
79	H	3.509872	1.862104	-4.192663
80	H	4.543314	-0.374144	-4.152117
81	H	2.153649	4.982924	-0.949772
82	H	2.709665	3.804451	-3.051115
83	H	-5.571504	2.152876	-2.881998
84	H	-6.261883	3.138376	-0.723234
85	H	-5.584579	2.020217	1.397234
86	H	-4.140041	0.071476	1.357627
87	H	-3.509856	-1.862053	-4.192677
88	H	-4.543324	0.374181	-4.152112
89	H	-2.153658	-4.982909	-0.949820
90	H	-2.709657	-3.804406	-3.051151
91	H	0.406568	-1.166664	-4.149047
92	H	-0.406544	1.166743	-4.149024
93	H	1.831208	3.762066	3.595779
94	H	1.879518	4.976256	1.441060
95	H	-1.831225	-3.762107	3.595746
96	H	-1.879546	-4.976271	1.441012

[17]helicene

1	C	-0.292432	3.480877	0.613410
2	C	0.292510	3.480970	-0.612760
3	C	0.703143	2.259845	-1.216262
4	C	0.483194	1.033577	-0.539589
5	C	-0.483221	1.033504	0.539813
6	C	-0.703133	2.259666	1.216693
7	C	-1.251097	-0.113206	0.979604
8	C	-1.704022	-0.122356	2.322987
9	C	-1.725718	1.090293	3.066828
10	C	-1.337523	2.257734	2.490470
11	C	-1.614665	-1.243423	0.151533
12	C	-1.865203	-2.478629	0.800262
13	C	-2.117305	-2.499557	2.200418
14	C	-2.141437	-1.342121	2.911461
15	C	2.117103	-2.499262	-2.200843
16	C	2.141234	-1.341710	-2.911697
17	C	1.703888	-0.122029	-2.322999
18	C	1.251016	-0.113087	-0.979598
19	C	1.614603	-1.243447	-0.151729
20	C	1.865083	-2.478554	-0.800669
21	C	1.795420	-1.203679	1.284060
22	C	1.640062	-2.419180	1.998470
23	C	1.667602	-3.657500	1.298278
24	C	1.884267	-3.682385	-0.042938
25	C	2.212499	-0.039621	2.041472
26	C	1.893711	-0.012534	3.424395
27	C	1.522724	-1.215209	4.088811
28	C	1.501230	-2.394351	3.414496
29	C	-3.367374	3.502515	-1.597045
30	C	-3.955401	3.465523	-0.373535
31	C	-4.290498	2.219169	0.220906
32	C	-3.929909	1.006062	-0.414187
33	C	-2.991925	1.070217	-1.519472
34	C	-2.893548	2.310245	-2.207290
35	C	-2.212458	-0.039264	-2.041512
36	C	-1.893638	-0.011965	-3.424423
37	C	-1.971618	1.210358	-4.146917
38	C	-2.356144	2.353636	-3.523553
39	C	-1.795394	-1.203430	-1.284261
40	C	-1.640011	-2.418822	-1.998852
41	C	-1.501119	-2.393776	-3.414869
42	C	-1.522603	-1.214533	-4.089006
43	C	6.155915	-1.312020	-1.580376
44	C	6.201337	-2.430710	-0.785915
45	C	5.537002	-2.420445	0.450326
46	C	4.789526	-1.331332	0.834088
47	C	4.664037	-0.185996	0.014129
48	C	5.434682	-0.164178	-1.185940
49	C	3.929912	1.006002	0.414295
50	C	4.290479	2.219210	-0.220618
51	C	5.058425	2.203078	-1.424044
52	C	5.557400	1.048334	-1.925784
53	C	2.991921	1.069964	1.519584
54	C	2.893514	2.309889	2.207584
55	C	3.367295	3.502264	1.597508
56	C	3.955328	3.465468	0.373995
57	C	1.337494	2.258105	-2.490058
58	C	1.725609	1.090746	-3.066633
59	C	-1.884353	-3.682341	0.042342
60	C	-1.667604	-3.657250	-1.298856
61	C	-5.058409	2.202847	1.424351
62	C	-4.663983	-0.186019	-0.014161
63	C	1.971666	1.209685	4.147068
64	C	2.356134	2.353069	3.523863
65	C	-6.155768	-1.312306	1.580252
66	C	-5.434588	-0.164389	1.185939
67	C	-5.536897	-2.420440	-0.450619
68	C	-5.557324	1.048017	1.925952
69	C	-4.789475	-1.331251	-0.834266
70	C	-6.201182	-2.430894	0.785647
71	H	-0.511318	4.410832	1.125655
72	H	0.511424	4.411004	-1.124851
73	H	-2.117068	1.071421	4.077519
74	H	-1.453156	3.200883	3.012262
75	H	-2.354157	-3.447172	2.670492
76	H	-2.442710	-1.335453	3.952705
77	H	2.353913	-3.446805	-2.671084
78	H	2.442463	-1.334878	-3.952953
79	H	1.582935	-4.576910	1.866613
80	H	2.024795	-4.621125	-0.566489
81	H	1.322755	-1.178124	5.153686
82	H	1.330905	-3.330135	3.934686
83	H	-3.221911	4.442474	-2.117229
84	H	-4.257548	4.378413	0.127198
85	H	-1.670972	1.216587	-5.188433
86	H	-2.327815	3.306707	-4.039489
87	H	-1.330777	-3.329481	-3.935195
88	H	-1.322608	-1.177287	-5.153871
89	H	6.710077	-1.277567	-2.512173
90	H	6.773285	-3.298952	-1.091540
91	H	5.617924	-3.272059	1.115280
92	H	4.317673	-1.342945	1.803672
93	H	5.239169	3.147356	-1.925604
94	H	6.126381	1.042237	-2.848818
95	H	3.221812	4.442141	2.117834
96	H	4.257458	4.378440	-0.126599
97	H	1.453157	3.201339	-3.011690
98	H	2.116929	1.072033	-4.077339
99	H	-2.024926	-4.621161	0.565738
100	H	-1.582919	-4.576573	-1.867328
101	H	-5.239169	3.147049	1.926048
102	H	1.671039	1.215751	5.188590
103	H	2.327781	3.306063	4.039940
104	H	-6.709896	-1.277996	2.512074
105	H	-5.617821	-3.271967	-1.115683
106	H	-6.126273	1.041775	2.849005
107	H	-4.317673	-1.342721	-1.803873
108	H	-6.773090	-3.299195	1.091179

Supplementary Table 19: Cartesian coordinates (Å) of the optimized structures of [*n*]rylenes in the ground state (S_0) calculated at the B3LYP/6-311G(2d,p) level of theory.

[3]rylene

1	C	-2.899839	-1.241879	0.000232
2	C	-3.602617	0.000000	0.000000
3	C	-5.032993	0.000000	0.000012
4	C	-5.728854	-1.230354	0.000349
5	C	-5.038269	-2.414374	0.000675
6	C	-3.637419	-2.418512	0.000615
7	C	-2.899840	1.241880	-0.000235
8	C	-3.637420	2.418512	-0.000573
9	C	-5.038270	2.414374	-0.000617
10	C	-5.728855	1.230353	-0.000307
11	C	5.728855	-1.230354	-0.000271
12	C	5.032993	0.000000	0.000015
13	C	3.602617	0.000000	0.000000
14	C	2.899840	-1.241879	-0.000230
15	C	3.637419	-2.418512	-0.000525
16	C	5.038269	-2.414374	-0.000552
17	C	5.728855	1.230353	0.000319
18	C	5.038270	2.414374	0.000606
19	C	3.637420	2.418512	0.000556
20	C	2.899840	1.241879	0.000224
21	C	1.430766	-1.236801	-0.000127
22	C	0.718367	0.000000	-0.000012
23	C	-0.718367	0.000000	-0.000011
24	C	-1.430766	-1.236801	0.000051
25	C	-0.693933	-2.411784	-0.000064
26	C	0.693933	-2.411784	-0.000163
27	C	1.430767	1.236801	0.000089
28	C	0.693933	2.411785	0.000060
29	C	-0.693933	2.411785	-0.000040
30	C	-1.430766	1.236802	-0.000087
31	H	-6.812755	-1.219819	0.000365
32	H	-5.571268	-3.357838	0.000973
33	H	-3.135807	-3.375807	0.000902
34	H	-3.135810	3.375808	-0.000823
35	H	-5.571270	3.357837	-0.000886
36	H	-6.812756	1.219817	-0.000310
37	H	6.812755	-1.219818	-0.000269
38	H	3.135808	-3.375808	-0.000749
39	H	5.571269	-3.357838	-0.000789
40	H	6.812755	1.219817	0.000335
41	H	5.571270	3.357837	0.000867
42	H	3.135809	3.375808	0.000804
43	H	-1.194922	-3.369410	-0.000115
44	H	1.194922	-3.369410	-0.000248
45	H	1.194922	3.369410	0.000083
46	H	-1.194922	3.369410	-0.000056

[4]rylene

1	C	-0.731460	1.232978	-0.000859
2	C	-1.447062	-0.000001	-0.000128
3	C	-2.883028	0.000000	-0.000048
4	C	-3.594063	1.236902	-0.002067
5	C	-2.854469	2.412330	-0.003477
6	C	-1.468354	2.411796	-0.002773
7	C	-0.731459	-1.232979	0.000549
8	C	-1.468354	-2.411797	0.002641
9	C	-2.854470	-2.412331	0.003462
10	C	-3.594063	-1.236902	0.002059
11	C	7.890579	1.230312	0.003357
12	C	7.194706	0.000001	0.000190
13	C	5.764288	0.000000	0.000059
14	C	5.061598	1.242179	0.002761
15	C	5.799699	2.419347	0.006039
16	C	7.200098	2.414759	0.006343
17	C	7.890581	-1.230310	-0.002805
18	C	7.200101	-2.414758	-0.005737
19	C	5.799702	-2.419347	-0.005635
20	C	5.061600	-1.242179	-0.002708
21	C	3.594063	1.236902	0.001968
22	C	2.883028	-0.000001	-0.000112
23	C	1.447062	-0.000001	-0.000162
24	C	0.731459	1.232978	0.000566
25	C	1.468353	2.411796	0.002468
26	C	2.854469	2.412330	0.003241
27	C	3.594063	-1.236902	-0.002236
28	C	2.854470	-2.412330	-0.003999
29	C	1.468354	-2.411796	-0.003302
30	C	0.731460	-1.232979	-0.001019
31	C	-5.061599	1.242178	-0.002712
32	C	-5.764288	0.000000	0.000089
33	C	-7.194706	0.000000	0.000147
34	C	-7.890580	1.230311	-0.002991
35	C	-7.200099	2.414758	-0.006056
36	C	-5.799700	2.419346	-0.005884
37	C	-5.061599	-1.242179	0.002839
38	C	-5.799700	-2.419346	0.006089
39	C	-7.200100	-2.414757	0.006372
40	C	-7.890580	-1.230310	0.003347
41	H	-3.355066	3.370192	-0.004986
42	H	-0.967155	3.369120	-0.004018
43	H	-0.967156	-3.369121	0.003992
44	H	-3.355066	-3.370193	0.005074
45	H	8.974467	1.219706	0.003462
46	H	5.297969	3.376572	0.008550
47	H	7.733498	3.357990	0.008933
48	H	8.974469	-1.219703	-0.002750
49	H	7.733502	-3.357989	-0.008097
50	H	5.297974	-3.376573	-0.007939
51	H	0.967155	3.369120	0.003636
52	H	3.355066	3.370192	0.004671
53	H	3.355066	-3.370192	-0.005852
54	H	0.967156	-3.369120	-0.004907
55	H	-8.974468	1.219705	-0.002979
56	H	-7.733500	3.357990	-0.008589
57	H	-5.297971	3.376572	-0.008379
58	H	-5.297972	-3.376572	0.008564
59	H	-7.733501	-3.357988	0.008958
60	H	-8.974468	-1.219704	0.003418

[5]rylene

1	C	1.431902	1.233041	-0.000855
2	C	0.717622	0.000000	-0.000283
3	C	-0.717622	0.000000	-0.000279
4	C	-1.431902	1.233041	-0.000127
5	C	-0.691984	2.412640	-0.000683
6	C	0.691984	2.412640	-0.001090
7	C	1.431902	-1.233042	0.000245
8	C	0.691984	-2.412640	0.000263
9	C	-0.691984	-2.412640	-0.000138
10	C	-1.431902	-1.233042	-0.000467
11	C	10.051376	1.230285	-0.000951
12	C	9.355525	0.000000	0.000318
13	C	7.925077	0.000000	0.000193
14	C	7.222329	1.242153	-0.001202
15	C	7.960673	2.419612	-0.002357
16	C	9.360852	2.414892	-0.002249
17	C	10.051376	-1.230285	0.001729
18	C	9.360852	-2.414892	0.002995
19	C	7.960672	-2.419612	0.002884
20	C	7.222329	-1.242153	0.001497
21	C	5.755412	1.236836	-0.001339
22	C	5.044353	0.000000	-0.000046
23	C	3.608281	0.000000	-0.000166
24	C	2.892725	1.233243	-0.001265
25	C	3.630366	2.412922	-0.002677
26	C	5.015634	2.413033	-0.002754
27	C	5.755412	-1.236836	0.001339
28	C	5.015634	-2.413033	0.002533
29	C	3.630366	-2.412922	0.002263
30	C	2.892725	-1.233243	0.000806
31	C	-2.892725	1.233243	0.000646
32	C	-3.608281	0.000000	-0.000156
33	C	-5.044353	0.000000	-0.000038
34	C	-5.755412	1.236836	0.001400
35	C	-5.015633	2.413033	0.002529
36	C	-3.630365	2.412922	0.002135
37	C	-2.892726	-1.233243	-0.001075
38	C	-3.630366	-2.412922	-0.002489
39	C	-5.015634	-2.413033	-0.002691
40	C	-5.755412	-1.236836	-0.001378
41	C	-7.222329	1.242153	0.001704
42	C	-7.925077	0.000000	0.000194
43	C	-9.355525	0.000000	0.000313
44	C	-10.051376	1.230285	0.002018
45	C	-9.360851	2.414892	0.003565
46	C	-7.960672	2.419612	0.003405
47	C	-7.222329	-1.242153	-0.001409
48	C	-7.960673	-2.419612	-0.002909
49	C	-9.360852	-2.414891	-0.002855
50	C	-10.051376	-1.230284	-0.001260
51	H	-1.192843	3.370170	-0.000967
52	H	1.192842	3.370170	-0.001497
53	H	1.192842	-3.370170	0.000466
54	H	-1.192842	-3.370171	-0.000057
55	H	11.135258	1.219719	-0.000859
56	H	7.459010	3.376854	-0.003351
57	H	9.894361	3.358063	-0.003194
58	H	11.135257	-1.219719	0.001803

59	H	9.894361	-3.358063	0.004095
60	H	7.459009	-3.376854	0.003930
61	H	3.128982	3.370139	-0.003826
62	H	5.516670	3.370650	-0.003947
63	H	5.516669	-3.370650	0.003676
64	H	3.128982	-3.370139	0.003313
65	H	-5.516669	3.370650	0.003689
66	H	-3.128981	3.370139	0.003165
67	H	-3.128982	-3.370139	-0.003587
68	H	-5.516670	-3.370650	-0.003863
69	H	-11.135257	1.219719	0.002100
70	H	-9.894361	3.358062	0.004915
71	H	-7.459009	3.376853	0.004680
72	H	-7.459010	-3.376853	-0.004162
73	H	-9.894362	-3.358062	-0.004069
74	H	-11.135258	-1.219718	-0.001179

[6]rylene

1	C	-3.593148	-1.232952	-0.000399
2	C	-2.878821	0.000000	-0.000289
3	C	-1.443442	0.000000	-0.000281
4	C	-0.729177	-1.233275	0.000450
5	C	-1.470016	-2.413962	0.000772
6	C	-2.852857	-2.413538	0.000302
7	C	-3.593148	1.232952	-0.000194
8	C	-2.852857	2.413538	-0.000951
9	C	-1.470016	2.413962	-0.001421
10	C	-0.729176	1.233275	-0.001019
11	C	-12.211446	-1.230270	-0.001525
12	C	-11.515611	0.000000	0.000422
13	C	-10.085148	0.000000	0.000248
14	C	-9.382370	-1.242133	-0.001754
15	C	-10.120847	-2.419764	-0.003585
16	C	-11.520897	-2.414970	-0.003504
17	C	-12.211445	1.230270	0.002571
18	C	-11.520896	2.414970	0.004519
19	C	-10.120846	2.419764	0.004300
20	C	-9.382369	1.242133	0.002128
21	C	-7.915815	-1.236775	-0.001739
22	C	-7.204761	0.000000	-0.000046
23	C	-5.768619	0.000000	-0.000173
24	C	-5.052990	-1.233197	-0.001281
25	C	-5.791050	-2.413369	-0.003173
26	C	-7.175845	-2.413343	-0.003460
27	C	-7.915814	1.236775	0.001712
28	C	-7.175844	2.413343	0.003025
29	C	-5.791050	2.413370	0.002524
30	C	-5.052990	1.233197	0.000767
31	C	0.729176	-1.233275	0.000936
32	C	1.443442	0.000000	-0.000200
33	C	2.878821	0.000000	-0.000127
34	C	3.593148	-1.232951	0.001229
35	C	2.852856	-2.413537	0.002636
36	C	1.470015	-2.413961	0.002484
37	C	0.729176	1.233275	-0.001415
38	C	1.470015	2.413961	-0.002952
39	C	2.852857	2.413536	-0.002985
40	C	3.593148	1.232951	-0.001427
41	C	5.052990	-1.233197	0.001129
42	C	5.768619	0.000000	0.000014
43	C	7.204761	0.000000	0.000079
44	C	7.915815	-1.236775	0.001058
45	C	7.175845	-2.413344	0.002076
46	C	5.791050	-2.413370	0.002126
47	C	5.052990	1.233197	-0.001143
48	C	5.791050	2.413370	-0.001959
49	C	7.175845	2.413344	-0.001792
50	C	7.915815	1.236775	-0.000817
51	C	9.382370	-1.242134	0.000968
52	C	10.085149	0.000000	0.000179
53	C	11.515612	0.000000	0.000214
54	C	12.211446	-1.230272	0.000953
55	C	11.520897	-2.414973	0.001621
56	C	10.120847	-2.419766	0.001628
57	C	9.382370	1.242134	-0.000652
58	C	10.120847	2.419766	-0.001303
59	C	11.520897	2.414973	-0.001219
60	C	12.211446	1.230271	-0.000495
61	H	-0.968887	-3.371336	0.001278
62	H	-3.354223	-3.370782	0.000694
63	H	-3.354223	3.370782	-0.001368
64	H	-0.968887	3.371336	-0.001991
65	H	-13.295324	-1.219716	-0.001410
66	H	-9.619197	-3.377000	-0.005112
67	H	-12.054469	-3.358103	-0.004993
68	H	-13.295323	1.219717	0.002689
69	H	-12.054467	3.358103	0.006236
70	H	-9.619195	3.376999	0.005929
71	H	-5.289668	-3.370558	-0.004664
72	H	-7.677047	-3.370865	-0.005035
73	H	-7.677047	3.370866	0.004382
74	H	-5.289668	3.370559	0.003711
75	H	3.354221	-3.370781	0.003894
76	H	0.968885	-3.371334	0.003683
77	H	0.968886	3.371334	-0.004231
78	H	3.354222	3.370780	-0.004296
79	H	7.677048	-3.370867	0.002897
80	H	5.289669	-3.370560	0.002937
81	H	5.289669	3.370560	-0.002686
82	H	7.677047	3.370868	-0.002469
83	H	13.295324	-1.219718	0.000965
84	H	12.054468	-3.358107	0.002154
85	H	9.619196	-3.377003	0.002147
86	H	9.619196	3.377004	-0.001888
87	H	12.054468	3.358107	-0.001743
88	H	13.295324	1.219718	-0.000451

[7]rylene

1	C	14.370934	1.230263	0.000317
2	C	13.675111	0.000000	0.000017
3	C	12.244638	0.000000	0.000008
4	C	11.541846	1.242123	0.000174
5	C	12.280402	2.419867	0.000533
6	C	13.680371	2.415026	0.000608
7	C	14.370934	-1.230262	-0.000274
8	C	13.680371	-2.415026	-0.000564
9	C	12.280402	-2.419866	-0.000502
10	C	11.541846	-1.242123	-0.000162
11	C	10.075516	1.236738	-0.000053
12	C	9.364482	0.000000	-0.000004
13	C	7.928289	0.000000	-0.000009
14	C	7.212625	1.233164	-0.000187
15	C	7.950928	2.413654	-0.000397
16	C	9.335430	2.413549	-0.000322
17	C	10.075516	-1.236738	0.000045
18	C	9.335430	-2.413549	0.000291
19	C	7.950928	-2.413654	0.000357
20	C	7.212625	-1.233164	0.000161
21	C	5.753379	1.232870	-0.000167
22	C	5.039055	0.000000	-0.000012
23	C	3.603569	0.000000	-0.000009
24	C	2.889218	1.233207	-0.000149
25	C	3.630595	2.414550	-0.000285
26	C	5.012781	2.413986	-0.000292
27	C	5.753379	-1.232871	0.000142
28	C	5.012781	-2.413986	0.000268
29	C	3.630595	-2.414550	0.000264
30	C	2.889218	-1.233207	0.000132
31	C	1.432095	1.233162	-0.000143
32	C	0.717776	0.000000	-0.000005
33	C	-0.717776	0.000000	-0.000002
34	C	-1.432095	1.233162	-0.000133
35	C	-0.690764	2.415003	-0.000262
36	C	0.690764	2.415003	-0.000268
37	C	1.432095	-1.233162	0.000132
38	C	0.690764	-2.415003	0.000264
39	C	-0.690764	-2.415003	0.000262
40	C	-1.432095	-1.233163	0.000131
41	C	-2.889218	1.233207	-0.000126
42	C	-3.603569	0.000000	0.000003
43	C	-5.039055	0.000000	0.000007
44	C	-5.753379	1.232870	-0.000088
45	C	-5.012781	2.413986	-0.000227
46	C	-3.630595	2.414550	-0.000249
47	C	-2.889218	-1.233208	0.000128
48	C	-3.630595	-2.414550	0.000245
49	C	-5.012781	-2.413986	0.000229
50	C	-5.753379	-1.232871	0.000104
51	C	-7.212625	1.233163	-0.000038
52	C	-7.928289	0.000000	0.000009
53	C	-9.364482	0.000000	0.000007
54	C	-10.075516	1.236738	0.000081
55	C	-9.335430	2.413549	0.000023
56	C	-7.950928	2.413654	-0.000044
57	C	-7.212625	-1.233164	0.000063
58	C	-7.950928	-2.413655	0.000093
59	C	-9.335430	-2.413550	0.000023
60	C	-10.075516	-1.236738	-0.000063
61	C	-11.541846	1.242123	0.000222
62	C	-12.244638	0.000000	-0.000002
63	C	-13.675111	0.000001	-0.000009
64	C	-14.370934	1.230264	0.000295
65	C	-13.680370	2.415027	0.000594
66	C	-12.280401	2.419867	0.000553
67	C	-11.541846	-1.242123	-0.000222
68	C	-12.280403	-2.419866	-0.000573
69	C	-13.680371	-2.415025	-0.000625
70	C	-14.370935	-1.230261	-0.000323
71	H	15.454810	1.219722	0.000335
72	H	11.778764	3.377104	0.000796
73	H	14.213981	3.358137	0.000887
74	H	15.454810	-1.219721	-0.000282

75	H	14.213981	-3.358137	-0.000830
76	H	11.778764	-3.377103	-0.000756
77	H	7.449529	3.370820	-0.000620
78	H	9.836738	3.371012	-0.000518
79	H	9.836738	-3.371012	0.000470
80	H	7.449530	-3.370820	0.000561
81	H	3.129428	3.371871	-0.000388
82	H	5.514353	3.371111	-0.000391
83	H	5.514353	-3.371111	0.000368
84	H	3.129428	-3.371871	0.000365
85	H	-1.192445	3.372063	-0.000362
86	H	1.192445	3.372063	-0.000371
87	H	1.192445	-3.372063	0.000371
88	H	-1.192445	-3.372063	0.000368
89	H	-5.514353	3.371110	-0.000332
90	H	-3.129428	3.371871	-0.000362
91	H	-3.129427	-3.371872	0.000347
92	H	-5.514353	-3.371111	0.000321
93	H	-9.836738	3.371012	0.000006
94	H	-7.449529	3.370819	-0.000084
95	H	-7.449530	-3.370820	0.000160
96	H	-9.836738	-3.371012	0.000065
97	H	-15.454809	1.219723	0.000299
98	H	-14.213980	3.358138	0.000856
99	H	-11.778763	3.377104	0.000805
100	H	-11.778765	-3.377103	-0.000836
101	H	-14.213983	-3.358136	-0.000900
102	H	-15.454810	-1.219720	-0.000336

[8]rylene

1	C	16.529897	-1.230248	-0.000124
2	C	15.834079	0.000000	0.000021
3	C	14.403601	0.000000	0.000015
4	C	13.700786	-1.242108	-0.000076
5	C	14.439421	-2.419934	-0.000246
6	C	15.839322	-2.415055	-0.000272
7	C	16.529897	1.230248	0.000171
8	C	15.839322	2.415056	0.000311
9	C	14.439420	2.419934	0.000273
10	C	13.700786	1.242108	0.000099
11	C	12.234633	-1.236703	0.000021
12	C	11.523601	0.000000	0.000004
13	C	10.087371	0.000000	-0.000001
14	C	9.371672	-1.233127	0.000081
15	C	10.110161	-2.413851	0.000166
16	C	11.494438	-2.413692	0.000131
17	C	12.234632	1.236703	-0.000008
18	C	11.494438	2.413692	-0.000121
19	C	10.110161	2.413851	-0.000165
20	C	9.371672	1.233127	-0.000088
21	C	7.912871	-1.232804	0.000084
22	C	7.198542	0.000000	-0.000009
23	C	5.762986	0.000000	-0.000012
24	C	5.048567	-1.233143	0.000082
25	C	5.790316	-2.414948	0.000175
26	C	7.172038	-2.414297	0.000175
27	C	7.912871	1.232804	-0.000101
28	C	7.172038	2.414297	-0.000202
29	C	5.790316	2.414948	-0.000207
30	C	5.048568	1.233143	-0.000109
31	C	3.592285	-1.233047	0.000078
32	C	2.877938	0.000000	-0.000013
33	C	1.442257	0.000000	-0.000012
34	C	0.727820	-1.233061	0.000075
35	C	1.469825	-2.415712	0.000160
36	C	2.850514	-2.415569	0.000162
37	C	3.592285	1.233047	-0.000106
38	C	2.850514	2.415569	-0.000190
39	C	1.469825	2.415712	-0.000186
40	C	0.727820	1.233061	-0.000098
41	C	-0.727820	-1.233061	0.000073
42	C	-1.442257	0.000000	-0.000008
43	C	-2.877938	0.000000	-0.000005
44	C	-3.592285	-1.233047	0.000072
45	C	-2.850514	-2.415569	0.000147
46	C	-1.469825	-2.415712	0.000149
47	C	-0.727820	1.233061	-0.000092
48	C	-1.469825	2.415712	-0.000167
49	C	-2.850514	2.415569	-0.000160
50	C	-3.592285	1.233047	-0.000078
51	C	-5.048568	-1.233143	0.000067
52	C	-5.762986	0.000000	0.000000
53	C	-7.198542	0.000000	0.000001
54	C	-7.912871	-1.232804	0.000038
55	C	-7.172038	-2.414297	0.000115
56	C	-5.790316	-2.414948	0.000132
57	C	-5.048567	1.233143	-0.000067
58	C	-5.790316	2.414948	-0.000122
59	C	-7.172038	2.414297	-0.000102
60	C	-7.912871	1.232804	-0.000033
61	C	-9.371672	-1.233127	-0.000007
62	C	-10.087371	0.000000	0.000003
63	C	-11.523601	0.000000	0.000004
64	C	-12.234632	-1.236703	-0.000086
65	C	-11.494438	-2.413692	-0.000098
66	C	-10.110161	-2.413851	-0.000054
67	C	-9.371672	1.233127	0.000011
68	C	-10.110161	2.413851	0.000051
69	C	-11.494438	2.413692	0.000097
70	C	-12.234633	1.236703	0.000093
71	C	-13.700786	-1.242108	-0.000167
72	C	-14.403601	0.000000	0.000006
73	C	-15.834079	0.000000	0.000008
74	C	-16.529896	-1.230249	-0.000212
75	C	-15.839322	-2.415056	-0.000426
76	C	-14.439420	-2.419934	-0.000401
77	C	-13.700786	1.242108	0.000179
78	C	-14.439421	2.419934	0.000418
79	C	-15.839322	2.415055	0.000446
80	C	-16.529897	1.230248	0.000231
81	H	17.613769	-1.219721	-0.000125
82	H	13.937802	-3.377171	-0.000378
83	H	16.372970	-3.358143	-0.000405
84	H	17.613769	1.219721	0.000182
85	H	16.372969	3.358143	0.000447
86	H	13.937802	3.377171	0.000396
87	H	9.608743	-3.370991	0.000256
88	H	11.995810	-3.371112	0.000211
89	H	11.995810	3.371112	-0.000194
90	H	9.608743	3.370991	-0.000255
91	H	5.289096	-3.372217	0.000249
92	H	7.673755	-3.371335	0.000247
93	H	7.673755	3.371334	-0.000280
94	H	5.289096	3.372217	-0.000289
95	H	0.968065	-3.372689	0.000227
96	H	3.352445	-3.372479	0.000230
97	H	3.352445	3.372479	-0.000260
98	H	0.968065	3.372689	-0.000255
99	H	-3.352445	-3.372479	0.000207
100	H	-0.968065	-3.372689	0.000211
101	H	-0.968065	3.372689	-0.000232
102	H	-3.352445	3.372479	-0.000221
103	H	-7.673755	-3.371334	0.000172
104	H	-5.289096	-3.372217	0.000195
105	H	-5.289096	3.372217	-0.000178
106	H	-7.673755	3.371335	-0.000150
107	H	-11.995810	-3.371112	-0.000129
108	H	-9.608743	-3.370991	-0.000070
109	H	-9.608743	3.370991	0.000059
110	H	-11.995810	3.371112	0.000120
111	H	-17.613769	-1.219721	-0.000215
112	H	-16.372969	-3.358143	-0.000611
113	H	-13.937802	-3.377171	-0.000580
114	H	-13.937802	3.377171	0.000601
115	H	-16.372970	3.358142	0.000635
116	H	-17.613769	1.219720	0.000236

[9]rylene

1	C	-10.071355	-1.232892	-0.000446
2	C	-9.357181	0.000001	0.000024
3	C	-7.921404	0.000001	0.000027
4	C	-7.207069	-1.233242	-0.000087
5	C	-7.949066	-2.415505	-0.000327
6	C	-9.330374	-2.414779	-0.000529
7	C	-10.071355	1.232893	0.000499
8	C	-9.330375	2.414780	0.000629
9	C	-7.949067	2.415507	0.000433
10	C	-7.207069	1.233244	0.000152
11	C	-18.687894	-1.230430	-0.001452
12	C	-17.992236	-0.000002	-0.000070
13	C	-16.561657	-0.000002	-0.000054
14	C	-15.858797	-1.242231	-0.001357
15	C	-16.597286	-2.420177	-0.002717
16	C	-17.997219	-2.415259	-0.002770
17	C	-18.687895	1.230425	0.001293
18	C	-17.997221	2.415254	0.002611
19	C	-16.597288	2.420173	0.002584
20	C	-15.858798	1.242228	0.001258
21	C	-14.392694	-1.236790	-0.001204
22	C	-13.681828	-0.000001	-0.000020
23	C	-12.245414	0.000000	0.000000
24	C	-11.529829	-1.233262	-0.000846
25	C	-12.268417	-2.414241	-0.002023
26	C	-13.652507	-2.414009	-0.002218
27	C	-14.392694	1.236788	0.001147
28	C	-13.652509	2.414007	0.002189
29	C	-12.268419	2.414241	0.002026
30	C	-11.529830	1.233262	0.000866
31	C	-5.751438	-1.233103	0.000092
32	C	-5.037221	0.000001	0.000017
33	C	-3.601280	0.000001	0.000005
34	C	-2.886900	-1.233124	0.000179

35	C	-3.629354	-2.416481	0.000386
36	C	-5.009358	-2.416242	0.000329
37	C	-5.751438	1.233105	-0.000046
38	C	-5.009358	2.416245	-0.000296
39	C	-3.629355	2.416484	-0.000370
40	C	-2.886900	1.233127	-0.000178
41	C	-1.432335	-1.233071	0.000153
42	C	-0.718001	0.000001	-0.000014
43	C	0.718000	0.000002	-0.000022
44	C	1.432335	-1.233071	0.000201
45	C	0.689810	-2.416652	0.000305
46	C	-0.689810	-2.416652	0.000273
47	C	-1.432335	1.233074	-0.000176
48	C	-0.689810	2.416655	-0.000316
49	C	0.689810	2.416655	-0.000359
50	C	1.432335	1.233074	-0.000252
51	C	11.529829	-1.233263	0.000641
52	C	12.245415	0.000000	0.000020
53	C	13.681829	-0.000001	0.000030
54	C	14.392694	-1.236790	0.000746
55	C	13.652507	-2.414009	0.001378
56	C	12.268418	-2.414242	0.001321
57	C	11.529829	1.233262	-0.000609
58	C	12.268419	2.414241	-0.001276
59	C	13.652509	2.414008	-0.001314
60	C	14.392695	1.236788	-0.000676
61	C	2.886899	-1.233124	0.000311
62	C	3.601280	0.000001	-0.000026
63	C	5.037221	0.000001	-0.000023
64	C	5.751438	-1.233103	0.000409
65	C	5.009357	-2.416242	0.000792
66	C	3.629354	-2.416481	0.000736
67	C	2.886900	1.233127	-0.000367
68	C	3.629355	2.416484	-0.000804
69	C	5.009359	2.416244	-0.000854
70	C	5.751438	1.233105	-0.000453
71	C	7.207068	-1.233242	0.000452
72	C	7.921404	0.000000	-0.000009
73	C	9.357181	0.000000	0.000001
74	C	10.071355	-1.232893	0.000545
75	C	9.330374	-2.414779	0.000979
76	C	7.949066	-2.415505	0.000927
77	C	7.207069	1.233243	-0.000480
78	C	7.949067	2.415506	-0.000944
79	C	9.330375	2.414779	-0.000979
80	C	10.071355	1.232893	-0.000535
81	C	15.858798	-1.242231	0.000809
82	C	16.561657	-0.000002	0.000049
83	C	17.992236	-0.000002	0.000057
84	C	18.687894	-1.230430	0.000849
85	C	17.997220	-2.415260	0.001603
86	C	16.597287	-2.420178	0.001580
87	C	15.858798	1.242228	-0.000720
88	C	16.597288	2.420174	-0.001482
89	C	17.997221	2.415255	-0.001488
90	C	18.687895	1.230426	-0.000726
91	H	-7.447420	-3.372614	-0.000410
92	H	-9.832590	-3.371628	-0.000672
93	H	-9.832591	3.371628	0.000813
94	H	-7.447422	3.372616	0.000562
95	H	-19.771798	-1.219993	-0.001461
96	H	-16.095454	-3.377362	-0.003788
97	H	-18.530926	-3.358348	-0.003855
98	H	-19.771799	1.219986	0.001283
99	H	-18.530929	3.358343	0.003673
100	H	-16.095458	3.377359	0.003642
101	H	-11.766656	-3.371269	-0.002900
102	H	-14.154284	-3.371283	-0.003154
103	H	-14.154287	3.371281	0.003128
104	H	-11.766659	3.371269	0.002910
105	H	-3.127100	-3.373257	0.000599
106	H	-5.511878	-3.372913	0.000507
107	H	-5.511878	3.372916	-0.000475
108	H	-3.127100	3.373259	-0.000581
109	H	1.192274	-3.373324	0.000390
110	H	-1.192274	-3.373324	0.000339
111	H	-1.192274	3.373327	-0.000391
112	H	1.192273	3.373327	-0.000453
113	H	14.154285	-3.371284	0.001924
114	H	11.766657	-3.371270	0.001845
115	H	11.766659	3.371269	-0.001801
116	H	14.154287	3.371282	-0.001851
117	H	5.511877	-3.372913	0.001137
118	H	3.127099	-3.373256	0.001062
119	H	3.127101	3.373259	-0.001143
120	H	5.511879	3.372915	-0.001209
121	H	9.832590	-3.371628	0.001352
122	H	7.447420	-3.372615	0.001278
123	H	7.447421	3.372616	-0.001298
124	H	9.832591	3.371628	-0.001347
125	H	19.771798	-1.219993	0.000853
126	H	18.530927	-3.358349	0.002219
127	H	16.095455	-3.377363	0.002190
128	H	16.095457	3.377361	-0.002098
129	H	18.530929	3.358345	-0.002098
130	H	19.771799	1.219988	-0.000717

Supplementary Table 20: Cartesian coordinates (Å) of the optimized structures of π-extended [*n*]helicenes in the ground state (S_0) calculated at the B3LYP/6-311G(2d,p) level of theory.

π-extended [11]helicene

1	C	-6.311522	-1.431298	0.789789	57	C	2.085015	3.084110	-1.464037
2	C	-5.598366	-2.419306	1.418063	58	C	0.701141	3.294950	-1.844853
3	C	-4.288957	-2.165039	1.889558	59	C	-0.158889	2.171867	-1.935081
4	C	-3.707630	-0.878621	1.669080	60	C	0.187294	4.567125	-2.140842
5	C	-4.501954	0.164499	1.114141	61	C	-1.128602	4.726216	-2.522171
6	C	-5.774208	-0.141499	0.657327	62	C	-1.959357	3.618089	-2.659472
7	C	-3.553898	-3.141634	2.597343	63	C	3.968440	1.534568	-1.133202
8	C	-2.306270	-2.844305	3.087098	64	C	4.775816	2.629905	-0.846032
9	C	-1.709718	-1.608112	2.809032	65	C	4.267005	3.924860	-0.846828
10	C	-2.351352	-0.644057	2.042369	66	C	2.940141	4.149327	-1.144499
11	C	-3.968549	1.534573	1.133690	67	C	2.351419	-0.644064	-2.042189
12	C	-1.720660	0.640244	1.702593	68	C	4.501835	0.164493	-1.113533
13	C	-2.597535	1.758588	1.457889	69	C	1.709944	-1.608117	-2.808989
14	C	-2.085059	3.084114	1.464154	70	C	2.306552	-2.844310	-3.086933
15	C	-2.940246	4.149331	1.144778	71	C	3.554078	-3.141642	-2.596919
16	C	-4.267168	3.924864	0.847364	72	C	4.288993	-2.165048	-1.888984
17	C	-4.775980	2.629910	0.846673	73	C	3.707622	-0.878628	-1.668627
18	C	-0.339113	0.849862	1.651513	74	C	5.598305	-2.419315	-1.417221
19	C	0.158922	2.171873	1.934837	75	C	6.311334	-1.431307	-0.788804
20	C	-0.701119	3.294957	1.844730	76	C	5.773996	-0.141506	-0.656458
21	C	1.494053	2.333632	2.398282	77	H	-7.309324	-1.631083	0.417005
22	C	1.959495	3.618094	2.658971	78	H	-6.027176	-3.403686	1.567606
23	C	1.128727	4.726224	2.521766	79	H	-6.383747	0.621621	0.193320
24	C	-0.187225	4.567133	2.140632	80	H	-4.001702	-4.113281	2.771626
25	C	0.650568	-0.209593	1.427106	81	H	-1.762943	-3.572291	3.677441
26	C	2.315004	1.142332	2.655878	82	H	-0.719113	-1.411936	3.193313
27	C	0.464724	-1.293850	0.564341	83	H	-2.557397	5.159663	1.115707
28	C	1.148725	-2.521466	0.869959	84	H	-4.918138	4.758185	0.610604
29	C	2.271215	-2.521567	1.740265	85	H	-5.823809	2.485064	0.623541
30	C	2.728914	-1.257216	2.285028	86	H	2.980963	3.770707	2.978236
31	C	1.893316	-0.117770	2.147076	87	H	1.513461	5.715684	2.738733
32	C	3.947270	-1.124821	2.968366	88	H	-0.829904	5.435149	2.094605
33	C	4.326140	0.087713	3.505295	89	H	4.608946	-1.974141	3.065965
34	C	3.520125	1.211961	3.346749	90	H	5.264154	0.172031	4.041164
35	C	0.628103	-3.752606	0.380867	91	H	3.849163	2.150641	3.770465
36	C	1.301037	-4.930608	0.690647	92	H	0.946668	-5.876169	0.304427
37	C	2.430957	-4.923464	1.503393	93	H	2.932393	-5.856176	1.733078
38	C	2.898190	-3.740733	2.037871	94	H	3.747250	-3.764123	2.706712
39	C	-0.464835	-1.293841	-0.564595	95	H	-3.747257	-3.764090	-2.707152
40	C	-0.628236	-3.752597	-0.381139	96	H	-2.932485	-5.856146	-1.733452
41	C	-1.148830	-2.521451	-0.870248	97	H	-0.946820	-5.876158	-0.304718
42	C	-2.271265	-2.521545	-1.740625	98	H	-3.848930	2.150632	-3.771120
43	C	-2.898239	-3.740705	-2.038257	99	H	-5.263933	0.172040	-4.041891
44	C	-2.431053	-4.923438	-1.503742	100	H	-4.608863	-1.974112	-3.066555
45	C	-1.301170	-4.930592	-0.690944	101	H	0.829967	5.435140	-2.094724
46	C	-0.650606	-0.209589	-1.427379	102	H	-1.513302	5.715676	-2.739200
47	C	-1.893285	-0.117763	-2.147466	103	H	-2.980784	3.770703	-2.978869
48	C	-2.728892	-1.257198	-2.285461	104	H	5.823600	2.485059	-0.622693
49	C	-2.314899	1.142331	-2.656351	105	H	4.917930	4.758181	-0.609943
50	C	-3.519951	1.211959	-3.347342	106	H	2.557286	5.159658	-1.115499
51	C	-4.325973	0.087722	-3.505928	107	H	0.719420	-1.411940	-3.193477
52	C	-3.947180	-1.124801	-2.968920	108	H	1.763347	-3.572295	-3.677390
53	C	-1.493960	2.333627	-2.398700	109	H	4.001917	-4.113289	-2.771111
54	C	0.339105	0.849857	-1.651684	110	H	6.027143	-3.403697	-1.566673
55	C	1.720661	0.640239	-1.702538	111	H	7.309059	-1.631092	-0.415816
56	C	2.597491	1.758584	-1.457672	112	H	6.383443	0.621615	-0.192332

π-extended [13]helicene

1	C	-3.689178	4.940696	-2.452610	28	C	1.505791	-0.530307	-2.384133
2	C	-2.808525	4.577594	-3.438587	29	C	1.817086	-1.760800	-3.022164
3	C	-2.381036	3.234075	-3.554742	30	C	0.993819	-2.920746	-2.735977
4	C	-2.848942	2.270627	-2.609077	31	C	-0.235820	-2.724455	-2.055329
5	C	-3.845326	2.643243	-1.663002	32	C	1.345970	-4.217858	-3.138235
6	C	-4.220294	3.975203	-1.583580	33	C	0.510698	-5.285774	-2.884506
7	C	-1.526901	2.816619	-4.599343	34	C	-0.683165	-5.098326	-2.192886
8	C	-1.174659	1.494851	-4.714479	35	C	2.236874	0.645570	-2.714426
9	C	-1.575910	0.565532	-3.746650	36	C	3.318106	0.535763	-3.583776
10	C	-2.342052	0.938072	-2.649659	37	C	3.655207	-0.682127	-4.165636
11	C	-4.494220	1.583052	-0.877668	38	C	2.904315	-1.810493	-3.906542
12	C	-2.735502	-0.017164	-1.602646	39	C	0.278366	0.749840	-0.641990
13	C	-3.953220	0.263071	-0.881833	40	C	1.788572	1.943643	-2.192833
14	C	-4.580537	-0.754756	-0.113365	41	C	0.827870	1.981382	-1.143999
15	C	-5.719565	-0.437672	0.641759	42	C	0.476885	3.220155	-0.546120
16	C	-6.243579	0.836914	0.628294	43	C	1.049031	4.399753	-1.045875
17	C	-5.638411	1.835312	-0.128437	44	C	1.936367	4.361799	-2.100509
18	C	-2.038616	-1.193169	-1.307850	45	C	2.307920	3.144632	-2.664600
19	C	-2.781915	-2.307895	-0.777449	46	C	-0.278398	0.749847	0.641878
20	C	-4.047695	-2.102709	-0.173850	47	C	-0.827980	1.981377	1.143837
21	C	-2.282766	-3.628687	-0.950451	48	C	-0.477065	3.220147	0.545912
22	C	-3.004596	-4.691388	-0.417877	49	C	-1.788693	1.943620	2.192662
23	C	-4.221002	-4.483425	0.225770	50	C	-2.308119	3.144597	2.664377
24	C	-4.746670	-3.212294	0.327014	51	C	-1.936630	4.361764	2.100244
25	C	-0.618212	-1.411162	-1.606054	52	C	-1.049286	4.399730	1.045618
26	C	-1.068346	-3.837026	-1.750709	53	C	-2.236929	0.645538	2.714295
27	C	0.376578	-0.433772	-1.497024	54	C	-0.376555	-0.433742	1.496951

55	C	0.618285	-1.411076	1.606026
56	C	0.235954	-2.724377	2.055328
57	C	-0.993684	-2.920715	2.735966
58	C	-1.817017	-1.760803	3.022110
59	C	-1.505777	-0.530310	2.384047
60	C	-2.904258	-1.810528	3.906473
61	C	-3.655212	-0.682192	4.165526
62	C	-3.318167	0.535699	3.583636
63	C	1.068544	-3.836910	1.750750
64	C	0.683429	-5.098217	2.192959
65	C	-0.510433	-5.285713	2.884571
66	C	-1.345768	-4.217835	3.138259
67	C	2.038684	-1.193018	1.307860
68	C	2.282964	-3.628524	0.950507
69	C	2.735511	-0.016990	1.602686
70	C	3.953235	0.263300	0.881912
71	C	4.580616	-0.754494	0.113457
72	C	4.047836	-2.102471	0.173932
73	C	2.782051	-2.307712	0.777494
74	C	4.746880	-3.212026	-0.326900
75	C	4.221270	-4.483182	-0.225655
76	C	3.004859	-4.691196	0.417965
77	C	4.494175	1.583304	0.877769
78	C	5.638372	1.835620	0.128566
79	C	6.243602	0.837253	-0.628156
80	C	5.719646	-0.437357	-0.641638
81	C	2.341995	0.938213	2.649699
82	C	3.845215	2.643459	1.663097
83	C	1.575841	0.565625	3.746664
84	C	1.174522	1.494915	4.714493
85	C	1.526707	2.816701	4.599381
86	C	2.380852	3.234207	3.554807
87	C	2.848826	2.270789	2.609144
88	C	2.808283	4.577747	3.438675
89	C	3.688944	4.940898	2.452722
90	C	4.220125	3.975437	1.583697
91	H	-4.005670	5.972708	-2.356186
92	H	-2.433014	5.309740	-4.144427
93	H	-4.944277	4.293472	-0.846214
94	H	-1.181514	3.548288	-5.320699
95	H	-0.564779	1.161858	-5.545620
96	H	-1.267208	-0.463837	-3.857451
97	H	-6.189658	-1.193960	1.254286
98	H	-7.129262	1.065196	1.209457
99	H	-6.079842	2.822120	-0.132123
100	H	-2.626982	-5.700738	-0.503911
101	H	-4.768185	-5.328768	0.625941
102	H	-5.720302	-3.080823	0.777921
103	H	2.287467	-4.392545	-3.639983
104	H	0.789783	-6.280171	-3.212396
105	H	-1.313971	-5.955641	-2.002631
106	H	3.912252	1.406468	-3.822718
107	H	4.498843	-0.737587	-4.843484
108	H	3.150418	-2.734829	-4.410500
109	H	0.809381	5.351614	-0.593482
110	H	2.361629	5.282224	-2.482907
111	H	3.016433	3.146619	-3.481092
112	H	-3.016643	3.146573	3.480860
113	H	-2.361951	5.282179	2.482602
114	H	-0.809690	5.351588	0.593190
115	H	-3.150321	-2.734863	4.410453
116	H	-4.498855	-0.737678	4.843364
117	H	-3.912363	1.406379	3.822545
118	H	1.314286	-5.955502	2.002739
119	H	-0.789465	-6.280115	3.212488
120	H	-2.287262	-4.392560	3.640003
121	H	5.720518	-3.080514	-0.777778
122	H	4.768506	-5.328503	-0.625799
123	H	2.627294	-5.700563	0.504005
124	H	6.079760	2.822448	0.132268
125	H	7.129288	1.065577	-1.209298
126	H	6.189787	-1.193620	-1.254157
127	H	1.267183	-0.463759	3.857444
128	H	0.564633	1.161885	5.545614
129	H	1.181266	3.548347	5.320736
130	H	2.432720	5.309870	4.144511
131	H	4.005391	5.972925	2.356315
132	H	4.944111	4.293745	0.846351

π-extended [15]helicene

1	C	2.147163	-2.497508	3.516636
2	C	2.079170	-3.852624	3.872881
3	C	2.835958	-4.795538	3.211475
4	C	3.673088	-4.415167	2.167397
5	C	3.762460	-3.089330	1.757014
6	C	2.992531	-2.104404	2.443994
7	C	3.018245	-0.730288	2.005090
8	C	2.010299	0.132750	2.447074
9	C	1.366090	-0.164440	3.701426
10	C	1.419651	-1.473353	4.242032
11	C	0.762279	0.887143	4.444914
12	C	0.131953	0.580471	5.646015
13	C	0.133058	-0.717753	6.147526
14	C	0.785786	-1.725578	5.468802
15	C	1.001293	4.893142	2.963479
16	C	0.639576	4.661352	4.274124
17	C	0.581211	3.361523	4.769849
18	C	0.892692	2.269908	3.965988
19	C	1.349611	3.829997	2.116893
20	C	1.316051	2.506849	2.628696
21	C	1.644382	1.389074	1.782039
22	C	1.615556	1.577317	0.396075
23	C	1.903304	2.896894	-0.101795
24	C	2.427434	3.047579	-1.416718
25	C	2.706191	4.329654	-1.880594
26	C	2.533466	5.444430	-1.066298
27	C	2.101904	5.296482	0.236034
28	C	1.786228	4.029363	0.747647
29	C	3.158417	-0.430480	-3.794722
30	C	3.693719	0.785064	-4.165364
31	C	3.477815	1.916790	-3.384146
32	C	2.733841	1.850370	-2.210593
33	C	2.418680	-0.557813	-2.609575
34	C	2.228362	0.587673	-1.793953
35	C	1.459227	0.497777	-0.580327
36	C	0.596501	-0.593939	-0.424575
37	C	0.939254	-1.823346	-1.091402
38	C	0.417583	-3.053324	-0.601795
39	C	0.765272	-4.231529	-1.254306
40	C	1.639763	-4.225183	-2.336538
41	C	2.195866	-3.042406	-2.777149
42	C	1.863384	-1.822966	-2.169178
43	C	-1.863320	-1.819606	2.171915
44	C	-2.195961	-3.038115	2.781646
45	C	-1.640053	-4.221607	2.342699
46	C	-0.765591	-4.229629	1.260458
47	C	-0.417749	-3.052410	0.606248
48	C	-0.939262	-1.821666	1.094084
49	C	-0.596440	-0.593286	0.425435
50	C	-1.459126	0.498703	0.579576
51	C	-2.228134	0.590511	1.793150
52	C	-2.418364	-0.553725	2.610550
53	C	-2.733383	1.853899	2.207973
54	C	-3.476686	1.922207	3.381856
55	C	-3.692445	0.791708	4.164872
56	C	-3.157623	-0.424520	3.795777
57	C	-1.786569	4.028133	-0.754090
58	C	-2.102780	5.296010	-0.244720
59	C	-2.534515	5.446042	1.057318
60	C	-2.706793	4.332619	1.873536
61	C	-2.427457	3.049827	1.411956
62	C	-1.903344	2.897054	0.097262
63	C	-1.615484	1.576692	-0.398487
64	C	-1.644334	1.386217	-1.784151
65	C	-1.316011	2.502637	-2.632584
66	C	-1.349781	3.826608	-2.122963
67	C	-0.892505	2.263547	-3.969430
68	C	-0.581226	3.353869	-4.775126
69	C	-0.639752	4.654504	-4.281533
70	C	-1.001496	4.888396	-2.971262
71	C	4.153987	-0.316720	1.166721
72	C	4.636716	-2.690720	0.644133
73	C	4.889312	-1.303799	0.445848
74	C	5.920259	-0.898436	-0.456565
75	C	6.286848	0.464243	-0.517718
76	C	5.674032	1.379632	0.301232
77	C	4.615489	0.992915	1.132675
78	C	5.292223	-3.606989	-0.163242
79	C	6.237759	-3.200807	-1.117313
80	C	6.569565	-1.876761	-1.245519
81	C	-1.419505	-1.480084	-4.239616
82	C	-0.784936	-1.734338	-5.465621
83	C	-0.131715	-0.727691	-6.145604
84	C	-0.130832	0.571372	-5.646245
85	C	-0.761802	0.880012	-4.446002
86	C	-1.365958	-0.170336	-3.701038
87	C	-2.010310	0.128875	-2.447248
88	C	-3.018346	-0.733400	-2.003990
89	C	-2.992845	-2.108148	-2.440902
90	C	-2.147555	-2.502977	-3.512975
91	C	-3.763058	-3.091920	-1.752572
92	C	-3.674359	-4.418292	-2.161335
93	C	-2.837489	-4.800318	-3.205026
94	C	-2.080249	-3.858576	-3.867566
95	C	-4.636945	-2.691596	-0.640026
96	C	-4.154033	-0.318455	-1.166200
97	C	-4.889346	-1.304368	-0.443724
98	C	-5.920068	-0.897566	0.458288
99	C	-6.569251	-1.874659	1.248869
100	C	-6.237538	-3.198919	1.122608
101	C	-5.292262	-3.606613	0.168921
102	C	-4.615473	0.991247	-1.134130
103	C	-5.673865	1.379303	-0.303102
104	C	-6.286563	0.465230	0.517403

105	H	1.417338	-4.170091	4.666175
106	H	2.779925	-5.837700	3.503164
107	H	4.267438	-5.173600	1.677297
108	H	-0.366324	1.356218	6.210552
109	H	-0.359600	-0.930201	7.088973
110	H	0.826047	-2.713279	5.906286
111	H	1.000818	5.906820	2.587837
112	H	0.386100	5.493498	4.920305
113	H	0.288271	3.210356	5.799628
114	H	3.069040	4.474307	-2.888457
115	H	2.763195	6.430965	-1.451355
116	H	2.028943	6.169552	0.869566
117	H	3.301355	-1.287012	-4.438194
118	H	4.273005	0.865478	-5.077673
119	H	3.900644	2.858222	-3.706277
120	H	0.356888	-5.175739	-0.923003
121	H	1.899006	-5.158695	-2.822072
122	H	2.911150	-3.065908	-3.587103
123	H	-2.911243	-3.060347	3.591639
124	H	-1.899427	-5.154386	2.829569
125	H	-0.357341	-5.174370	0.930507
126	H	-3.899076	2.864194	3.702916
127	H	-4.271201	0.873641	5.077382
128	H	-3.300544	-1.280093	4.440529
129	H	-2.030164	6.167984	-0.879799
130	H	-2.764789	6.433133	1.440620
131	H	-3.069824	4.478934	2.881089
132	H	-0.288350	3.201008	-5.804674
133	H	-0.386369	5.485622	-4.929072
134	H	-1.001041	5.902672	-2.597239
135	H	7.076179	0.766755	-1.196268
136	H	5.990576	2.415693	0.296665
137	H	4.143791	1.741623	1.752353
138	H	5.088309	-4.664095	-0.062830
139	H	6.726470	-3.948120	-1.731519
140	H	7.335344	-1.561762	-1.945252
141	H	-0.824921	-2.722751	-5.901511
142	H	0.361559	-0.941762	-7.086360
143	H	0.367809	1.346192	-6.211735
144	H	-4.269080	-5.175823	-1.670287
145	H	-2.782039	-5.842841	-3.495533
146	H	-1.418690	-4.177354	-4.660559
147	H	-7.334857	-1.558565	1.948299
148	H	-6.726130	-3.945261	1.738088
149	H	-5.088375	-4.663874	0.070079
150	H	-4.143837	1.738975	-1.755032
151	H	-5.990372	2.415381	-0.300102
152	H	-7.075714	0.768855	1.195665

π-extended [17]helicene

1	C	-0.819142	-0.619399	4.510005
2	C	-0.266925	-0.301091	5.759733
3	C	-0.363314	0.977450	6.268050
4	C	-0.982810	1.983707	5.531721
5	C	-1.524837	1.727550	4.276500
6	C	-1.472279	0.399612	3.769367
7	C	-2.030008	0.114832	2.472279
8	C	-1.594777	-1.033583	1.801659
9	C	-1.172729	-2.151085	2.605020
10	C	-0.792596	-1.961973	3.960529
11	C	-1.262717	-3.467442	2.070828
12	C	-0.859175	-4.537740	2.863429
13	C	-0.427268	-4.343741	4.171497
14	C	-0.415206	-3.078072	4.721222
15	C	-2.827419	-4.030288	-1.868738
16	C	-2.694560	-5.106952	-1.017574
17	C	-2.205589	-4.927211	0.273206
18	C	-1.853386	-3.665233	0.740748
19	C	-2.507623	-2.733147	-1.440948
20	C	-2.042166	-2.543943	-0.113860
21	C	-1.687089	-1.228856	0.353526
22	C	-1.435939	-0.234295	-0.599437
23	C	-2.110131	-0.334490	-1.868216
24	C	-2.302763	0.837689	-2.651413
25	C	-2.956618	0.723318	-3.873668
26	C	-3.459563	-0.498436	-4.310120
27	C	-3.334470	-1.624933	-3.524162
28	C	-2.664489	-1.570348	-2.293030
29	C	-0.857439	4.593145	-1.208738
30	C	-1.749878	4.553647	-2.259747
31	C	-2.244555	3.336226	-2.716981
32	C	-1.869199	2.136629	-2.121003
33	C	-0.461497	3.415104	-0.559067
34	C	-0.997494	2.176363	-0.997387
35	C	-0.595062	0.943674	-0.369528
36	C	0.595056	0.943648	0.369648
37	C	0.997447	2.176268	0.997672
38	C	1.869135	2.136411	2.121296
39	C	2.244457	3.335942	2.717431
40	C	1.749752	4.553409	2.260344
41	C	0.857325	4.593018	1.209329
42	C	0.461420	3.415048	0.559504
43	C	2.664571	-1.570556	2.292841
44	C	3.334524	-1.625275	3.523986
45	C	3.459532	-0.498884	4.310108
46	C	2.956536	0.722907	3.873811
47	C	2.302730	0.837417	2.651545
48	C	2.110167	-0.334665	1.868181
49	C	1.435976	-0.234331	0.599413
50	C	1.687132	-1.228776	-0.353666
51	C	2.042254	-2.543911	0.113561
52	C	2.507750	-2.733256	1.440617
53	C	1.853471	-3.665110	-0.741169
54	C	2.205718	-4.927132	-0.273780
55	C	2.694747	-5.107006	1.016959
56	C	2.827607	-4.030437	1.868242
57	C	0.792534	-1.961516	-3.960723
58	C	0.415171	-3.077538	-4.721539
59	C	0.427282	-4.343270	-4.171957
60	C	0.859225	-4.537404	-2.863923
61	C	1.262748	-3.467183	-2.071205
62	C	1.172703	-2.150769	-2.605244
63	C	1.594771	-1.033347	-1.801778
64	C	2.030009	0.115124	-2.472295
65	C	1.472264	0.400035	-3.769345
66	C	0.819071	-0.618886	-4.510057
67	C	1.524857	1.728016	-4.276368
68	C	0.982805	1.984298	-5.531552
69	C	0.363223	0.978136	-6.267937
70	C	0.266795	-0.300442	-5.759724
71	C	-3.045365	1.045519	1.964630
72	C	-2.115857	2.789874	3.451621
73	C	-2.912732	2.433691	2.328552
74	C	-3.650428	3.428748	1.640050
75	C	-4.550555	3.019373	0.578648
76	C	-4.861900	1.639529	0.442058
77	C	-4.188935	0.658429	1.258294
78	C	-1.987279	4.134934	3.780704
79	C	-2.664282	5.118010	3.065227
80	C	-3.501052	4.770223	2.025311
81	C	3.650532	3.428932	-1.639802
82	C	3.501191	4.770443	-2.024951
83	C	2.664428	5.118339	-3.064836
84	C	1.987389	4.135342	-3.780387
85	C	2.115931	2.790251	-3.451416
86	C	2.912804	2.433954	-2.328383
87	C	3.045393	1.045748	-1.964567
88	C	4.188940	0.658568	-1.258248
89	C	4.861929	1.639584	-0.441922
90	C	4.550636	3.019449	-0.578420
91	C	5.793076	1.223232	0.555246
92	C	6.384023	2.187991	1.364180
93	C	6.067533	3.536352	1.232812
94	C	5.159878	3.946432	0.280281
95	C	6.119327	-0.202703	0.703224
96	C	4.802628	-0.675664	-1.342327
97	C	5.696686	-1.102277	-0.315948
98	C	6.207986	-2.436353	-0.333189
99	C	7.007541	-2.881924	0.745387
100	C	7.317355	-2.030972	1.774767
101	C	6.893440	-0.693609	1.742883
102	C	4.635461	-1.503138	-2.445373
103	C	5.195774	-2.785636	-2.495989
104	C	5.928580	-3.267849	-1.440258
105	C	-5.159768	3.946439	-0.279983
106	C	-5.793089	1.223288	-0.555118
107	C	-7.007700	-2.881799	-0.745562
108	C	-7.317458	-2.030770	-1.774895
109	C	-6.893484	-0.693428	-1.742917
110	C	-6.119379	-0.202622	-0.703206
111	C	-5.696776	-1.102282	0.315902
112	C	-4.802674	-0.675784	1.342289
113	C	-4.635509	-1.503356	2.445261
114	C	-5.195880	-2.785831	2.495794
115	C	-5.928741	-3.267929	1.440048
116	C	-6.208132	-2.436336	0.333049
117	C	-6.384035	2.188129	-1.363951
118	C	-6.067478	3.536468	-1.232506
119	H	0.253417	-1.057642	6.330252
120	H	0.056165	1.205651	7.240807
121	H	-1.039105	2.977556	5.953856
122	H	-0.881667	-5.543479	2.468115
123	H	-0.122780	-5.195645	4.768047
124	H	-0.131476	-2.956008	5.757330
125	H	-3.168249	-4.197707	-2.880616
126	H	-2.957245	-6.101632	-1.358178
127	H	-2.102548	-5.791696	0.914187
128	H	-3.084975	1.592507	-4.503133
129	H	-3.970908	-0.558245	-5.263731
130	H	-3.778888	-2.552064	-3.857576
131	H	-0.447146	5.544495	-0.900874
132	H	-2.054864	5.473159	-2.745517
133	H	-2.933357	3.335269	-3.549911
134	H	2.933242	3.334893	3.550377

135	H	2.054708	5.472869	2.746234
136	H	0.447034	5.544402	0.901566
137	H	3.778977	-2.552431	3.857285
138	H	3.970842	-0.558804	5.263731
139	H	3.084815	1.592015	4.503407
140	H	2.102683	-5.791545	-0.914858
141	H	2.957468	-6.101719	1.357439
142	H	3.168467	-4.197964	2.880092
143	H	0.131427	-2.955360	-5.757630
144	H	0.122822	-5.195117	-4.768600
145	H	0.881765	-5.543189	-2.468727
146	H	1.039131	2.978179	-5.953608
147	H	-0.056289	1.206440	-7.240655
148	H	-0.253613	-1.056922	-6.330277
149	H	-1.357267	4.433084	4.607091
150	H	-2.552723	6.158881	3.345162
151	H	-4.064543	5.543969	1.522954
152	H	4.064707	5.544133	-1.522535
153	H	2.552892	6.159237	-3.344678
154	H	1.357369	4.433577	-4.606735
155	H	7.111959	1.896753	2.108479
156	H	6.537272	4.264661	1.883400
157	H	4.909407	4.995333	0.208183
158	H	7.374431	-3.902041	0.739067
159	H	7.919183	-2.377086	2.606991
160	H	7.193850	-0.042158	2.551906
161	H	4.050226	-1.160428	-3.286232
162	H	5.026170	-3.401745	-3.370795
163	H	6.326303	-4.276117	-1.454559
164	H	-4.909238	4.995322	-0.207826
165	H	-7.374643	-3.901898	-0.739308
166	H	-7.919281	-2.376808	-2.607154
167	H	-7.193832	-0.041913	-2.551910
168	H	-4.050225	-1.160750	3.286127
169	H	-5.026280	-3.402013	3.370549
170	H	-6.326518	-4.276178	1.454287
171	H	-7.112026	1.896975	-2.108228
172	H	-6.537204	4.264842	-1.883032

3. Charts of ^{1}H and ^{13}C NMR Spectra

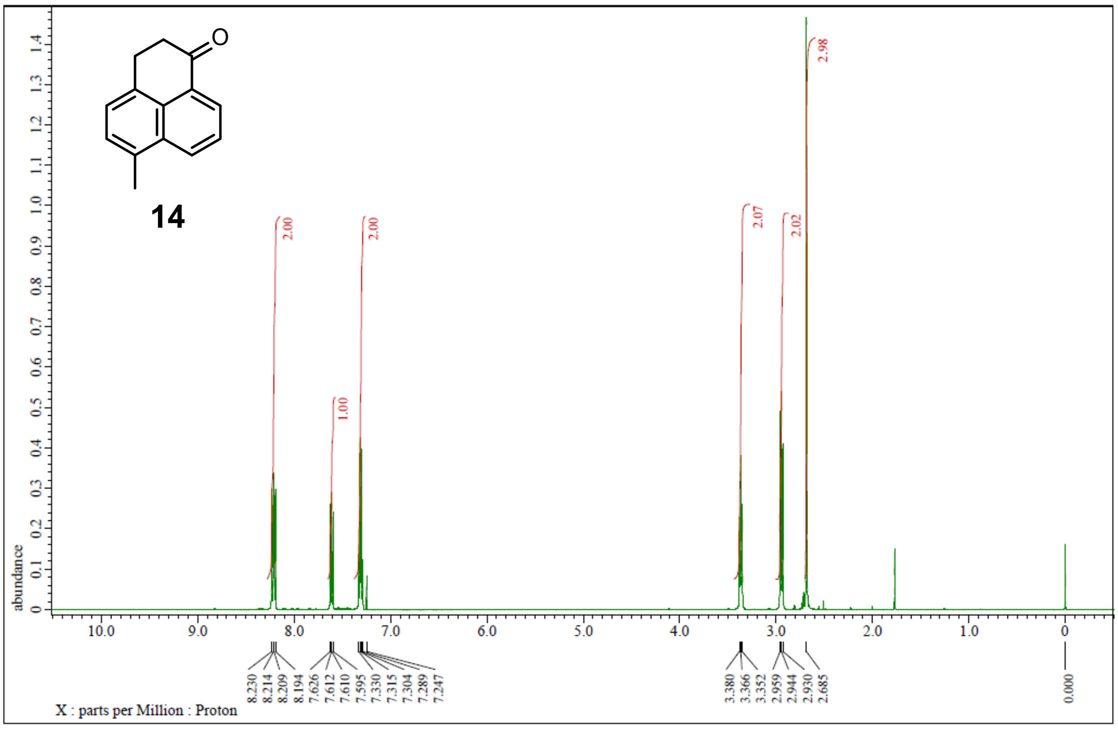

Supplementary Figure 33: ^{1}H NMR spectrum of 14 at room temperature ($CDCl_3$, 500 MHz)

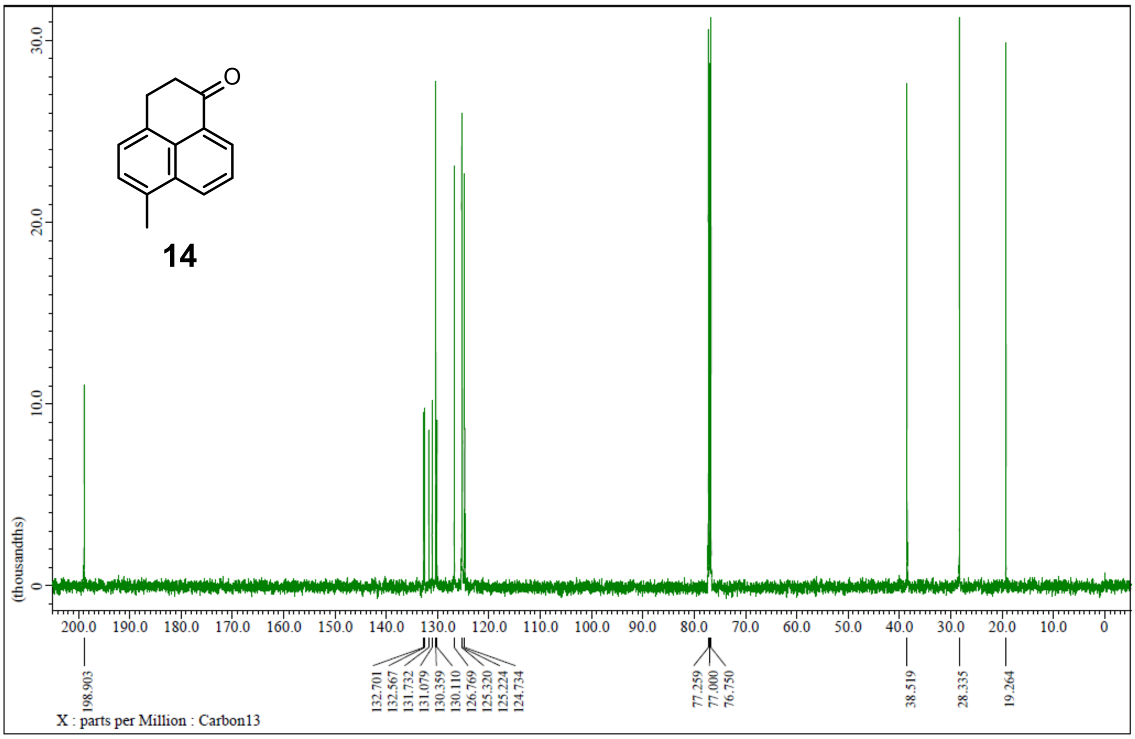

Supplementary Figure 34: ^{13}C NMR spectrum of 14 at room temperature ($CDCl_3$, 126 MHz)

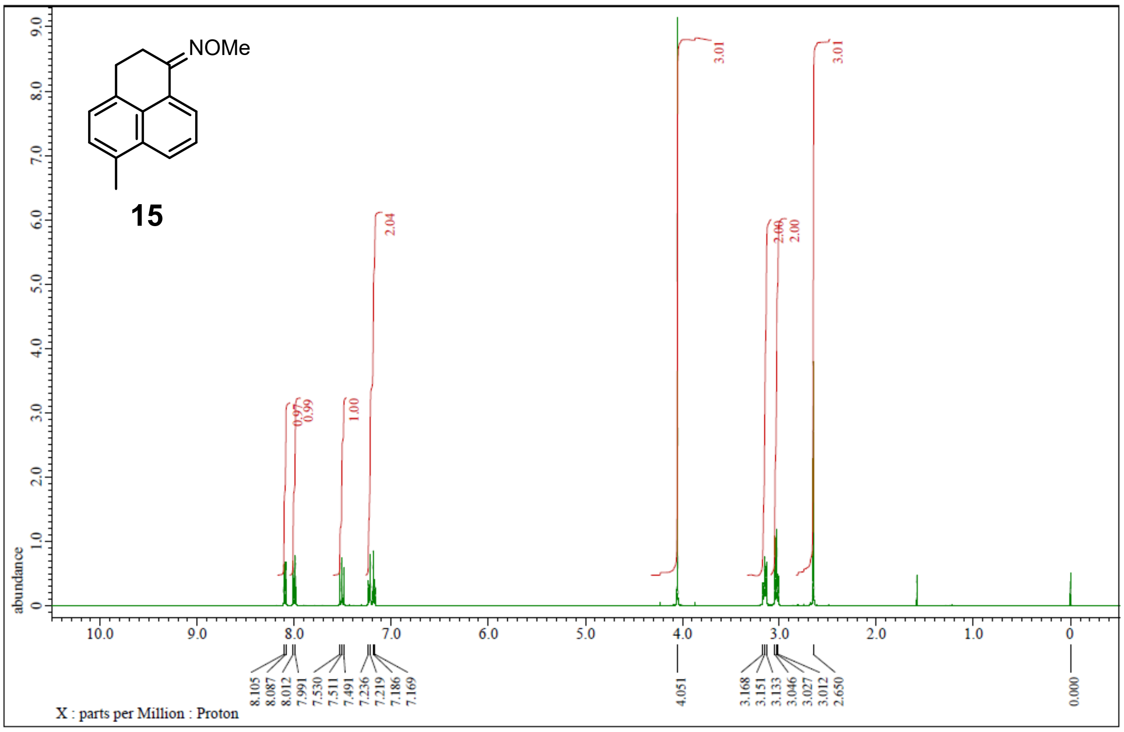

Supplementary Figure 35: ^{1}H NMR spectrum of 15 at room temperature ($CDCl_3$, 400 MHz)

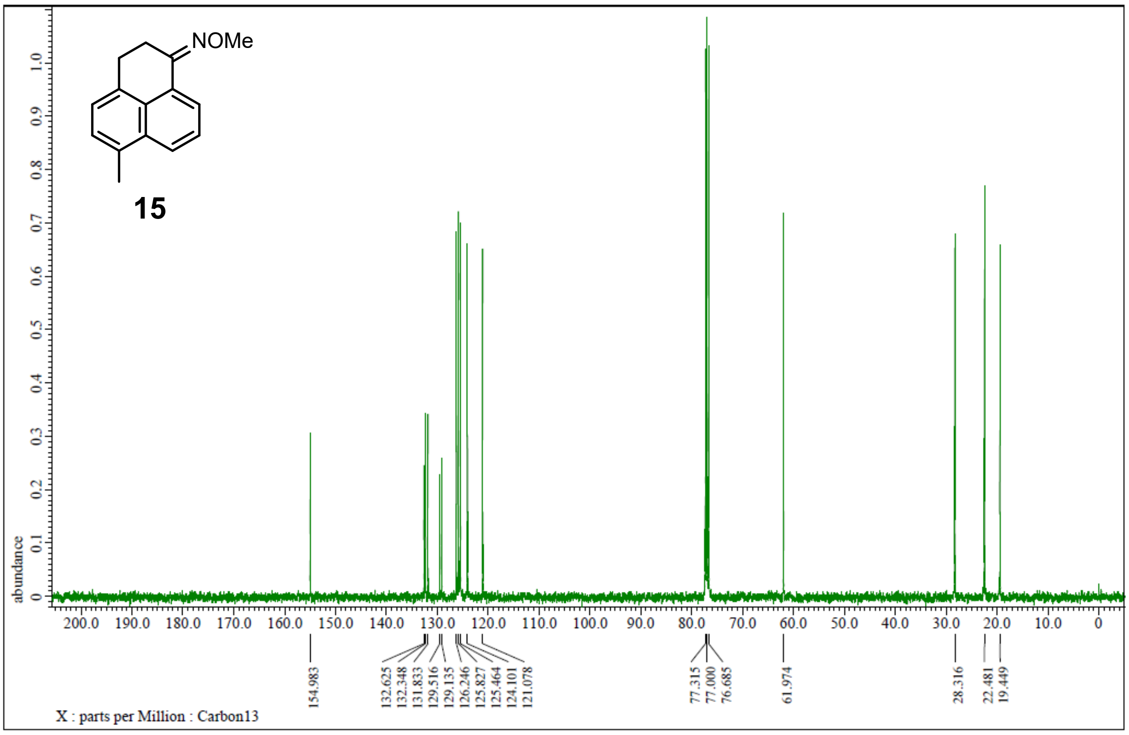

Supplementary Figure 36: ^{13}C NMR spectrum of 15 at room temperature ($CDCl_3$, 101 MHz)

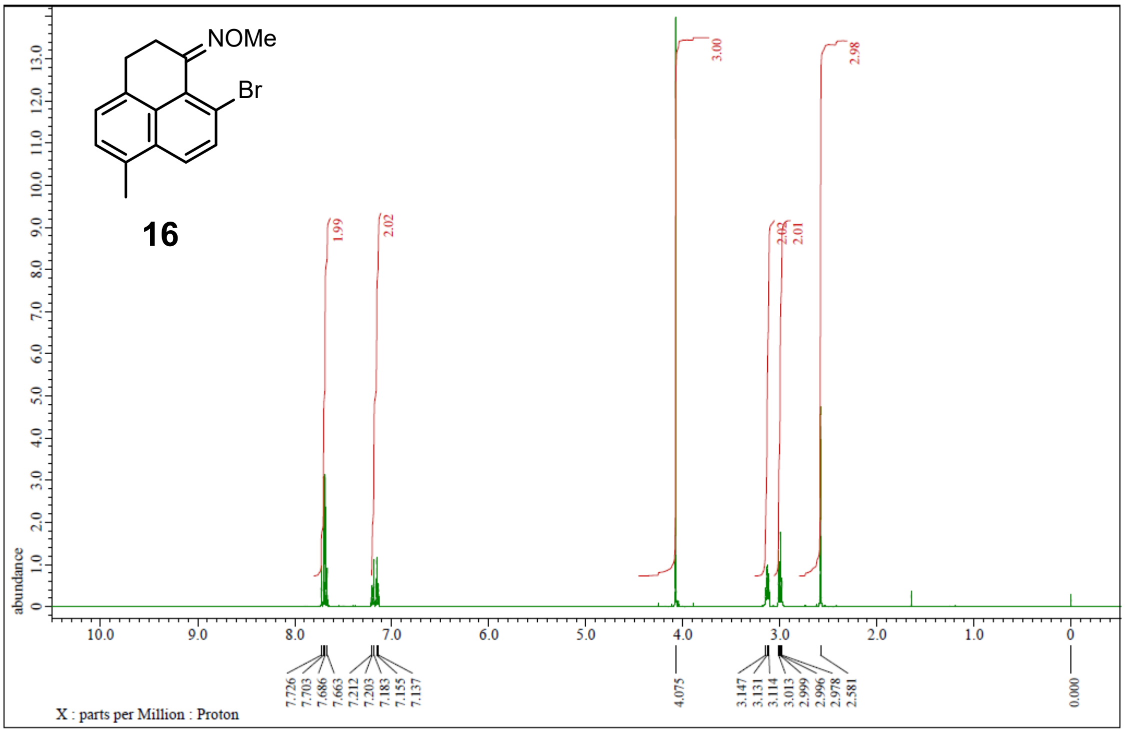

Supplementary Figure 37: ^{1}H NMR spectrum of 16 at room temperature ($CDCl_3$, 400 MHz)

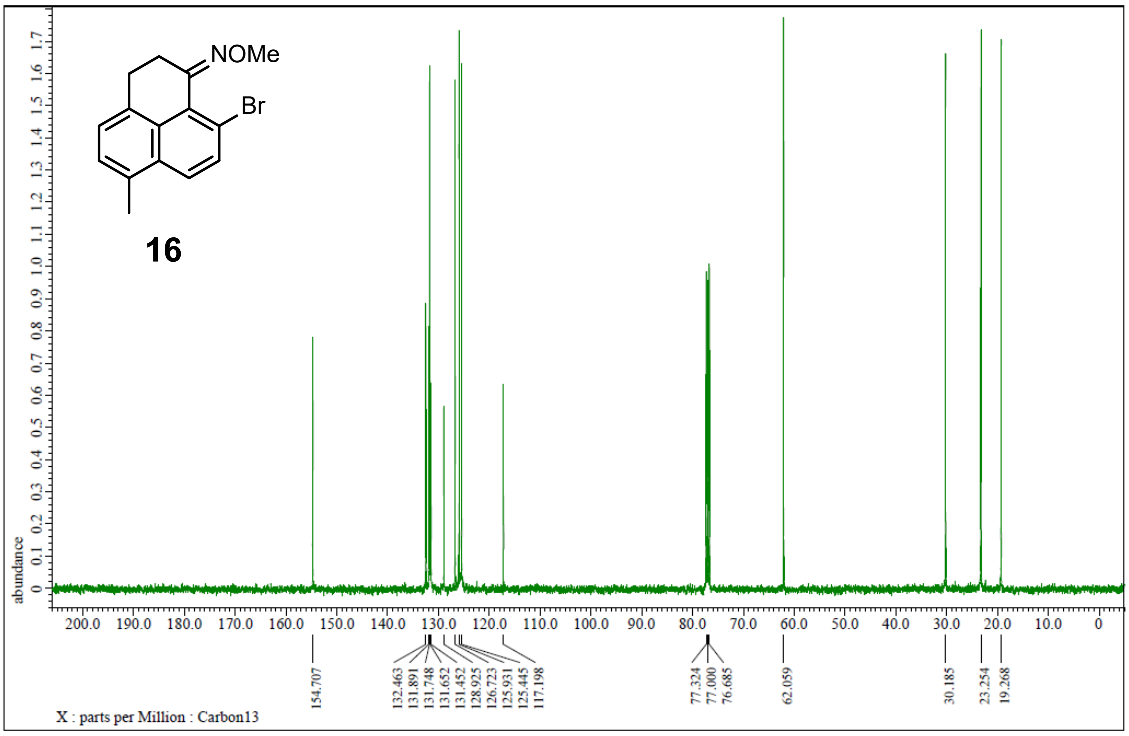

Supplementary Figure 38: ^{13}C NMR spectrum of 16 at room temperature ($CDCl_3$, 101 MHz)

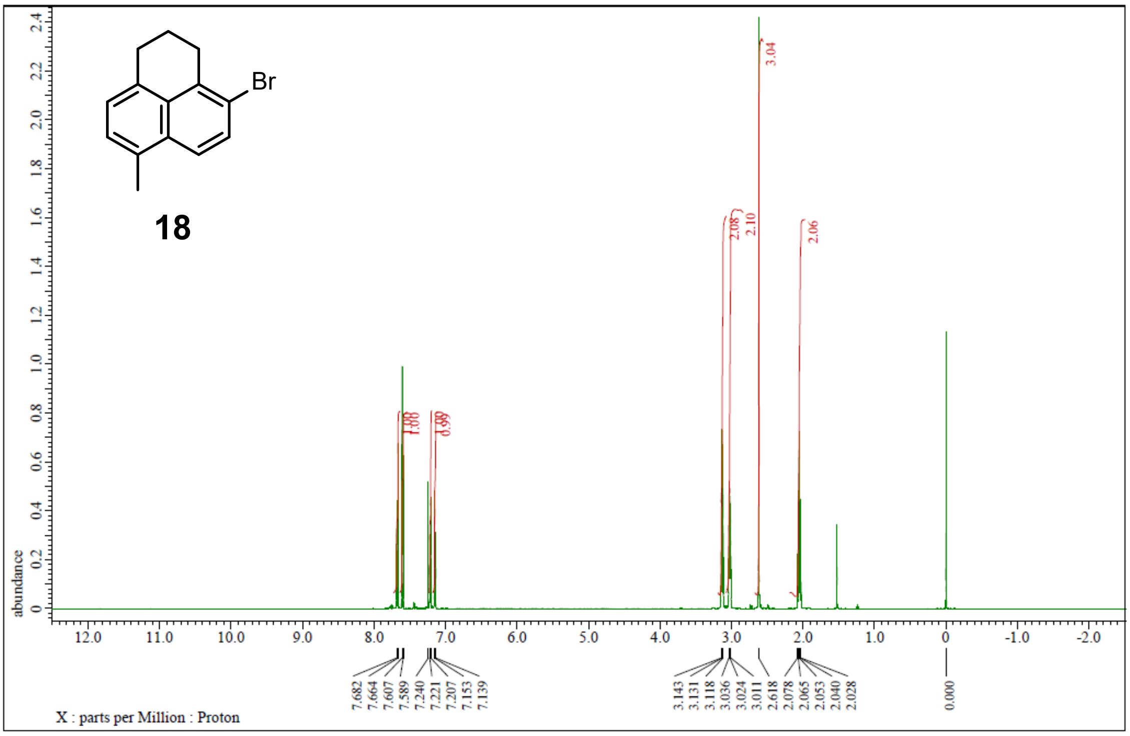

Supplementary Figure 39: **^{1}H NMR spectrum of 18** at room temperature ($CDCl_3$, 500 MHz)

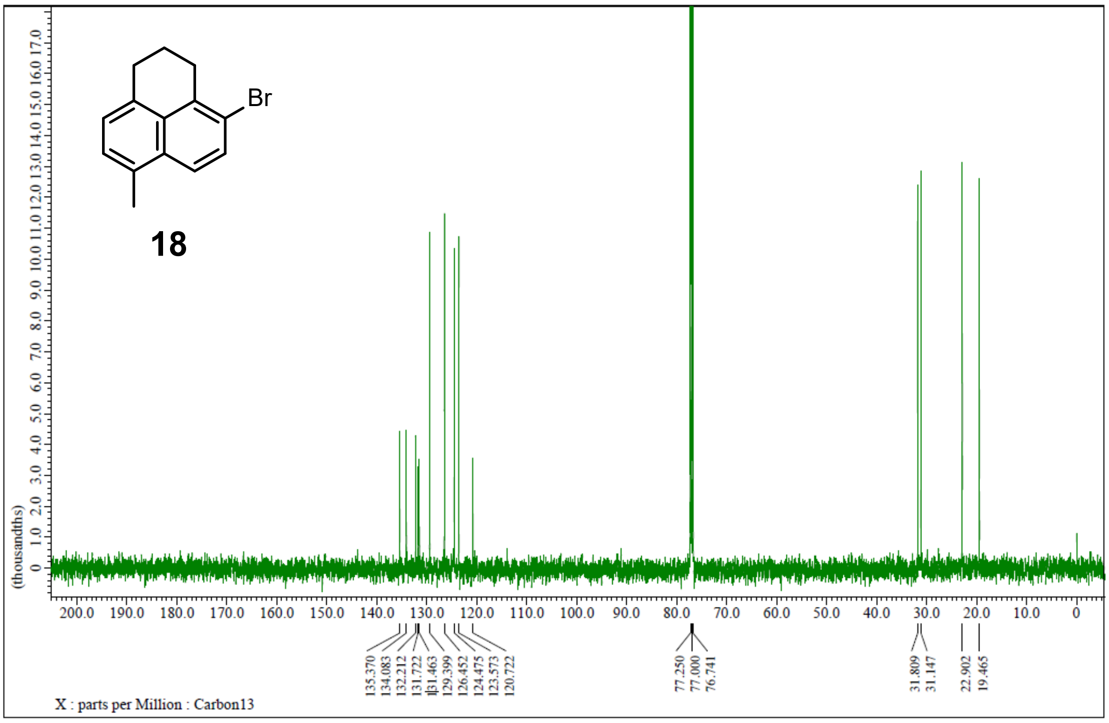

Supplementary Figure 40: ^{13}C NMR spectrum of 18 at room temperature ($CDCl_3$, 126 MHz)

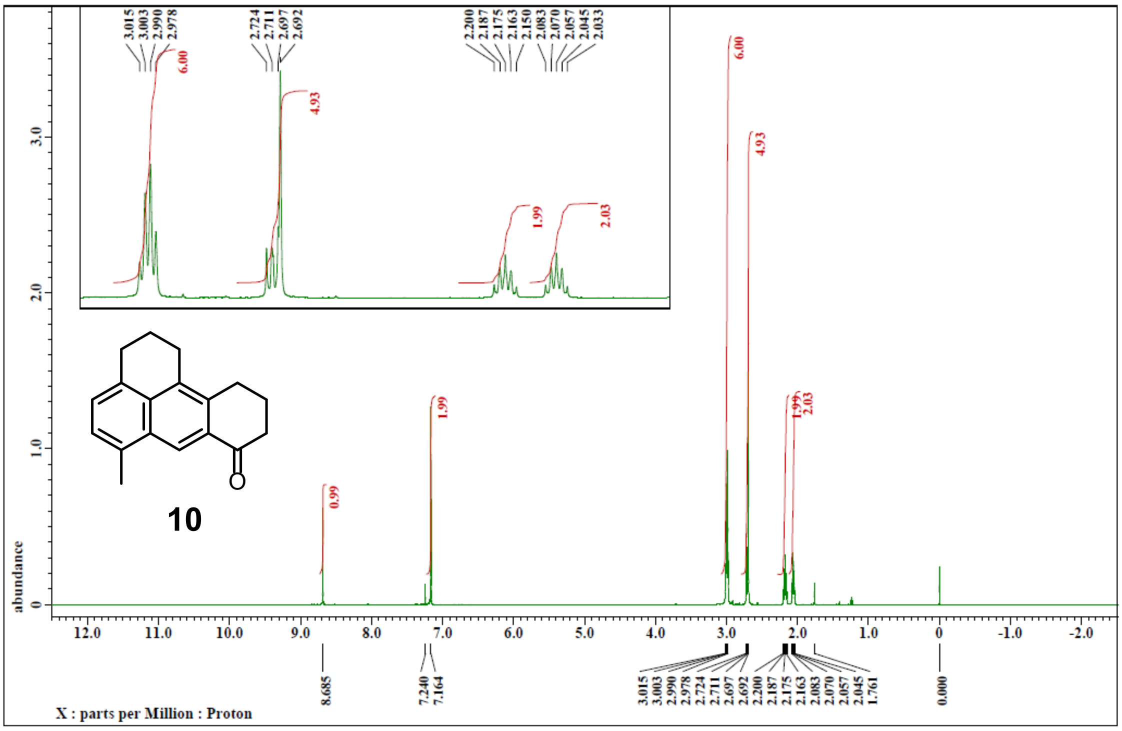

Supplementary Figure 41: ^{1}H NMR spectrum of 10 at room temperature ($CDCl_3$, 500 MHz)

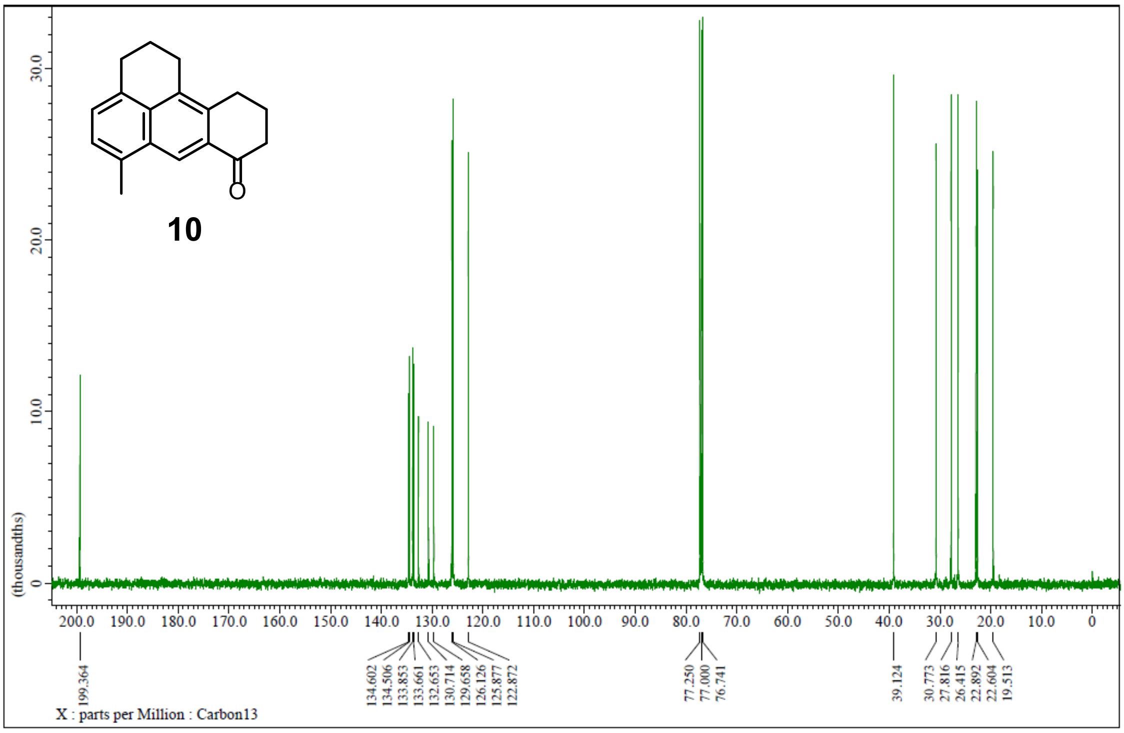

Supplementary Figure 42: ^{13}C NMR spectrum of 10 at room temperature ($CDCl_3$, 126 MHz)

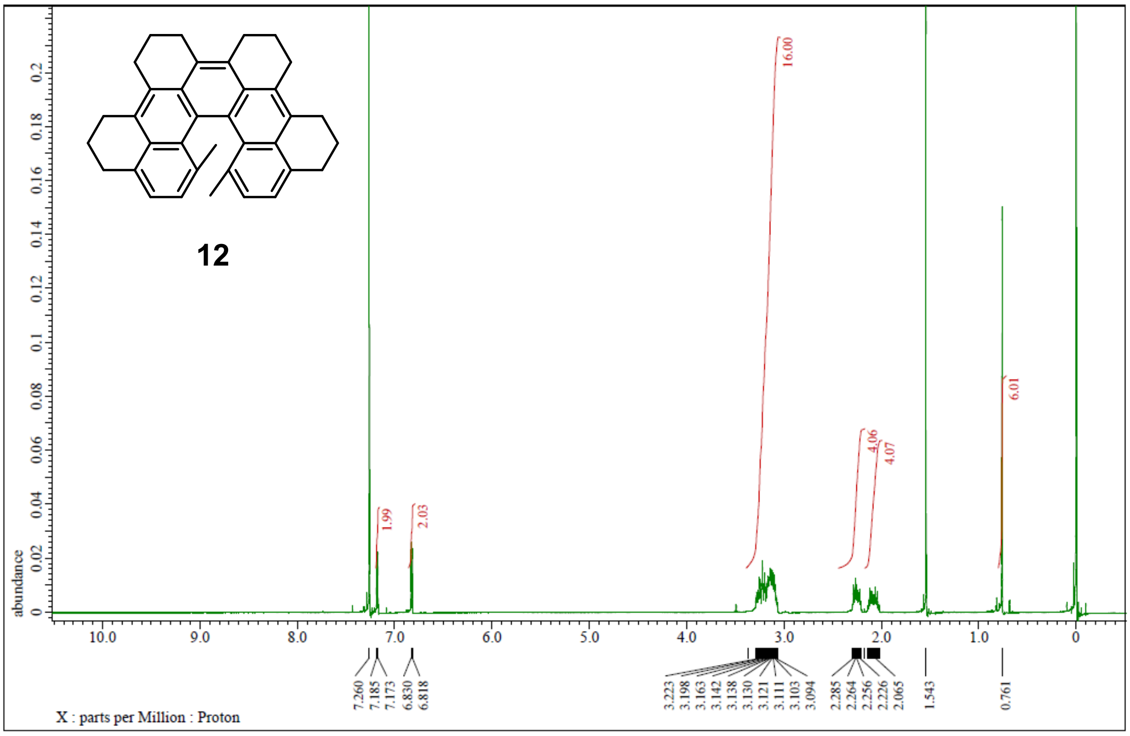

Supplementary Figure 43: ^{1}H NMR spectrum of 12 at room temperature ($CDCl_3$, 600 MHz)

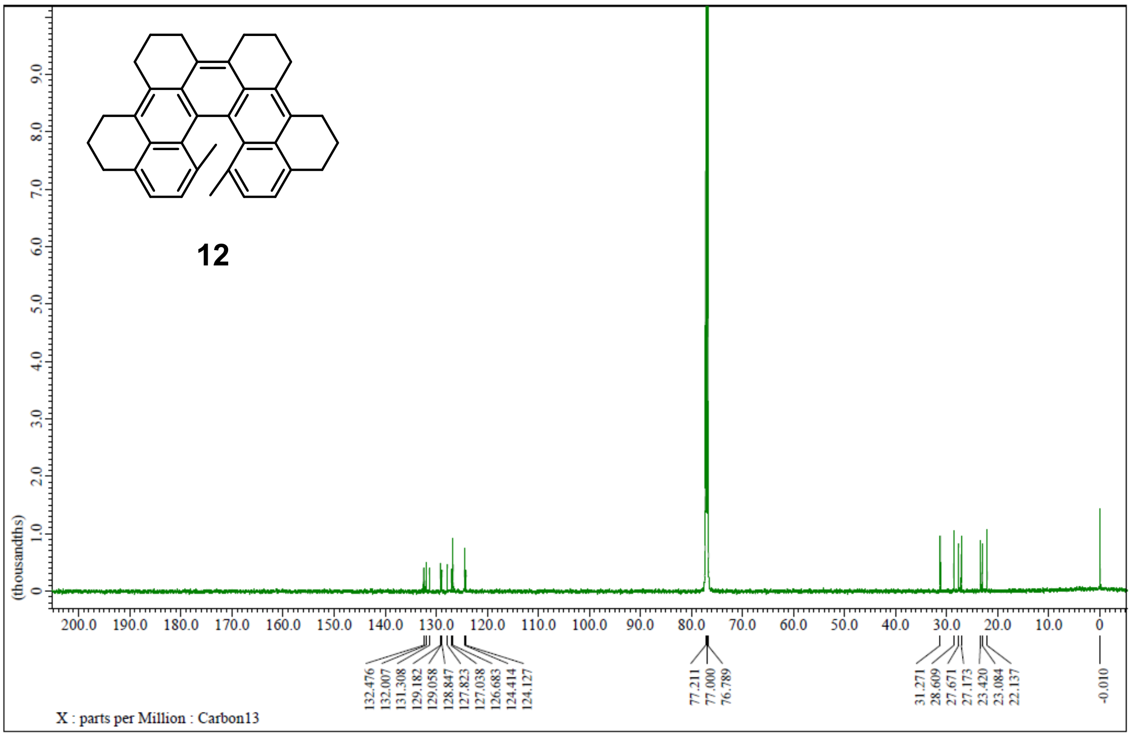

Supplementary Figure 44: ^{13}C NMR spectrum of 12 at room temperature ($CDCl_3$, 151 MHz)

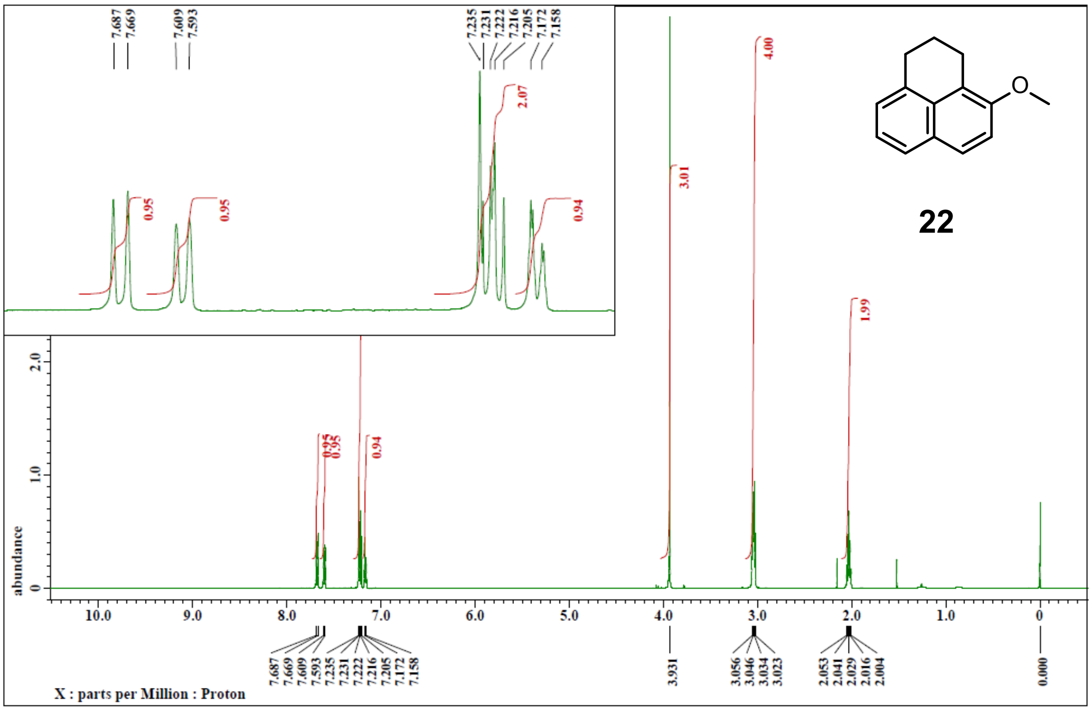

Supplementary Figure 45: ^{1}H NMR spectrum of 22 at room temperature ($CDCl_3$, 500 MHz)

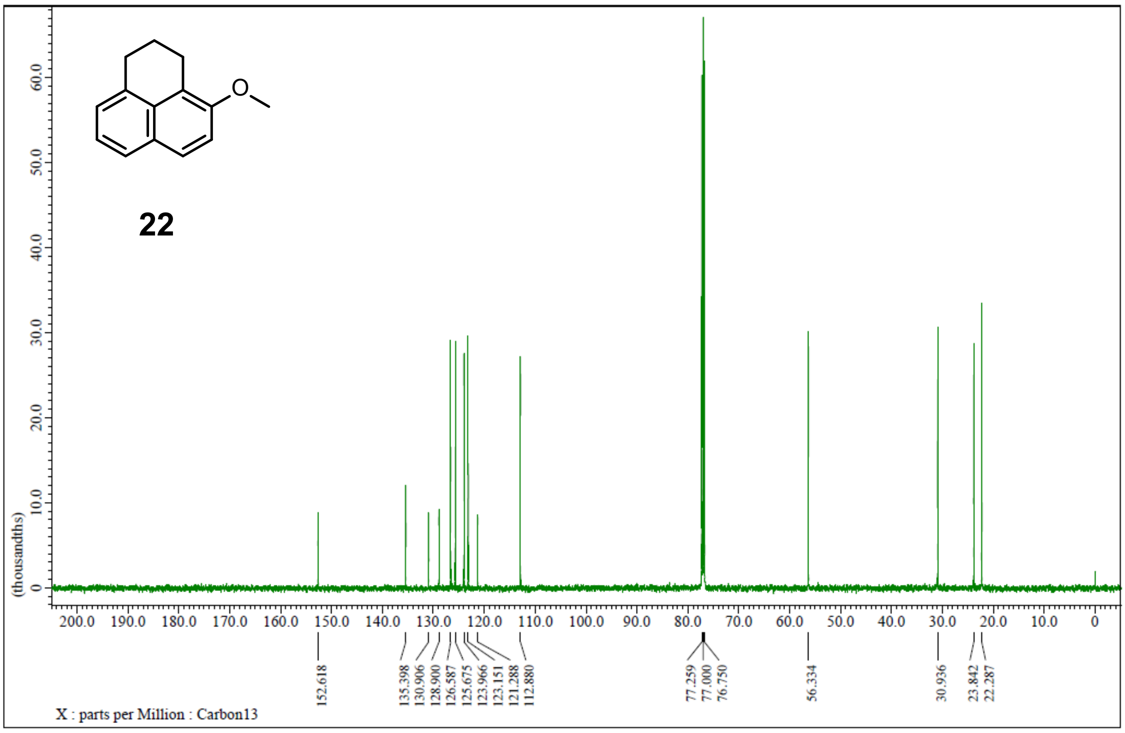

Supplementary Figure 46: ^{13}C NMR spectrum of 22 at room temperature ($CDCl_3$, 126 MHz)

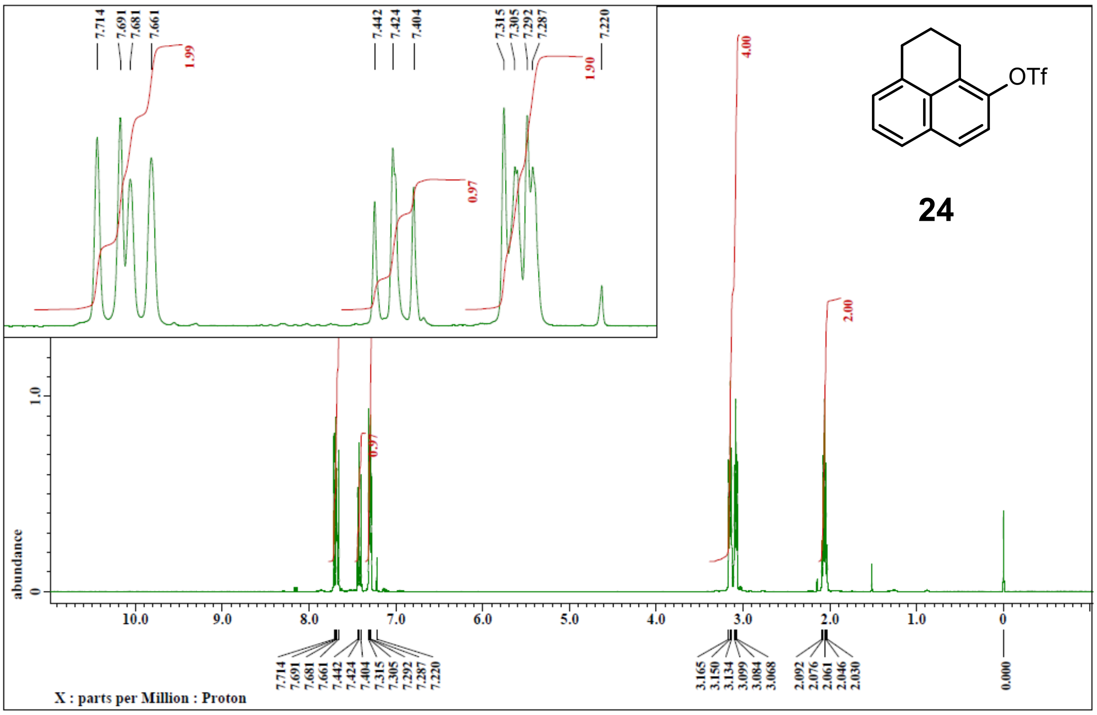

Supplementary Figure 47: ^{1}H NMR spectrum of 24 at room temperature ($CDCl_3$, 400 MHz)

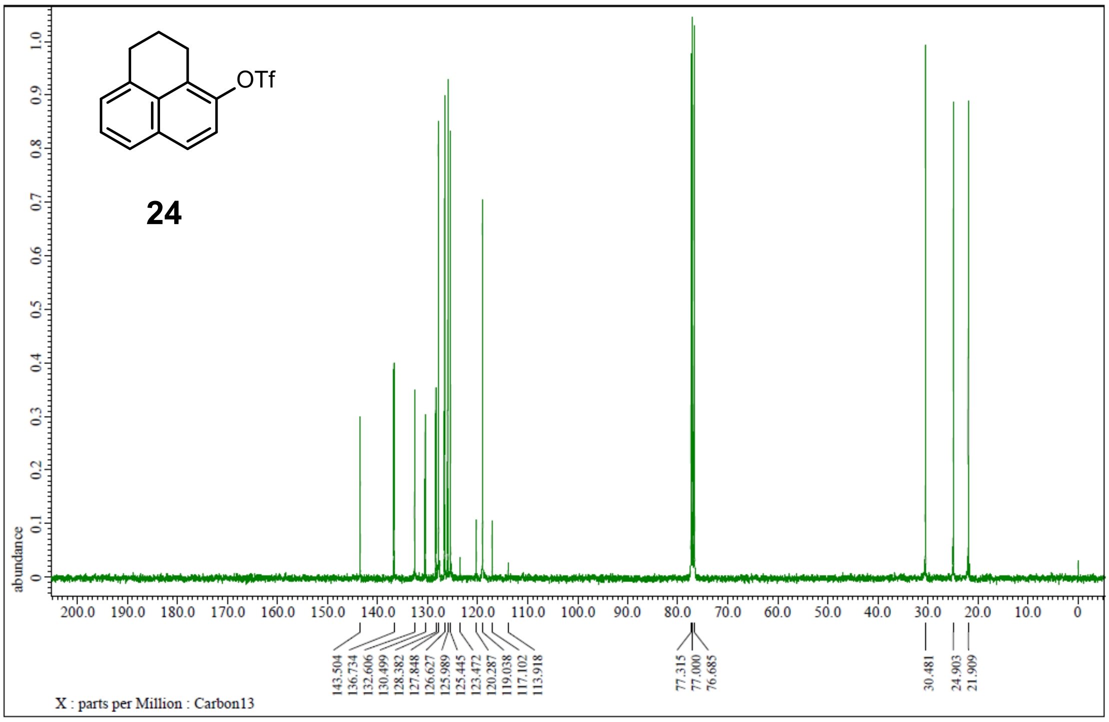

Supplementary Figure 48: ^{13}C NMR spectrum of 24 at room temperature ($CDCl_3$, 101 MHz)

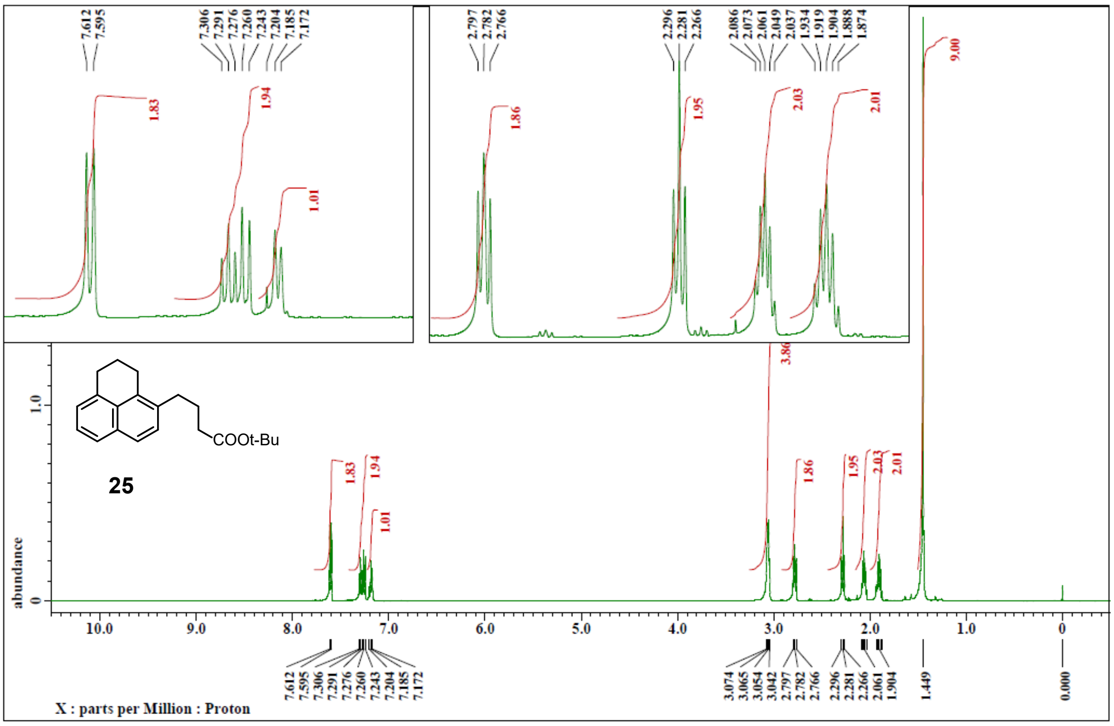

Supplementary Figure 49: ^{1}H NMR spectrum of 25 at room temperature ($CDCl_3$, 500 MHz)

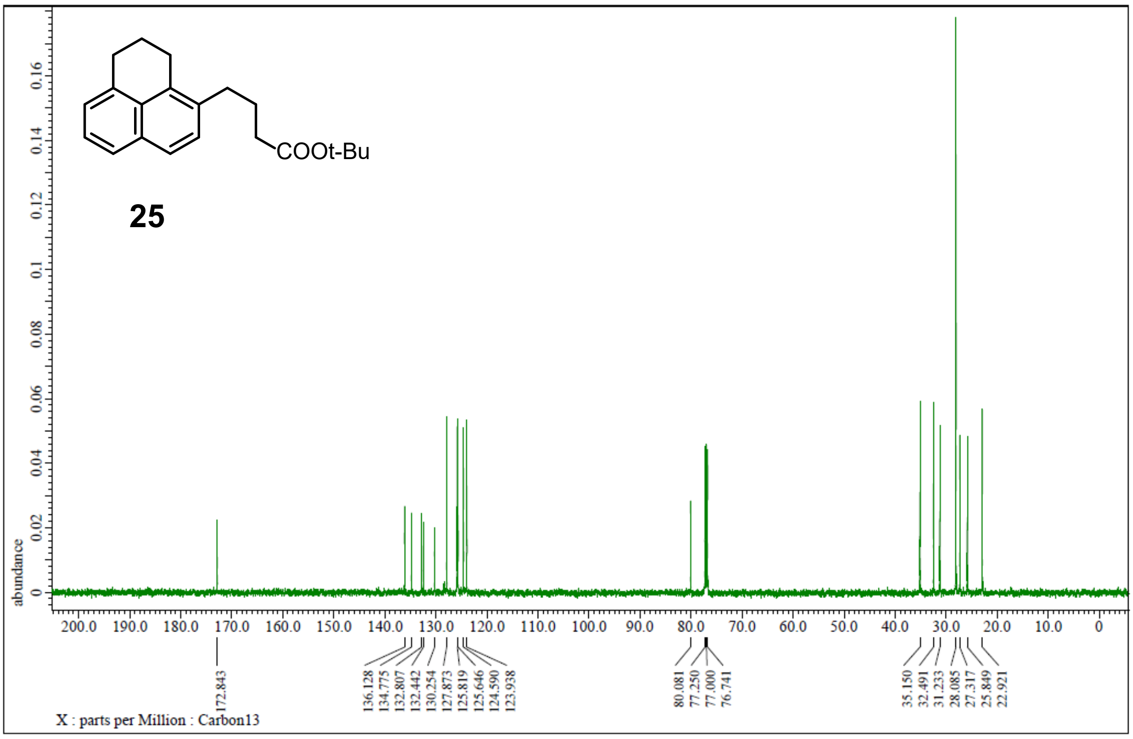

Supplementary Figure 50: ^{13}C NMR spectrum of 25 at room temperature ($CDCl_3$, 126 MHz)

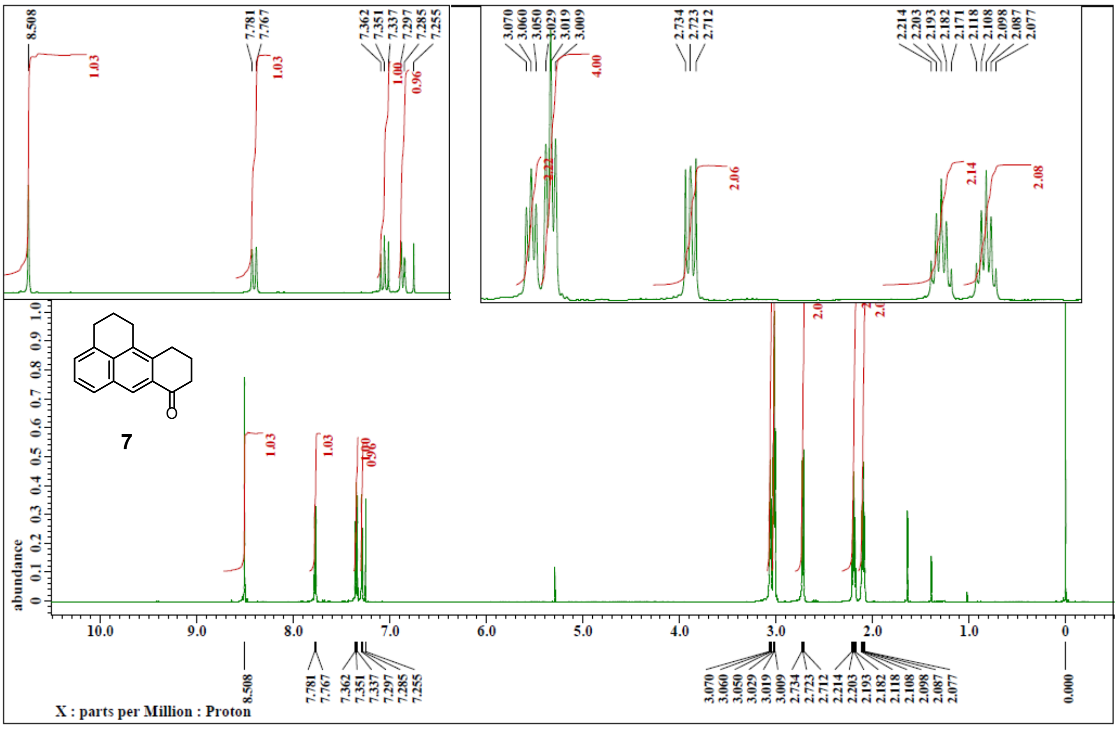

Supplementary Figure 51: ^{1}H NMR spectrum of 7 at room temperature ($CDCl_3$, 600 MHz)

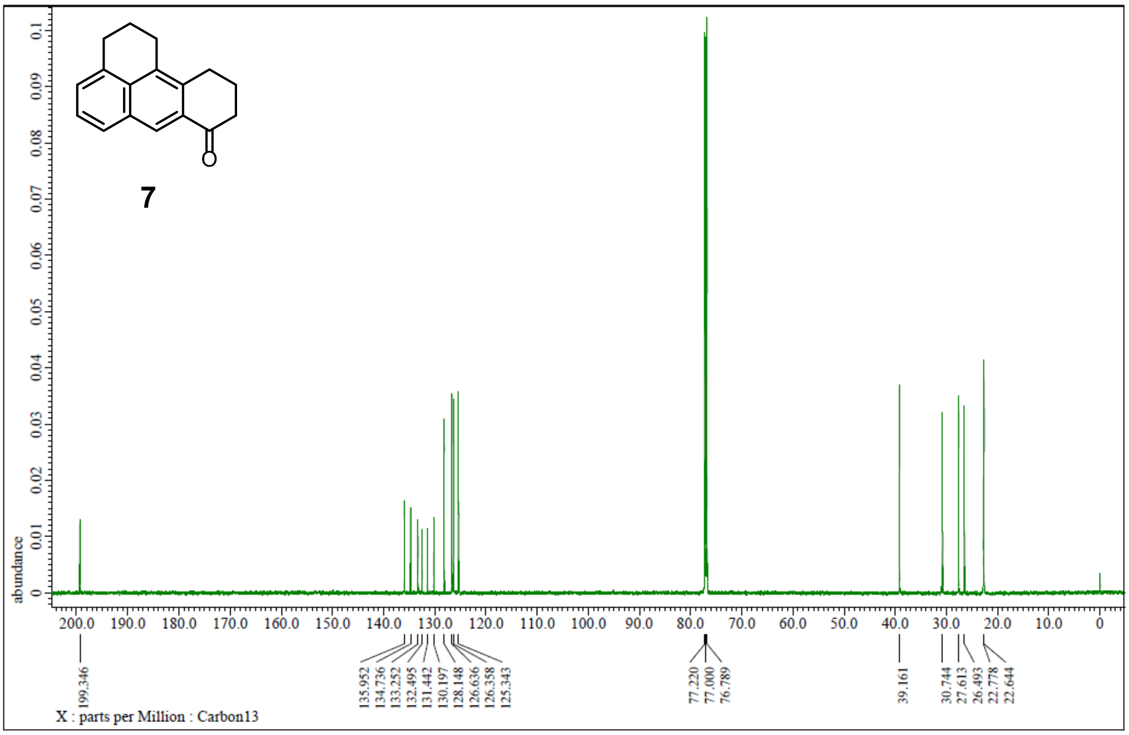

Supplementary Figure 52: ^{13}C NMR spectrum of 7 at room temperature ($CDCl_3$, 151 MHz)

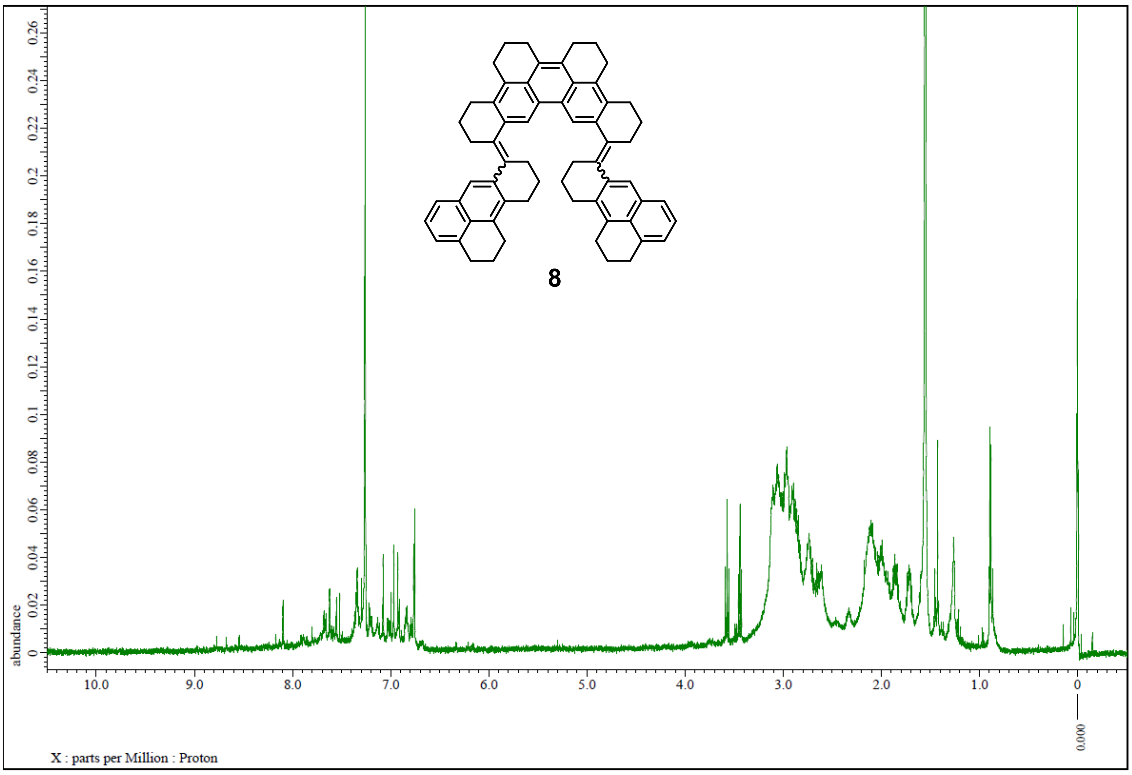

Supplementary Figure 53: ^{1}H NMR spectrum of 8 at room temperature ($CDCl_3$, 400 MHz)

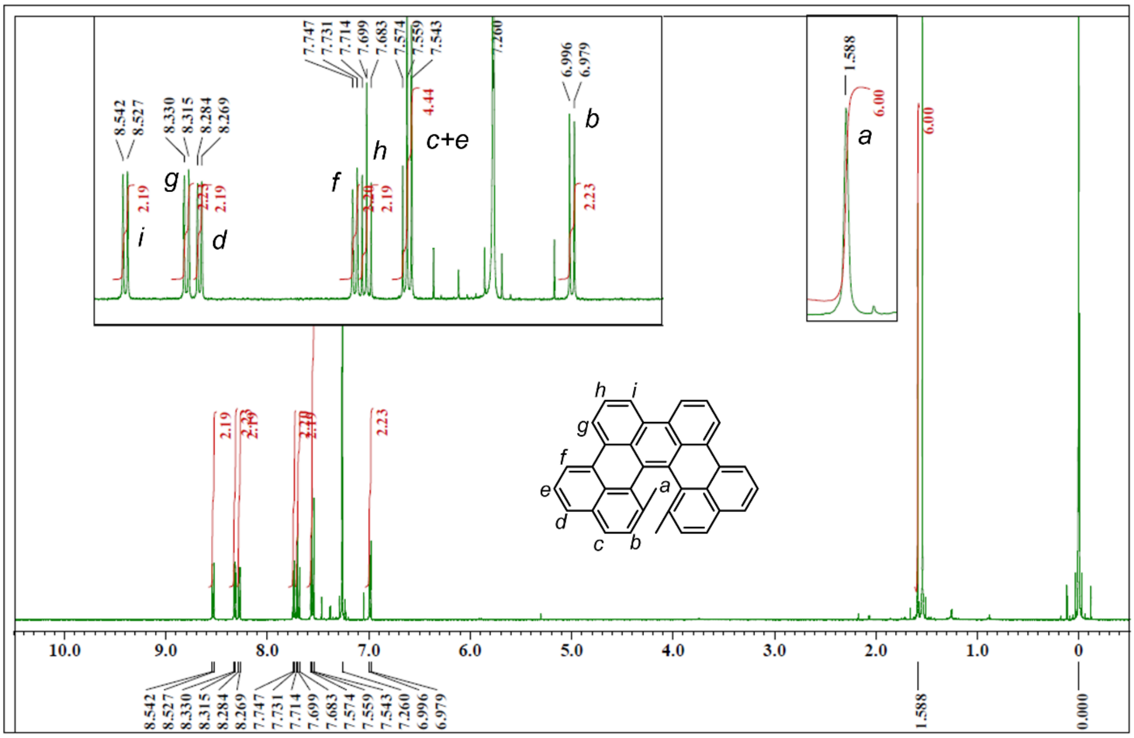

Supplementary Figure 54: ^{1}H NMR spectrum of 1 at room temperature ($CDCl_3$, 500 MHz)

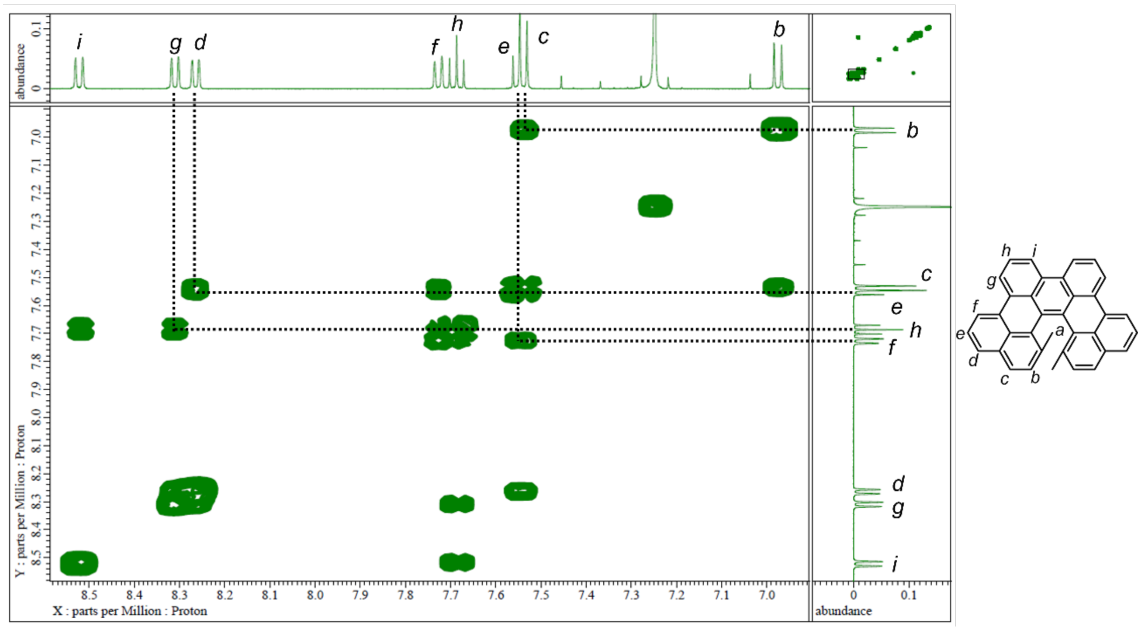

Supplementary Figure 55: ^{1}H-^{1}H COSY spectrum of 1 at room temperature ($CDCl_3$, 600 MHz)

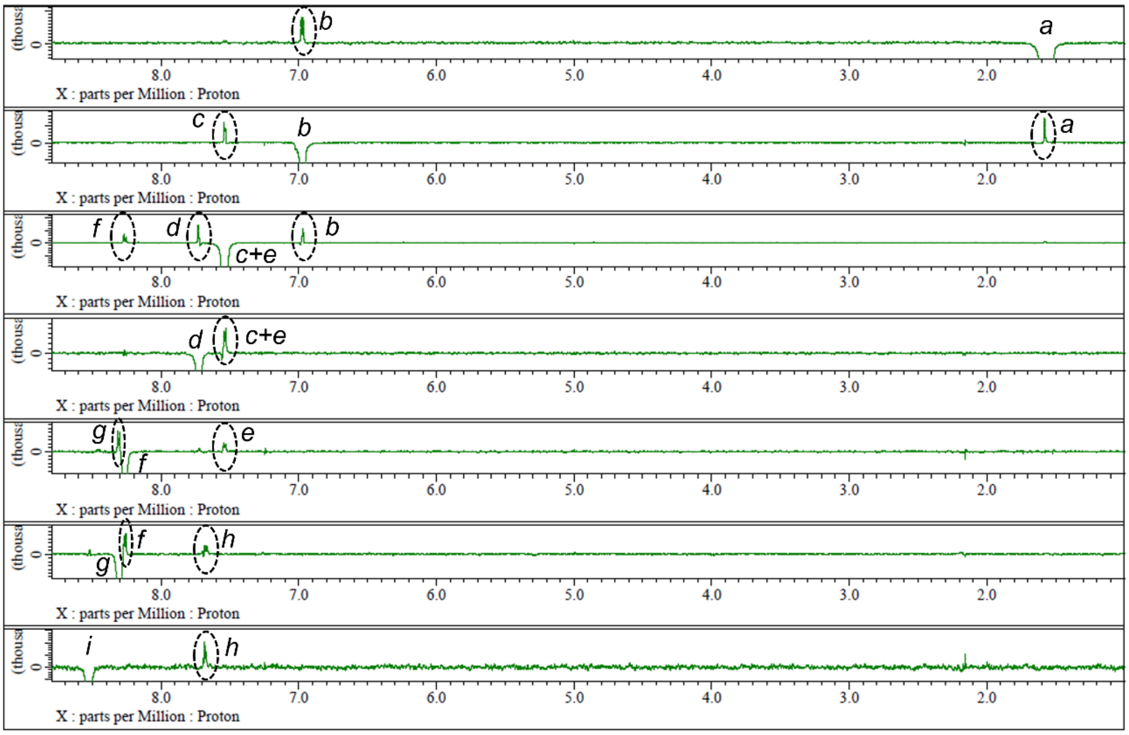

Supplementary Figure 56: NOE difference spectra of 1 at room temperature ($CDCl_3$, 600 MHz)

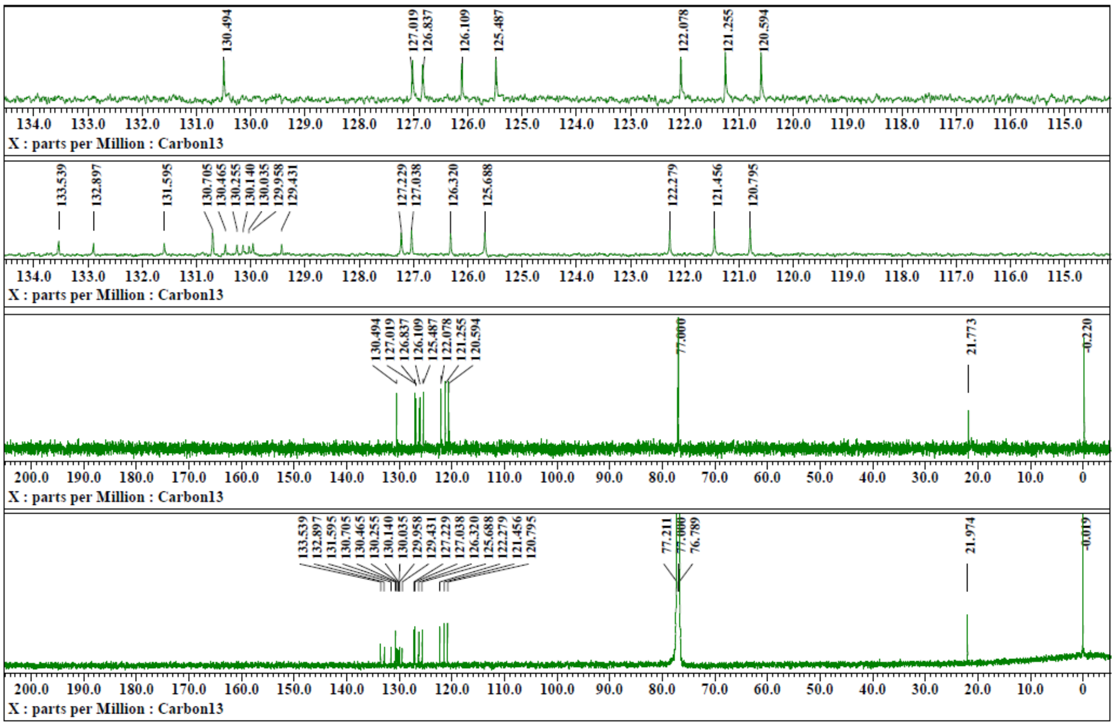

Supplementary Figure 57: ^{13}C NMR spectrum of 1 at room temperature ($CDCl_3$, 151 MHz)

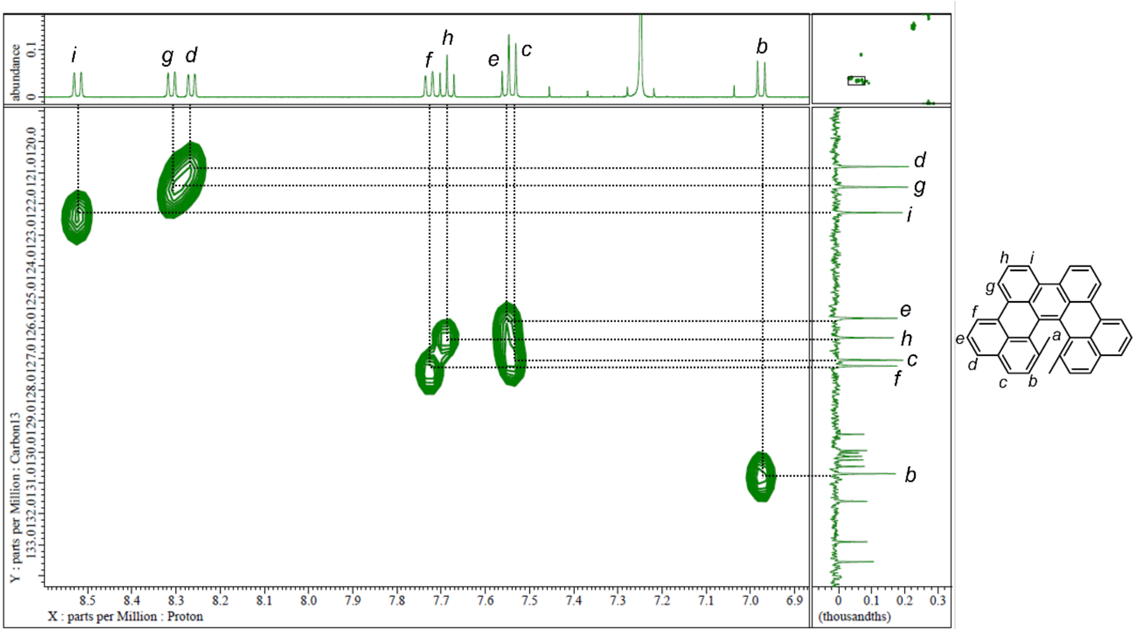

Supplementary Figure 58: $^{13}C/^{1}H$ HMQC spectra of 1 at room temperature ($CDCl_3$, 151 MHz)

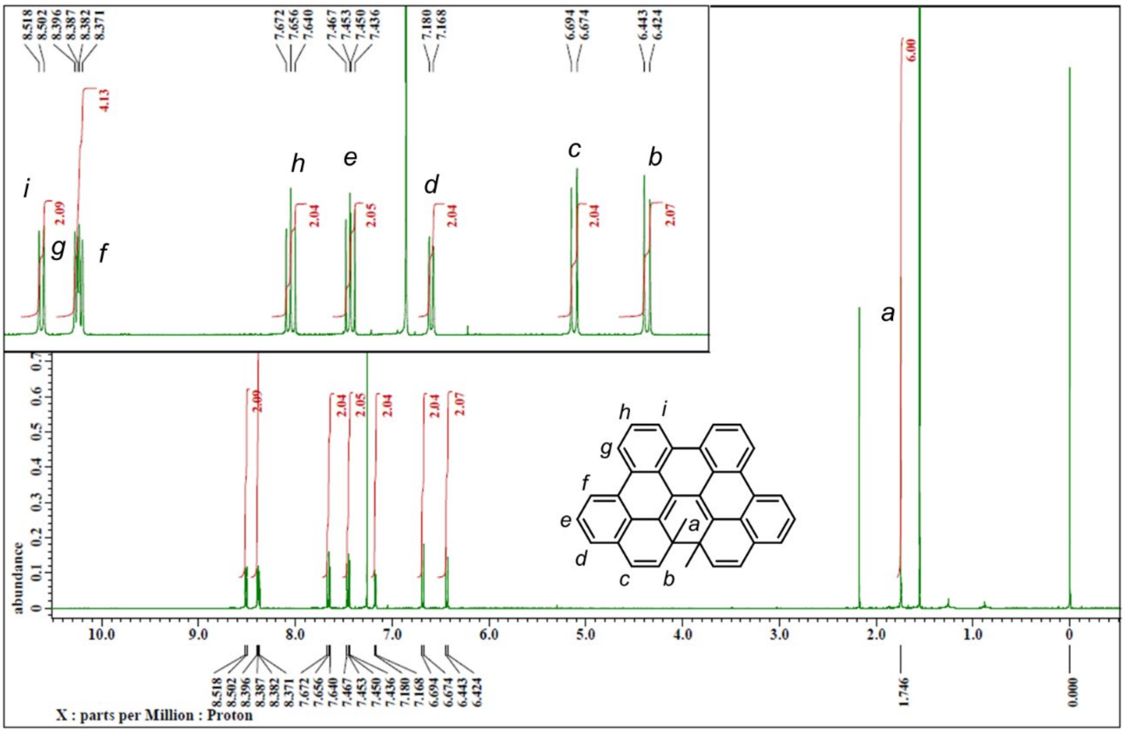

Supplementary Figure 59: ^{1}H NMR spectrum of 1c at room temperature ($CDCl_3$, 500 MHz)

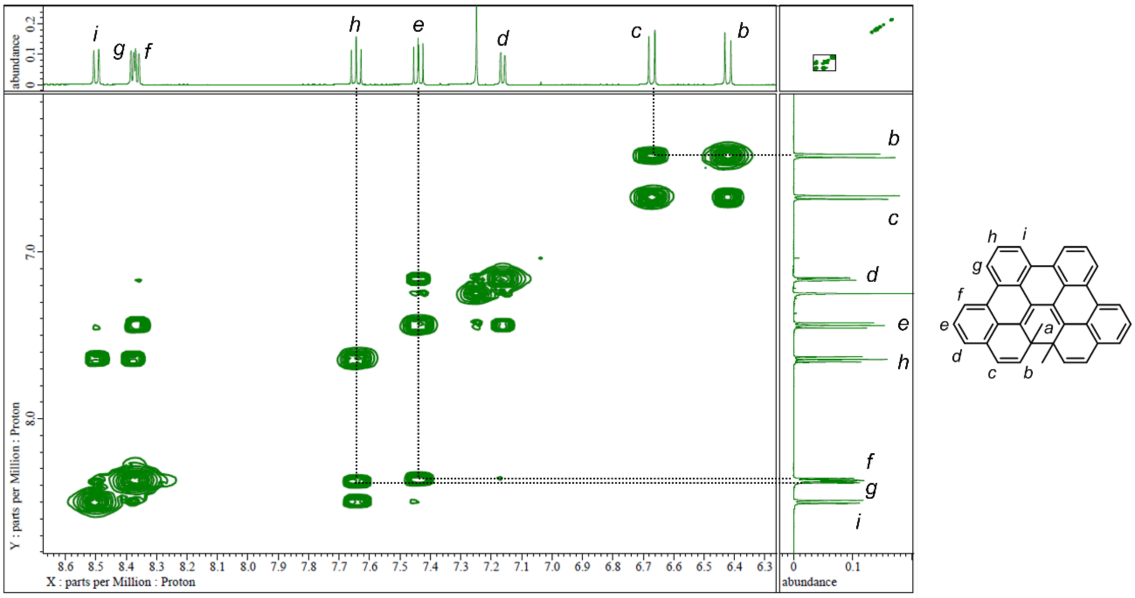

Supplementary Figure 60: ^{1}H-^{1}H COSY spectrum of 1c at room temperature ($CDCl_3$, 600 MHz)

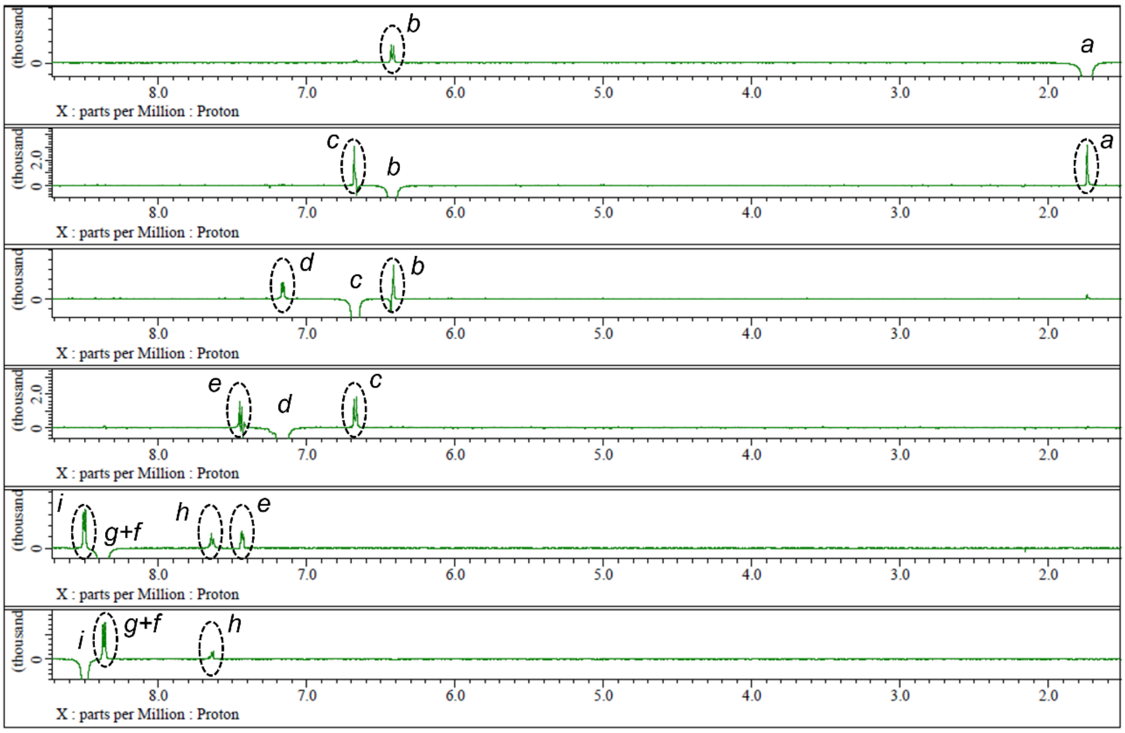

Supplementary Figure 61: NOE difference spectra of 1c at room temperature ($CDCl_3$, 600 MHz)

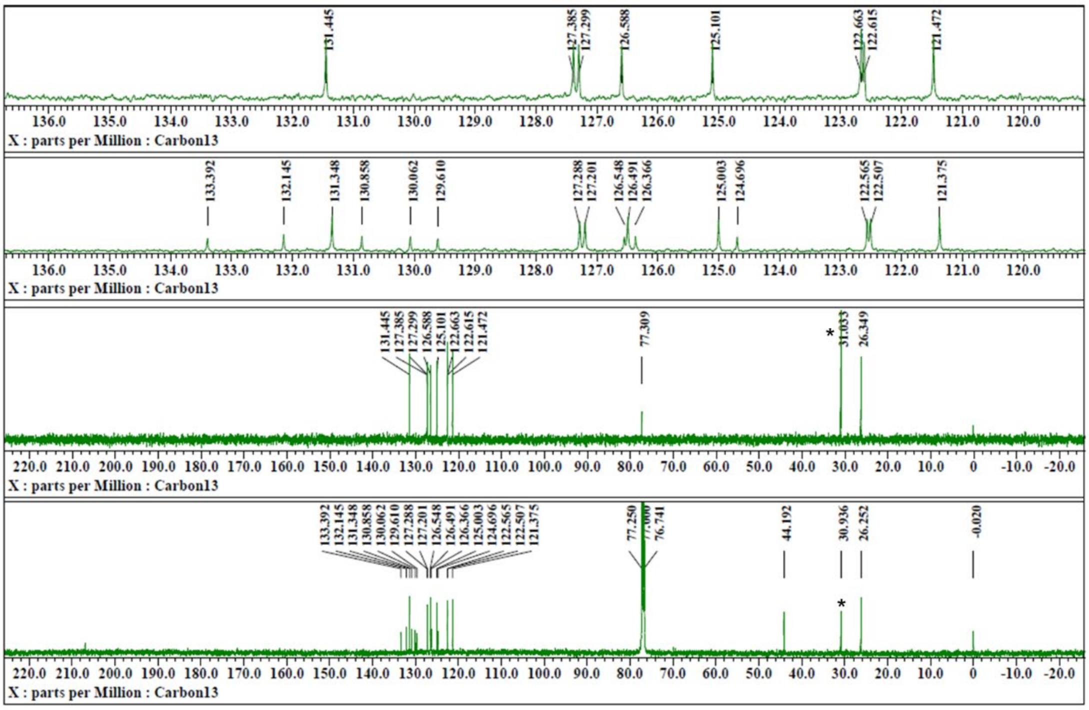

Supplementary Figure 62: ^{13}C NMR spectrum of 1c at room temperature ($CDCl_3$, 126 MHz). Peaks denoted by asterisk are due to residual acetone.

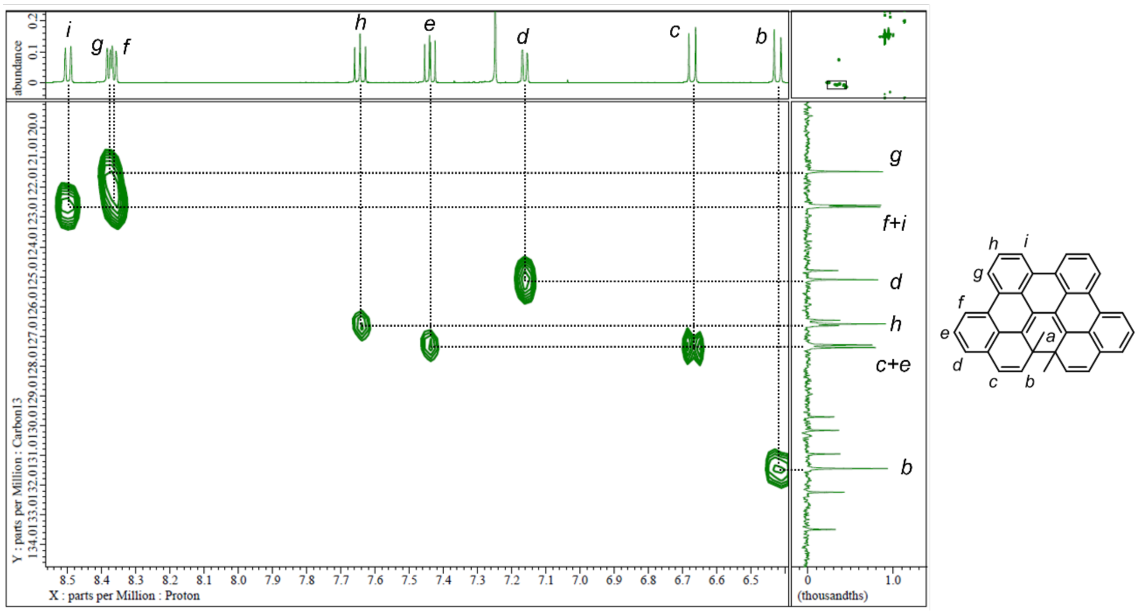

Supplementary Figure 63: $^{13}C/^{1}H$ HMQC spectra of 1c at room temperature ($CDCl_3$, 151 MHz)

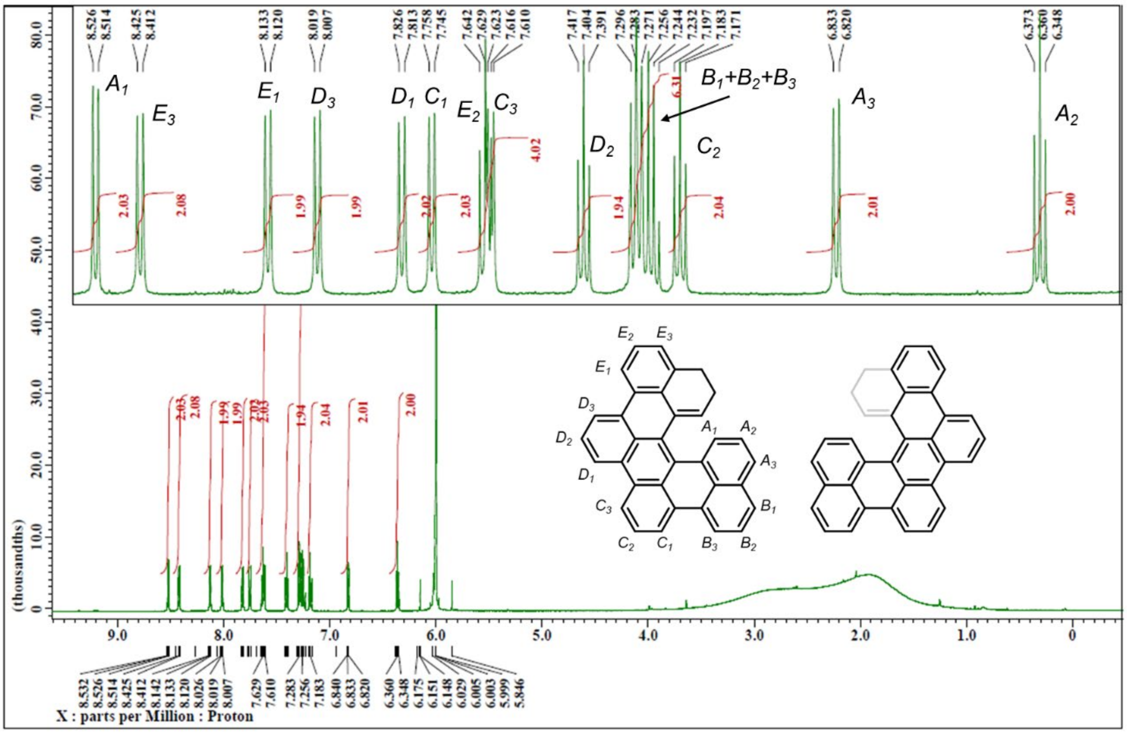

Supplementary Figure 64: ^{1}H NMR spectrum of 3 at room temperature (600 MHz, $C_2D_2Cl_4$ with a small excess amount of $N_2H_4 \cdot H_2O$)

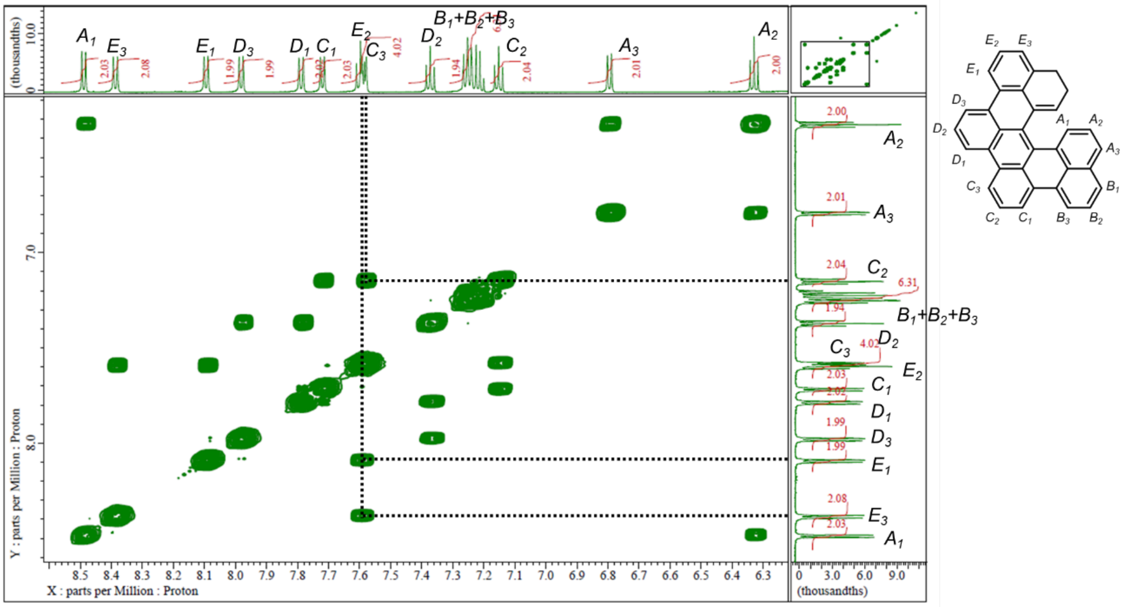

Supplementary Figure 65: ^{1}H-^{1}H COSY spectrum of 3 at room temperature (600 MHz, $C_2D_2Cl_4$ with a small excess amount of $N_2H_4 \cdot H_2O$)

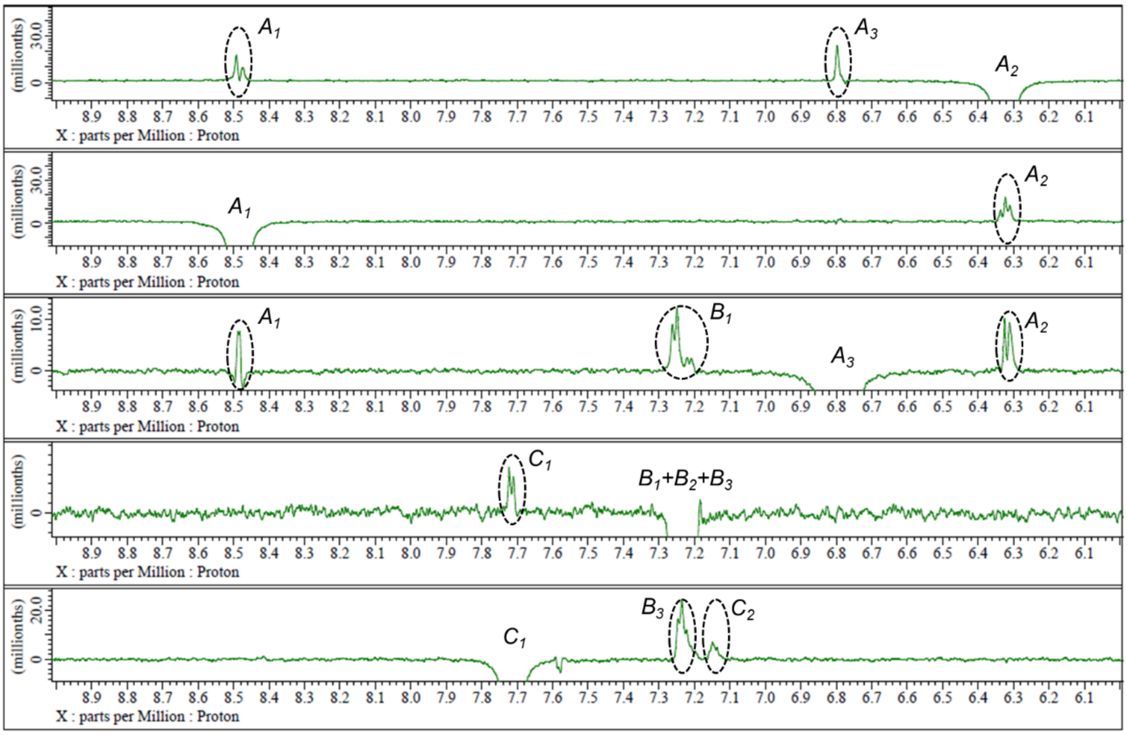
A1
A3
A2
A1
A2
A1
B1
A3
A2
C1
B1+B2+B3
B3
C2
C1
(millionths)
X : parts per Million : Proton

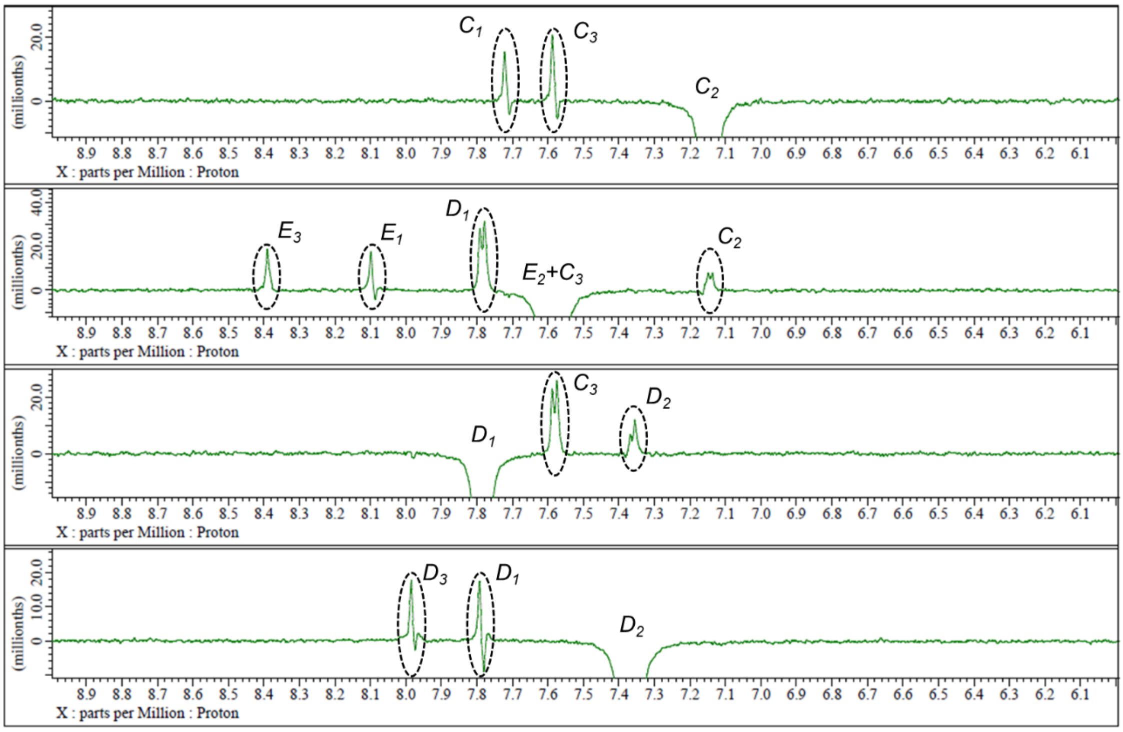
C1
C3
C2
E3
E1
D1
E2+C3
C2
C3
D2
D1
D3
D1
D2
(millionths)
X : parts per Million : Proton

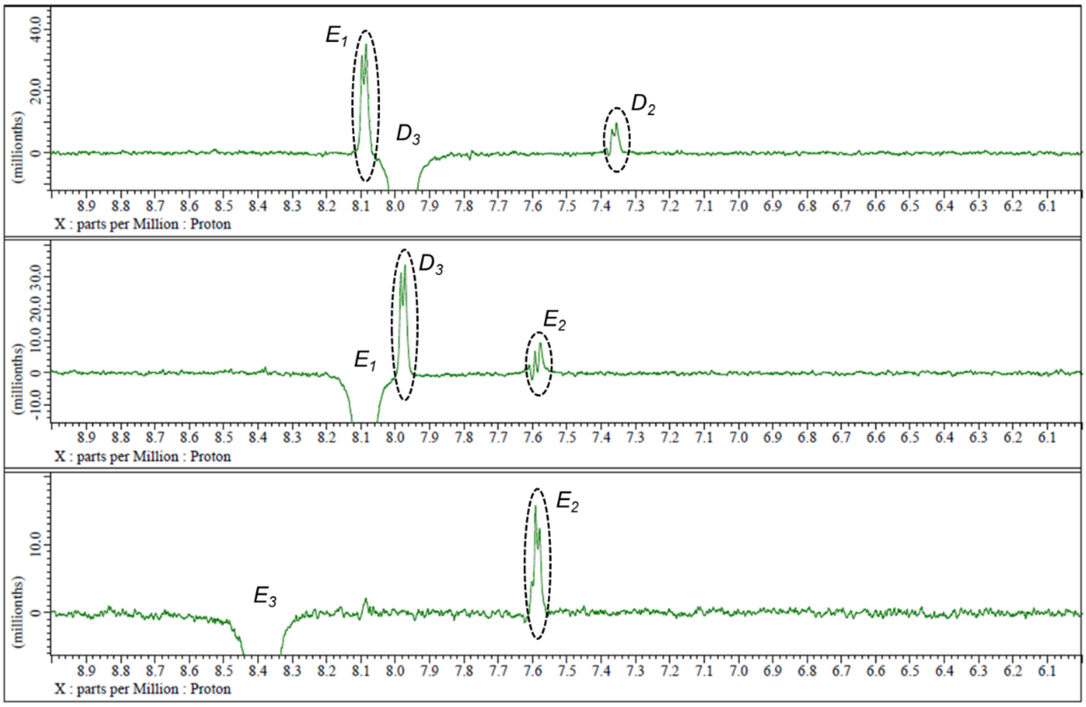

Supplementary Figure 66: NOE difference spectra of 3 at room temperature (600 MHz, $C_2D_2Cl_4$ + small excess amount of $N_2H_4{\cdot}H_2O$)

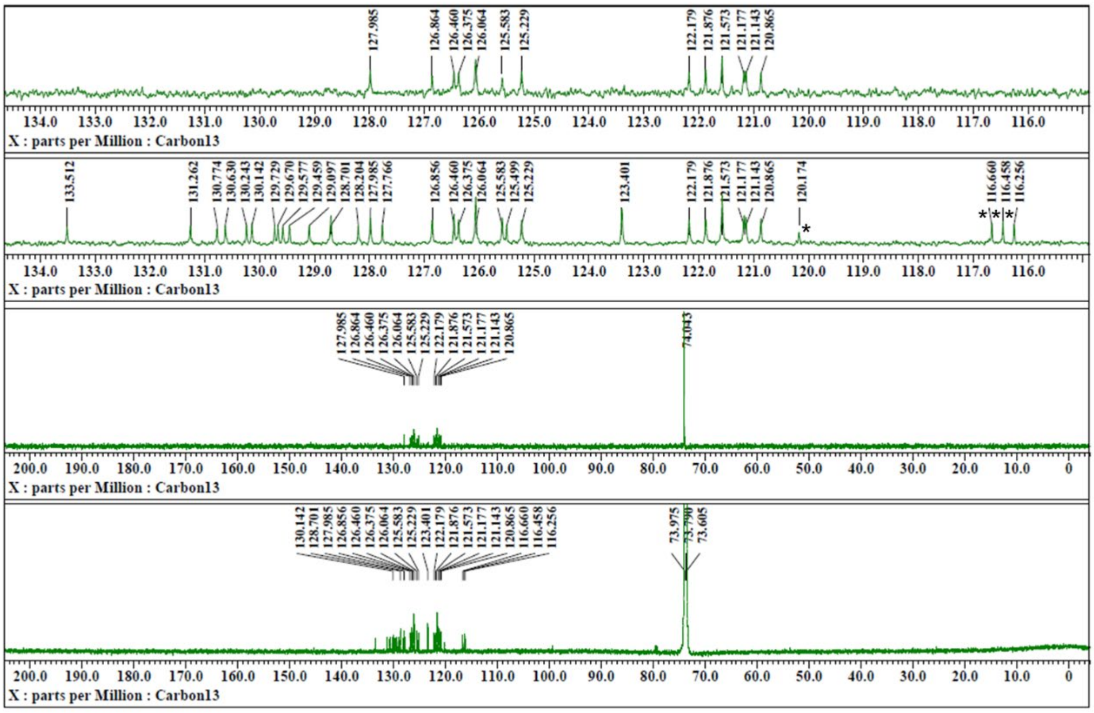

Supplementary Figure 67: ^{13}C NMR spectrum of 3 at room temperature (151 MHz, $C_2D_2Cl_4$ with a small excess amount of $N_2H_4{\cdot}H_2O$). Peaks denoted by asterisk are due to deuterated trichloroethylene derived from $C_2D_2Cl_4$.

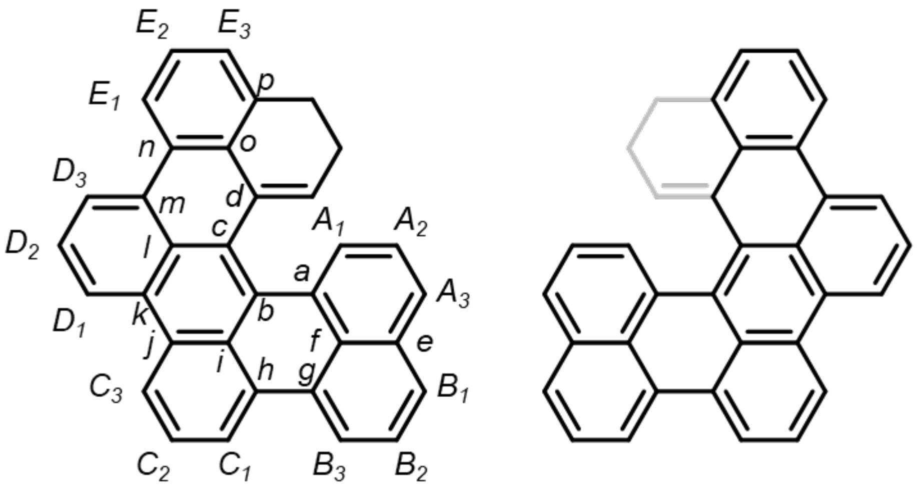

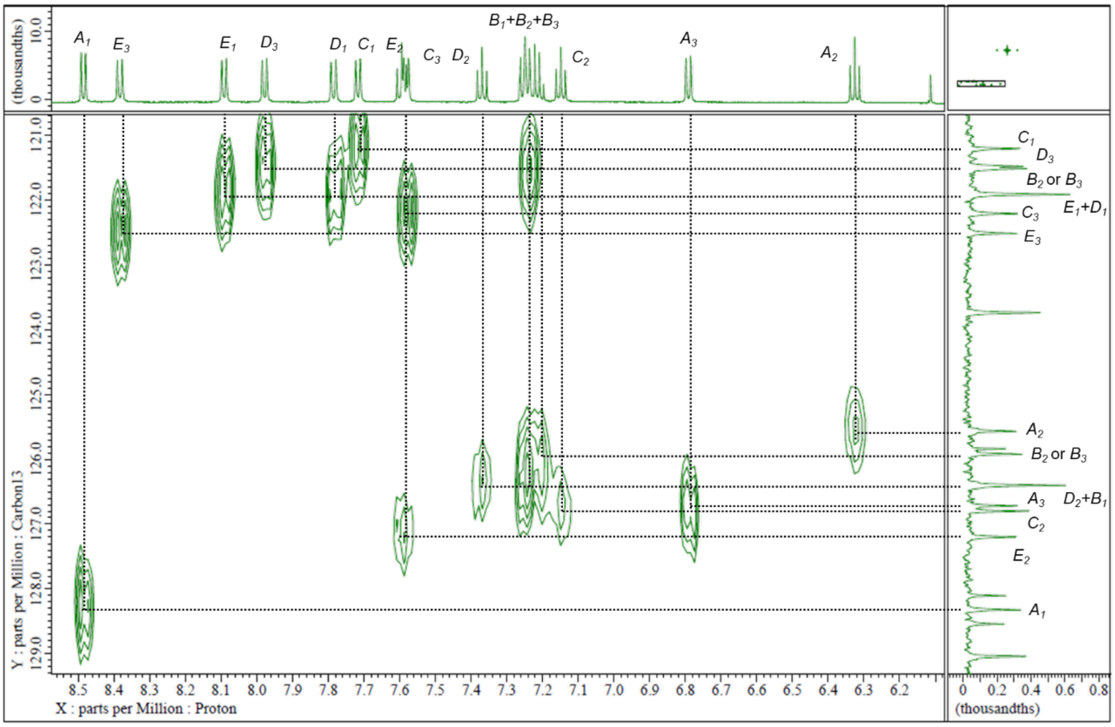

Supplementary Figure 68: $^{13}C/^{1}H$ HMQC spectra of 3 at room temperature (151 MHz, $C_2D_2Cl_4$ with a small excess amount of $N_2H_4\cdot H_2O$)

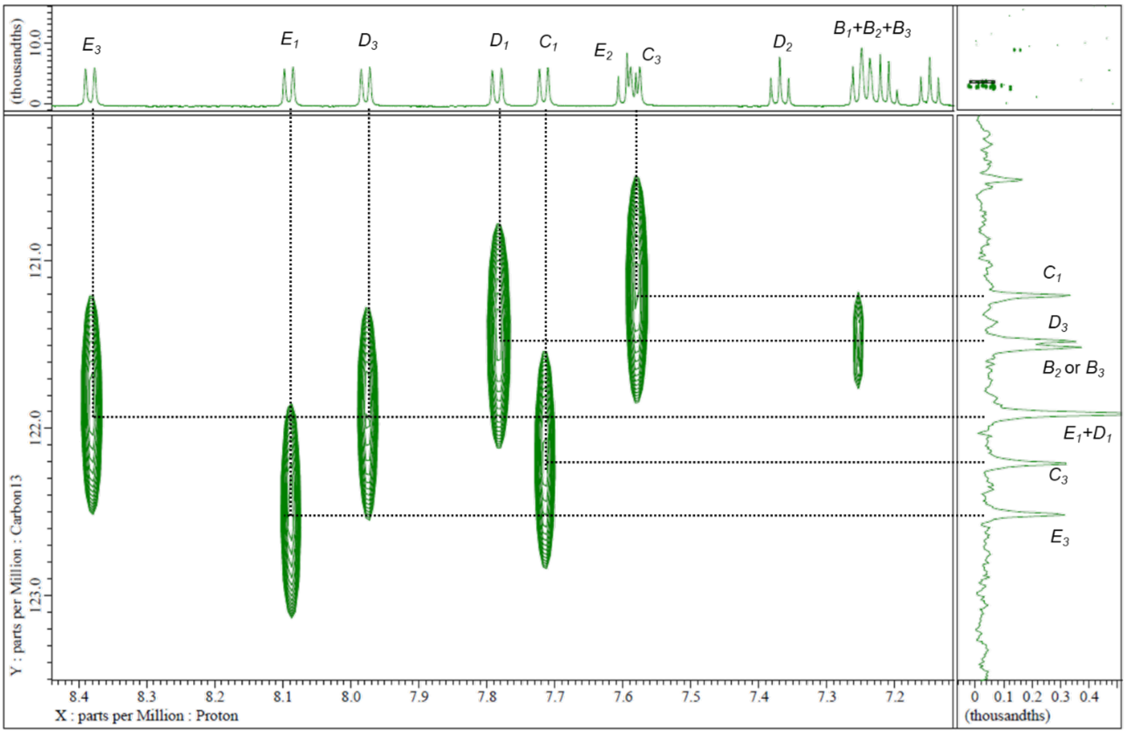

E3
E1
D3
D1
C1
E2
C3
D2
B1+B2+B3
C1
D3
B2 or B3
E1+D1
C3
E3
Y : parts per Million : Carbon13
X : parts per Million : Proton
(thousandths)

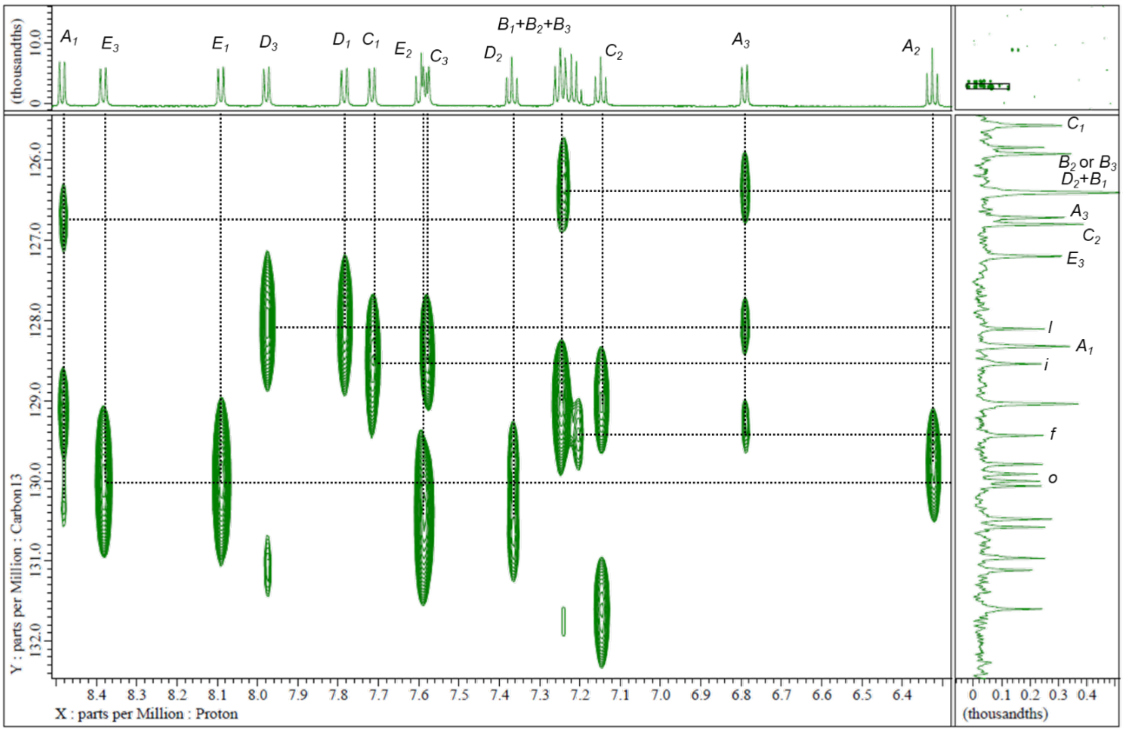

A1
E3
E1
D3
D1
C1
E2
C3
D2
B1+B2+B3
C2
A3
A2
C1
B2 or B3
D2+B1
A3
C2
E3
l
A1
i
f
o
Y : parts per Million : Carbon13
X : parts per Million : Proton
(thousandths)

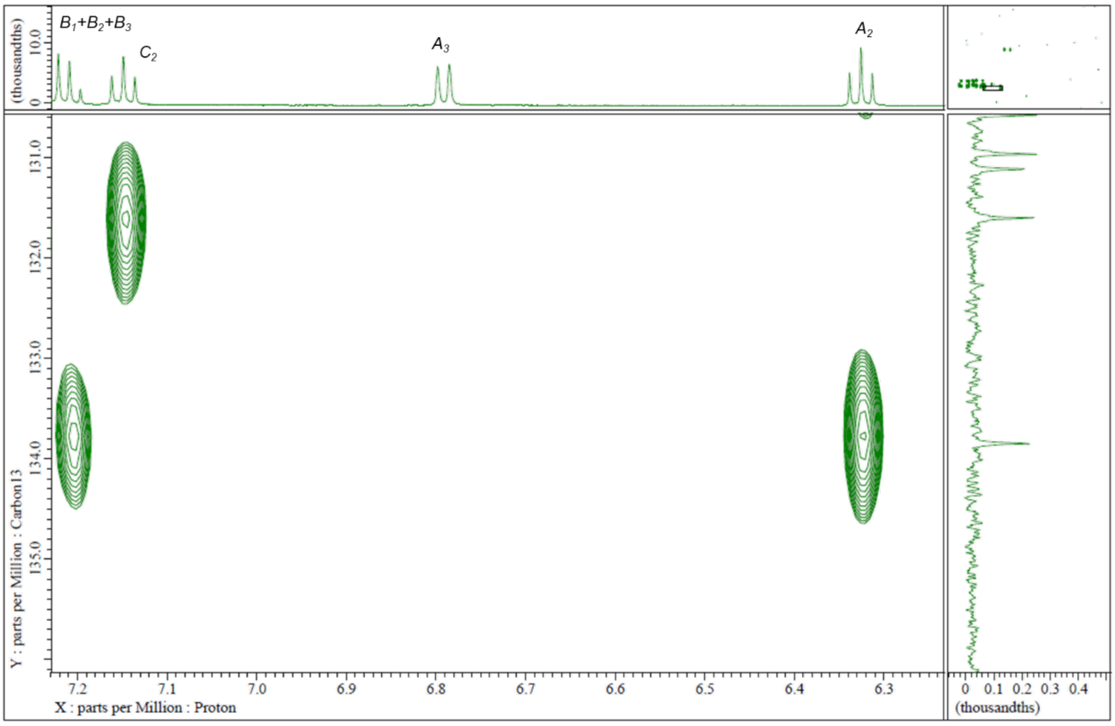

Supplementary Figure 69: $^{13}C/^{1}H$ HMBC spectra of 3 at room temperature (151 MHz, $C_2D_2Cl_4$ with a small excess amount of $N_2H_4 \cdot H_2O$)

4. Supplementary References

1. Nishino, H., Tsunoda, K. & Kurosawa, K. Manganese(III)-Mediated Formylation of Aromatic Compounds in the Presence of Malonic Acid. *Bull. Chem. Soc. Jpn.* **62**, 545–550 (1989).

2. Gaussian 16, Revision C.01, Frisch, M. J.; Trucks, G. W.; Schlegel, H. B.; Scuseria, G. E.; Robb, M. A.; Cheeseman, J. R.; Scalmani, G.; Barone, V.; Petersson, G. A.; Nakatsuji, H.; Li, X.; Caricato, M.; Marenich, A. V.; Bloino, J.; Janesko, B. G.; Gomperts, R.; Mennucci, B.; Hratchian, H. P.; Ortiz, J. V.; Izmaylov, A. F.; Sonnenberg, J. L.; Williams-Young, D.; Ding, F.; Lipparini, F.; Egidi, F.; Goings, J.; Peng, B.; Petrone, A.; Henderson, T.; Ranasinghe, D.; Zakrzewski, V. G.; Gao, J.; Rega, N.; Zheng, G.; Liang, W.; Hada, M.; Ehara, M.; Toyota, K.; Fukuda, R.; Hasegawa, J.; Ishida, M.; Nakajima, T.; Honda, Y.; Kitao, O.; Nakai, H.; Vreven, T.; Throssell, K.; Montgomery, J. A., Jr.; Peralta, J. E.; Ogliaro, F.; Bearpark, M. J.; Heyd, J. J.; Brothers, E. N.; Kudin, K. N.; Staroverov, V. N.; Keith, T. A.; Kobayashi, R.; Normand, J.; Raghavachari, K.; Rendell, A. P.; Burant, J. C.; Iyengar, S. S.; Tomasi, J.; Cossi, M.; Millam, J. M.; Klene, M.; Adamo, C.; Cammi, R.; Ochterski, J. W.; Martin, R. L.; Morokuma, K.; Farkas, O.; Foresman, J. B.; Fox, D. J. Gaussian, Inc., Wallingford CT, 2016.

3. Shao, Y., Gan, Z., Epifanovsky, E., Gilbert, A. T. B., Wormit, M., Kussmann, J., Lange, A. W., Behn, A., Deng, J., Feng, X., Ghosh, D., Goldey, M., Horn, P. R., Jacobson, L. D., Kaliman, I., Khaliullin, R. Z., Kuś, T., Landau, A., Liu, J., Proynov, E. I., Rhee, Y. M., Richard, R. M., Rohrdanz, M. A., Steele, R. P., Sundstrom, E. J., III, H. L. W., Zimmerman, P. M., Zuev, D., Albrecht, B., Alguire, E., Austin, B., Beran, G. J. O., Bernard, Y. A., Berquist, E., Brandhorst, K., Bravaya, K. B., Brown, S. T., Casanova, D., Chang, C.-M., Chen, Y., Chien, S. H., Closser, K. D., Crittenden, D. L., Diedenhofen, M., Jr, R. A. D., Do, H., Dutoi, A. D., Edgar, R. G., Fatehi, S., Fusti-Molnar, L., Ghysels, A., Golubeva-Zadorozhnaya, A., Gomes, J., Hanson-Heine, M. W. D., Harbach, P. H. P., Hauser, A. W., Hohenstein, E. G., Holden, Z. C., Jagau, T.-C., Ji, H., Kaduk, B., Khistyaev, K., Kim, J., Kim, J., King, R. A., Klunzinger, P., Kosenkov, D., Kowalczyk, T., Krauter, C. M., Lao, K. U., Laurent, A. D., Lawler, K. V., Levchenko, S. V., Lin, C. Y., Liu, F., Livshits, E., Lochan, R. C., Luenser, A., Manohar, P., Manzer, S. F., Mao, S.-P., Mardirossian, N., Marenich, A. V., Maurer, S. A., Mayhall, N. J., Neuscamman, E., Oana, C. M., Olivares-Amaya, R., O'Neill, D. P., Parkhill, J. A., Perrine, T. M., Peverati, R., Prociuk, A., Rehn, D. R., Rosta, E., Russ, N. J., Sharada, S. M., Sharma, S., Small, D. W., Sodt, A., Stein, T., Stück, D., Su, Y.-C., Thom, A. J. W., Tsuchimochi, T., Vanovschi, V., Vogt, L., Vydrov, O., Wang, T.,

Watson, M. A., Wenzel, J., White, A., Williams, C. F., Yang, J., Yeganeh, S., Yost, S. R., You, Z.-Q., Zhang, I. Y., Zhang, X., Zhao, Y., Brooks, B. R., Chan, G. K. L., Chipman, D. M., Cramer, C. J., III, W. A. G., Gordon, M. S., Hehre, W. J., Klamt, A., III, H. F. S., Schmidt, M. W., Sherrill, C. D., Truhlar, D. G., Warshel, A., Xu, X., Aspuru-Guzik, A., Baer, R., Bell, A. T., Besley, N. A., Chai, J.-D., Dreuw, A., Dunietz, B. D., Furlani, T. R., Gwaltney, S. R., Hsu, C.-P., Jung, Y., Kong, J., Lambrecht, D. S., Liang, W., Ochsenfeld, C., Rassolov, V. A., Slipchenko, L. V., Subotnik, J. E., Voorhis, T. V., Herbert, J. M., Krylov, A. I., Gill, P. M. W. & Head-Gordon, M. Advances in molecular quantum chemistry contained in the Q-Chem 4 program package. *Molecular Physics* **113**, 184–215 (2015).

4. Schleyer, P. von R., Maerker, C., Dransfeld, A., Jiao, H. & van Eikema Hommes, N. J. R. Nucleus-Independent Chemical Shifts: A Simple and Efficient Aromaticity Probe. *J. Am. Chem. Soc.* **118**, 6317–6318 (1996).

5. Chen, Z., Wannere, C. S., Corminboeuf, C., Puchta, R. & Schleyer, P. von R. Nucleus-Independent Chemical Shifts (NICS) as an Aromaticity Criterion. *Chem. Rev.* **105**, 3842–3888 (2005).

6. Herges, R. & Geuenich, D. Delocalization of Electrons in Molecules. *J. Phys. Chem. A* **105**, 3214–3220 (2001).

7. Geuenich, D., Hess, K., Köhler, F. & Herges, R. Anisotropy of the Induced Current Density (ACID), a General Method To Quantify and Visualize Electronic Delocalization. *Chem. Rev.* **105**, 3758–3772 (2005).

8. Koch, K.-H. & Müllen, K. Polyarylenes and Poly(arylenevinylene)s, V. Synthesis of Tetraalkyl-Substituted Oligo(1,4-naphthylene)s and Cyclization to Soluble Oligo(*peri*-naphthylene). *Chem. Ber.* **124**, 2091–2100 (1991).

9. Furche, F. *et al.* Circular Dichroism of Helicenes Investigated by Time-Dependent Density Functional Theory. *J. Am. Chem. Soc.* **122**, 1717–1724 (2000).

10. Schellman, J. A. Circular dichroism and optical rotation. *Chem. Rev.* **75**, 323–331 (1975).

11. Nakakuki, Y., Hirose, T., Sotome, H., Miyasaka, H. & Matsuda, K. Hexa-*peri*-hexabenzo[7]helicene: Homogeneously π-Extended Helicene as a Primary Substructure of Helically Twisted Chiral Graphenes. *J. Am. Chem. Soc.* **140**, 4317–4326 (2018).

12. Birks, J. B., Birch, D. J. S., Cordemans, E. & Vander Donckt, E. Fluorescence of the Higher Helicenes. *Chem. Phys. Lett.* **43**, 33–36 (1976).

13. Ware, W. R. & Cunningham, P. T. Fluorescence Lifetime and Fluorescence Enhancement of Perylene Vapor. *J. Chem. Phys.* **44**, 4364–4365 (1966).

14. Meyer, Y. H., Plaza, P. & Müllen, K. Ultrafast Spectroscopy of Soluble Terrylene and Quaterrylene. *Chem. Phys. Lett.* **264**, 643–648 (1997).

15. Cruz, C. M., Castro-Fernández, S., Maçôas, E., Cuerva, J. M. & Campaña, A. G. Undecabenzo[7]superhelicene: A Helical Nanographene Ribbon as a Circularly Polarized Luminescence Emitter. *Angew. Chem. Int Ed.* **57**, 14782–14786 (2018).

16. Wang, Y., Yin, Z., Zhu, Y., Gu, J., Li, Y. & Wang, J. Hexapole [9]Helicene. *Angew. Chem. Int Ed.* **58**, 587–591 (2019).

17. Hu, Y., Paternò, G. M., Wang, X.-Y., Wang, X.-C., Guizzardi, M., Chen, Q., Schollmeyer, D., Cao, X.-Y., Cerullo, G., Scotognella, F., Müllen, K. & Narita, A. π-Extended Pyrene-Fused Double [7]Carbohelicene as a Chiral Polycyclic Aromatic Hydrocarbon. *J. Am. Chem. Soc.* **141**, 12797–12803 (2019).

18. Qiu, Z., Ju, C.-W., Frédéric, L., Hu, Y., Schollmeyer, D., Pieters, G., Müllen, K. & Narita, A. Amplification of Dissymmetry Factors in p-Extended [7]- and [9]Helicenes. *J. Am. Chem. Soc.* **143**, 4661–4667 (2021).
